# The “cesium effect” magnified: exceptional chemoselectivity in cesium ion mediated nucleophilic reactions†

**DOI:** 10.1039/d4sc00316k

**Published:** 2024-03-06

**Authors:** Soumen Biswas, William B. Hughes, Luca De Angelis, Graham C. Haug, Ramon Trevino, Seth O. Fremin, Hadi D. Arman, Oleg V. Larionov, Michael P. Doyle

**Affiliations:** a Department of Chemistry, The University of Texas at San Antonio One UTSA Circle San Antonio TX 78249 USA oleg.larionov@utsa.edu michael.doyle@utsa.edu

## Abstract

Chemodivergent construction of structurally distinct heterocycles from the same precursors by adjusting specific reaction parameters is an emergent area of organic synthesis; yet, understanding of the processes that underpin the reaction divergence is lacking, preventing the development of new synthetic methods by systematically harnessing key mechanistic effects. We describe herein cesium carbonate-promoted oxadiaza excision cross-coupling reactions of β-ketoesters with 1,2,3-triazine 1-oxides that form pyridones in good to high yields, instead of the sole formation of pyridines when the same reaction is performed in the presence of other alkali metal carbonates or organic bases. The reaction can be further extended to the construction of synthetically challenging pyridylpyridones. A computational study comparing the effect of cesium and sodium ions in the oxadiaza excision cross-coupling reactions reveals that the cesium-coordinated species changes the reaction preference from attack at the ketone carbonyl to attack at the ester carbon due to metal ion-specific transition state conformational accommodation, revealing a previously unexplored role of cesium ions that may facilitate the development of chemodivergent approaches to other heterocyclic systems.

## Abstract 2

Synthetically challenging pyridones and pyridylpyridones can now be readily accessed from simple precursors in a chemodivergent oxadiaza excision cross-coupling enabled by an unprecedented conformational accommodation-driven cesium effect.

## Graphical abstract

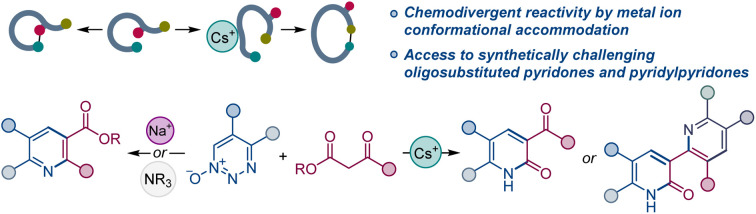

## Introduction

Development of new synthetic methods that enable construction of divergent products from the same reactants by adjusting specific reaction parameters, reagents, and ligands has emerged as a powerful strategy that can facilitate rapid access to unexplored chemical space.^1^

**Scheme 1:**
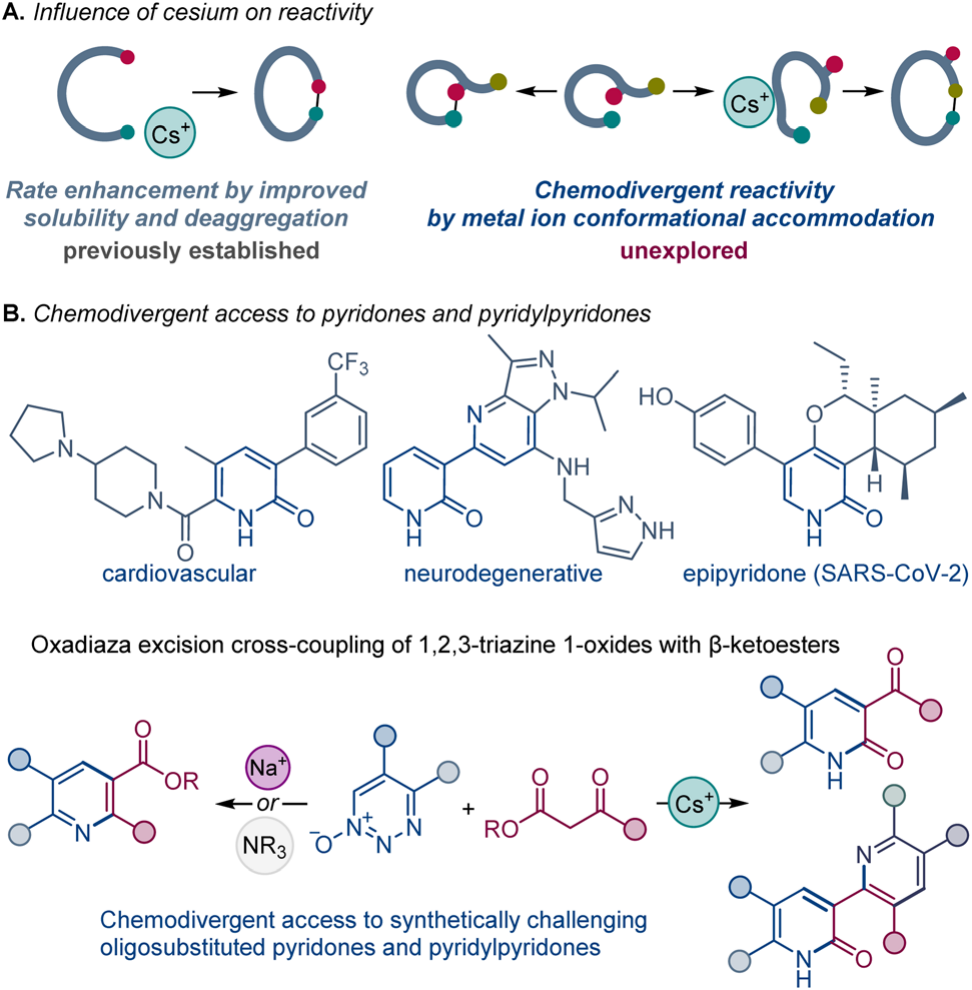
Chemodivergence and the influence of cesium on the oxadiaza excision cross-coupling of 1,2,3-triazine 1-oxides with β-ketoesters.

The beneficial influence of cesium salts on the rates of organic chemical reactions, leading to improved reaction efficiencies, is well known. Evidence from base-mediated ring closing reactions,^2^ which demonstrated the exceptional suitability of cesium over other alkali metals, initiated reports of similar phenomenon in a large number of diverse chemical transformations,^3,4^ commonly labeled as the “cesium effect” (Scheme 1A).^5^ In the long history of these special effects, the cesium effect has been confined to the enhancement of a specific process, rather than replacement of one process by another. More generally, the effect of the metal cation in base-catalyzed and base-mediated reactions remains poorly understood, and the specific cation-mediated processes underlying experimentally observed changes in the selectivity and reactivity of various reactions have not been systematically investigated, precluding rational design of reactions and catalytic systems based on well-defined cation–substrate interactions.

2-Pyridones possess a secondary amide moiety embedded in a 6-membered aromatic ring. They are a unique class of heterocycles that are more polar than their phenyl, pyridine, or phenol counterparts; and they are widely recognized in medicinal chemistry as bioisosteres for pyridine, phenol, and pyridine *N*-oxide groups, as well as bidentate hinge binding scaffolds, used as key design elements of diverse kinase inhibitors.^6^ Two main synthetic approaches have been used for their preparation: from other heterocyclic systems, mainly pyridines, and by condensation of acyclic systems.^7^ Notably, oligoheteroaryl systems containing pyridone units, *e.g.*, pyridylpyridones, have key roles in drug discovery and materials science,^8^ yet, they have remained synthetically challenging and are typically accessed by multistep synthetic sequences.^9^ Direct and modular methods of construction of pyridone-containing oligoheteroaryl systems that rely on simple precursors would facilitate rapid construction of libraries of structural analogues for structure–activity relationship studies.

We have been engaged in investigations of 1,2,3-triazine 1-oxides, whose formation from vinyldiazo compounds and *tert*-butyl nitrite occurs in high yield under mild conditions without catalysis.^10^ Our recent report showed that these heterocyclic compounds undergo a facile oxadiaza excision cross coupling with β-ketocarbonyl compounds to form oligosubstituted pyridines in high yields.^11^ The oxadiaza excision cross coupling reactions were particularly efficient with β-ketoesters, producing oligosubstituted pyridine derivatives (Scheme 1B) at room temperature with DBU as the base. The same product was formed with potassium carbonate as the base. The pyridine ring formation takes place by a nucleophilic attack at the β-ketoester ketone group instead of the ester group, although the mechanistic effects that underpin the observed chemoselectivity were unclear. Additionally, the alternative pathway that proceeds *via* an attack at the ester group and unlocks new synthetic route to 2-pyridones remained unknown.

We report herein that the chemoselectivity of the oxadiaza excision cross-coupling can be controlled by the effect of the base metal cation, enabling the previously elusive construction of 2-pyridones with cesium carbonate. We present evidence for this general, but unexpected, divergence in reactivity, including DFT calculations that point to the conformation-induced accommodation of the metal cation as the underlying cause of the switch in the reaction outcomes. Furthermore, we report the surprising *in situ* reaction between the cesium carbonate-formed pyridone with 1,2,3-triazine 1-oxides that enables facile access to a novel class of synthetically challenging pyridylpyridones by direct construction of up to four C–C/C–N bonds.

## Results and discussion

**Scheme 2:**
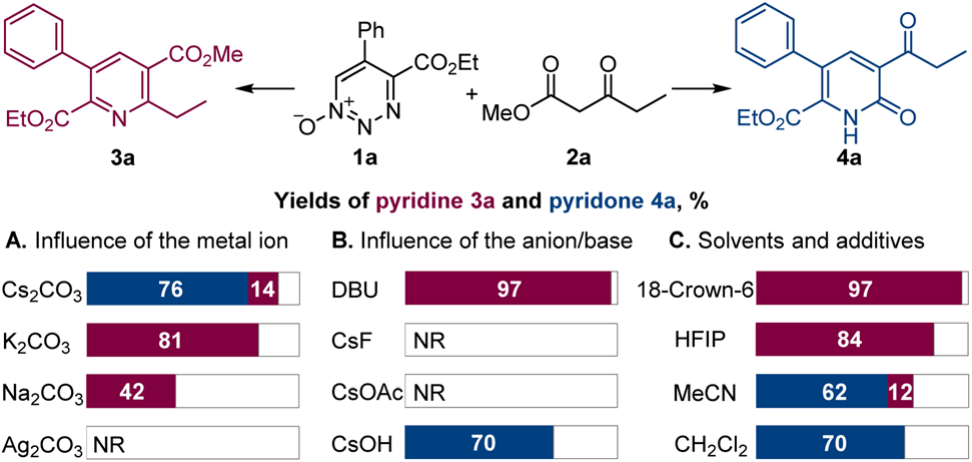
Influence of the reaction parameters on the oxadiaza excision cross-coupling of 1,2,3-triazine 1-oxide 1a with β-ketoester 2a. Reaction conditions: 1a (0.1 mmol), 2a (0.15 mmol), Cs_2_CO_3_ or other base (0.2 mmol), chloroform or other solvent (2 mL), rt. For other details, see Table S1 in the ESI.†

**Scheme 3:**
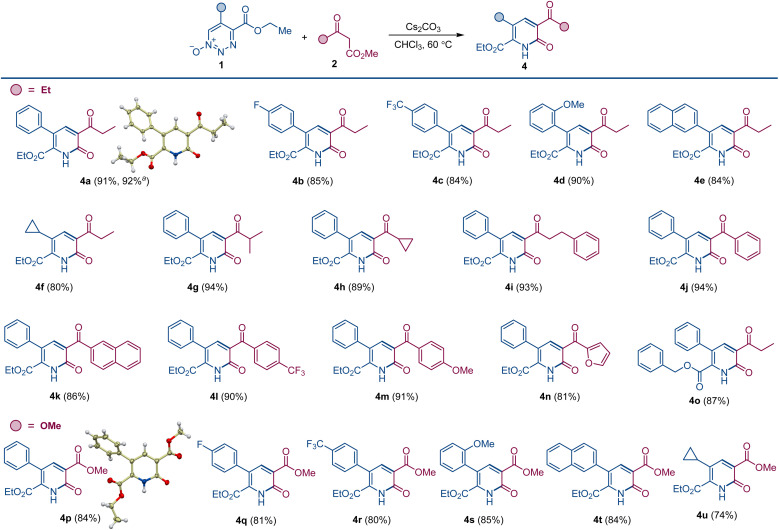
Construction of pyridones from 1,2,3-triazine 1-oxides and β-keto esters by oxadiaza excision cross-coupling. Reaction conditions: 1 (0.1 mmol), 2 (0.15 mmol), Cs_2_CO_3_ (0.2 mmol), CHCl_3_ (2 mL), 60 °C, 1–2 h. ^*a*^ 1.0 mmol scale.

Dropwise addition of 1,2,3-triazine 1-oxide 1a to a 50% molar excess of β-ketoester 2a using 2.0 equiv. of cesium carbonate, instead of DBU, in chloroform at room temperature resulted in the formation of pyridone 4a as the major product (Scheme 2). The structure of 4a was confirmed spectroscopically and from its X-ray crystal structure. This reaction was complete in less than 4 h, and pyridone 4a was easily separated from pyridine 3a by chromatography. Notably, when performed with K_2_CO_3_ or Na_2_CO_3_ as the base, the reactions required much longer reaction times to complete, and only pyridine 3a was formed, while no reaction was observed with Ag_2_CO_3_, pointing to a strong influence of the metal on the reaction outcome and efficiency. In addition, only 3a was produced with cesium carbonate in the presence of an equivalent amount of 18-crown-6 that coordinates strongly with the cesium ion.^12^ Similarly, use of the polar protic solvent hexafluoroisopropanol (HFIP) with cesium carbonate led to formation of pyridine 3a as the sole product, indicating that metal–substrate interactions that can be disrupted by solvent hydrogen bonding have an important role on reaction chemodivergence. Other aprotic solvents demonstrated similar preference for the pyridone formation with cesium carbonate as the base. On the other hand, less basic anions, *e.g.*, fluoride and acetate, failed to trigger the reaction, whereas cesium hydroxide proved to be a competent base, generating pyridone 4a. Pleasingly, increasing the reaction temperature in the cesium carbonate-promoted reactions caused an increase in the yield of pyridone 4a and a decrease in the amount of pyridine 3a. The optimum conditions for the reaction forming 4a used chloroform as the solvent with cesium carbonate as the base at 60 °C (Scheme 3). Importantly, the same substituted 1,2,3-triazine did not exhibit chemodivergence with this β-ketoester and, with cesium carbonate as the base, produced the pyridine derivative predominantly (91 : 9) at room temperature, suggesting involvement of a mechanistically distinct pathway in the 1,2,3-triazine-mediated reaction.

To evaluate the substrate scope and generality of this method for the formation of the pyridone scaffold under the developed conditions, we used representative 1,2,3-triazine 1-oxides 1 with various ketoesters 2; and the results are summarized in Scheme 3. Reactions were performed under optimum conditions in chloroform with cesium carbonate as the base at 60 °C. Yields of pyridones 4a–o are consistently high, and the alternative pyridine product was only detected in trace amount (<3%). A slight drop in yield was observed for triazine 1-oxides having an electron-withdrawing group on the aryl group at the 5-position (4b and 4c). Electronically neutral or donating groups provided the pyridone derivatives in very high yields (4a and 4d). Triazine 1-oxides with aliphatic substituents at the 5-position (except cyclopropyl, 4f) undergo ring opening through elimination of an α-proton attached to 1,2,3-triazine 1-oxide ring.^13^ A wide range of ketoesters were also examined, and there was no significant difference in pyridone yields with alkyl or aryl substituted ketoesters (4f–o). When dimethyl malonate was used as the β-ketoester coupling partner, pyridone derivatives bearing the ester group were produced. Thus, pyridones 4p–4u featuring aryl and alkyl substituents were isolated in good yields, and the structure of pyridone 4p was confirmed by X-ray crystallographic analysis.

**Scheme 4:**
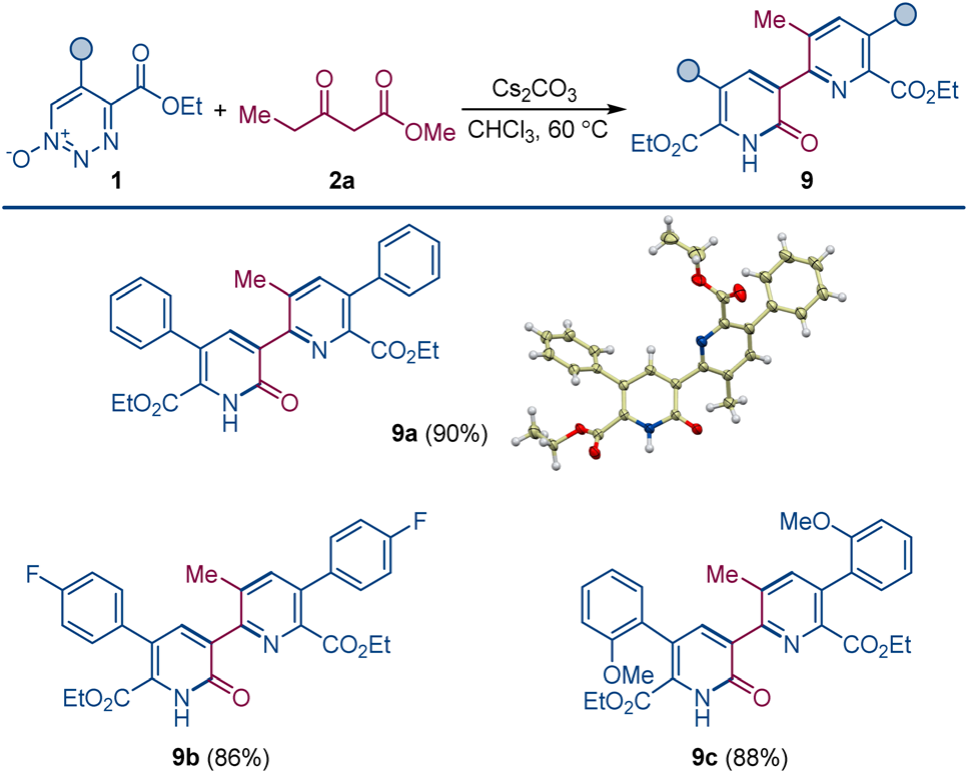
Pyridylpyridone construction by dual oxadiazo excision cross-coupling. Reaction conditions: 1 (0.1 mmol), 2a (0.05 mmol), Cs_2_CO_3_ (0.1 mmol), CHCl_3_ (1.5 mL), 60 °C, 4–6 h.

When the order of addition of 1,2,3-triazine 1-oxide and β-ketoester was reversed, a new compound was produced that was neither 2-pyridone 4 or pyridine 3. This new compound was identified to be pyridylpyridone 9, which is a combination of two molecules of 1,2,3-triazine 1-oxide and one molecule of β-ketoester with the loss of two molecules of N_2_O and one each of the β-ketoester alcohol and water. Indeed, when the amount of β-ketoester was made the limiting reagent and its amount reduced to 0.50 equiv., compound 9a was formed in 90% isolated yield (Scheme 4). This transformation takes advantage of the cesium effect to form pyridone 4 from the 1,2,3-triazine 1-oxide that, because of the acyl group at its position 3, is now primed to form the enolate that undergoes oxadiaza excision-mediated formation of the pyridylpyridone by reaction with a second 1,2,3-triazine 1-oxide. Pyridylpyridones are a diverse classification of relatively unexplored heterocyclic compounds in which pyridine substituents are attached from positions 2–6 onto the 2-pyridone template positions 1, 3–6.^14^ Most of these isomeric structures are unknown, but pyridine attachment from its position 2 to the 2-pyridone 1-position^15^ and to its 4- and 6-positions^16^ are well known.

Pyridylpyridone 9 provides a template for a pyridine substituted from its 2-position to the 3-position of the pyridone (2-pyridyl-3-pyridones) that can be easily built upon by common variations in the substituents of the reactants. Indeed, changing the substituents of the 1,2,3-triazine 1-oxide gives the corresponding symmetrically substituted pyridylpyridones 9a–c in similar high yield.

**Scheme 5:**
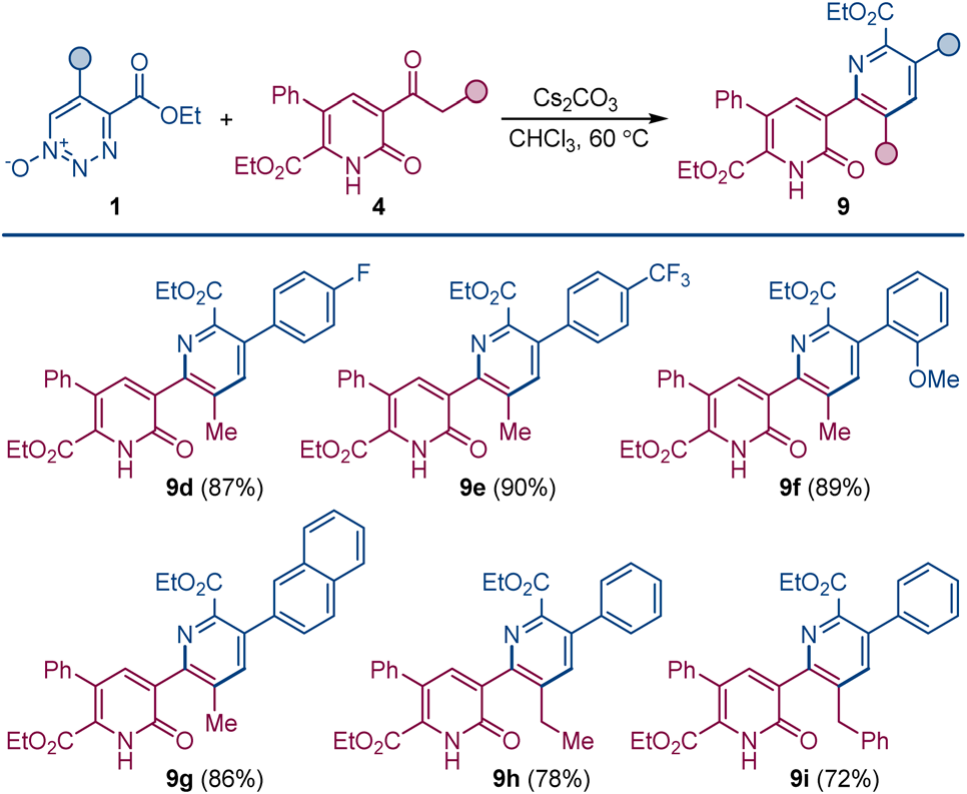
Construction of unsymmetrically substituted pyridylpyridones by oxadiazo excision cross-coupling of 1,2,3-triazine 1-oxides and pyridones. Reaction conditions: 1 (0.1 mmol), 4 (0.1 mmol), Cs_2_CO_3_ (0.2 mmol), CHCl_3_ (1 mL), 60 °C, 4–12 h.

In addition to the direct approach to symmetrically substituted 9a–c, a direct combination of 1,2,3-triazine 1-oxide with a 2-pyridone having different substituents allows the synthesis of unsymmetrically substituted pyridylpyridones 9d–i (Scheme 5).

**Fig. 1:**
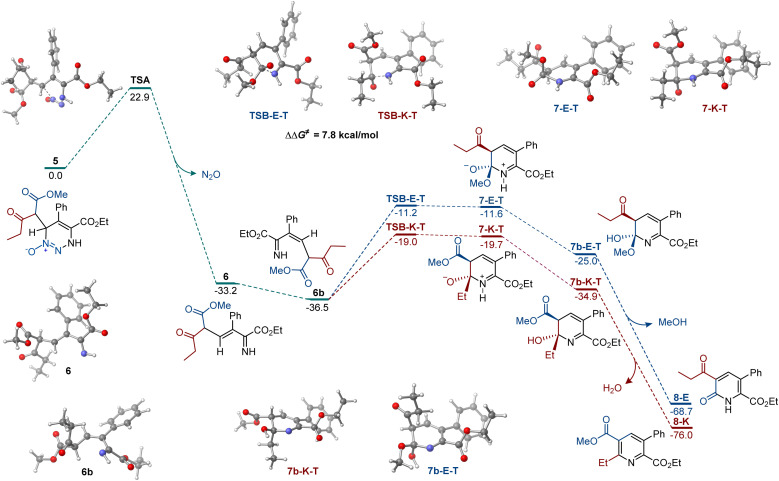
Computed Gibbs free energy profile of the chemodivergent formation of pyridine and pyridone products, Δ*G*, kcal mol^−1^.

Our prior reports established that carbon nucleophiles undergo addition to the 6-position of 5-substituted 4-carboxylato-1,2,3-triazine 1-oxides,^11,17^ and they point to the addition of the enolate to the 1,2,3-triazine *N*-oxide as the initial step in the reaction. With this information, we proceeded with the computational investigation of the divergent formation of pyridine and pyridone products in the presence of different bases (Fig. 1). To account for diastereomeric and conformational diversity, Boltzmann ensemble-averaged values were used for all intermediates and transition states. Consistent with previous computational studies of the reactions of 1,2,3-triazines and amidines,^18^ elimination of dinitrogen oxide from addition intermediate 5 proceeded over an accessible barrier TSA (Δ*G*^≠^ = 22.9 kcal mol^−1^), producing *E*-imine 6 that can undergo a thermodynamically favorable base-mediated isomerization to the more stable *Z*-imine 6b. In the absence of coordinating metals, the cyclization initiated by the nucleophilic addition of the imine nitrogen to the keto group is, kinetically, substantially more favorable (*cf.*, TSB-K-T and TSB-E-T, ΔΔ*G*^≠^ = 7.8 kcal mol^−1^). The pathways that lead to the zwitterionic *trans*-intermediates 7-K-T and 7-E-T are more kinetically favorable over the *cis*-pathways (Scheme S1†). Importantly, the kinetic preference for the nucleophilic attack at the keto group was also observed in the *cis*-pathways. Subsequent proton transfer produces hemiaminal 7b-K-T that is also more thermodynamically favored than the ester addition product 7b-E-T. Intermediates 7b-K-T and 7b-E-T can afford pyridine 8-E and pyridone 8-K by exergonic elimination of methanol (for 7b-K-T) and water (for 7b-E-T). These results are in agreement with the experimental data, indicating that pyridines are formed preferentially with organic bases.

**Fig. 2:**
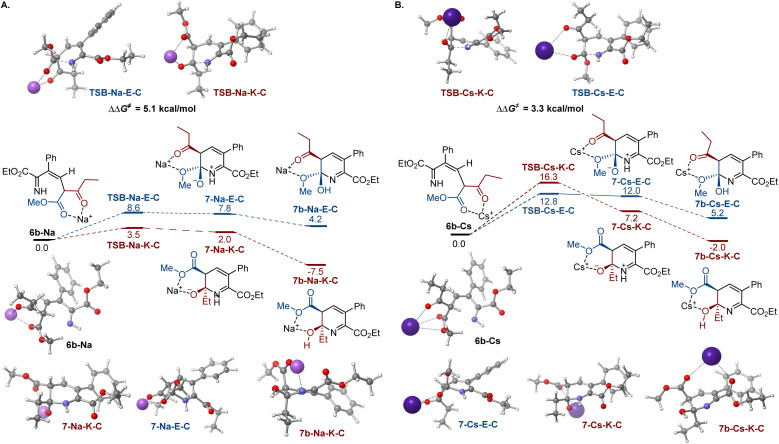
Influence of sodium (A) and cesium (B) ion chelation on the cyclization process, Δ*G*, kcal mol^−1^.

To gain insight into the effects of metal ions on the reaction, calculations were conducted for the cyclization step with sodium and cesium ions bound to imine 6b (Fig. 2). Notably, nucleophilic addition of the imine nitrogen to the keto group is more kinetically favored in the sodium-mediated pathway (ΔΔ*G*^≠^ = 5.1 kcal mol^−1^, TSB-Na-K-C*vs.*TSB-Na-E-C, Fig. 2A), leading to the pyridine product *via* a sequence of the cyclic zwitterion 7-Na-K-C and hemiaminal 7b-Na-K-C, in line with the experimental observations. The sodium coordination rendered the *cis*-cyclization the most kinetically favorable pathway, while the cyclization at the keto group remained kinetically favored in the *trans*-pathway (Scheme S2†). By contrast, the imine addition to the ester group proceeds with a lower barrier in the cesium-mediated *cis*-pathway (ΔΔ*G*^≠^ = 3.5 kcal mol^−1^, TSB-Cs-E-C*vs.*TSB-Cs-K-C, Fig. 2B), generating cyclic hemiaminal 7b-Cs-E-C as a major product. The kinetic preference for the nucleophilic attack at the ester group was also observed in the less kinetically favorable *trans*-pathway (Fig. S3†). These results point to a reversal of the reaction selectivity in favor of pyridone 8-E and are consistent with the selectivity observed experimentally.

**Fig. 3:**
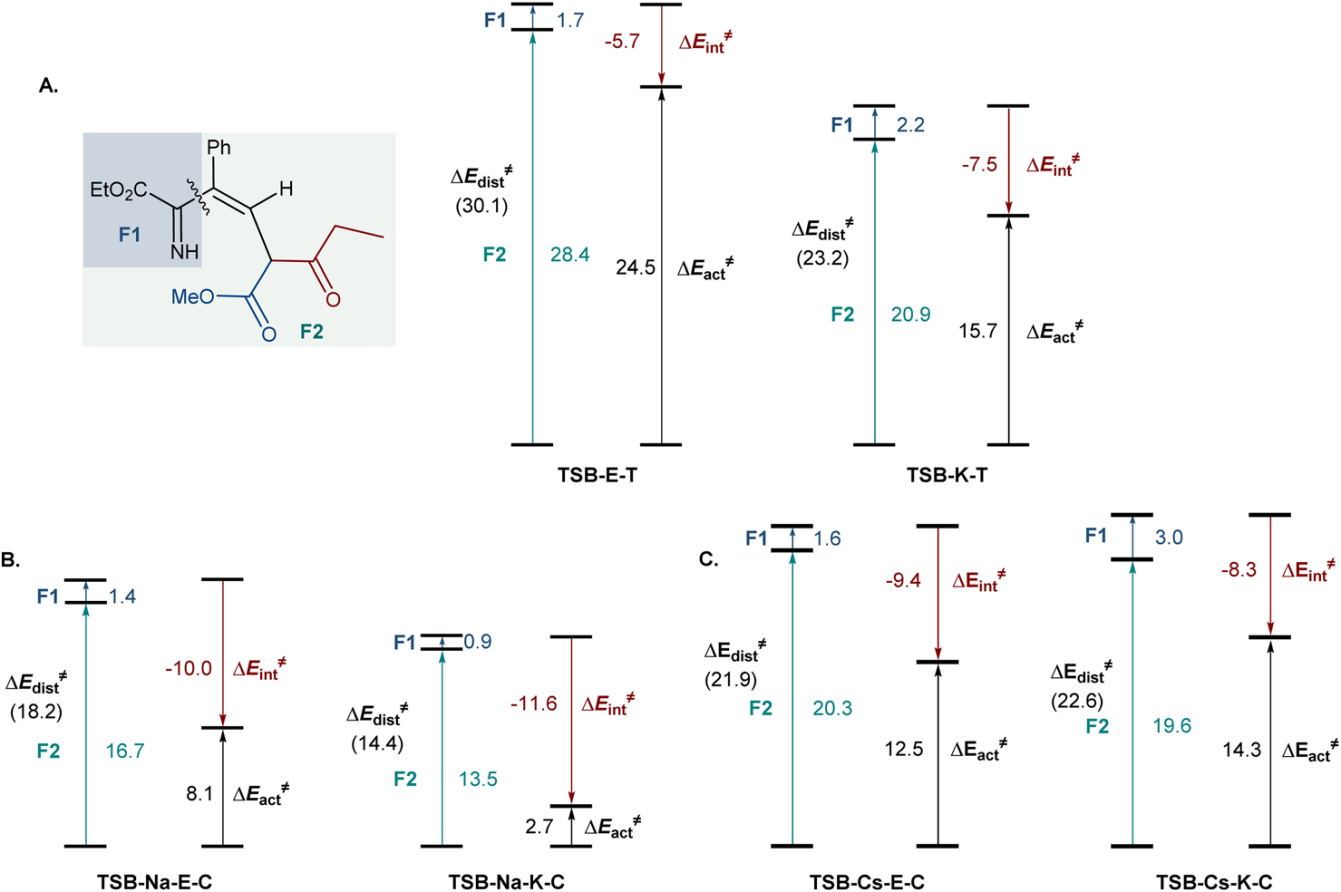
Distortion/interaction analysis of the cyclization transition states for the cyclization processes in the absence of metal ions (A), and with sodium (B) or cesium (C) chelation, Δ*E*, kcal mol^−1^.

To understand the effect of cesium ions that underlies the reversal of reaction selectivity, activation strain model (ASM) distortion/interaction analysis^19^ was conducted on cyclization transition states TSB in the three reaction modalities (no metal, sodium, and cesium). The analysis of both the kinetically more favored pathways (Fig. 3) and the less kinetically favored ones (Fig. S2†) in each modality indicates that the distortion in ketoester fragment F2 is substantially higher than in the smaller imine fragment F1 in all of the transition states, as it accommodates the proximal arrangement of the reacting carbonyl and imine nitrogen moieties. In the absence of a chelated metal ion, both transition states have similar interaction energies (Fig. 3A). However, the ester-centered transition state TSB-E-T has a significantly higher distortion energy, predominantly in the ketoester fragment, as it necessitates a larger intraannular bond angle at the ketoester β-carbon (115.8° in TSB-E-T*vs.* 111.8° in TSB-K-T). Interestingly, sodium ion chelation leads to a significant increase in the stabilizing interaction energies and a decrease in the distortion energies in both transition states (Fig. 3B). However, the stabilizing effect of sodium ion chelation does not change the kinetic preference for nucleophilic attack at the keto group, pointing to comparable influence of sodium chelation on both transition states. Interestingly, the beneficial effect of cesium ion chelation on interaction and distortion energies was less pronounced than for sodium (Fig. 3C) and commensurate with the lower charge density and larger ion radius of cesium.^20^ However, while the distortion energies were similar in TSB-Cs-E-C and TSB-Cs-K-C, the interaction energy was significantly higher for TSB-Cs-E-C, reflective of a more effective accommodation of the larger cation in TSB-Cs-E-C. The same trends were also observed in the less kinetically favored *trans*-pathway (Fig. S2C†).

These results indicate that divergent reaction outcomes can be achieved by exploiting differences in conformational accommodation of specifically selected chelated metal ions, pointing to potential applications in other systems with competing cyclization processes.

## Conclusions

In summary, chemodivergent outcomes of the oxadiaza excision cross-coupling of 1,2,3-triazine 1-oxides and β-ketoesters can be encoded by alkali metal ions, unlocking an efficient and direct construction of synthetically challenging oligosubstituted pyridones and pyridylpyridones from abundant precursors, requiring only cesium carbonate as a base. Notably, cesium carbonate almost exclusively generated pyridones, while other inorganic and organic bases provided the pyridine scaffold. The key difference in formation of pyridines and pyridones lies in the cyclization of the imine intermediate. The computational study comparing the effect of cesium and sodium ions in reactions with ketoesters reveals that the cesium-coordinated species is selective for attack of the ester carbon that gives rise to the pyridone derivative. In contrast, the sodium-coordinated species was selective for attack of the ketone carbon that favored formation of pyridine product. Further analysis pointed to key differences in the conformational accommodation of the metal ions, suggesting that new divergent outcomes can be systematically harnessed by exploiting differences in conformational accommodation of specifically selected chelated metal ions and underscoring potential applications in other systems with competing cyclization processes.

## Data availability

All experimental procedures, characterization data, NMR spectra for all new compounds, and details of the computational studies can be found in the ESI.†

## Author contributions

SB and LDA carried out the experiments. WBH, GCH, RT, and SOF performed the computational studies. HDA performed the X-ray crystallography studies. MPD and OVL conceived the project, wrote the manuscript, and co-wrote the ESI.† SB, LDA, WBH, GCH, RT, and SOF co-wrote the ESI† and contributed to writing the manuscript.

## Conflicts of interest

There are no conflicts to declare.

## Supplementary Material

SC-015-D4SC00316K-s001

SC-015-D4SC00316K-s002

SC-015-D4SC00316K-s003
