# Supplementary material for: The “cesium effect” magnified: exceptional chemoselectivity in cesium ion mediated nucleophilic reactions
Source: Chem Sci. 2024 Mar 6;15(14):5277–83. doi: 10.1039/d4sc00316k (PMC10988617; doi:10.1039/d4sc00316k)
Supplement: SC-015-D4SC00316K-s001 [file SC-015-D4SC00316K-s001.pdf]

Electronic Supplementary Material (ESI) for Chemical Science.
This journal is © The Royal Society of Chemistry 2024

Supporting information

The "Cesium Effect" Magnified: Exceptional Chemoselectivity in Cesium Ion Mediated Nucleophilic Reactions

Soumen Biswas, William B. Hughes, Luca De Angelis, Graham C. Haug, Ramon Trevino, Seth O. Fremin, Hadi D. Arman, Oleg V. Larionov*, and Michael P. Doyle*

Department of Chemistry, The University of Texas at San Antonio, San Antonio, Texas 78249, United States

*Email: oleg.larionov@utsa.edu, michael.doyle@utsa.edu

Table of Contents

1. General conditions S2

2. Optimization for the reaction conditions for the formation of pyridones S3

3. General procedure for the synthesis of 1,2,3-triazine 1-oxide compounds S4

4. General procedures for synthesis of pyridone and pyridylpyridone compounds S5

5. Analytical and spectral characterization data for products S6

6. Preparative scale reaction S15

7. Crystallographic data S15

8. Computational data S18

9. NMR spectra S85

10. References S115

1. General conditions

All reactions, unless noted, were performed in oven-dried (150 °C) glassware with magnetic stirring under an atmosphere of air. Analytical thin layer chromatography (TLC) was carried out using EM Science silica gel 60 F254 plates; visualization was accomplished with UV light (254 nm). Column chromatography was performed on CombiFlash® Rf200 and Rf+ purification systems using normal phase disposable columns. NMR spectra were recorded on a on a Bruker spectrometer (500 MHz and 300 MHz) and calibrated using the resonance signal of the residual undeuterated solvent for ^{1}H-NMR [δ_H = 7.26 ppm ($CDCl_3$)] and deuterated solvent for ^{13}C-NMR [δ_C = 77.16 ($CDCl_3$)] as an internal reference at 298 K. Spectra were reported as follows: chemical shift (δ ppm), multiplicity (Mi), coupling constants (Hz), integration and assignment. The peak information was described as: br = broad, s = singlet, d = doublet, t = triplet, q = quartet, dd = doublet of doublet, m = multiplet, and comp = composite of magnetically non-equivalent protons. ^{13}C-NMR spectra were collected on Bruker instruments (126 MHz and 75 MHz) with complete proton decoupling. High-resolution mass spectra (HRMS) were performed on a Bruker MicroTOFESI mass spectrometer with an ESI resource using CsI or LTQ ESI positive ion calibration solution as the standard. Tetrahydrofuran, dichloromethane, chloroform were purified using a JC-Meyer solvent purification system.

Materials: All β-keto-esters, DBU (1,8-diazabicyclo[5.4.0]undec-7-ene), DABCO (1,4-diaza-bicyclo[2.2.2]octane), DMAP (4-dimethylaminopyridine), Et_3N (triethylamine), Cs_2CO_3, CsOH, CsOAc, K_2CO_3, and Na_2CO_3 were purchased from Sigma Aldrich, TCI, and Alfa Aesar, and they were used without further purification. 1,2,3-Triazine-1-oxides were prepared by the reported literature procedure. [1,2]

1,2,3-Triazine 1-oxides used in the study

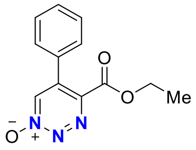

1a

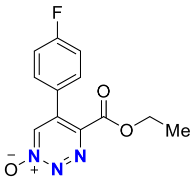

1b

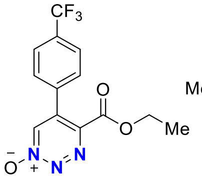

1c

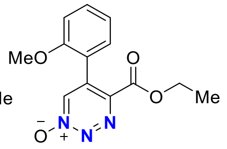

1d

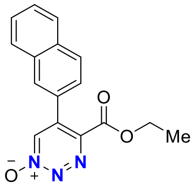

1e

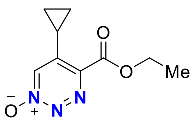

1f

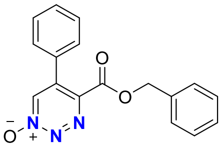

1g

2. Table S1. Optimization for the reaction conditions for the formation of pyridone compounds

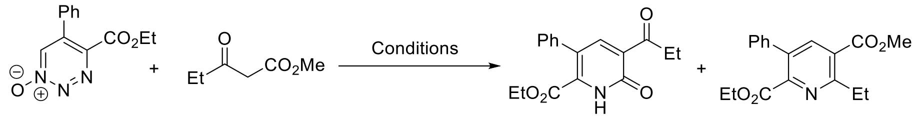

1a **2a** **4a** **3a**

Entry	Conditions	Base	Yield of **4a** (%)[a]	Yield of **3a** (%)[a]
1	$CHCl_3$, 4 h, rt	Cs_2CO_3	76	14
2	$CHCl_3$, 12 h, rt	K_2CO_3	-	81
3	$CHCl_3$, 72 h, rt	Na_2CO_3	-	42
4	$CHCl_3$, 72 h, 60 °C	Na_2CO_3	-	64
9b	$CHCl_3$, 24 h, rt	Ag_2CO_3	-	-
6	THF, 6 h, rt	Cs_2CO_3	53	35
7	DCM, 6 h, rt	Cs_2CO_3	70	20
8	ACN, 6 h, rt	Cs_2CO_3	62	12
9[c]	$CHCl_3$, 4 h, rt	Cs_2CO_3	64	18
10[d]	$CHCl_3$, 4 h, rt	Cs_2CO_3	75	15
11[e]	$CHCl_3$, 18-crown-6, 6 h, rt	Cs_2CO_3	-	72
12	HFIP, rt, 24 h	Cs_2CO_3	-	84
13	$CHCl_3$, 0 °C, 6 h	Cs_2CO_3	64	18
14[f]	$CHCl_3$, 60 °C, 2 h	Cs_2CO_3	86	<5
15[g]	$CHCl_3$, 60 °C, 1.5 h	Cs_2CO_3	93 (91)	trace
16[h]	$CHCl_3$, 6 h	CsF	NR	-
17[i]	$CHCl_3$, 6 h	CsOAc	NR	-
18	$CHCl_3$, 1 h	CsOH	70	trace
19	$CHCl_3$, rt, 24 h	Et_3N	-	13
20	$CHCl_3$, rt, 24 h	DMAP	-	95
21	$CHCl_3$, rt, 24 h	DABCO	-	93
22	$CHCl_3$, rt, 10 min	DBU	-	97

Reaction conditions: 1 mL solution of triazine 1-oxide **1a** (0.1 mmol) was added to a stirred solution of ketoester **2a** (0.15 mmol) and base (0.2 mmol) over 30 minutes. [a]NMR yields. Isolated yield in parenthesis. [b] 92% of **1a** was detected. [c]0.5 equiv. of Cs_2CO_3. [d] 1.0 equiv. of Cs_2CO_3. [e]2.0 equiv. of 18-crown-6 was used. [f] triazine 1-oxide **1a** was added at rt and then temperature was increased to 60 °C. [g] triazine 1-oxide **1a** was added to a preheated solution of ketoester **2a** (0.15 mmol) and Cs_2CO_3 at 60 °C. [h] 94% of **1a** was detected. [i]93% of **1a** was detected.

3. General procedure for the synthesis of 1,2,3-triazine 1-oxide compounds 1a-1g

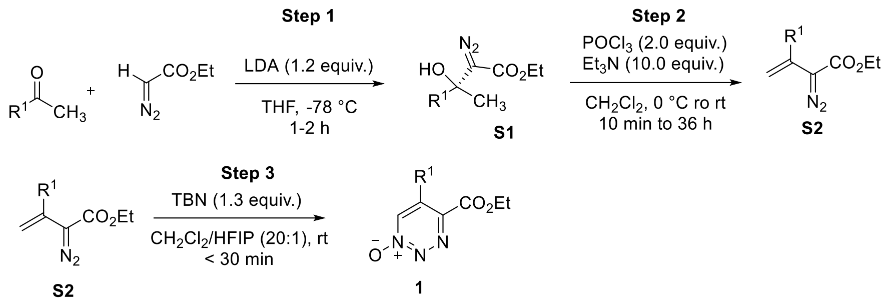

Step 1: Following the reported procedure.[1] To a solution of ketone (10.0 mmol, 1.00 equiv.) and ethyl diazoacetate (10.0 mmol, 1.00 equiv.) in 20 mL of dry THF at -78 ℃, was slowly added a solution of freshly prepared LDA (12.0 mmol, 1.20 equiv. 1.0 M in THF) over 30 minutes using a syringe pump. The resulting solution was quenched with water after stirring at -78 ℃ for 1-2 h. The reaction solution was extracted with ethyl acetate (3 x 15 mL), and the combined organic layer was washed with brine and then dried over anhydrous $MgSO_4$. After the solvent was evaporated, the crude product was purified by flash chromatography (% hexanes in ethyl acetate = 2%-10%) to give the β-hydroxy diazo **S1** compounds in 45%-83% yield. These compounds are stable at 0 ℃ and could be stored for months.

Step 2: Following the reported procedure.[1] To a solution of the **S1** compound (1.0 mmol, 1.0 equiv.) and Et_3N (1.4 mL, 10 mmol, 10 equiv.) in 10 mL CH_2Cl_2 at 0 ℃ was slowly added a solution of $POCl_3$ (300 mg, 2.0 mmol, 2 equiv.) in 4 mL of CH_2Cl_2 over 5 min. The reaction solution was warmed to room temperature, and the progress of the reaction was followed by TLC until consumption of the β-hydroxy diazo compound was complete. The reaction solution was quenched with water and extracted with ethyl acetate (3 x 10 mL), and the combined organic layer was dried over anhydrous $MgSO_4$. After the solvent was evaporated, the crude product was purified by flash chromatography (% ethyl acetate in hexanes = 2%-5%) to give the vinyl diazo **S2** compound in 50%-90% yield. These compounds are not stable, slowly undergoing intramolecular cycloaddition to pyrazoles, and were used immediately following their preparation.

Step 3: Following the reported procedure,[2] ^{t}BuONO (1.3 mmol, 1.3 equiv.) was added to 10 mL solution containing 20:1 v/v DCM:HFIPA (HFIPA = hexafluoroisopropyl alcohol), and the vinyl diazo compound **S2** (1.0 mmol, 1.0 equiv., 0.10 M in DCM) was added dropwise to the solution over 5 minutes. The reaction solution was stirred at room temperature under air for 1 h. After the solvent was evaporated, the crude product was purified by flash chromatography (% ethyl acetate in hexanes = 20%-50%) to give the 1,2,3-triazine 1-oxide **1** in 80%-99% yields. These compounds are bench stable and could be stored for months.

4. General procedures for synthesis of pyridone and pyridylpyridone compounds

Synthesis of pyridone compounds from 1,2,3-triazine 1-oxides and β-ketoesters.

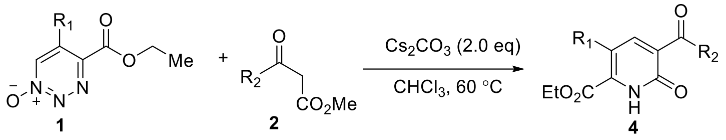

A chloroform solution (1.0 mL) of 1,2,3-triazine 1-oxide derivative **1** (0.1 mmol, 1 equiv.) was added dropwise over 30 minutes to a 1.0 mL solution of $CHCl_3$ containing β-keto ester **2** (0.15 mmol, 1.5 equiv.) and a Cs_2CO_3 (0.2 mmol, 65.2 mg) at 60 °C. The reaction was continued for 30-90 minutes at same temperature. After the completion of the reaction (monitored by TLC), the reaction mixture was cooled to room temperature, then diluted with 5.0 mL of dichloromethane. The organic layer was washed with saturated ammonium chloride solution (5.0 mL) followed by water and brine solution, then dried over anhydrous $MgSO_4$, filtered, and the solvent was evaporated under reduced pressure. The product mixture was purified by flash chromatography (ethyl acetate in hexanes = 20-60%) to give the pyridone compound **4** with yields of 80-94%.

Synthesis of symmetrical pyridylpyridones compounds from 1,2,3-triazine 1-oxides and β-ketoesters.

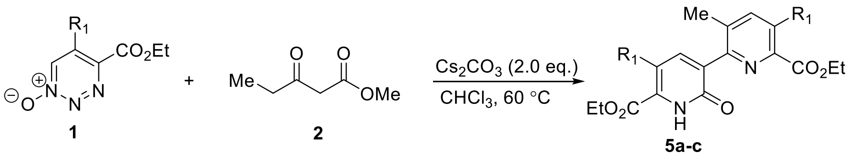

A chloroform solution (1.0 mL) of 1,2,3-triazine 1-oxide derivative **1** (0.1 mmol, 1 equiv.) was added dropwise over 30 minutes to a 0.5 mL solution of $CHCl_3$ containing β-keto ester **2** (0.05 mmol, 0.5 equiv.) and a Cs_2CO_3 (0.1 mmol, 32.5 mg) at 60 °C. The reaction was continued for 4-6 h at same temperature. After the completion of the reaction (monitored by TLC), the reaction mixture was cooled to room temperature, then diluted with 5.0 mL of dichloromethane. The organic layer was washed with saturated ammonium chloride solution (5.0 mL) followed by water and brine solution, then dried over anhydrous $MgSO_4$, filtered, and the solvent was evaporated under reduced pressure. The product mixture was purified by flash chromatography (ethyl acetate in hexanes = 30-70%) to give 2-pyridyl-3-pyridones **9a-c** with yields of 86-90%.

Synthesis of unsymmetrical pyridylpyridones compounds from 1,2,3-triazine 1-oxides and pyridones.

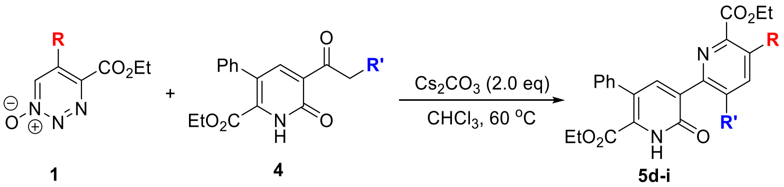

1,2,3-Triazine 1-oxide derivative **1** (0.1 mmol, 1.0 equiv.) was added all at once to a 1.0 mL solution of $CHCl_3$ containing pyridone **4** (0.1 mmol, 1.0 equiv.) and Cs_2CO_3 (0.2 mmol, 65.2 mg) at 60 °C. The reaction was continued for 4-12 h at same temperature. After the completion of the reaction (monitored by TLC), the reaction mixture was cooled to room temperature, then diluted with 5.0 mL of dichloromethane. The organic layer was washed with saturated ammonium chloride

solution (5.0 mL) followed by water and brine solution, then dried over anhydrous $MgSO_4$, filtered, and the solvent was evaporated under reduced pressure. The product mixture was purified by flash chromatography (ethyl acetate in hexanes = 30-70%) to give 2-pyridyl-3-pyridones **9d-i** with yields of 72-90%.

5. Analytical and spectral characterization data for products

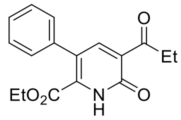

4a

Ethyl 6-Oxo-3-phenyl-5-propionyl-1,6-dihydropyridine-2-carboxylate, 4a: White solid (27.2 mg, 91% yield) 0.1 mmol scale reaction. Flash column chromatography conditions: hexane:ethyl acetate = 1:1. Mp 108-110 °C. **^{1}H NMR** (500 MHz, $CDCl_3$) δ 10.02 (br s, 1H), 8.15 (s, 1H), 7.48-7.37 (comp, 3H), 7.28 (d, *J* = 7.5 Hz, 2H), 4.19 (q, *J* = 7.2 Hz, 2H), 3.21 (q, *J* = 7.2 Hz, 2H), 1.21 (t, *J* = 7.2 Hz, 3H), 1.05 (t, *J* = 7.2 Hz, 3H). **^{13}C NMR** (126 MHz, $CDCl_3$) δ 201.2, 162.1, 160.6, 146.2, 137.5, 136.7, 129.0, 128.9, 128.4, 128.3, 125.4, 62.9, 35.6, 13.59, 8.0. **HRMS** (ESI) m/z: $[M + H]^+$ Calcd for $C_{17}H_{17}NO_4$ 300.1230; Found: 300.1229.

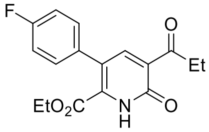

4b

Ethyl 3-(4-Fluorophenyl)-6-oxo-5-propionyl-1,6-dihydropyridine-2-carboxylate, 4b: White solid (26.9 mg, 85% yield) 0.1 mmol scale reaction. Flash column chromatography conditions: hexane:ethyl acetate = 1:1. Mp 142-144 °C. **^{1}H NMR** (500 MHz, $CDCl_3$) δ 9.95 (br s, 1H), 8.08 (s, 1H), 7.23 (d, *J* = 8.4 Hz, 2H), 7.10 (d, *J* = 8.4 Hz, 2H), 4.20 (q, *J* = 7.1 Hz, 2H), 3.19 (q, *J* = 7.2 Hz, 2H), 1.19 (t, *J* = 7.2 Hz, 3H), 1.08 (t, *J* = 7.1 Hz, 3H).**^{13}C NMR** (126 MHz, $CDCl_3$) δ 201.0, 162.8 (d, J_{C-F} = 240 Hz), 161.9, 160.4, 146.1, 132.7, 132.6, 130.8, 130.7 (d, J_{C-F} = 8 Hz), 124.3, 115.4 (d, J_{C-F} = 21 Hz), 63.1, 35.8, 13.7, 8.0. **^{19}F NMR** (471 MHz, $CDCl_3$) δ -113.5. **HRMS** (ESI) m/z: $[M + H]^+$ Calcd for $C_{17}H_{16}FNO_4$ 318.1136; Found: 318.1134.

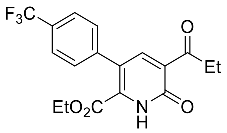

4c

Ethyl 6-Oxo-5-propionyl-3-(4-trifluoromethylphenyl)-1,6-dihydropyridine-2-carboxylate, 4c; White solid (30.8 mg, 84% yield) 0.1 mmol scale reaction. Flash column chromatography conditions: hexane:ethyl acetate = 1:1. Mp 165-167 °C. **^{1}H NMR** (500 MHz, $CDCl_3$) 10.31 (br s, 1H), 8.09 (s, 1H), 7.67 (d, *J* = 7.9 Hz, 2H), 7.39 (d, *J* = 7.9 Hz, 2H), 4.19 (q, *J* = 7.1 Hz, 2H), 3.19 (q, *J* = 7.1 Hz, 2H), 1.19 (t, *J* = 7.1 Hz, 3H), 1.03 (t, *J* = 7.1 Hz, 3H).**^{13}C NMR** (126 MHz, $CDCl_3$) δ 200.8, 161.5, 160.5, 145.7, 140.5, 130.5 (q, J_{C-F} = 32 Hz), 129.4, 125.4 (q, J_{C-F} = 3 Hz), 125.2, 123.7, 123.4 (q, J_{C-F} = 289 Hz), 123.0, 63.2, 35.8, 13.5, 8.0. **^{19}F NMR** (471 MHz, $CDCl_3$) δ -62.7. **HRMS** (ESI) m/z: $[M + H]^+$ Calcd for $C_{18}H_{16}F_3NO_4$ 368.1104; Found: 368.1102.

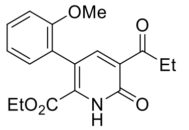

4d

Ethyl 3-(2-Methoxyphenyl)-6-oxo-5-propionyl-1,6-dihydropyridine-2-carboxylate, 4d; White solid (29.6 mg, 90% yield) 0.1 mmol scale reaction. Flash column chromatography conditions: hexane:ethyl acetate = 1:1. Mp 140-142 °C. **^{1}H NMR** (500 MHz, $CDCl_3$) δ 10.27 (br s, 1H), 8.15 (s, 1H), 7.33 (t, *J* = 7.5 Hz, 1H), 6.95 (comp, 1H), 6.89-6.78 (comp, 2H), 4.21 (q, *J* = 7.1 Hz, 2H), 3.84 (s, 3H), 3.20 (q, *J* = 7.1 Hz, 2H), 1.21 (t, *J* = 7.1 Hz, 3H), 1.08 (t, *J* = 7.1 Hz, 3H). **^{13}C NMR** (126 MHz, $CDCl_3$) δ 201.3, 162.2, 160.7, 159.6, 146.0, 145.9, 137.9, 129.5, 129.4, 125.2, 121.3, 114.6, 113.7, 62.9, 55.5, 35.6, 13.6, 8.0. **HRMS** (ESI) m/z: $[M + H]^+$ Calcd for $C_{18}H_{19}NO_5$ 330.1336; Found: 330.1334.

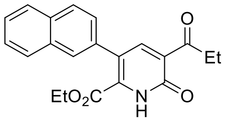

4e

Ethyl 3-(Naphthalen-2-yl)-6-oxo-5-propionyl-1,6-dihydropyridine-2-carboxylate, 4e; White solid (32.1 mg, 92% yield) 0.1 mmol scale reaction. Flash column chromatography conditions: hexane:ethyl acetate = 1:1. Mp 155-157 °C. **^{1}H NMR** (500 MHz, $CDCl_3$) δ 10.41 (br s, 1H), 8.23 (s, 1H), 7.89-7.83 (comp, 3H), 7.74 (s, 1H), 7.55-7.51 (comp, 2H), 7.36 (d, *J* = 8.3 Hz, 1H), 4.15 (q, *J* = 7.1 Hz, 2H), 3.21 (q, *J* = 7.2 Hz, 2H), 1.20 (t, *J* = 7.1 Hz, 3H), 0.93 (t, *J* = 7.2 Hz, 3H). **^{13}C NMR** (126 MHz, $CDCl_3$) δ 201.2, 162.0, 160.6, 146.4, 134.1, 133.1, 132.9, 128.1, 128.0, 127.9, 127.9, 127.8, 127.0, 126.9, 126.7, 126.7, 125.3, 63.0, 35.8, 13.6, 8.0. **HRMS** (ESI) m/z: $[M + H]^+$ Calcd for $C_{21}H_{19}NO_4$ 350.1387; Found: 350.1385.

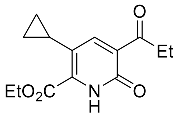

4f

Ethyl 3-Cyclopropyl-6-oxo-5-propionyl-1,6-dihydropyridine-2-carboxylate, **4f**; White solid (21.0 mg, 80% yield) 0.1 mmol scale reaction. Flash column chromatography conditions: hexane:ethyl acetate = 1:1. Mp 98-100 °C. **^{1}H NMR** (500 MHz, $CDCl_3$) δ 9.93 (br s, 1H), 7.79 (s, 1H), 4.46 (q, *J* = 7.1 Hz, 2H), 3.16 (q, *J* = 7.1 Hz, 2H), 2.56 (tt, *J* = 8.6, 4.4 Hz, 1H), 1.43 (t, *J* = 7.1 Hz, 3H), 1.15 (t, *J* = 7.1 Hz, 3H), 1.08- 0.97 (comp, 2H), 0.72-0.70 (comp, 2H). **^{13}C NMR** (126 MHz, $CDCl_3$) δ 200.7, 161.4, 159.6, 142.8, 127.9, 126.8, 126.5, 63.2, 36.2, 14.3, 11.2, 8.1, 7.9. **HRMS** (ESI) m/z: $[M + H]^+$ Calcd for $C_{14}H_{17}NO_4$ 264.1230; Found: 264.1227.

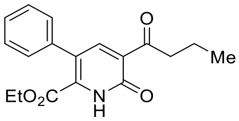

4g

Ethyl 5-Butyryl-6-oxo-3-phenyl-1,6-dihydropyridine-2-carboxylate, 4g; White solid (29.4 mg, 94% yield) 0.1 mmol scale reaction. Flash column chromatography conditions: hexane:ethyl

acetate = 2:1. Mp 128-130 °C. **^{1}H NMR** (500 MHz, $CDCl_3$) δ 10.46 (br s, 1H), 8.14 (s, 1H), 7.51-7.33 (comp, 3H), 7.28 (dd, *J* = 6.7, 2.5 Hz, 2H), 4.19 (q, *J* = 7.1 Hz, 2H), 3.16 (t, *J* = 7.1 Hz, 2H), 1.77-1.73 (comp, 2H), 1.06 (t, *J* = 7.1 Hz, 3H), 1.00 (t, *J* = 7.2 Hz, 3H). **^{13}C NMR** (126 MHz, $CDCl_3$) δ 200.8, 162.2, 160.7, 146.1, 136.7, 128.8, 128.7, 128.5, 128.4, 128.3, 125.4, 62.9, 44.0, 17.4, 13.9, 13.6. **HRMS** (ESI) m/z: $[M + H]^+$ Calcd for $C_{18}H_{19}NO_4$ 314.1387; Found: 314.1385.

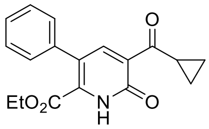

4h

Ethyl 5-(Cyclopropanecarbonyl)-6-oxo-3-phenyl-1,6-dihydropyridine-2-carboxylate, 4h; White solid (27.7 mg, 89% yield) 0.1 mmol scale reaction. Flash column chromatography conditions: hexane:ethyl acetate = 1:1. Mp 136-138 °C. **^{1}H NMR** (500 MHz, $CDCl_3$) δ 10.74 (br s, 1H), 8.14 (s, 1H), 7.43-7.40 (comp, 3H), 7.34-7.24 (comp, 2H), 4.19 (q, *J* = 7.1 Hz, 2H), 3.26-3.23 (m, 1H), 1.30-1.28 (comp, 2H), 1.15-1.12 (comp, 2H), 1.05 (t, *J* = 7.1 Hz, 3H). **^{13}C NMR** (126 MHz, $CDCl_3$) δ 201.0, 162.3, 161.1, 145.7, 145.6, 136.7, 128.8, 128.4, 128.3, 128.2, 125.5, 62.8, 19.6, 13.7, 13.6. **HRMS** (ESI) m/z: $[M + H]^+$ Calcd for $C_{18}H_{17}NO_4$ 312.1230; Found: 312.1229.

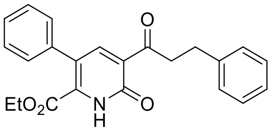

4i

Ethyl 6-Oxo-3-phenyl-5-(3-phenylpropanoyl)-1,6-dihydropyridine-2-carboxylate, **4i**; White solid (34.9 mg, 93% yield) 0.1 mmol scale reaction. Flash column chromatography conditions: hexane:ethyl acetate = 1:1. Mp 148-150 °C. **^{1}H NMR** (500 MHz, $CDCl_3$) δ 10.10 (br s, 1H), 8.13 (s, 1H), 7.45 – 7.38 (comp, 3H), 7.32 – 7.23 (comp, 6H), 7.21 (dd, *J* = 6.7, 2.1 Hz, 1H), 4.16 (q, *J* = 7.1 Hz, 2H), 3.53 (t, *J* = 7.5 Hz, 2H), 3.06 (t, *J* = 7.5 Hz, 2H), 1.03 (t, *J* = 7.1 Hz, 3H). **^{13}C NMR** (126 MHz, $CDCl_3$) δ 199.3, 161.7, 160.2, 146.6, 141.0, 136.5, 129.2, 128.7, 128.7, 128.6, 128.4, 128.3, 128.2, 126.0, 125.0, 62.9, 43.9, 29.9, 13.4. **HRMS** (ESI) m/z: $[M + H]^+$ Calcd for $C_{23}H_{21}NO_4$ 376.1543; Found: 376.1542.

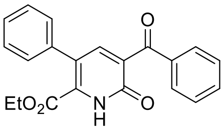

4j

Ethyl 5-Benzoyl-6-oxo-3-phenyl-1,6-dihydropyridine-2-carboxylate, 4j; White solid (32.7 mg, 94% yield) 0.1 mmol scale reaction. Flash column chromatography conditions: hexane:ethyl acetate = 1:1. Mp 140-142 °C. **^{1}H NMR** (500 MHz, $CDCl_3$) δ 10.81 (br s, 1H), 7.91 (d, *J* = 7.6 Hz, 2H), 7.76 (s, 1H), 7.62 (t, *J* = 7.6 Hz, 1H), 7.50 (t, *J* = 7.6 Hz, 2H), 7.42-7.40 (comp, 3H), 7.34 – 7.25 (comp, 2H), 4.14 (q, *J* = 7.1 Hz, 2H), 1.03 (t, *J* = 7.1 Hz, 3H). **^{13}C NMR** (126 MHz, $CDCl_3$) δ 193.9, 161.6, 160.0, 145.4, 136.6, 136.4, 135.4, 133.7, 132.5, 129.8, 128.8, 128.7, 128.4, 128.3, 124.9, 62.9, 13.6. **HRMS** (ESI) m/z: $[M + H]^+$ Calcd for $C_{21}H_{17}NO_4$ 348.1230; Found: 348.1228.

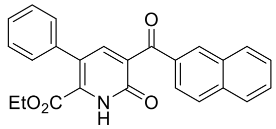

4k

Ethyl 5-(2-Naphthoyl)-6-oxo-3-phenyl-1,6-dihydropyridine-2-carboxylate, 4k; White solid (34.1 mg, 86% yield) 0.1 mmol scale reaction. Flash column chromatography conditions: hexane:ethyl acetate = 1:1. Mp 163-165 °C. **^{1}H NMR** (500 MHz, $CDCl_3$) δ 10.61 (br s, 1H), 8.39 (s, 1H), 8.04 – 7.87 (comp, 4H), 7.82 (s, 1H), 7.62-7.58 (comp, 2H), 7.43-7.38 (comp, 3H), 7.35 – 7.25 (comp, 2H), 4.13 (q, J = 7.1 Hz, 2H), 1.02 (t, J = 7.1 Hz, 3H). **^{13}C NMR** (126 MHz, $CDCl_3$) δ 193.7, 161.5, 160.0, 145.4, 136.5, 136.0, 135.0, 133.8, 133.2, 132.5, 132.1, 129.8, 128.9, 128.8, 128.5, 128.4, 128.3, 128.3, 127.9, 126.9, 124.8, 62.9, 13.5. **HRMS** (ESI) m/z: $[M + H]^+$ Calcd for $C_{25}H_{19}NO_4$ 398.1387; Found: 398.1387.

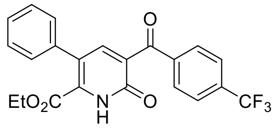

4l

Ethyl 6-Oxo-3-phenyl-5-(4-(trifluoromethyl)benzoyl)-1,6-dihydropyridine-2-carboxylate, 4l; White solid (37.3 mg, 90% yield) 0.1 mmol scale reaction. Flash column chromatography conditions: hexane:ethyl acetate = 1:1. Mp 176-178 °C. **^{1}H NMR** (500 MHz, $CDCl_3$) δ 10.68 (br s, 1H), 8.01 (d, J = 8.0 Hz, 2H), 7.85 (s, 1H), 7.76 (d, J = 8.0 Hz, 2H), 7.43-7.41 (comp, 3H), 7.32 – 7.26 (comp, 2H), 4.14 (q, J = 7.2 Hz, 2H), 1.01 (t, J = 7.2 Hz, 3H). **^{13}C NMR** (126 MHz, $CDCl_3$) δ 192.7, 161.3, 159.6, 146.7, 139.4, 136.3, 135.0, 138.8, 134.6 132.5, 129.9, 128.9, 128.5, 125.6 (q, $J_{C\text{-}F}$ = 4 Hz), 124.7, 123.5 (q, $J_{C\text{-}F}$ = 260 Hz), 63.1, 13.5. **HRMS** (ESI) m/z: $[M + H]^+$ Calcd for $C_{22}H_{16}F_3NO_4$ 416.1104; Found: 416.1102.

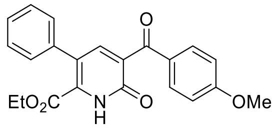

4m

Ethyl 5-(4-Methoxybenzoyl)-6-oxo-3-phenyl-1,6-dihydropyridine-2-carboxylate, 4m; White solid (34.3, 91% yield) 0.1 mmol scale reaction. Flash column chromatography conditions: hexane:ethyl acetate = 1:1. Mp 168-170 °C. **^{1}H NMR** (500 MHz, $CDCl_3$) δ 10.40 (br s, 1H), 7.91 (d, J = 8.4 Hz, 2H), 7.71 (s, 1H), 7.46 – 7.37 (comp, 3H), 7.34 – 7.26 (comp, 2H), 6.97 (d, J = 8.4 Hz, 2H), 4.17 (q, J = 7.1 Hz, 2H), 3.90 (s, 3H), 1.04 (t, J = 7.1 Hz, 3H). **^{13}C NMR** (126 MHz, $CDCl_3$) δ 192.0, 164.3, 161.6, 159.7, 144.9, 136.7, 134.2, 133.6, 132.4, 129.3, 128.9, 128.4, 128.3, 125.0, 114.0, 77.4, 62.9, 55.7, 13.6. **HRMS** (ESI) m/z: $[M + H]^+$ Calcd for $C_{22}H_{20}NO_5$ 378.1336; Found: 378.1334.

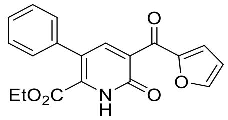

4n

Ethyl 5-(furan-2-carbonyl)-6-oxo-3-phenyl-1,6-dihydropyridine-2-carboxylate, 4n; White solid (27.3 mg, 81% yield) 0.1 mmol scale reaction,. Flash column chromatography conditions: hexane:ethyl acetate = 1:1. Mp 102-104 °C. **^{1}H NMR** (500 MHz, $CDCl_3$) δ 10.19 (br s, 1H), 7.98 (s, 1H), 7.68 (s, 1H), 7.41-7.31 (comp, 4H), 7.29-7.27 (comp, 2H), 6.60 (d, *J* = 4.3 Hz, 1H), 4.18 (q, *J* = 7.2 Hz, 2H), 1.03 (t, *J* = 7.2 Hz, 3H). **^{13}C NMR** (126 MHz, $CDCl_3$) δ 179.8, 161.9, 159.8, 152.1, 147.9, 145.5, 136.7, 131.1, 129.1, 128.9, 128.5, 128.5, 125.3, 121.3, 112.9, 63.0, 13.6. **HRMS** (ESI) m/z: $[M + H]^+$ Calcd for $C_{19}H_{16}NO_5$ 338.1023; Found: 338.1022.

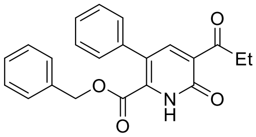

4o

Benzyl 6-oxo-3-phenyl-5-propionyl-1,6-dihydropyridine-2-carboxylate, 4o; White solid (31.4 mg, 87% yield) 0.1 mmol scale reaction. Flash column chromatography conditions: hexane:ethyl acetate = 1:1. Mp 158-160 °C. **^{1}H NMR** (500 MHz, $CDCl_3$) δ 9.82 (br s, 1H), 8.10 (s, 1H), 7.38 – 7.25 (comp, 6H), 7.21 (d, *J* = 7.1 Hz, 2H), 6.99 (d, *J* = 7.1 Hz, 2H), 5.14 (s, 2H), 3.17 (q, *J* = 7.2 Hz, 2H), 1.19 (t, *J* = 7.2 Hz, 3H). **^{13}C NMR** (126 MHz, $CDCl_3$) δ 201.2, 162.2, 160.8, 146.1, 146.0, 136.5, 133.9, 128.8, 128.7, 128.6, 128.6, 128.5, 128.4, 128.3, 125.5, 68.6, 35.6, 8.0. **HRMS** (ESI) m/z: $[M + H]^+$ Calcd for $C_{22}H_{20}NO_4$ 362.1387; Found: 362.1387.

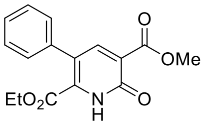

4p

2-Ethyl 5-methyl 6-oxo-3-phenyl-1,6-dihydropyridine-2,5-dicarboxylate, 4p; White solid, (25.3 mg, 84% yield), 0.1 mmol scale reaction,. Flash column chromatography conditions: hexane:ethyl acetate = 1:1. mp 103-105 °C. **^{1}H NMR** (500 MHz, $CDCl_3$) δ 10.77 (br s, 1H), 8.25 (s, 1H), 7.48 – 7.38 (comp, 3H), 7.32-7.29 (comp, 2H), 4.21 (q, *J* = 7.1 Hz, 2H), 3.99 (s, 3H), 1.09 (t, *J* = 7.1 Hz, 3H). **^{13}C NMR** (126 MHz, $CDCl_3$) δ 166.9, 163.6, 161.3, 145.3, 136.6, 128.7, 128.6, 128.5, 128.5, 128.3, 127.0, 62.6, 53.1, 13.7. **HRMS** (ESI) m/z: $[M + H]^+$ Calcd for $C_{16}H_{15}NO_4$ 302.1023; Found: 302.1021.

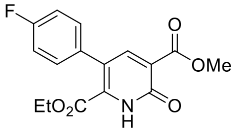

4q

2-Ethyl 5-methyl 3-(4-Fluorophenyl)-6-oxo-1,6-dihydropyridine-2,5-dicarboxylate, **4q**; White solid, (25.8 mg, 81% yield), 0.1 mmol scale reaction, Flash column chromatography conditions: hexane:ethyl acetate = 1:1. mp 145-147 °C. **^{1}H NMR** (500 MHz, $CDCl_3$) δ 10.73 (br s, 1H), 8.22 (s, 1H), 7.27-7.29 (comp, 2H), 7.14-7.12 (comp, 2H), 4.23 (q, *J* = 7.2 Hz, 2H), 4.00 (s, 3H), 1.14 (t, *J* = 7.2 Hz, 3H). **^{13}C NMR** (126 MHz, $CDCl_3$) δ. 166.9, 163.4, 162.7 (d, $J_{C\text{-}F}$ = 220 Hz), 161.3, 145.0, 132.6, 132.5, 130.5, 130.4, (d, $J_{C\text{-}F}$ = 4 Hz), 126.0, 115.5 (d, $J_{C\text{-}F}$ = 21 Hz), 62.6, 53.1, 13.7. **^{19}F NMR** (471 MHz, $CDCl_3$) δ -113.9. **HRMS** (ESI) m/z: $[M + H]^+$ Calcd for $C_{16}H_{14}FNO_5$ 320.0929; Found: 320.0928.

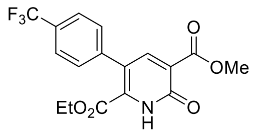

4r

2-Ethyl 5-methyl 6-oxo-3-(4-(Trifluoromethyl)phenyl)-1,6-dihydropyridine-2,5-dicarboxylate, 4r; White solid, (29.5 mg, 80% yield), 0.1 mmol scale reaction,. Flash column chromatography conditions: hexane:ethyl acetate = 1:1. mp 162-164 ℃. ^{1}**H NMR** (500 MHz, $CDCl_3$) δ 10.84 (br s, 1H), 8.23 (s, 1H), 7.71 (d, *J* = 8.0 Hz, 2H), 7.45 (d, *J* = 8.0 Hz, 2H), 4.24 (q, *J* = 7.2 Hz, 2H), 4.01 (s, 3H), 1.12 (t, *J* = 7.2 Hz, 3H). ^{13}**C NMR** (126 MHz, $CDCl_3$) δ 166.8, 163.2, 161.6, 144.7, 131.0, 130.4 (q, $J_{C\text{-}F}$ = 37 Hz), 129.1, 125.4 (q, $J_{C\text{-}F}$ = 7.2 Hz), 135.4, 123.5 (q, $J_{C\text{-}F}$ = 7.2 Hz), 123.0 (q, $J_{C\text{-}F}$ = 272 Hz), 120.7, 62.7, 53.2, 13.6. ^{19}**F NMR** (471 MHz, $CDCl_3$) δ -63.1. **HRMS** (ESI) m/z: $[M + H]^+$ Calcd for $C_{17}H_{14}F_3NO_5$ 370.0897; Found: 370.0895.

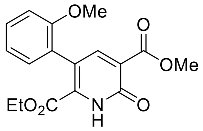

4s

2-Ethyl 5-methyl 3-(2-Methoxyphenyl)-6-oxo-1,6-dihydropyridine-2,5-dicarboxylate, 4s; White solid, (28.1 mg, 85% yield), 0.1 mmol scale reaction, following procedure 2. Flash column chromatography conditions: hexane:ethyl acetate = 1:1. mp 94-96 ℃. ^{1}**H NMR** (500 MHz, $CDCl_3$) δ 10.54 (br s, 1H), 8.17 (s, 1H), 7.37-7.25 (m, 1H), 7.18-7.16 (m, 1H), 7.01 (t, *J* = 7.4 Hz, 1H), 6.90 (d, *J* = 8.2 Hz, 1H), 4.17 (q, *J* = 7.1 Hz, 2H), 3.94 (s, 3H), 3.73 (s, 3H), 1.06 (t, *J* = 7.1 Hz, 3H). ^{13}**C NMR** (126 MHz, $CDCl_3$) δ 166.5, 162.8, 160.5, 156.4, 147.3, 132.3, 130.0, 129.9, 125.8, 122.2, 120.8, 120.3, 110.5, 62.4, 55.4, 53.0, 13.7. **HRMS** (ESI) m/z: $[M + H]^+$ Calcd for $C_{17}H_{17}NO_6$ 332.1129; Found: 332.1130.

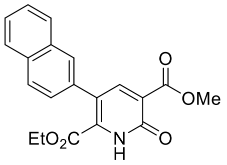

4t

2-Ethyl 5-methyl 3-(Naphthalen-2-yl)-6-oxo-1,6-dihydropyridine-2,5-dicarboxylate, 4t; White solid, (28.8 mg, 82% yield), 0.1 mmol scale reaction, Flash column chromatography conditions: hexane:ethyl acetate = 1:1. mp 156-158 ℃. ^{1}**H NMR** (500 MHz, $CDCl_3$) δ 10.71 (br s, 1H), 8.24 (s, 1H), 7.90 (d, *J* = 8.3 Hz, 2H), 7.53-7.43 (comp, 4H), 7.32 (d, *J* = 7.0 Hz, 1H), 3.93 (s, 3H), 3.90 (t, *J* = 7.1 Hz, 2H), 0.59 (t, *J* = 7.1 Hz, 3H). ^{13}**C NMR** (126 MHz, $CDCl_3$) δ 166.1, 162.2, 160.4, 147.2, 134.3, 133.4, 131.9, 128.7, 128.7, 128.7, 128.4, 126.8, 126.6, 126.1, 125.1, 124.8, 123.9, 62.4, 52.9, 13.0. **HRMS** (ESI) m/z: $[M + H]^+$ Calcd for $C_{20}H_{17}NO_5$ 352.1179; Found: 352.1177.

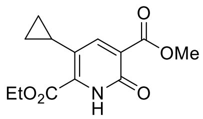

4u

2-Ethyl 5-methyl 3-cyclopropyl-6-oxo-1,6-dihydropyridine-2,5-dicarboxylate, 4u; White solid, (19.6 mg, 74% yield), 0.1 mmol scale reaction,. Flash column chromatography conditions: hexane:ethyl acetate = 1:1. mp 130-132 ℃. ^{1}**H NMR** (500 MHz, $CDCl_3$) δ 10.29 (br s, 1H), 7.86

(s, 1H), 4.45 (q, *J* = 7.1 Hz, 2H), 3.94 (s, 3H), 2.47-2.45 (m, 1H), 1.42 (t, *J* = 7.1 Hz, 3H), 1.01 (comp, 2H), 0.67 (comp, 2H). ^{13}**C NMR** (126 MHz, $CDCl_3$) δ 166.5, 162.7, 159.7, 142.8, 142.0, 142.0, 127.5, 62.8, 53.0, 14.3, 11.2, 8.1, 8.0. **HRMS** (ESI) m/z: $[M + H]^+$ Calcd for $C_{13}H_{15}NO_5$ 266.1023; Found: 266.1021.

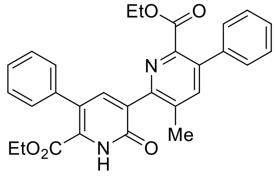

9a

Diethyl 3-methyl-2'-oxo-5,5'-diphenyl-1',2'-dihydro-[2,3'-bipyridine]-6,6'-dicarboxylate, 9a; White solid (26.8 mg, 90% yield) 0.05 mmol scale reaction. Flash column chromatography conditions: hexane:ethyl acetate = 1:2. Mp 142-144 °C ^{1}**H NMR** (500 MHz, $CDCl_3$) ^{1}H NMR δ 10.01 (br s, 1H), 7.78 (s, 1H), 7.66 (s, 1H), 7.45 – 7.35 (comp, 8H), 7.33 (dd, *J* = 6.3, 2.6 Hz, 2H), 4.20 (q, *J* = 7.1 Hz, 2H), 4.14 (q, *J* = 7.1 Hz, 2H), 2.48 (s, 3H), 1.05 (t, *J* = 7.1 Hz, 3H), 1.01 (t, *J* = 7.1 Hz, 3H). ^{13}**C NMR** (126 MHz, $CDCl_3$) δ ^{13}C NMR (126 MHz, $CDCl_3$) δ 167.0, 161.3, 159.3, 151.9, 146.7, 145.4, 140.2, 138.2, 137.0, 136.8, 136.5, 135.4, 130.3, 129.0, 128.4, 128.3, 128.2, 128.0, 129.9, 125.5, 62.7, 61.5, 19.0, 13.6, 13.4. **HRMS** (ESI) m/z: $[M + H]^+$ Calcd for $C_{29}H_{26}N_2O_5$ 483.1914; Found: 483.1918.

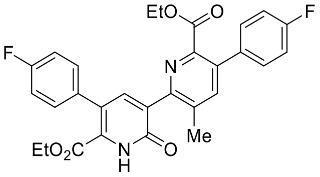

9b

Diethyl 5,5'-bis(4-Fluorophenyl)-3-methyl-2'-oxo-1',2'-dihydro-[2,3'-bipyridine]-6,6'-dicarboxylate, 9b; White solid (22.3 mg, 86% yield) 0.05 mmol scale reaction. Flash column chromatography conditions: hexane:ethyl acetate = 1:2. Mp 162-164 °C. ^{1}**H NMR** (500 MHz, $CDCl_3$) δ 10.06 (br s, 1H), 7.73 (s, 1H), 7.63 (s, 1H), 7.35-7.29 (comp, 4H), 7.15-7.09 (comp, 4H), 4.24 – 4.14 (comp, 4H), 2.48 (s, 3H), 1.10-1.04 (comp, 6H). ^{13}**C NMR** (126 MHz, $CDCl_3$) δ 166.8, 162.7 (d, J_{C-F} = 252 Hz), 162.6 (d, J_{C-F} = 250 Hz), 161.1, 159.3, 152.0, 146.6, 145.2, 140.3, 136.5, 135.9, 135.6, 134.2 (d, J_{C-F} = 4 Hz), 132.9 (d, J_{C-F} = 4 Hz), 130.8 (d, J_{C-F} = 8 Hz), 130.6, 130.0 (d, J_{C-F} = 8 Hz), 124.3, 115.4 (d, J_{C-F} = 22 Hz), 115.0 (d, J_{C-F} = 22 Hz), 62.8, 61.6, 19.0, 13.7, 13.5. ^{19}**F NMR** (471 MHz, $CDCl_3$) δ -113.8, -114.0. **HRMS** (ESI) m/z: $[M + H]^+$ Calcd for $C_{29}H_{24}F_2N_2O_5$ 519.1726; Found: 519.1728.

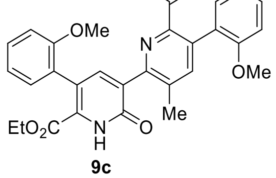

9c

Diethyl 5,5'-bis(2-Methoxyphenyl)-3-methyl-2'-oxo-1',2'-dihydro-[2,3'-bipyridine]-6,6'-dicarboxylate, 9c; White solid (23.9 mg, 88% yield) 0.05 mmol scale reaction. Flash column chromatography conditions: hexane:ethyl acetate = 1:2. Mp 149-151 °C. ^{1}**H NMR** (500 MHz, $CDCl_3$) δ 10.03 (br s, 1H), 7.76 (s, 1H), 7.60 (s, 1H), 7.39-7.34 (comp, 2H), 7.28 – 7.23 (comp, 2H), 7.08-6.99 (comp, 2H), 6.92 (d, *J* = 8.2 Hz, 2H), 4.19-4.10 (comp, 4H), 3.77 (s, 3H), 3.74 (s,

3H), 2.48 (s, 3H), 1.09-1.04 (comp, 6H). **^{13}C NMR** (126 MHz, $CDCl_3$) δ 166.4, 161.7, 159.5, 156.4, 156.1, 151.8, 146.9, 146.0, 141.2, 136.2, 135.7, 133.8, 131.7, 130.3, 130.2, 130.00, 129.5, 129.4, 127.9, 126.1, 120.8, 120.4, 110.3, 110.1, 62.3, 61.0, 55.4, 55.1, 19.1, 13.7, 13.5. **HRMS** (ESI) m/z: $[M + H]^+$ Calcd for $C_{31}H_{30}N_2O_7$ 543.2126; Found: 543.2132.

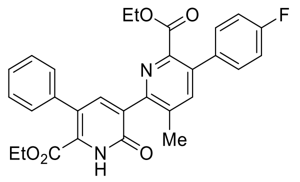

9d

Diethyl 5-(4-Fluorophenyl)-3-methyl-2'-oxo-5'-phenyl-1',2'-dihydro-[2,3'-bipyridine]-6,6'-dicarboxylate, 9d; White solid (43.5 mg, 87% yield) 0.1 mmol scale reaction. Flash column chromatography conditions: hexane:ethyl acetate = 1:2. Mp 154-156 °C. **^{1}H NMR** (500 MHz, $CDCl_3$) δ 9.95 (br s, 1H), 7.77 (s, 1H), 7.63 (s, 1H), 7.42-7.39 (comp, 3H), 7.35-7.31 (comp, 4H), 7.14 (t, *J* = 8.4 Hz, 2H), 4.18 (comp, 4H), 2.48 (s, 3H), 1.05 (comp, 6H). **^{13}C NMR** (126 MHz, $CDCl_3$) δ 166.8, 162.7 (d, J_{C-F} = 252 Hz), 161.4, 159.3, 152.2, 146.6, 145.4, 140.2, 137.0, 136.4, 135.8, 135.5, 134.2 (d, J_{C-F} = 4 Hz), 130.4, 130.0 (d, J_{C-F} = 9 Hz),, 129.0, 128.0, 128.0, 125.4, 115.4 (d, J_{C-F} = 25 Hz), 62.7, 61.6, 19.0, 13.7, 13.4. **^{19}F NMR** (471 MHz, $CDCl_3$) δ -114.0. **HRMS** (ESI) m/z: $[M + H]^+$ Calcd for $C_{29}H_{25}FN_2O_5$ 501.1820; Found: 501.1823.

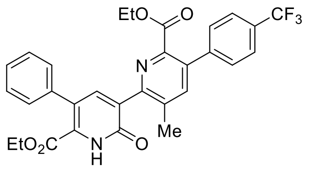

9e

Diethyl 3-Methyl-2'-oxo-5'-phenyl-5-(4-(Trifluoromethyl)phenyl)-1',2'-dihydro-[2,3'-bipyridine]-6,6'-dicarboxylate, 9e; White solid (49.5 mg, 90% yield) 0.1 mmol scale reaction. Flash column chromatography conditions: hexane:ethyl acetate = 1:2. Mp 168-170 °C. **^{1}H NMR** (500 MHz, $CDCl_3$) δ 9.99 (br s, 1H), 7.78 (s, 1H), 7.71 (d, *J* = 8.0 Hz, 2H), 7.64 (s, 1H), 7.49 (d, *J* = 8.0 Hz, 2H), 7.44 – 7.39 (comp, 3H), 7.34-7.31 (comp, 2H), 4.23 – 4.13 (comp, 4H), 2.50 (s, 3H), 1.07 – 0.99 (comp, 6H). **^{13}C NMR** (126 MHz, $CDCl_3$) δ 166.3, 161.3, 159.2, 152.8, 146.2, 145.4, 142.1, 140.2, 136.6, 136.2, 135.9, 135.8, 130.5, 130.2 (q, J_{C-F} = 32 Hz), 129.0, 128.7, 128.1, 128.0, 125.5, 125.3 (q, J_{C-F} = 4 Hz), 124.1 (q, J_{C-F} = 272 Hz), 62.7, 61.7, 19.1, 13.6, 13.4. **^{19}F NMR** (471 MHz, $CDCl_3$) δ -62.6. **HRMS** (ESI) m/z: $[M + H]^+$ Calcd for $C_{30}H_{25}F_3N_2O_5$ 551.1788; Found: 551.1793.

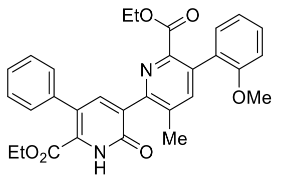

9f

Diethyl 5-(2-Methoxyphenyl)-3-methyl-2'-oxo-5'-phenyl-1',2'-dihydro-[2,3'-bipyridine]-6,6'-dicarboxylate, 9f; White solid (45.6 mg, 89% yield) 0.1 mmol scale reaction. Flash column chromatography conditions: hexane:ethyl acetate = 1:2. Mp 150-152 °C. **^{1}H NMR** (500 MHz, $CDCl_3$) δ 10.01 (br s, 1H), 7.81 (s, 1H), 7.61 (s, 1H), 7.42-7.36 (comp, 4H), 7.35 – 7.31 (comp, 2H), 7.28-7.26 (comp, 1H), 7.07 (t, *J* = 8.2 Hz, 1H), 6.93 (d, *J* = 8.2 Hz, 1H), 4.19 (q, *J* = 7.5 Hz,

2H), 4.13 (q, *J* = 7.1 Hz, 2H), 3.74 (s, 3H), 2.47 (s, 3H), 1.06-1.01 (comp, 6H). **^{13}C NMR** (126 MHz, $CDCl_3$) δ 166.3, 161.5, 159.4, 156.1, 151.5, 146.9, 145.3, 141.3, 137.1, 136.6, 135.7, 134.0, 130.4, 130.0, 129.5, 129.0, 128.0, 127.9, 127.8, 125.6, 120.8, 110.1, 62.6, 61.1, 55.1, 19.1, 13.7, 13.5. **HRMS** (ESI) m/z: $[M + H]^+$ Calcd for $C_{30}H_{28}N_2O_6$ 513.2020; Found: 513.2022.

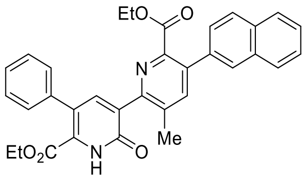

9g

Diethyl 3-Methyl-5-(naphthalen-2-yl)-2'-oxo-5'-phenyl-1',2'-dihydro-[2,3'-bipyridine]-6,6'-dicarboxylate, 9g; White solid (45.8 mg, 86% yield) 0.1 mmol scale reaction. Flash column chromatography conditions: hexane:ethyl acetate = 1:2. Mp 172-174 °C. **^{1}H NMR** (500 MHz, $CDCl_3$) δ 9.87 (br s, 1H), 7.89 (d, *J* = 8.3 Hz, 2H), 7.84 (s, 1H), 7.69 (s, 1H), 7.57 (d, *J* = 8.3 Hz, 1H), 7.54 – 7.46 (comp, 2H), 7.45 – 7.37 (comp, 4H), 7.35-7.32 (comp, 3H), 4.19 (q, *J* = 7.1 Hz, 2H), 3.80 (q, *J* = 7.1 Hz, 2H), 2.49 (s, 3H), 1.03 (t, *J* = 7.1 Hz, 3H), 0.53 (t, *J* = 7.1Hz, 3H). **^{13}C NMR** (126 MHz, $CDCl_3$) δ 166.0, 161.4, 159.3, 152.6, 147.1, 145.6, 141.7, 137.0, 136.6, 136.4, 136.0, 135.8, 133.3, 131.8, 130.4, 129.0, 128.2, 128.2, 128.1, 128.0, 127.9, 126.4, 126.3, 126.0, 125.5, 125.1, 62.7, 61.1, 19.1, 13.4, 13.1. **HRMS** (ESI) m/z: $[M + H]^+$ Calcd for $C_{33}H_{28}N_2O_5$ 533.2071; Found: 533.2076.

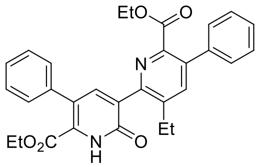

9h

Diethyl 3-Ethyl-2'-oxo-5,5'-diphenyl-1',2'-dihydro-[2,3'-bipyridine]-6,6'-dicarboxylate, 9h; White solid (38.7 mg, 78% yield) 0.1 mmol scale reaction. Flash column chromatography conditions: hexane:ethyl acetate = 1:2. Mp 145-147 °C. **^{1}H NMR** (500 MHz, $CDCl_3$) δ 9.86 (br s, 1H), 7.73 (s, 1H), 7.71 (s, 1H), 7.44-7.36 (comp, 8H), 7.34-7.30 (comp, 2H), 4.20 (q, *J* = 7.1, 2H), 4.15 (q, *J* = 7.1, 2H), 2.83 (q, *J* = 7.1 Hz, 2H), 1.26 (t, *J* = 7.1 Hz, 3H), 1.05 (t, *J* = 7.1 Hz, 3H), 1.01 (t, *J* = 7.1 Hz, 3H). **^{13}C NMR** (126 MHz, $CDCl_3$) δ 167.0, 161.3, 159.6, 151.6, 146.7, 145.3, 140.9, 138.4, 138.3, 137.1, 137.0, 136.7, 136.7, 130.1, 129.0, 128.4, 128.3, 128.0, 127.9, 125.5, 62.7, 61.5, 25.1, 14.1, 13.6, 13.5. **HRMS** (ESI) m/z: $[M + H]^+$ Calcd for $C_{30}H_{28}N_2O_5$ 497.2071; Found: 497.2070.

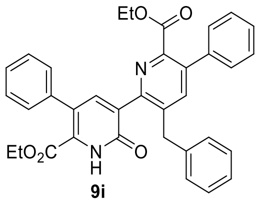

9i

Diethyl 3-Benzyl-2'-oxo-5,5'-diphenyl-1',2'-dihydro-[2,3'-bipyridine]-6,6'-dicarboxylate, 9i; White solid (40.2 mg, 72% yield) 0.1 mmol scale reaction. Flash column chromatography conditions: hexane:ethyl acetate = 1:2. Mp 160-162 °C. **^{1}H NMR** (500 MHz, $CDCl_3$) δ 9.85 (br s, 1H), 7.62 (s, 1H), 7.49 (s, 1H), 7.46 – 7.31 (comp, 8H), 7.26 – 7.12 (comp, 5H), 7.07 (d, *J* = 7.3 Hz, 2H), 4.23 (s, 2H), 4.19-4.15 (comp, 4H), 1.04-0.98 (comp, 6H). **^{13}C NMR** (126 MHz, $CDCl_3$) δ 167.0, 161.3, 159.6, 152.1, 147.2, 145.5, 139.9, 139.2, 138.7, 138.0, 136.9, 136.7, 136.5, 130.1,

129.1, 128.9, 128.5, 128.4, 128.3, 128.0, 127.9, 127.8, 126.4, 125.4, 62.7, 61.6, 38.6, 13.6, 13.4. **HRMS** (ESI) m/z: $[M + H]^+$ Calcd for $C_{35}H_{30}N_2O_5$ 559.2227; Found: 559.2231.

6. 1 mmol scale reaction: Procedure for the synthesis of pyridineone compound 4a from 1,2,3-triazine 1-oxide and ketoester

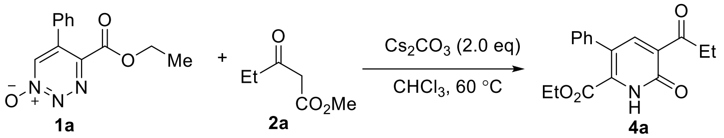

A chloroform solution (10.0 mL) of 1,2,3-triazine 1-oxide derivative **1a** (1.0 mmol, 1 equiv.) was added dropwise over 30 minutes to a 10.0 mL solution of $CHCl_3$ containing β-keto ester **2a** (1.5 mmol, 1.5 equiv.) and a Cs_2CO_3 (20 mmol, 652 mg) at 60 °C. The reaction was continued for 1 h at same temperature. After completion of the reaction (monitored by TLC), the reaction mixture was cooled to room temperature and diluted with 50.0 mL of dichloromethane. The organic layer was washed with saturated ammonium chloride solution (50.0 mL) followed by water and brine solution, then dried over anhydrous $MgSO_4$, filtered, and the solvent was evaporated under reduced pressure. Pure pyridone compound **4a** was obtained from DCM/hexane solvent (5:1) mixture by crystallization in 92% yield (275 mg).

7. Crystallographic data

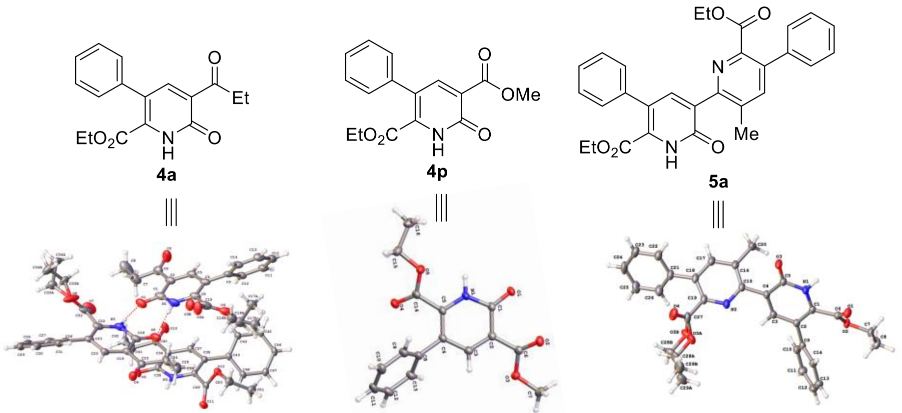

CCDC = 2127334 CCDC = 2284794 CCDC = 2292359

Single crystals of $C_{17}H_{17}NO_4$ (**4a**) were prepared by slow evaporation of a dichloromethane:hexane (1:10) solution. A suitable colorless plate-like crystal, with dimensions of 0.140 mm × 0.088 mm × 0.074 mm, was mounted in paratone oil onto a nylon loop. Single crystals of $C_{16}H_{15}NO_5$ (**4p**) were prepared by slow evaporation of a ethyl acetate:hexane (10:1) solution. A suitable colorless plate-like crystal of **4p**, with dimensions of 0.152 mm × 0.112 mm × 0.096 mm, was mounted in paratone oil onto a nylon loop. Single crystals of $C_{29}H_{26}N_2O_5$ (**9a**) were prepared by slow evaporation of a dichloromethane:hexane (1:10) solution. A suitable colorless plate-like crystal, with dimensions of 0.152 mm × 0.112 mm × 0.096 mm, was mounted in paratone oil onto a nylon loop. All data were collected at 298(1) K and 298(1) K for compounds

4a, 4p and **9a** respectively, using a XtaLAB Synergy/ Dualflex, HyPix fitted with CuKα radiation (λ = 1.54184 Å). Data collection and unit cell refinement were performed using *CrysAlisPro* software.[1] The total number of data were measured in the 6.2° < 2θ < 153.2°, 6.6° < 2θ < 153.0° and 9° < 2θ < 152.2° for compounds **4a**, **4p** and **9a** respectively, using ω scans. Data processing and absorption correction, giving minimum and maximum transmission factors (0.653, 1.000 for compound (**4a**)/ 0.621, 1.00 for compound (**4p**)/ 0.487, 1.00 for compound (**9a**)) were accomplished with *CrysAlisPro*[3] and *SCALE3 ABSPACK*[4], respectively. The structure, using Olex2[5], was solved with the ShelXT[6] structure solution program using direct methods and refined (on F^2) with the ShelXL[7] refinement package using full-matrix, least-squares techniques. All non-hydrogen atoms were refined with anisotropic displacement parameters. All hydrogen atom positions were determined by geometry and refined by a riding model.

Compound number	**4a**	**4p**	**9a**
Identification code	Hpd462(1)	Hpd614(2)	Hpd750
Empirical formula	$C_{17}H_{17}NO_4$	$C_{16}H_{15}NO_5$	$C_{29}H_{26}N_2O_5$
Formula weight	299.31	301.29	482.52
Crystal system	Triclinic	Monoclinic	Monoclinic
Space group	*P-1*	$P2_1/n$	$P2_1/c$
a (Å)	12.5967(2)	15.6843(2)	13.83436(15)
b (Å)	12.8747(2)	6.33089(7)	11.10289(10)
c (Å)	15.6198(3)	15.8937(2)	20.5729(2)
α (°)	72.566(2)	90	90
β (°)	69.811(1)	114.758(2)	109.0934(12)
γ (°)	75.602(1)	90	90
Volume ($Å^3$)	2238.30(7)	1433.11(3)	2986.18(6)
Z	6	4	4
ρ (calc.)	1.332	1.396	1.073
λ	1.54184	1.54184	1.54184
Temp. (K)	100.0(1)	298(1)	100(1)

F(000)	948	632	1016
μ (mm^{-1})	0.785	0.877	0.601
T_{min}, T_{max}	0.653, 1.000	0.621, 1.000	0.487, 1.000
$2\theta_{range}$ (°)	6.2 to 153.2	6.6, 153.0	6.76 to 152.8
Reflections collected	42637	30584	29239
Independent reflections	9064 [R(int) = 0.0360]	2921 [R(int) = 0.0329]	5984 [R(int) = 0.0315]
Completeness	99.8%	99.9%	99.9%
Data / restraints / parameters	9064 / 44 / 624	2921 / 0 / 204	5384 / 0 / 359
Observed data [I > 2σ(I)]	8008	2657	5338
$wR(F^2$ all data)	0.1031	0.0943	0.1291
$R(F$ obsd data)	0.0430	0.0351	0.0443
Goodness-of-fit on F^2	0.89	1.06	1.05
largest diff. peak and hole (e $Å^{-3}$)	0.38 / -0.46	0.20 / -0.21	0.20 / -0.32

$wR_2 = \{ \Sigma [w(F_o^2 - F_c^2)^2] / \Sigma [w(F_o^2)^2] \}^{1/2}$

$R_1 = \Sigma ||F_o| - |F_c|| / \Sigma |F_o|$

8. Computational data

Software

Quantum chemical calculations were performed using the Lonestar6 supercomputer at the Texas Advanced Computing Center (TACC) hosted by the University of Texas in Austin, Texas, and Bridges-2 supercomputer hosted by the Pittsburgh Supercomputing Center (PSC) and supported by Advanced Cyberinfrastructure Coordination Ecosystem: Services & Support (ACCESS) program. DFT geometry optimization, vibrational frequency, and IRC calculations were conducted using Gaussian 16 (rA.03).[8] The CREST utility of the xTB software suite[9] was used to locate initial starting geometries for optimization via DFT. Final images of minima and transition state structure geometries were rendered using CYLview (v1.0.600)[10] and VMD (v1.9.4a53).[11] Routine visualization and monitoring of calculations was performed with Chemcraft (v1.8-622b).[12]

Details of Computational Methods

Gaussian 16 DFT calculations

Geometries of ground state minima and transition state structures were optimized without constraints using MN15 density functional approximation and the def2-TZVP basis set in dichloromethane solvent using the SMD solvation model. Calculations were set to "tight" convergence criteria with an ultrafine grid. Frequency calculations at the same level of theory were used to confirm the nature of the isolated stationary points. Geometries with zero imaginary frequencies were deemed minima whereas those with exactly one imaginary frequency along the chemical path of interest were deemed transition state structures. Intrinsic reaction coordinate (IRC) calculations were performed to further corroborate that the located transition state structures connected reactants to products. The quasi-harmonic approximation at 1M concentration was applied via GoodVibes[13] to all structures to correct for potential errors associated with low magnitude vibrational frequencies using a cut-off frequency of 50 cm^{-1}. Single point corrections of the above geometries were calculated using PW6B95-D3(BJ) in dichloromethane solvent under the SMD solvation model. The def2-TZVPPD basis set was used by appending diffuse functions obtained from the EMSL BSE[14] to the G16-available def2-TZVPP basis set. This level of theory provided the final electronic component to the reported free energies.

Boltzmann Ensemble Averaging

To improve the accuracy of the DFT computational analysis of the reaction pathway, ensemble averaging was applied across the obtained structurally distinct conformers of each structure, as previously described.[15]

Diastereomeric Energy Diagrams

Cyclization transition state **TSB** resulted in cis and trans diastereomeric reaction pathways for structures **TSB**, **7**, and **7b**, with the pathways recombining at the final products, **8-E** and **8-K**. The metal-free pathway shows kinetic preference for trans configuration in the addition of the imine nitrogen to both the keto and the ester groups. The sodium- and cesium-mediated pathways show kinetic preference for cis configuration in the addition of the imine nitrogen to both the keto and ester groups. Energy difference between the most favorable and second most favorable transition states for each pathway (metal-free, $\Delta\Delta G^{\neq}$ = 5.5 kcal/mol; sodium-mediated, $\Delta\Delta G^{\neq}$ = 3.2 kcal/mol; cesium mediated, $\Delta\Delta G^{\neq}$ = 3.3 kcal/mol) suggest substantial kinetic preference for the lowest energy transition state over all other diastereomeric pathways.

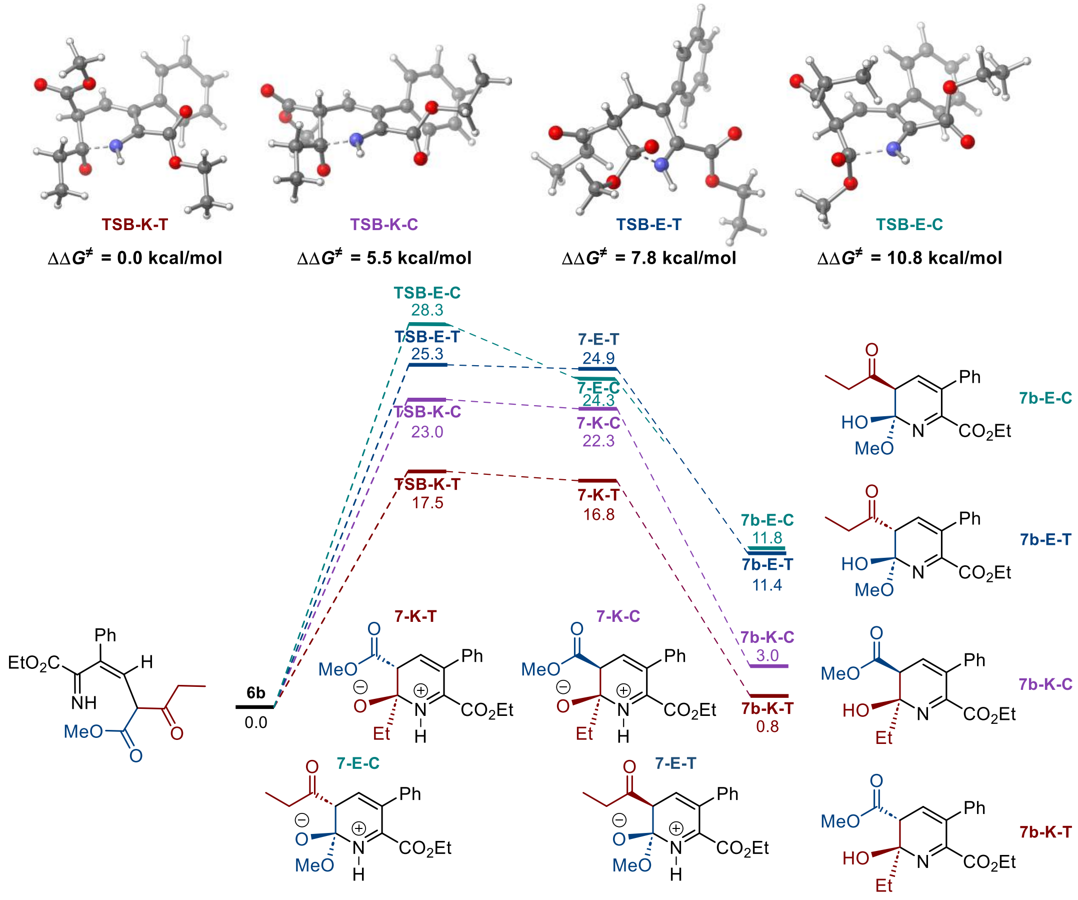

Scheme S1. Computed Gibbs free energy profile of metal ion-free cyclization pathways, ΔG, kcal/mol.

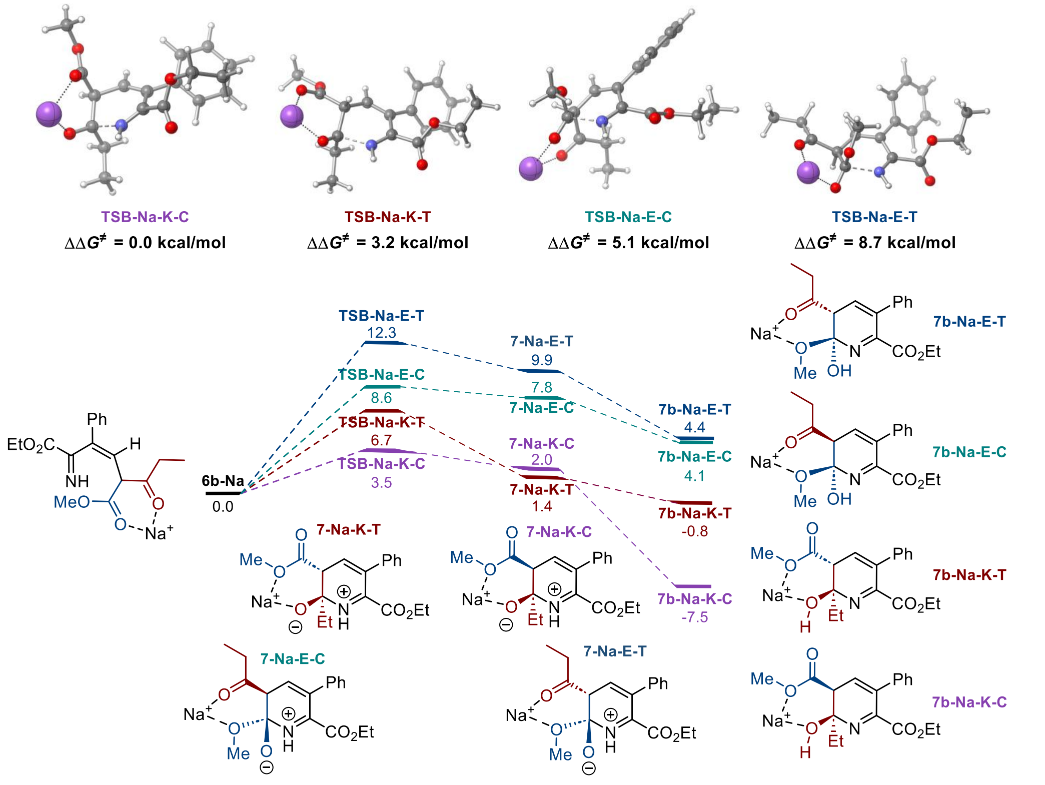

Scheme S2. Computed Gibbs free energy profile of the sodium-mediated cyclization pathways, ΔG, kcal/mol.

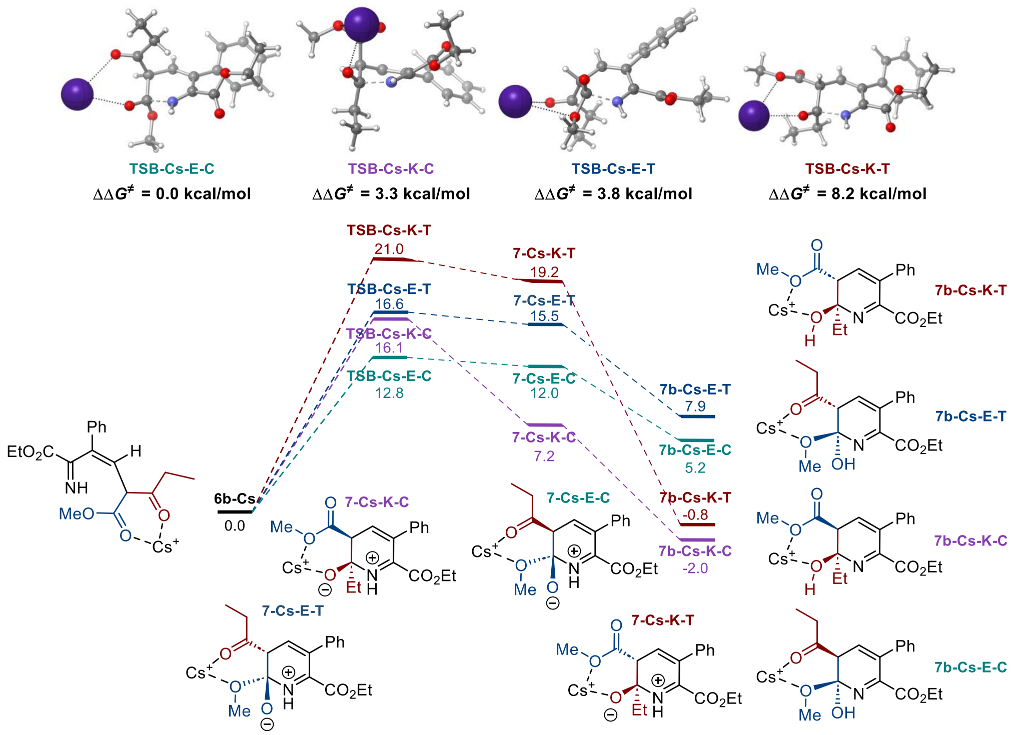

Scheme S3. Computed Gibbs free energy profile of the cesium-mediated cyclization pathways, ΔG, kcal/mol.

Distortion/Interaction-Activation Strain Analysis of TSB

A distortion/interaction-activation strain analysis[16] was performed as described previously[15] on each metal-free and metal-coordinated variant of **TSB** optimized at the MN15 / def2-TZVP / SMD (DCM) level of theory. Ensemble averaging was applied for the calculated distortion and interaction values for each fragment to improve accuracy. Radical fragment definitions were created for **TSB**, with the red fragment representing the imine portion of the molecule (Fragment **F1**) and the green fragment representing the remaining structure, including both sites of cyclization (Fragment **F2**), as previously described for the analysis of intramolecular processes.[17] For metal coordinated species, the metal cation was removed from each geometry, and all single point calculations were performed on the net neutral system.

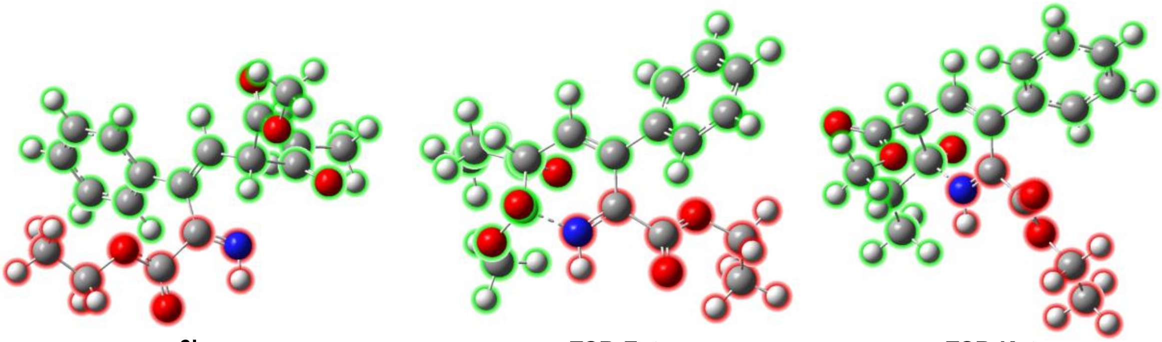

6b **TSB-Est** **TSB-Ket**

Figure S1. Division of **6b** and **TSB** into fragments for distortion/interaction-activation strain analysis.

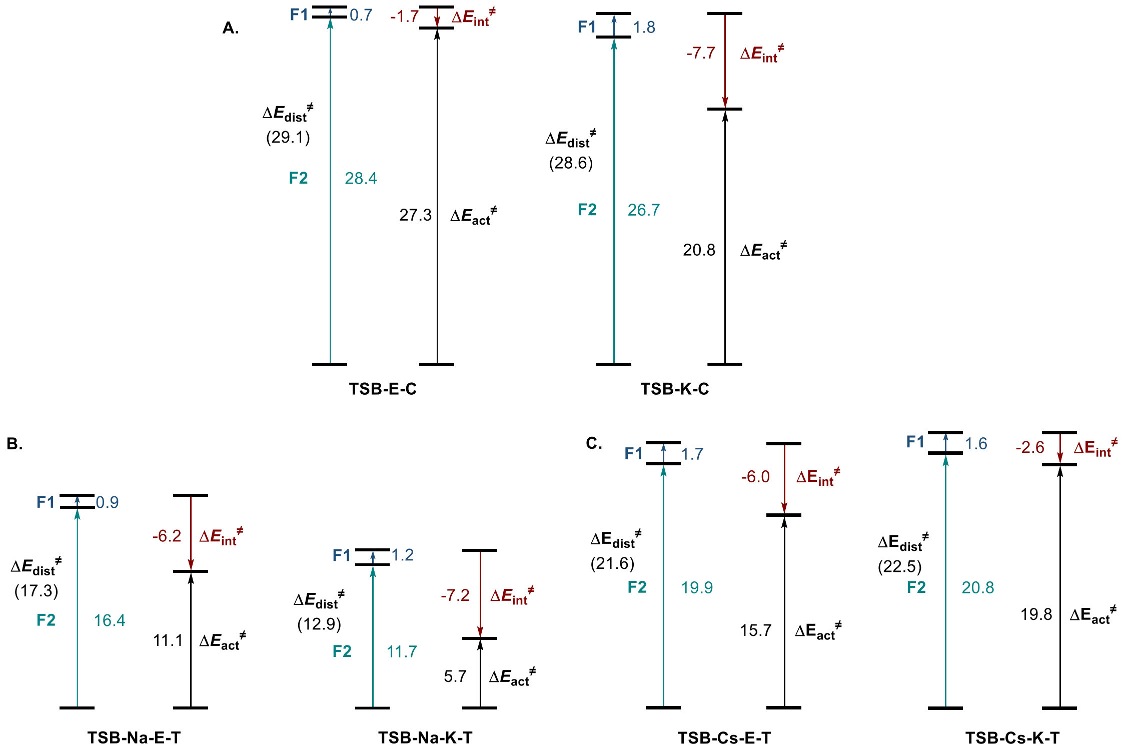

Figure S2. Distortion/Interaction analysis of the disfavored diastereomeric cyclization transition states. A. The cyclization process in the absence of metal ions (cis pathway). **B**. The sodium-mediated trans pathway. **C**. The cesium-mediated trans pathway. ΔE, kcal/mol.

Conformational Analysis and Tables of Thermodynamic Values by Structure – PW6B95(D3BJ) / def2-TZVPPD / SMD (DCM)

5

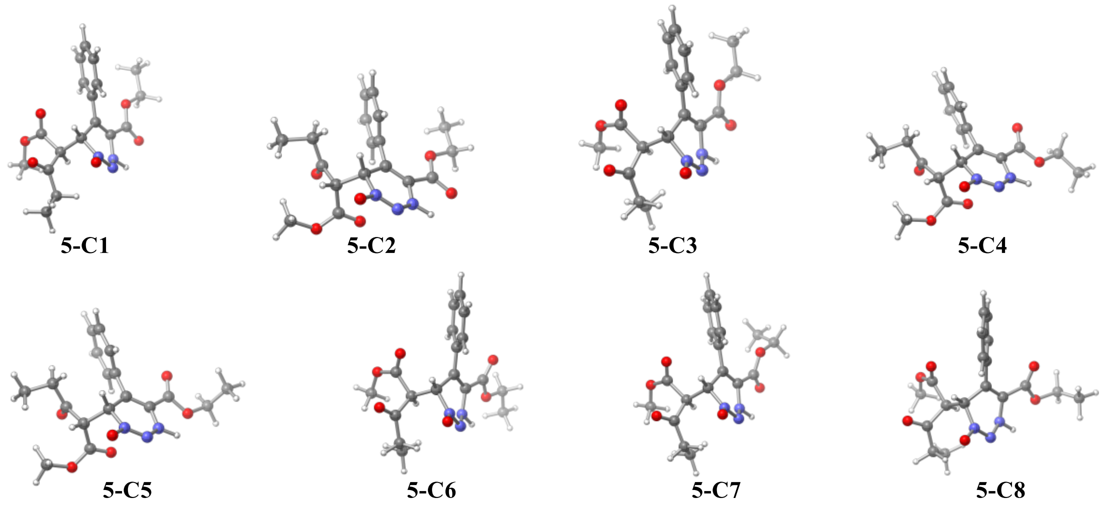

Figure S3. Conformers of addition intermediate **5**.

Table S2. Energies of conformers of addition intermediate **5**.[a]

Structure	Δ*E*	Δ*H*	Δ*G*	Δ*G* (GV50)	ΔΔ*G*
**5-C1**	-1316.242973	-1315.833191	-1315.918831	-1315.915303	0.00
**5-C2**	-1316.242367	-1315.832843	-1315.917799	-1315.914949	0.22
**5-C3**	-1316.240997	-1315.831176	-1315.916271	-1315.912885	1.52
**5-C4**	-1316.240446	-1315.83067	-1315.915392	-1315.912628	1.68
**5-C5**	-1316.23976	-1315.829803	-1315.914298	-1315.911359	2.47
**5-C6**	-1316.239083	-1315.829015	-1315.914742	-1315.911135	2.62
**5-C7**	-1316.240261	-1315.830115	-1315.914123	-1315.9111	2.64
**5-C8**	-1316.238423	-1315.828428	-1315.914413	-1315.910414	3.07

[a]Energies were obtained as single points at the PW6B95(D3BJ) / def2-TZVPPD / SMD (DCM) level of theory based on structures optimized at the MN15 / def2-TZVP / SMD (DCM) level of theory. ΔE, ΔH, ΔG, and ΔG (GV50), hartree; ΔΔG, kcal/mol.

TSA

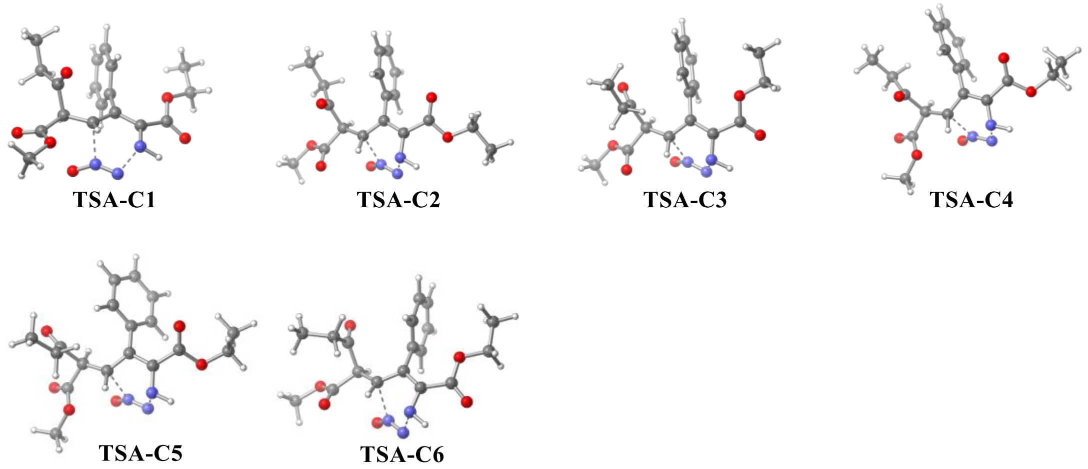

Figure S4. Conformers of N_2O extrusion transition state **TSA**.

Table S3. Energies of conformers of N_2O extrusion transition state **TSA**.[a]

Structure	Δ*E*	Δ*H*	Δ*G*	Δ*G* (GV50)	ΔΔ*G*
**TSA-C1**	-1316.199217	-1315.794156	-1315.880713	-1315.878142	0.00
**TSA-C2**	-1316.198488	-1315.793513	-1315.880669	-1315.877761	0.24
**TSA-C3**	-1316.19751	-1315.792557	-1315.88	-1315.876821	0.83
**TSA-C4**	-1316.196803	-1315.791721	-1315.879333	-1315.875928	1.39
**TSA-C5**	-1316.194997	-1315.789916	-1315.876806	-1315.873672	2.80
**TSA-C6**	-1316.194386	-1315.789174	-1315.876952	-1315.873606	2.85

[a]Energies were obtained as single points at the PW6B95(D3BJ) / def2-TZVPPD / SMD (DCM) level of theory based on structures optimized at the MN15 / def2-TZVP / SMD (DCM) level of theory. ΔE, ΔH, ΔG, and ΔG (GV50), hartree; ΔΔG, kcal/mol.

6

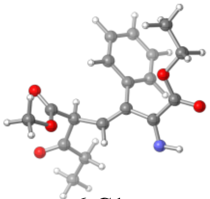

6-C1

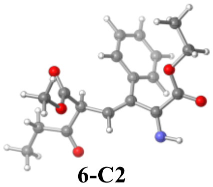

6-C2

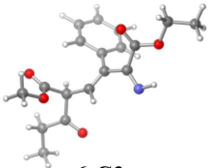

6-C3

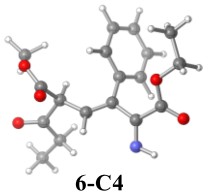

6-C4

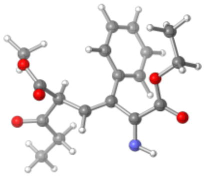

6-C5

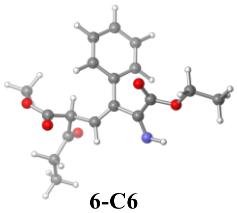

6-C6

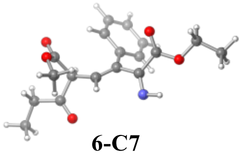

6-C7

Figure S5. Conformers of extrusion intermediate **6**.

Table S4. Energies of conformers of extrusion intermediate **6**.[a]

Structure	Δ*E*	Δ*H*	Δ*G*	Δ*G* (GV50)	ΔΔ*G*
**6-C1**	-1131.318146	-1130.927426	-1131.012624	-1131.008106	0.00
**6-C2**	-1131.316676	-1130.92589	-1131.011364	-1131.006838	0.80
**6-C3**	-1131.315077	-1130.924267	-1131.009997	-1131.005449	1.67
**6-C4**	-1131.310984	-1130.920123	-1131.004303	-1130.999982	5.10
**6-C5**	-1131.309155	-1130.918342	-1131.002077	-1130.998225	6.20
**6-C6**	-1131.306818	-1130.915871	-1130.999743	-1130.995827	7.71
**6-C7**	-1131.30445	-1130.913581	-1130.997096	-1130.993318	9.28

[a]Energies were obtained as single points at the PW6B95(D3BJ) / def2-TZVPPD / SMD (DCM) level of theory based on structures optimized at the MN15 / def2-TZVP / SMD (DCM) level of theory. ΔE, ΔH, ΔG, and ΔG (GV50), hartree; ΔΔG, kcal/mol.

6b

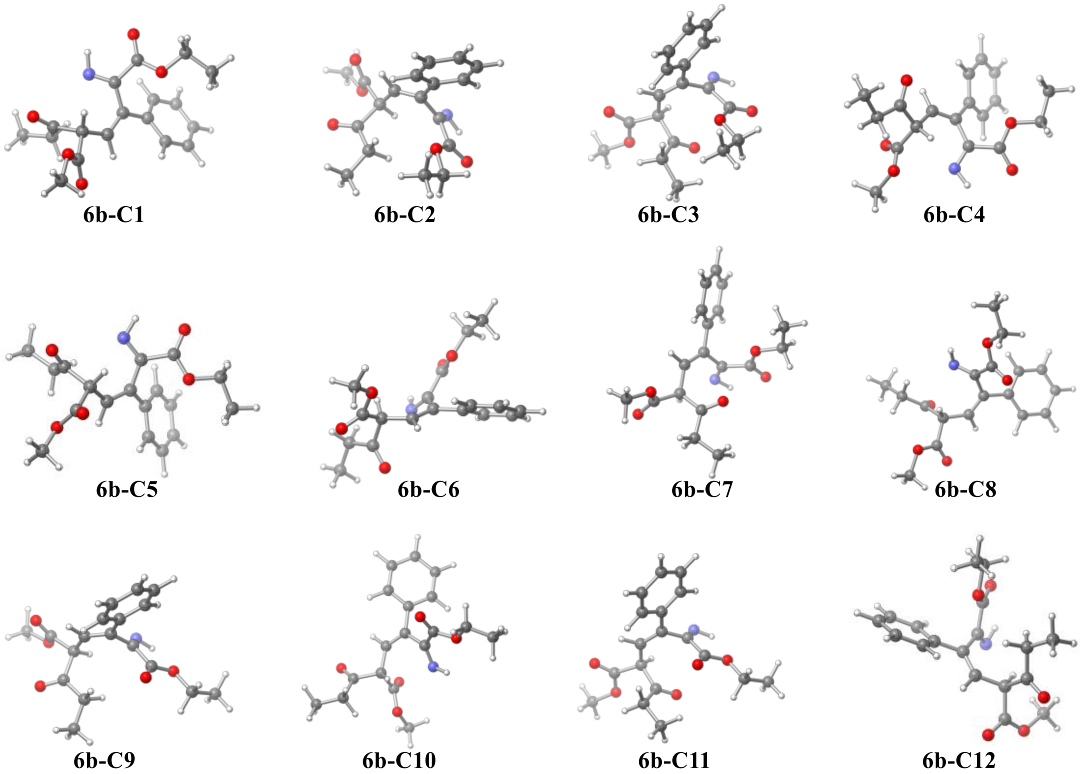

Figure S6. Conformers of isomerized intermediate **6b**.

Table S5. Energies of conformers of isomerized intermediate **6b**. [a]

Structure	Δ*E*	Δ*H*	Δ*G*	Δ*G* (GV50)	ΔΔ*G*
**6b-C1**	-1131.318958	-1130.928391	-1131.012545	-1131.0086	0.00
**6b-C2**	-1131.319154	-1130.928385	-1131.011964	-1131.008373	0.14
**6b-C3**	-1131.318089	-1130.927608	-1131.011613	-1131.007463	0.71
**6b-C4**	-1131.318157	-1130.927512	-1131.01067	-1131.007352	0.78
**6b-C5**	-1131.317976	-1130.927188	-1131.010913	-1131.0072	0.88
**6b-C6**	-1131.317632	-1130.926819	-1131.010577	-1131.006855	1.10
**6b-C7**	-1131.31655	-1130.926088	-1131.010651	-1131.006476	1.33
**6b-C8**	-1131.316878	-1130.926017	-1131.010281	-1131.00635	1.41
**6b-C9**	-1131.316688	-1130.925877	-1131.00989	-1131.006274	1.46
**6b-C10**	-1131.316339	-1130.925536	-1131.010182	-1131.006098	1.57
**6b-C11**	-1131.315391	-1130.924678	-1131.008922	-1131.004985	2.27
**6b-C12**	-1131.311704	-1130.9208	-1131.00364	-1131.000063	5.36

[a]Energies were obtained as single points at the PW6B95(D3BJ) / def2-TZVPPD / SMD (DCM) level of theory based on structures optimized at the MN15 / def2-TZVP / SMD (DCM) level of theory. ΔE, ΔH, ΔG, and ΔG (GV50), hartree; ΔΔG, kcal/mol.

6b-Cs

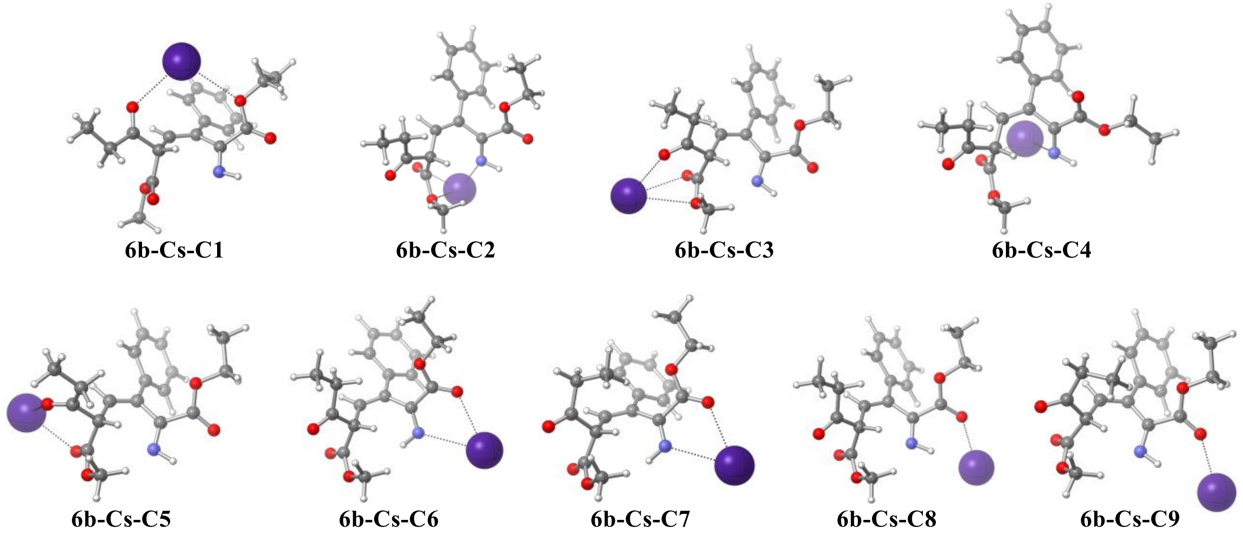

Figure S7. Conformers of cesium-coordinated isomerized intermediate **6b-Cs**.

Table S6. Energies of conformers of cesium-coordinated isomerized intermediate **6b-Cs**. [a]

Structure	Δ*E*	Δ*H*	Δ*G*	Δ*G* (GV50)	ΔΔ*G*
**6b-Cs-C1**	-1151.417318	-1151.023362	-1151.112575	-1151.108624	0.00
**6b-Cs-C2**	-1151.413913	-1151.019832	-1151.109166	-1151.10531	2.08
**6b-Cs-C3**	-1151.413084	-1151.019122	-1151.10913	-1151.104901	2.34
**6b-Cs-C4**	-1151.412111	-1151.018228	-1151.108677	-1151.104181	2.79
**6b-Cs-C5**	-1151.41269	-1151.018355	-1151.108971	-1151.104054	2.87
**6b-Cs-C6**	-1151.409636	-1151.015048	-1151.105093	-1151.100573	5.05
**6b-Cs-C7**	-1151.408912	-1151.01428	-1151.10385	-1151.099553	5.69
**6b-Cs-C8**	-1151.406531	-1151.012377	-1151.102211	-1151.097999	6.67
**6b-Cs-C9**	-1151.405714	-1151.011444	-1151.102591	-1151.097247	7.14

[a]Energies were obtained as single points at the PW6B95(D3BJ) / def2-TZVPPD / SMD (DCM) level of theory based on structures optimized at the MN15 / def2-TZVP / SMD (DCM) level of theory. ΔE, ΔH, ΔG, and ΔG (GV50), hartree; ΔΔG, kcal/mol.

6b-Na

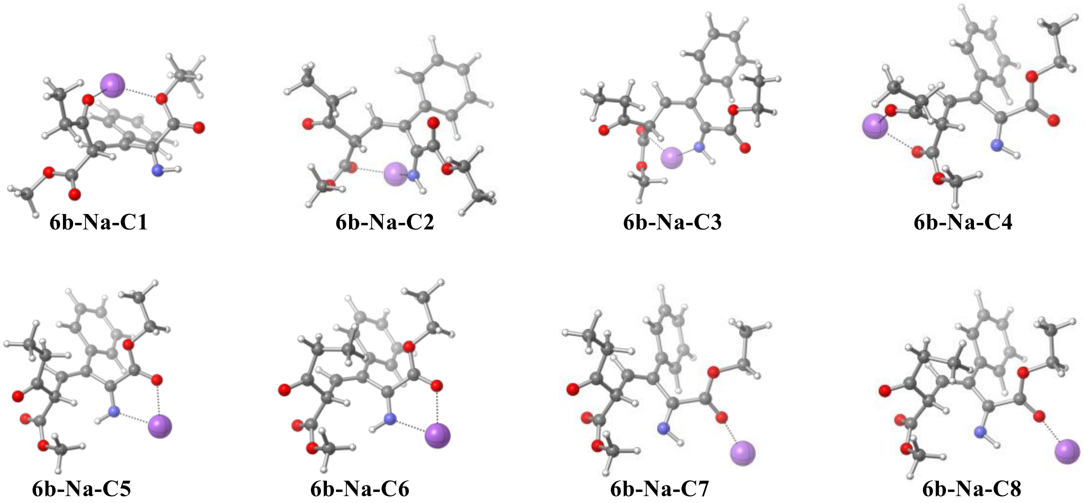

Figure S8. Conformers of sodium-coordinated isomerized intermediate **6b-Na**.

Table S7. Energies of conformers of sodium-coordinated isomerized intermediate **6b-Na**. [a]

Structure	Δ*E*	Δ*H*	Δ*G*	Δ*G* (GV50)	ΔΔ*G*
**6b-Na-C1**	-1293.708364	-1293.31402	-1293.399133	-1293.396588	0.00
**6b-Na-C2**	-1293.704391	-1293.309956	-1293.397078	-1293.393529	1.92
**6b-Na-C3**	-1293.703664	-1293.309525	-1293.396133	-1293.392842	2.35
**6b-Na-C4**	-1293.70428	-1293.309604	-1293.395373	-1293.392165	2.78
**6b-Na-C5**	-1293.702677	-1293.307711	-1293.394156	-1293.390357	3.91
**6b-Na-C6**	-1293.702336	-1293.30707	-1293.392202	-1293.389118	4.69
**6b-Na-C7**	-1293.686625	-1293.292733	-1293.381195	-1293.377066	12.25
**6b-Na-C8**	-1293.686112	-1293.291793	-1293.379874	-1293.375435	13.27

[a]Energies were obtained as single points at the PW6B95(D3BJ) / def2-TZVPPD / SMD (DCM) level of theory based on structures optimized at the MN15 / def2-TZVP / SMD (DCM) level of theory. ΔE, ΔH, ΔG, and ΔG (GV50), hartree; ΔΔG, kcal/mol.

TSB-E-T

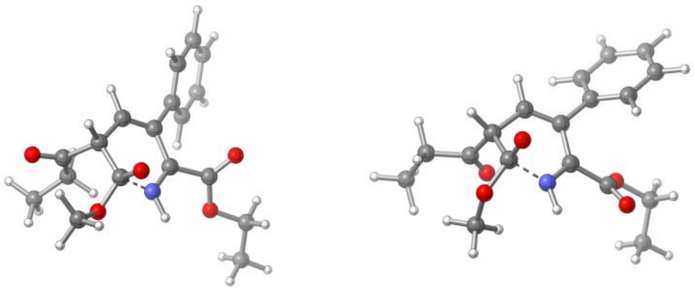

TSB-E-T-C1 **TSB-E-T-C2**

Figure S9. Conformers of ester cyclization trans transition state **TSB-E-T**.

Table S8. Energies of conformers of ester cyclization trans transition state **TSB-E-T**.[a]

Structure	ΔE	ΔH	ΔG	ΔG (GV50)	ΔΔG
**TSB-E-T-C1**	-1131.279933	-1130.891491	-1130.972814	-1130.969288	0.00
**TSB-E-T-C2**	-1131.276121	-1130.887405	-1130.965681	-1130.962761	4.10

[a]Energies were obtained as single points at the PW6B95(D3BJ) / def2-TZVPPD / SMD (DCM) level of theory based on structures optimized at the MN15 / def2-TZVP / SMD (DCM) level of theory. ΔE, ΔH, ΔG, and ΔG (GV50), hartree; ΔΔG, kcal/mol.

TSB-E-C

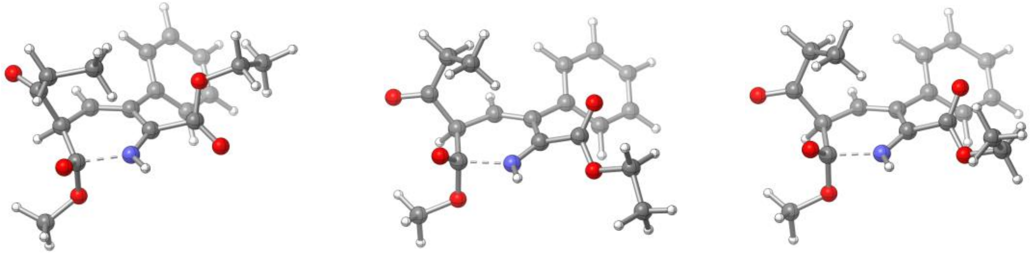

TSB-E-C-C1 **TSB-E-C-C2** **TSB-E-C-C3**

Figure S10. Conformers of ester cyclization cis transition state **TSB-E-C**.

Table S9. Energies of conformers of ester cyclization cis transition state **TSB-E-C**.[a]

Structure	ΔE	ΔH	ΔG	ΔG (GV50)	ΔΔG
**TSB-E-C-C1**	-1131.273262	-1130.884655	-1130.96559	-1130.961959	0.00
**TSB-E-C-C2**	-1131.273182	-1130.884249	-1130.964461	-1130.961458	0.31
**TSB-E-C-C3**	-1131.272725	-1130.883765	-1130.963504	-1130.960582	0.86

[a]Energies were obtained as single points at the PW6B95(D3BJ) / def2-TZVPPD / SMD (DCM) level of theory based on structures optimized at the MN15 / def2-TZVP / SMD (DCM) level of theory. ΔE, ΔH, ΔG, and ΔG (GV50), hartree; ΔΔG, kcal/mol.

TSB-K-T

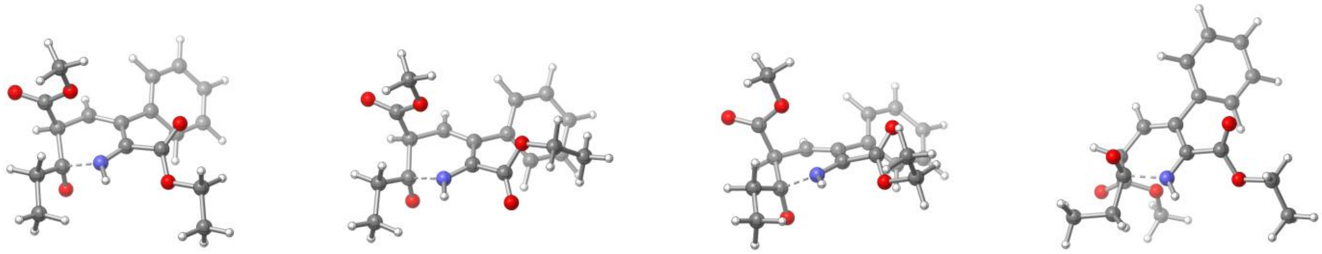

TSB-K-T-C1 **TSB-K-T-C2** **TSB-K-T-C3** **TSB-K-T-C4**

Figure S11. Conformers of ketone cyclization trans transition state **TSB-K-T**.

Table S10. Energies of conformers of ketone cyclization trans transition state **TSB-K-T**.[a]

Structure	ΔE	ΔH	ΔG	ΔG (GV50)	ΔΔG
**TSB-K-T-C1**	-1131.292553	-1130.903029	-1130.981893	-1130.979408	0.00
**TSB-K-T-C2**	-1131.292844	-1130.903409	-1130.981448	-1130.979066	0.21
**TSB-K-T-C3**	-1131.292053	-1130.90243	-1130.98105	-1130.978576	0.52
**TSB-K-T-C4**	-1131.290316	-1130.900729	-1130.979539	-1130.976878	1.59

[a]Energies were obtained as single points at the PW6B95(D3BJ) / def2-TZVPPD / SMD (DCM) level of theory based on structures optimized at the MN15 / def2-TZVP / SMD (DCM) level of theory. ΔE, ΔH, ΔG, and ΔG (GV50), hartree; ΔΔG, kcal/mol.

TSB-K-C

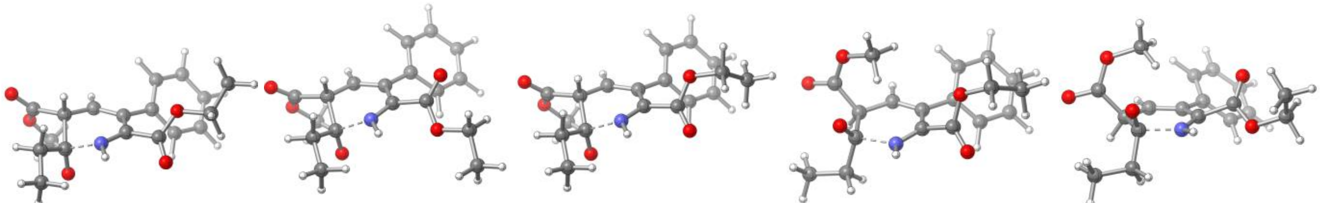

TSB-K-C-C1 **TSB-K-C-C2** **TSB-K-C-C3** **TSB-K-C-C4** **TSB-K-C-C5**

Figure S12. Conformers of ketone cyclization cis transition state **TSB-K-C**.

Table S11. Energies of conformers of ketone cyclization cis transition state **TSB-K-C**.[a]

Structure	ΔE	ΔH	ΔG	ΔG (GV50)	ΔΔG
**TSB-K-C-C1**	-1131.286239	-1130.897009	-1130.975863	-1130.973218	0.00
**TSB-K-C-C2**	-1131.285099	-1130.895775	-1130.974477	-1130.971894	0.83
**TSB-K-C-C3**	-1131.2855	-1130.896156	-1130.974216	-1130.971655	0.98
**TSB-K-C-C4**	-1131.280099	-1130.890806	-1130.968652	-1130.966269	4.36
**TSB-K-C-C5**	-1131.279246	-1130.889622	-1130.967568	-1130.9651	5.09

[a]Energies were obtained as single points at the PW6B95(D3BJ) / def2-TZVPPD / SMD (DCM) level of theory based on structures optimized at the MN15 / def2-TZVP / SMD (DCM) level of theory. ΔE, ΔH, ΔG, and ΔG (GV50), hartree; ΔΔG, kcal/mol.

TSB-Cs-E-T

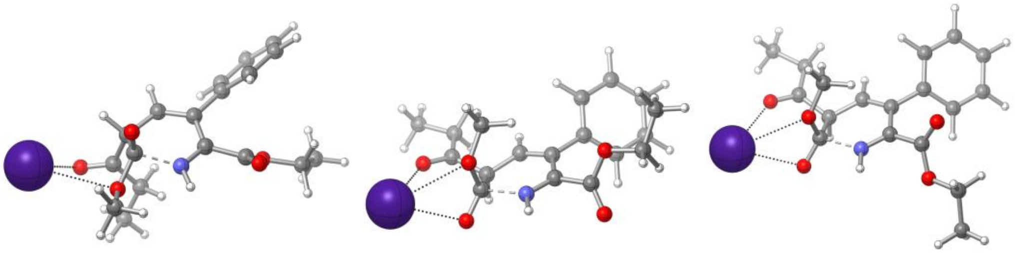

TSB-Cs-E-T-C1 **TSB-Cs-E-T-C2** **TSB-Cs-E-T-C3**

Figure S13. Conformers of cesium-coordinated ester cyclization trans transition state **TSB-Cs-E-T**.

Table S12. Energies of conformers of cesium-coordinated ester cyclization trans transition state **TSB-Cs-E-T**. [a]

Structure	ΔE	ΔH	ΔG	ΔG (GV50)	ΔΔG
**TSB-Cs-E-T-C1**	-1151.387739	-1150.995819	-1151.081755	-1151.078507	0.00

**TSB-Cs-E-T-C2**	-1151.385208	-1150.993566	-1151.078861	-1151.075213	2.07
**TSB-Cs-E-T-C3**	-1151.384459	-1150.992772	-1151.077911	-1151.074508	2.51

[a]Energies were obtained as single points at the PW6B95(D3BJ) / def2-TZVPPD / SMD (DCM) level of theory based on structures optimized at the MN15 / def2-TZVP / SMD (DCM) level of theory. ΔE, ΔH, ΔG, and ΔG (GV50), hartree; ΔΔG, kcal/mol.

TSB-Cs-E-C

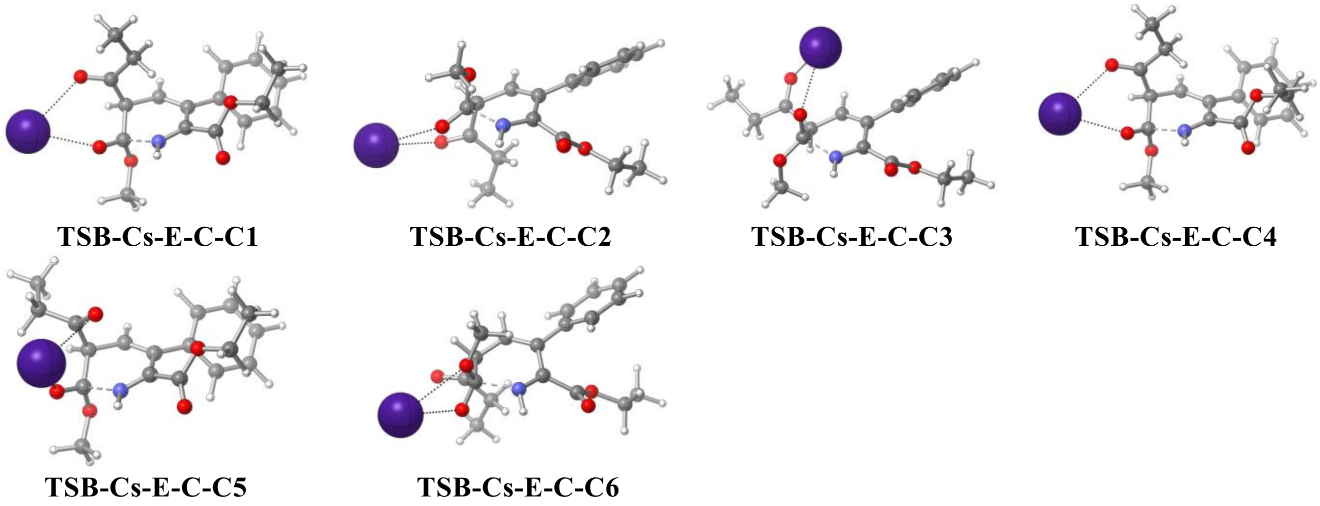

Figure S14. Conformers of cesium-coordinated ester cyclization cis transition state **TSB-Cs-E-C**.

Table S13. Energies of conformers of cesium-coordinated ester cyclization cis transition state **TSB-Cs-E-C**. [a]

Structure	ΔE	ΔH	ΔG	ΔG (GV50)	ΔΔG
**TSB-Cs-E-C-C1**	-1151.391698	-1151.000171	-1151.088019	-1151.083789	0.00
**TSB-Cs-E-C-C2**	-1151.391724	-1151.000243	-1151.086938	-1151.083121	0.42
**TSB-Cs-E-C-C3**	-1151.392381	-1151.000711	-1151.086222	-1151.082758	0.65
**TSB-Cs-E-C-C4**	-1151.39051	-1150.99878	-1151.086447	-1151.082228	0.98
**TSB-Cs-E-C-C5**	-1151.389591	-1150.997665	-1151.084742	-1151.081116	1.68
**TSB-Cs-E-C-C6**	-1151.387921	-1150.99624	-1151.084619	-1151.079629	2.61

[a]Energies were obtained as single points at the PW6B95(D3BJ) / def2-TZVPPD / SMD (DCM) level of theory based on structures optimized at the MN15 / def2-TZVP / SMD (DCM) level of theory. ΔE, ΔH, ΔG, and ΔG (GV50), hartree; ΔΔG, kcal/mol.

TSB-Cs-K-T

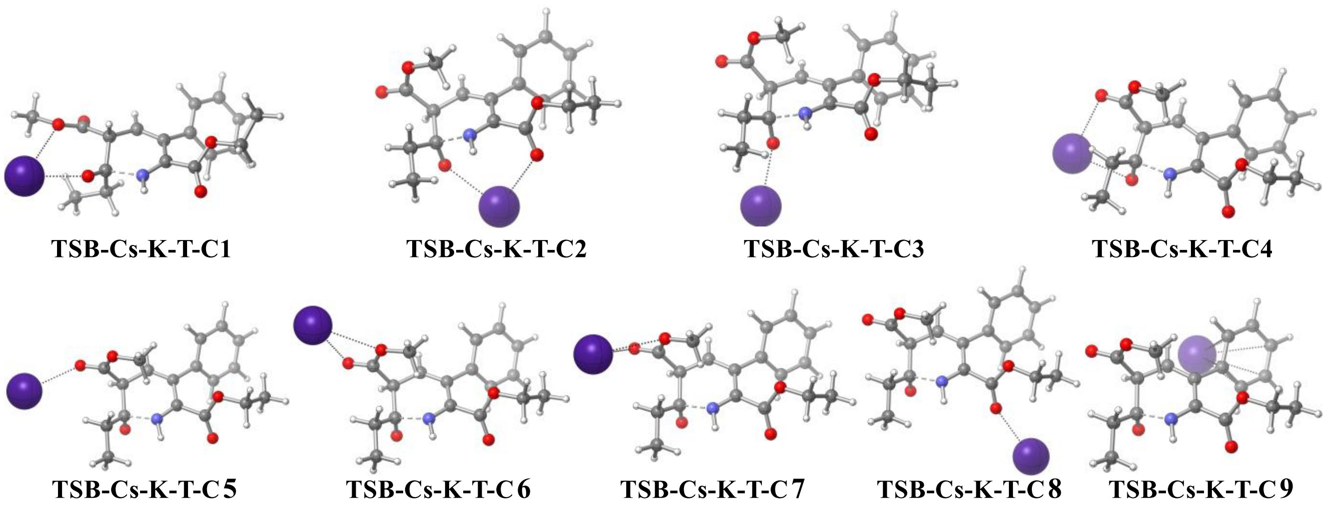

Figure S15. Conformers of cesium-coordinated ketone cyclization trans transition state **TSB-Cs-K-T**.

Table S14. Energies of conformers of cesium-coordinated ketone cyclization trans transition state **TSB-Cs-K-T**. [a]

Structure	ΔE	ΔH	ΔG	ΔG (GV50)	ΔΔG
**TSB-Cs-K-T-C1**	-1151.398956	-1151.00674	-1151.09289	-1151.089481	0.00
**TSB-Cs-K-T-C2**	-1151.382226	-1150.98941	-1151.074884	-1151.071333	11.39
**TSB-Cs-K-T-C3**	-1151.381473	-1150.988697	-1151.075356	-1151.071226	11.46
**TSB-Cs-K-T-C4**	-1151.379695	-1150.986665	-1151.072798	-1151.069043	12.83
**TSB-Cs-K-T-C5**	-1151.376443	-1150.983668	-1151.070114	-1151.066033	14.71
**TSB-Cs-K-T-C6**	-1151.375989	-1150.983253	-1151.069218	-1151.065563	15.01
**TSB-Cs-K-T-C7**	-1151.376068	-1150.983208	-1151.067824	-1151.064618	15.60
**TSB-Cs-K-T-C8**	-1151.373221	-1150.980386	-1151.0651	-1151.061769	17.39
**TSB-Cs-K-T-C9**	-1151.373216	-1150.980227	-1151.063851	-1151.06134	17.66

[a]Energies were obtained as single points at the PW6B95(D3BJ) / def2-TZVPPD / SMD (DCM) level of theory based on structures optimized at the MN15 / def2-TZVP / SMD (DCM) level of theory. ΔE, ΔH, ΔG, and ΔG (GV50), hartree; ΔΔG, kcal/mol.

TSB-Cs-K-C

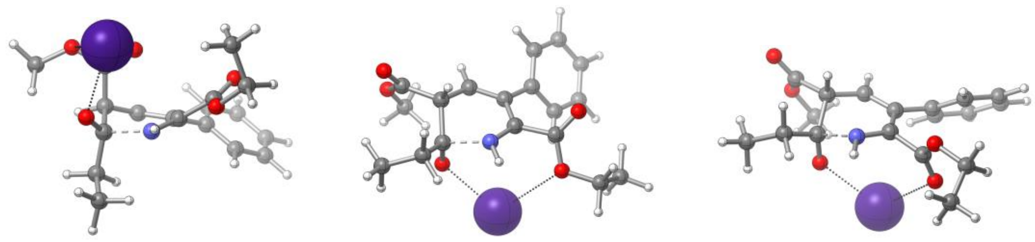

TSB-Cs-K-C-C1 **TSB-Cs-K-C-C2** **TSB-Cs-K-C-C3**

Figure S16. Conformers of cesium-coordinated ketone cyclization cis transition state **TSB-Cs-K-C**.

Table S15. Energies of conformers of cesium-coordinated ketone cyclization cis transition state **TSB-Cs-K-C**.[a]

Structure	ΔE	ΔH	ΔG	ΔG (GV50)	ΔΔG
**TSB-Cs-K-C-C1**	-1151.3932	-1151.000009	-1151.083425	-1151.080735	0.00
**TSB-Cs-K-C-C2**	-1151.385587	-1150.99305	-1151.078693	-1151.075312	3.40
**TSB-Cs-K-C-C3**	-1151.385812	-1150.992722	-1151.076855	-1151.074354	4.00

[a]Energies were obtained as single points at the PW6B95(D3BJ) / def2-TZVPPD / SMD (DCM) level of theory based on structures optimized at the MN15 / def2-TZVP / SMD (DCM) level of theory. ΔE, ΔH, ΔG, and ΔG (GV50), hartree; ΔΔG, kcal/mol.

TSB-Na-E-T

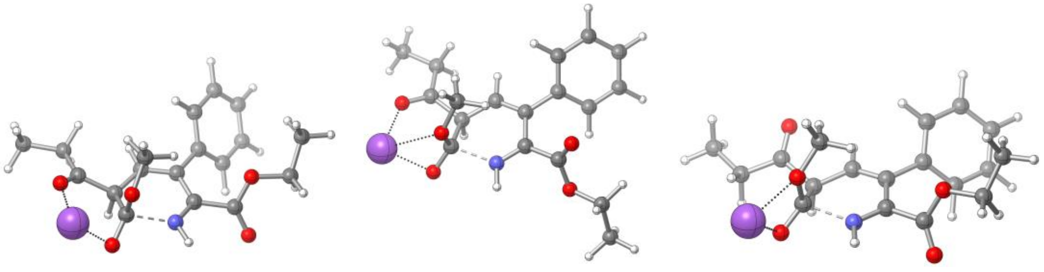

TSB-Na-E-T-C1 **TSB-Na-E-T-C2** **TSB-Na-E-T-C3**

Figure S17. Conformers of sodium-coordinated ester cyclization trans transition state **TSB-Na-E-T**.

Table S16. Energies of conformers of sodium-coordinated ester cyclization trans transition state **TSB-Na-E-T**.[a]

Structure	ΔE	ΔH	ΔG	ΔG (GV50)	ΔΔG

**TSB-Na-E-T-C1**	-1293.68462	-1293.292346	-1293.374027	-1293.371183	0.000
**TSB-Na-E-T-C2**	-1293.684077	-1293.291849	-1293.373488	-1293.370828	0.222
**TSB-Na-E-T-C3**	-1293.676163	-1293.284265	-1293.367953	-1293.364818	3.994

[a]Energies were obtained as single points at the PW6B95(D3BJ) / def2-TZVPPD / SMD (DCM) level of theory based on structures optimized at the MN15 / def2-TZVP / SMD (DCM) level of theory. ΔE, ΔH, ΔG, and ΔG (GV50), hartree; ΔΔG, kcal/mol.

TSB-Na-E-C

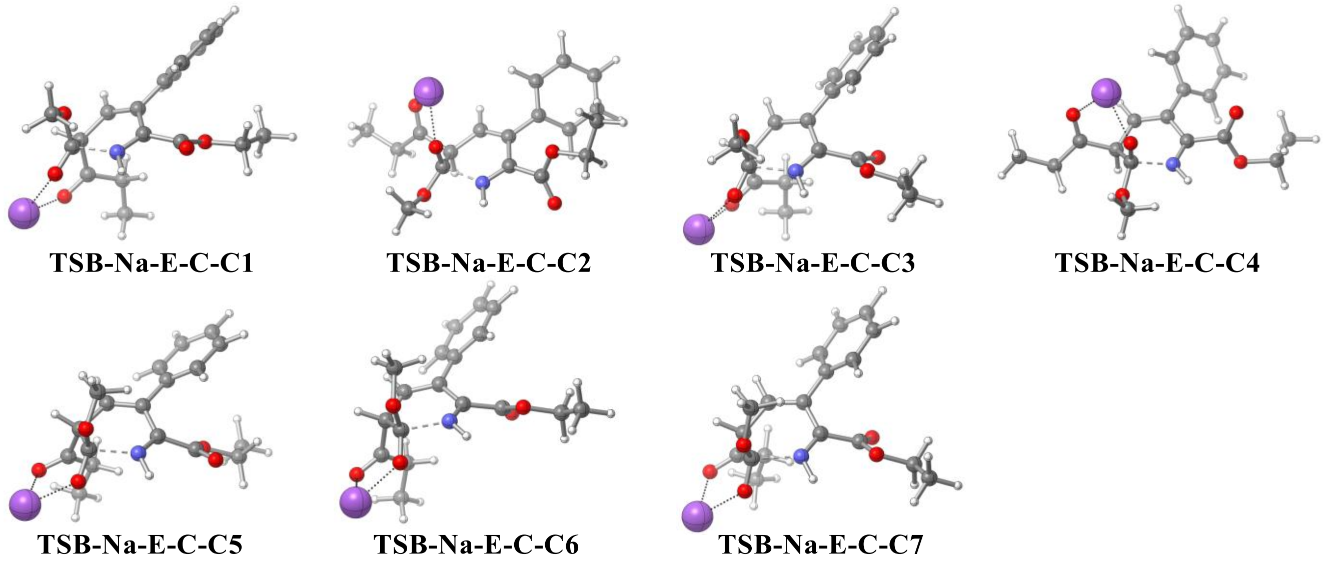

Figure S18. Conformers of sodium-coordinated ester cyclization cis transition state **TSB-Na-E-C**.

Table S17. Energies of conformers of sodium-coordinated ester cyclization cis transition state **TSB-Na-E-C**.[a]

Structure	ΔE	ΔH	ΔG	ΔG (GV50)	ΔΔG
**TSB-Na-E-C-C1**	-1293.690002	-1293.29828	-1293.382565	-1293.378835	0.00
**TSB-Na-E-C-C2**	-1293.688877	-1293.296999	-1293.38188	-1293.378059	0.49
**TSB-Na-E-C-C3**	-1293.688891	-1293.296945	-1293.381539	-1293.378026	0.51
**TSB-Na-E-C-C4**	-1293.688349	-1293.296011	-1293.379802	-1293.376652	1.37
**TSB-Na-E-C-C5**	-1293.683539	-1293.29155	-1293.37443	-1293.371119	4.84

**TSB-Na-E-C-C6**	-1293.682551	-1293.29057	-1293.374349	-1293.37067	5.12
**TSB-Na-E-C-C7**	-1293.682682	-1293.290246	-1293.372822	-1293.36958	5.81

[a]Energies were obtained as single points at the PW6B95(D3BJ) / def2-TZVPPD / SMD (DCM) level of theory based on structures optimized at the MN15 / def2-TZVP / SMD (DCM) level of theory. ΔE, ΔH, ΔG, and ΔG (GV50), hartree; ΔΔG, kcal/mol.

TSB-Na-K-T

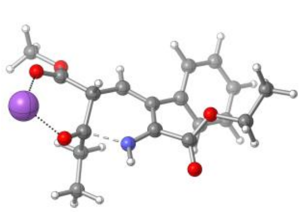

TSB-Na-K-T-C1

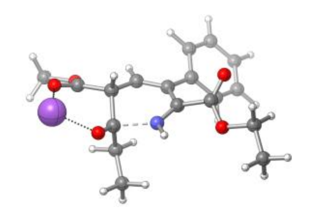

TSB-Na-K-T-C2

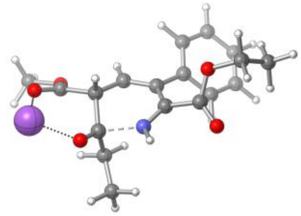

TSB-Na-K-T-C3

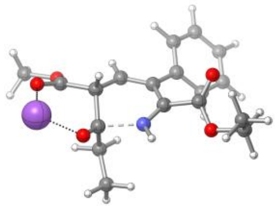

TSB-Na-K-T-C4

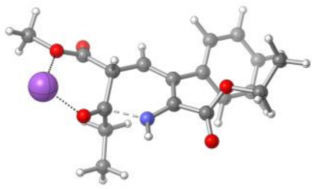

TSB-Na-K-T-C5

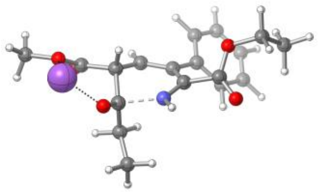

TSB-Na-K-T-C6

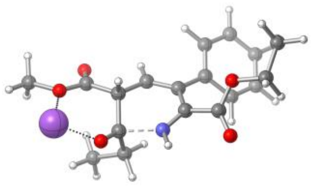

TSB-Na-K-T-C7

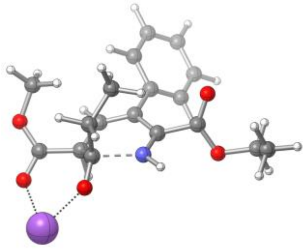

TSB-Na-K-T-C8

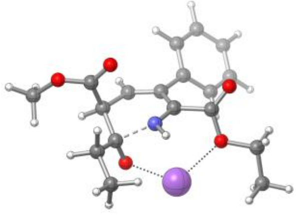

TSB-Na-K-T-C9

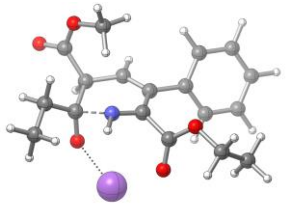

TSB-Na-K-T-C10

Figure S19. Conformers of sodium-coordinated ketone cyclization trans transition state **TSB-Na-K-T**.

Table S18. Energies of conformers of sodium-coordinated ketone cyclization trans transition state **TSB-Na-K-T**.[a]

Structure	ΔE	ΔH	ΔG	ΔG (GV50)	ΔΔG
**TSB-Na-K-T-C1**	-1293.700428	-1293.307574	-1293.390604	-1293.38811	0.00
**TSB-Na-K-T-C2**	-1293.699101	-1293.306369	-1293.39101	-1293.387474	0.40
**TSB-Na-K-T-C3**	-1293.699767	-1293.306802	-1293.389036	-1293.386596	0.95
**TSB-Na-K-T-C4**	-1293.698636	-1293.305451	-1293.388818	-1293.38578	1.46

**TSB-Na-K-T-C5**	-1293.69505	-1293.302329	-1293.385406	-1293.382821	3.32
**TSB-Na-K-T-C6**	-1293.694404	-1293.301477	-1293.383684	-1293.38136	4.24
**TSB-Na-K-T-C7**	-1293.693882	-1293.301042	-1293.383172	-1293.380893	4.53
**TSB-Na-K-T-C8**	-1293.675437	-1293.282229	-1293.363737	-1293.361179	16.90
**TSB-Na-K-T-C9**	-1293.675612	-1293.282179	-1293.362555	-1293.361088	16.96
**TSB-Na-K-T-C10**	-1293.674443	-1293.281039	-1293.363346	-1293.360422	17.37

[a]Energies were obtained as single points at the PW6B95(D3BJ) / def2-TZVPPD / SMD (DCM) level of theory based on structures optimized at the MN15 / def2-TZVP / SMD (DCM) level of theory. ΔE, ΔH, ΔG, and ΔG (GV50), hartree; ΔΔG, kcal/mol.

TSB-Na-K-C

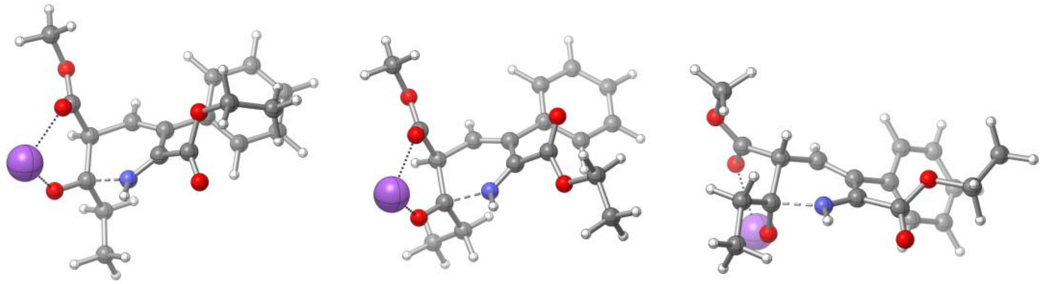

TSB-Na-K-C-C1 **TSB-Na-K-C-C2** **TSB-Na-K-C-C3**

Figure S20. Conformers of sodium-coordinated ketone cyclization cis transition state **TSB-Na-K-C**

Table S19. Energies of conformers of sodium-coordinated ketone cyclization cis transition state **TSB-Na-K-C**.[a]

Structure	ΔE	ΔH	ΔG	ΔG (GV50)	ΔΔG
**TSB-Na-K-C-C1**	-1293.696895	-1293.303979	-1293.388198	-1293.384486	0.00
**TSB-Na-K-C-C2**	-1293.695116	-1293.302311	-1293.387276	-1293.383284	0.75
**TSB-Na-K-C-C3**	-1293.69421	-1293.301267	-1293.38352	-1293.380796	2.32

[a]Energies were obtained as single points at the PW6B95(D3BJ) / def2-TZVPPD / SMD (DCM) level of theory based on structures optimized at the MN15 / def2-TZVP / SMD (DCM) level of theory. ΔE, ΔH, ΔG, and ΔG (GV50), hartree; ΔΔG, kcal/mol.

7-E-T

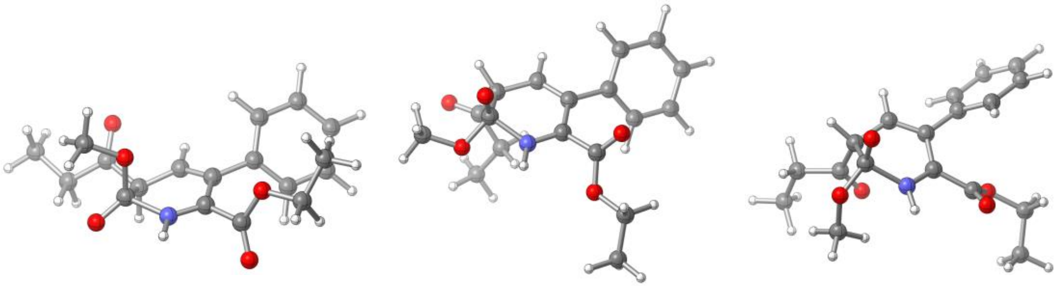

7-E-T-C1 **7-E-T-C2** **7-E-T-C3**

Figure S21. Conformers of the ester cyclization trans zwitterion **7-E-T**

Table S20. Energies of conformers of ester cyclization trans zwitterion **7-E-T**[a]

Structure	ΔE	ΔH	ΔG	ΔG (GV50)	ΔΔG
**7-E-T-C1**	-1131.27941	-1130.890415	-1130.971763	-1130.968923	0.00
**7-E-T-C2**	-1131.280776	-1130.890868	-1130.971201	-1130.968187	0.46
**7-E-T-C3**	-1131.276098	-1130.886372	-1130.966041	-1130.963103	3.65

[a]Energies were obtained as single points at the PW6B95(D3BJ) / def2-TZVPPD / SMD (DCM) level of theory based on structures optimized at the MN15 / def2-TZVP / SMD (DCM) level of theory. ΔE, ΔH, ΔG, and ΔG (GV50), hartree; ΔΔG, kcal/mol.

7-E-C

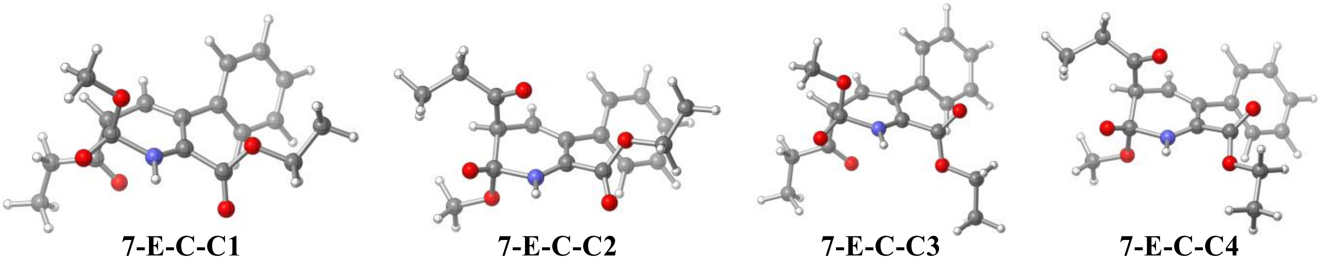

7-E-C-C1 **7-E-C-C2** **7-E-C-C3** **7-E-C-C4**

Figure S22. Conformers of the ester cyclization cis zwitterion **7-E-C**

Table S21. Energies of conformers of ester cyclization cis zwitterion **7-E-C**[a]

Structure	ΔE	ΔH	ΔG	ΔG (GV50)	ΔΔG
**7-E-C-C1**	-1131.280029	-1130.890687	-1130.97235	-1130.968938	0.00

**7-E-C-C2**	-1131.279698	-1130.889941	-1130.971489	-1130.968166	0.48
**7-E-C-C3**	-1131.279599	-1130.889997	-1130.97129	-1130.968049	0.56
**7-E-C-C4**	-1131.278432	-1130.888163	-1130.967698	-1130.965334	2.26

[a]Energies were obtained as single points at the PW6B95(D3BJ) / def2-TZVPPD / SMD (DCM) level of theory based on structures optimized at the MN15 / def2-TZVP / SMD (DCM) level of theory. ΔE, ΔH, ΔG, and ΔG (GV50), hartree; ΔΔG, kcal/mol.

7-K-T

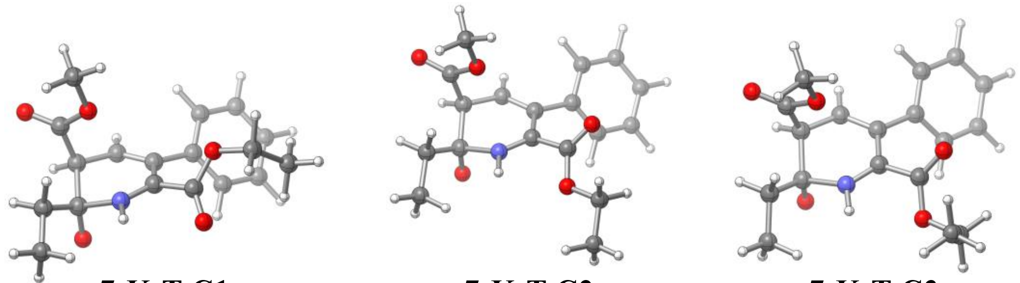

7-K-T-C1 **7-K-T-C2** **7-K-T-C3**

Figure S23. Conformers of the ketone cyclization trans zwitterion **7-K-T**

Table S22. Energies of conformers of ketone cyclization trans zwitterion **7-K-T**[a]

Structure	ΔE	ΔH	ΔG	ΔG (GV50)	ΔΔG
**7-K-T-C1**	-1131.292882	-1130.902452	-1130.983396	-1130.979933	0.00
**7-K-T-C2**	-1131.292506	-1130.901975	-1130.98232	-1130.979719	0.13
**7-K-T-C3**	-1131.292007	-1130.901402	-1130.981582	-1130.978962	0.61

[a]Energies were obtained as single points at the PW6B95(D3BJ) / def2-TZVPPD / SMD (DCM) level of theory based on structures optimized at the MN15 / def2-TZVP / SMD (DCM) level of theory. ΔE, ΔH, ΔG, and ΔG (GV50), hartree; ΔΔG, kcal/mol.

7-K-C

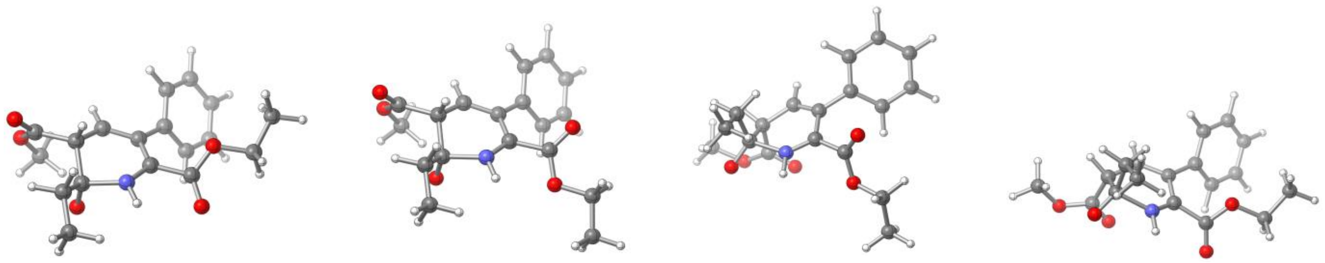

7-K-C-C1 7-K-C-C2 7-K-C-C3 7-K-C-C4

Figure S24. Conformers of the ketone cyclization cis zwitterion **7-K-C**

Table S23. Energies of conformers of ketone cyclization cis zwitterion **7-K-C**[a]

Structure	ΔE	ΔH	ΔG	ΔG (GV50)	ΔΔG
**7-K-C-C1**	-1131.286255	-1130.895797	-1130.974614	-1130.9723	0.00
**7-K-C-C2**	-1131.284978	-1130.894603	-1130.974559	-1130.971751	0.34
**7-K-C-C3**	-1131.282596	-1130.892263	-1130.972633	-1130.969883	1.52
**7-K-C-C4**	-1131.282558	-1130.892213	-1130.971671	-1130.969331	1.86

[a]Energies were obtained as single points at the PW6B95(D3BJ) / def2-TZVPPD / SMD (DCM) level of theory based on structures optimized at the MN15 / def2-TZVP / SMD (DCM) level of theory. ΔE, ΔH, ΔG, and ΔG (GV50), hartree; ΔΔG, kcal/mol.

7-Cs-E-T

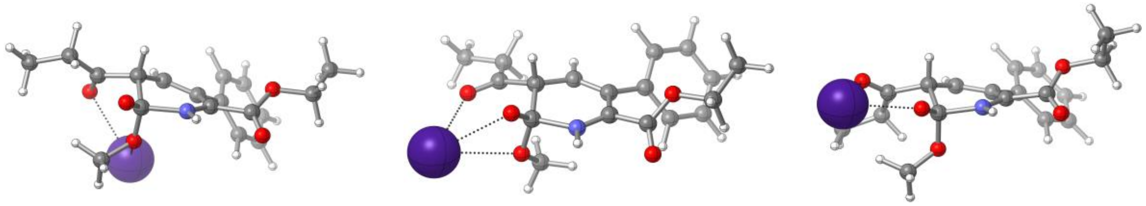

7-Cs-E-T-C1 7-Cs-E-T-C2 7-Cs-E-T-C3

Figure S25. Conformers of cesium-coordinated ester cyclization trans zwitterion **7-Cs-E-T**

Table S24. Energies of conformers of cesium-coordinated ester cyclization trans zwitterion **7-Cs-E-T**[a]

Structure	ΔE	ΔH	ΔG	ΔG (GV50)	ΔΔG
**7-Cs-E-T-C1**	-1151.387009	-1150.994511	-1151.083205	-1151.078839	0.00

7-Cs-E-T-C2	-1151.387394	-1150.994779	-1151.081461	-1151.077577	0.79
**7-Cs-E-T-C3**	-1151.387523	-1150.994716	-1151.081012	-1151.077543	0.81

[a]Energies were obtained as single points at the PW6B95(D3BJ) / def2-TZVPPD / SMD (DCM) level of theory based on structures optimized at the MN15 / def2-TZVP / SMD (DCM) level of theory. ΔE, ΔH, ΔG, and ΔG (GV50), hartree; ΔΔG, kcal/mol.

7-Cs-E-C

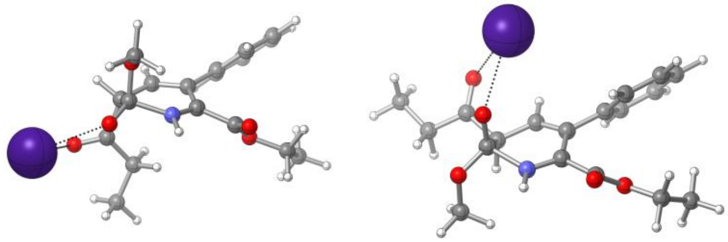

7-Cs-E-C1 **7-Cs-E-C2**

Figure S26. Conformers of cesium-coordinated ester cyclization cis zwitterion **7-Cs-E-C**

Table S25. Energies of conformers of cesium-coordinated ester cyclization cis zwitterion **7-Cs-E-C**[a]

Structure	ΔE	ΔH	ΔG	ΔG (GV50)	ΔΔG
**7-Cs-E-C-C1**	-1151.3915	-1150.998894	-1151.088153	-1151.083507	0.00
**7-Cs-E-C-C2**	-1151.392287	-1150.999742	-1151.087399	-1151.083429	0.05

[a]Energies were obtained as single points at the PW6B95(D3BJ) / def2-TZVPPD / SMD (DCM) level of theory based on structures optimized at the MN15 / def2-TZVP / SMD (DCM) level of theory. ΔE, ΔH, ΔG, and ΔG (GV50), hartree; ΔΔG, kcal/mol.

7-Cs-K-T

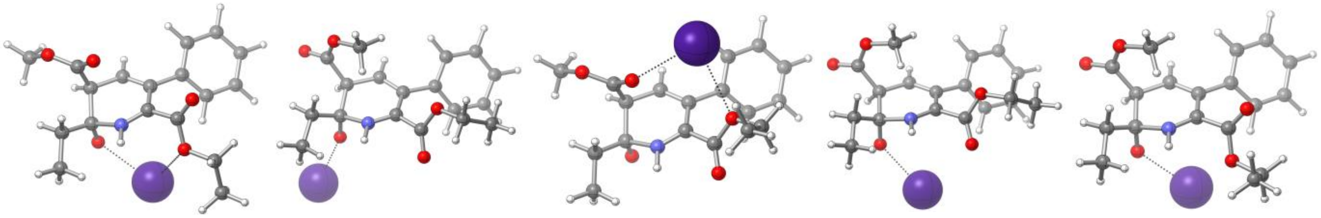

7-Cs-K-T-C1 **7-Cs-K-T-C2** **7-Cs-K-T-C3** **7-Cs-K-T-C4** **7-Cs-K-T-C5**

Figure S27. Conformers of cesium-coordinated ketone cyclization trans zwitterion **7-Cs-K-T**

Table S26. Energies of conformers of cesium-coordinated ketone cyclization trans zwitterion **7-Cs-K-T**[a]

Structure	ΔE	ΔH	ΔG	ΔG (GV50)	ΔΔG
**7-Cs-K-T-C1**	-1151.38841	-1150.993995	-1151.077868	-1151.075694	0.00
**7-Cs-K-T-C2**	-1151.384137	-1150.9897	-1151.0764	-1151.072137	2.23
**7-Cs-K-T-C3**	-1151.38519	-1150.990722	-1151.074451	-1151.072063	2.28
**7-Cs-K-T-C4**	-1151.383714	-1150.989291	-1151.073753	-1151.070826	3.05
**7-Cs-K-T-C5**	-1151.381412	-1150.98702	-1151.071301	-1151.068553	4.48

7-Cs-K-C

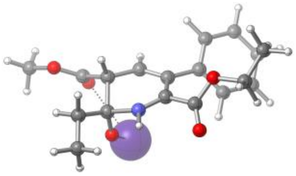

7-Cs-K-C-C1

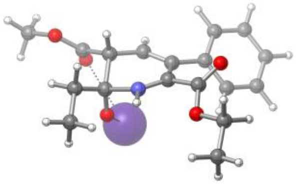

7-Cs-K-C-C2

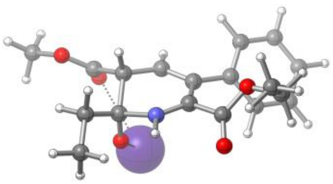

7-Cs-K-C-C3

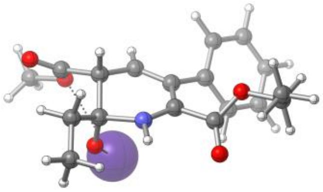

7-Cs-K-C-C4

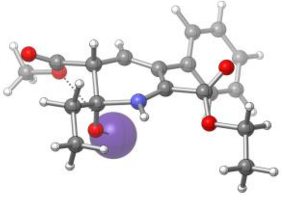

7-Cs-K-C-C5

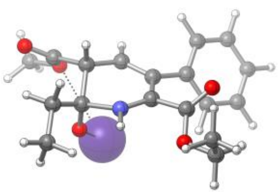

7-Cs-K-C-C6

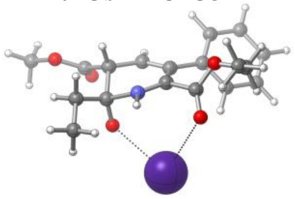

7-Cs-K-C-C7

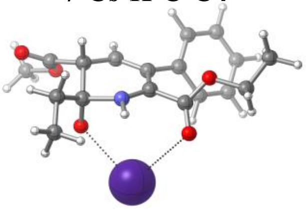

7-Cs-K-C-C8

Figure S28. Conformers of cesium-coordinated ketone cyclization cis zwitterion **7-Cs-K-C**

Table S27. Energies of conformers of cesium-coordinated ketone cyclization cis zwitterion **7-Cs-K-C**[a]

Structure	ΔE	ΔH	ΔG	ΔG (GV50)	ΔΔG
**7-Cs-K-C-C1**	-1151.405361	-1151.011357	-1151.098298	-1151.094438	0.00
**7-Cs-K-C-C2**	-1151.403936	-1151.009852	-1151.097115	-1151.093215	0.77
**7-Cs-K-C-C3**	-1151.404194	-1151.010077	-1151.097222	-1151.093118	0.83
**7-Cs-K-C-C4**	-1151.401705	-1151.007492	-1151.094033	-1151.090405	2.53
**7-Cs-K-C-C5**	-1151.4011	-1151.007184	-1151.092999	-1151.090201	2.66
**7-Cs-K-C-C6**	-1151.400965	-1151.006688	-1151.092327	-1151.089188	3.29
**7-Cs-K-C-C7**	-1151.39901	-1151.005086	-1151.09353	-1151.08904	3.39

7-Cs-K-C-C8	-1151.399391	-1151.00554	-1151.092655	-1151.088706	3.60

[a]Energies were obtained as single points at the PW6B95(D3BJ) / def2-TZVPPD / SMD (DCM) level of theory based on structures optimized at the MN15 / def2-TZVP / SMD (DCM) level of theory. ΔE, ΔH, ΔG, and ΔG (GV50), hartree; ΔΔG, kcal/mol.

7-Na-E-T

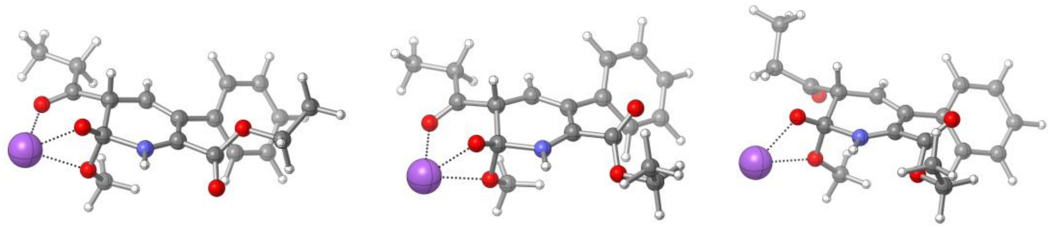

7-Na-E-T-C1 **7-Na-E-T-C2** **7-Na-E-T-C3**

Figure S29. Conformers of sodium-coordinated ester cyclization trans zwitterion **7-Na-E-T**

Table S28. Energies of conformers of sodium-coordinated ester cyclization trans zwitterion **7-Na-E-T**[a]

Structure	ΔE	ΔH	ΔG	ΔG (GV50)	ΔΔG
**7-Na-E-T-C1**	-1293.690763	-1293.297521	-1293.380594	-1293.377208	0.00
**7-Na-E-T-C2**	-1293.688945	-1293.295473	-1293.37647	-1293.374172	1.90
**7-Na-E-T-C3**	-1293.67976	-1293.286083	-1293.370204	-1293.366713	6.59

[a]Energies were obtained as single points at the PW6B95(D3BJ) / def2-TZVPPD / SMD (DCM) level of theory based on structures optimized at the MN15 / def2-TZVP / SMD (DCM) level of theory. ΔE, ΔH, ΔG, and ΔG (GV50), hartree; ΔΔG, kcal/mol.

7-Na-E-C

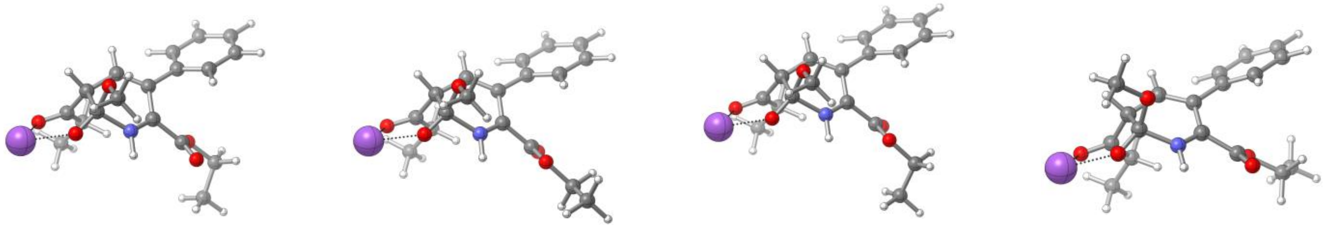

7-Na-E-C-C1 **7-Na-E-C-C2** **7-Na-E-C-C3** **7-Na-E-C-C4**

Figure S30. Conformers of sodium-coordinated ester cyclization cis zwitterion **7-Na-E-C**

Table S29. Energies of conformers of sodium-coordinated ester cyclization cis zwitterion **7-Na-E-C**[a]

Structure	ΔE	ΔH	ΔG	ΔG (GV50)	ΔΔG
**7-Na-E-C-C1**	-1293.689303	-1293.296063	-1293.379956	-1293.376738	0.00
**7-Na-E-C-C2**	-1293.689222	-1293.296017	-1293.379457	-1293.376619	0.07
**7-Na-E-C-C3**	-1293.688758	-1293.295534	-1293.378991	-1293.376066	0.42
**7-Na-E-C-C4**	-1293.687808	-1293.294649	-1293.378254	-1293.375061	1.05

[a]Energies were obtained as single points at the PW6B95(D3BJ) / def2-TZVPPD / SMD (DCM) level of theory based on structures optimized at the MN15 / def2-TZVP / SMD (DCM) level of theory. ΔE, ΔH, ΔG, and ΔG (GV50), hartree; ΔΔG, kcal/mol.

7-Na-K-T

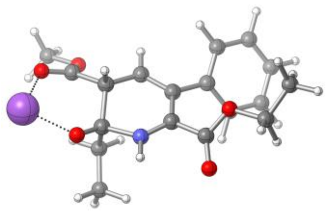

7-Na-K-T-C1

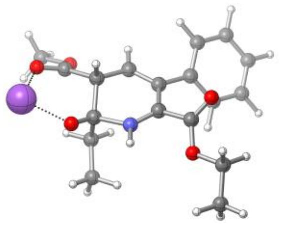

7-Na-K-T-C2

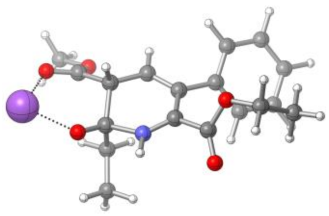

7-Na-K-T-C3

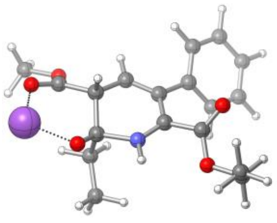

7-Na-K-T-C4

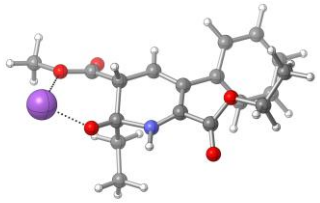

7-Na-K-T-C5

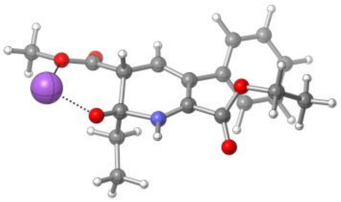

7-Na-K-T-C6

Figure S31. Conformers of sodium-coordinated ketone cyclization trans zwitterion **7-Na-K-T**

Table S30. Energies of conformers of sodium-coordinated ketone cyclization trans zwitterion **7-Na-K-T**[a]

Structure	ΔE	ΔH	ΔG	ΔG (GV50)	ΔΔG
**7-Na-K-T-C1**	-1293.702579	-1293.308272	-1293.392141	-1293.389103	0.00
**7-Na-K-T-C2**	-1293.701098	-1293.306942	-1293.391318	-1293.388068	0.65
**7-Na-K-T-C3**	-1293.701874	-1293.30756	-1293.390868	-1293.388011	0.69
**7-Na-K-T-C4**	-1293.70072	-1293.306273	-1293.389534	-1293.386776	1.46
**7-Na-K-T-C5**	-1293.697157	-1293.302892	-1293.386388	-1293.383571	3.47

**7-Na-K-T-C6**	-1293.696394	-1293.302099	-1293.384749	-1293.382255	4.30

[a]Energies were obtained as single points at the PW6B95(D3BJ) / def2-TZVPPD / SMD (DCM) level of theory based on structures optimized at the MN15 / def2-TZVP / SMD (DCM) level of theory. ΔE, ΔH, ΔG, and ΔG (GV50), hartree; ΔΔG, kcal/mol.

7-Na-K-C

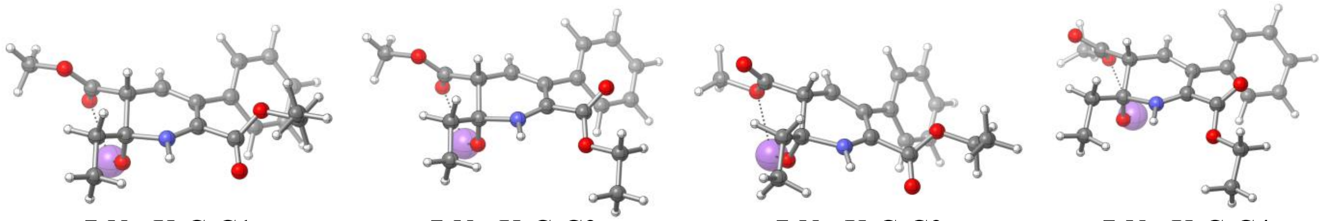

7-Na-K-C-C1 **7-Na-K-C-C2** **7-Na-K-C-C3** **7-Na-K-C-C4**

Figure S32. Conformers of sodium-coordinated ketone cyclization cis zwitterion **7-Na-K-C**

Table S31. Energies of conformers of sodium-coordinated ketone cyclization cis zwitterion **7-Na-K-C**[a]

Structure	ΔE	ΔH	ΔG	ΔG (GV50)	ΔΔG
**7-Na-K-C-C1**	-1293.702982	-1293.308205	-1293.391529	-1293.388466	0.00
**7-Na-K-C-C2**	-1293.702844	-1293.307994	-1293.390561	-1293.387995	0.30
**7-Na-K-C-C3**	-1293.696666	-1293.302098	-1293.384951	-1293.382424	3.79
**7-Na-K-C-C4**	-1293.696511	-1293.301949	-1293.384419	-1293.382145	3.97

[a]Energies were obtained as single points at the PW6B95(D3BJ) / def2-TZVPPD / SMD (DCM) level of theory based on structures optimized at the MN15 / def2-TZVP / SMD (DCM) level of theory. ΔE, ΔH, ΔG, and ΔG (GV50), hartree; ΔΔG, kcal/mol.

7b-E-T

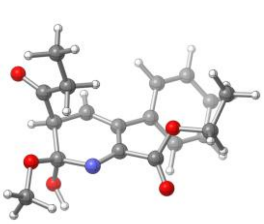

7b-E-T-C1

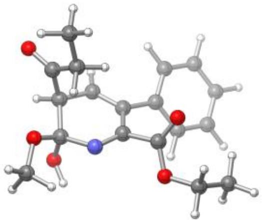

7b-E-T-C2

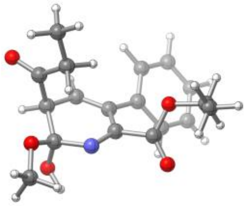

7b-E-T-C3

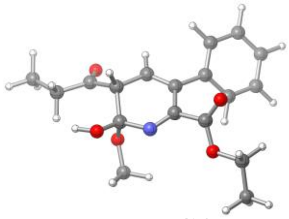

7b-E-T-C4

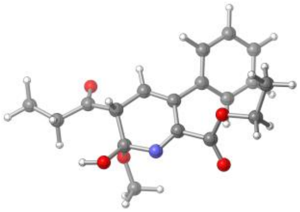

7b-E-T-C5

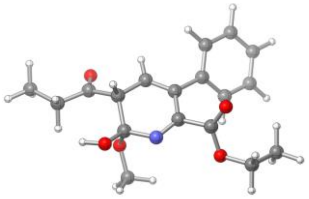

7b-E-T-C6

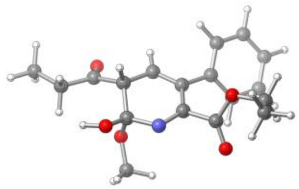

7b-E-T-C7

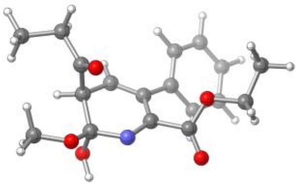

7b-E-T-C8

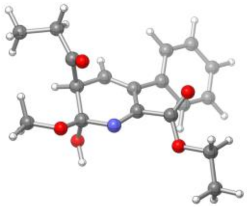

7b-E-T-C9

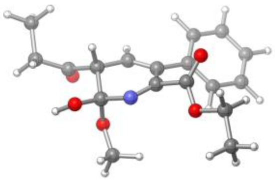

7b-E-T-C10

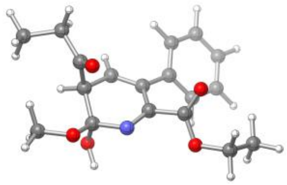

7b-E-T-C11

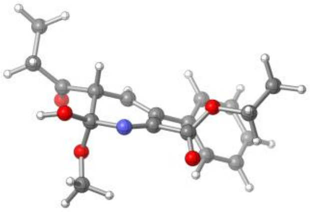

7b-E-T-C12

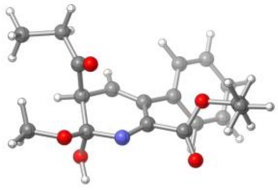

7b-E-T-C13

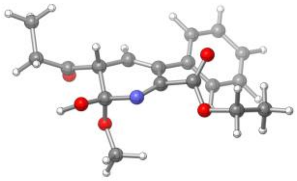

7b-E-T-C14

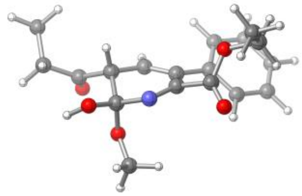

7b-E-T-C15

Figure S33. Conformers of ester cyclization trans intermediate **7b-E-T**.

Table S32. Energies of conformers of ester cyclization trans intermediate **7b-E-T**. [a]

Structure	ΔE	ΔH	ΔG	ΔG (GV50)	ΔΔG
**7b-E-T-C1**	-1131.307293	-1130.916683	-1130.996557	-1130.993753	0.00
**7b-E-T-C2**	-1131.306542	-1130.915885	-1130.995522	-1130.992876	0.55
**7b-E-T-C3**	-1131.30617	-1130.915425	-1130.995073	-1130.99241	0.84
**7b-E-T-C4**	-1131.302129	-1130.911645	-1130.992008	-1130.989077	2.93
**7b-E-T-C5**	-1131.302403	-1130.911847	-1130.991695	-1130.988891	3.05
**7b-E-T-C6**	-1131.301914	-1130.911358	-1130.990935	-1130.988308	3.42
**7b-E-T-C7**	-1131.301228	-1130.910491	-1130.990297	-1130.987478	3.94

**7b-E-T-C8**	-1131.301607	-1130.910899	-1130.989902	-1130.98733	4.03
**7b-E-T-C9**	-1131.301051	-1130.910474	-1130.989755	-1130.98728	4.06
**7b-E-T-C10**	-1131.29982	-1130.909024	-1130.989393	-1130.986583	4.50
**7b-E-T-C11**	-1131.3008	-1130.910074	-1130.988384	-1130.98628	4.69
**7b-E-T-C12**	-1131.300078	-1130.909299	-1130.988727	-1130.986248	4.71
**7b-E-T-C13**	-1131.300399	-1130.909594	-1130.988441	-1130.985939	4.90
**7b-E-T-C14**	-1131.299619	-1130.908811	-1130.988076	-1130.985675	5.07
**7b-E-T-C15**	-1131.29884	-1130.908034	-1130.988451	-1130.985277	5.32

[a]Energies were obtained as single points at the PW6B95(D3BJ) / def2-TZVPPD / SMD (DCM) level of theory based on structures optimized at the MN15 / def2-TZVP / SMD (DCM) level of theory. ΔE, ΔH, ΔG, and ΔG (GV50), hartree; ΔΔG, kcal/mol.

7b-E-C

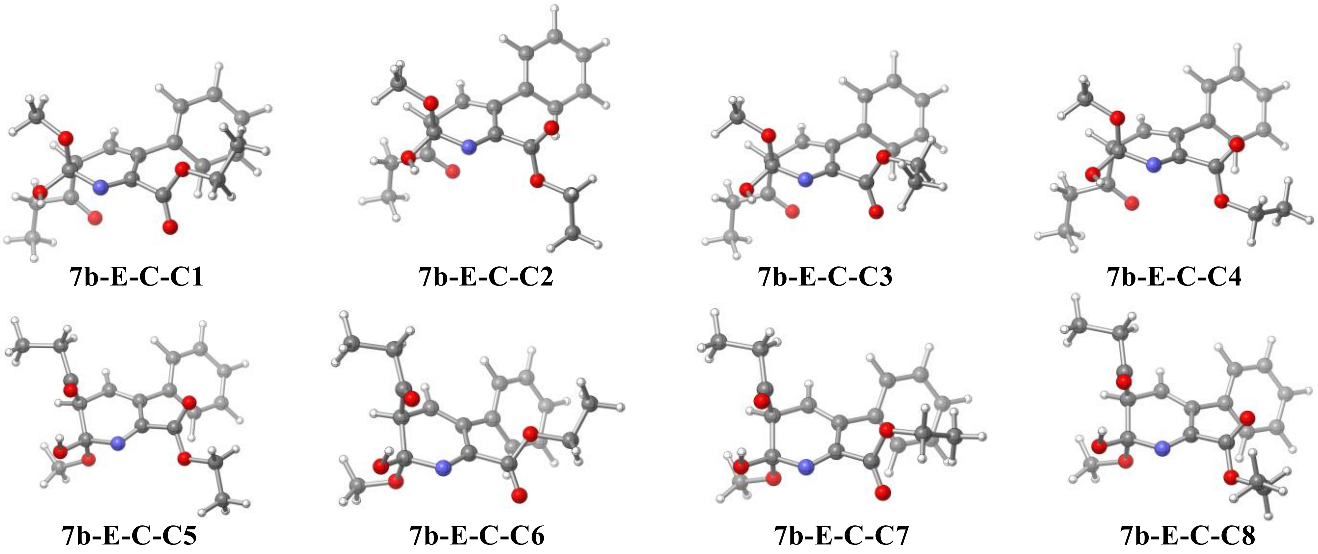

Figure S34. Conformers of ester cyclization cis intermediate **7b-E-C**.

Table S33. Energies of conformers of ester cyclization cis intermediate **7b-E-C**. [a]

Structure	ΔE	ΔH	ΔG	ΔG (GV50)	ΔΔG
**7b-E-C-C1**	-1131.30287	-1130.912383	-1130.992676	-1130.989573	0.00
**7b-E-C-C2**	-1131.302691	-1130.911955	-1130.991872	-1130.988916	0.41

**7b-E-C-C3**	-1131.301856	-1130.911192	-1130.991422	-1130.988345	0.77
**7b-E-C-C4**	-1131.302487	-1130.911712	-1130.99088	-1130.98817	0.88
**7b-E-C-C5**	-1131.300679	-1130.909622	-1130.989712	-1130.986772	1.76
**7b-E-C-C6**	-1131.300834	-1130.909761	-1130.989626	-1130.986527	1.91
**7b-E-C-C7**	-1131.300525	-1130.909562	-1130.988869	-1130.985894	2.31
**7b-E-C-C8**	-1131.300079	-1130.908941	-1130.988878	-1130.985821	2.35

[a]Energies were obtained as single points at the PW6B95(D3BJ) / def2-TZVPPD / SMD (DCM) level of theory based on structures optimized at the MN15 / def2-TZVP / SMD (DCM) level of theory. ΔE, ΔH, ΔG, and ΔG (GV50), hartree; ΔΔG, kcal/mol.

7b-K-T

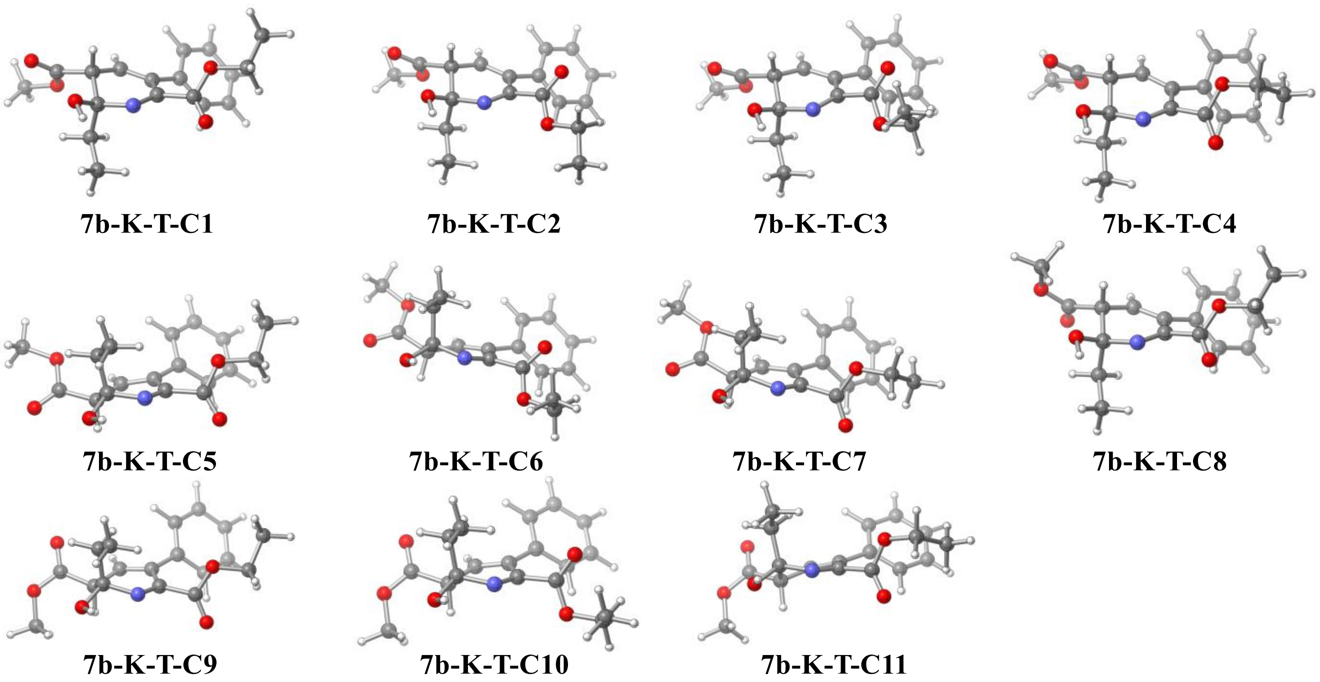

Figure S35. Conformers of ketone cyclization trans intermediate **7b-K-T**.

Table S34. Energies of conformers of ketone cyclization trans intermediate **7b-K-T**.[a]

Structure	Δ*E*	Δ*H*	Δ*G*	Δ*G* (GV50)	ΔΔ*G*
**7b-K-T-C1**	-1131.324063	-1130.932801	-1131.012585	-1131.010013	0.00
**7b-K-T-C2**	-1131.323542	-1130.9323	-1131.012369	-1131.0097	0.20

**7b-K-T-C3**	-1131.322876	-1130.931579	-1131.011849	-1131.008929	0.68
**7b-K-T-C4**	-1131.323612	-1130.932264	-1131.011088	-1131.008926	0.68
**7b-K-T-C5**	-1131.320677	-1130.929699	-1131.010148	-1131.007291	1.71
**7b-K-T-C6**	-1131.319884	-1130.928558	-1131.008495	-1131.005735	2.68
**7b-K-T-C7**	-1131.320005	-1130.928734	-1131.008239	-1131.00559	2.78
**7b-K-T-C8**	-1131.317387	-1130.926038	-1131.004712	-1131.002681	4.60
**7b-K-T-C9**	-1131.31374	-1130.922602	-1131.002495	-1130.999853	6.38
**7b-K-T-C10**	-1131.312726	-1130.9214	-1131.002179	-1130.998984	6.92
**7b-K-T-C11**	-1131.313067	-1130.921708	-1131.001315	-1130.998545	7.20

[a]Energies were obtained as single points at the PW6B95(D3BJ) / def2-TZVPPD / SMD (DCM) level of theory based on structures optimized at the MN15 / def2-TZVP / SMD (DCM) level of theory. ΔE, ΔH, ΔG, and ΔG (GV50), hartree; ΔΔG, kcal/mol.

7b-K-C

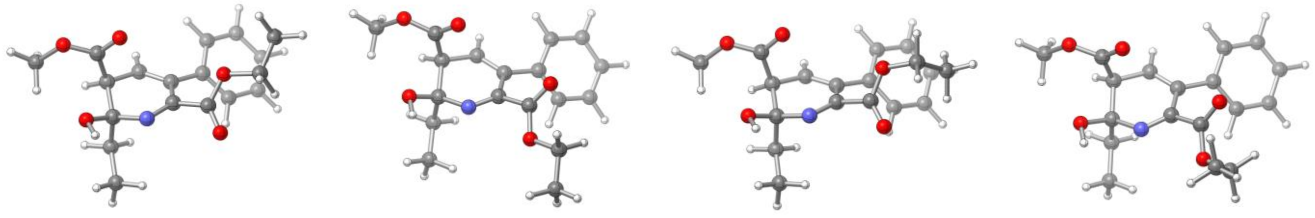

7b-K-C-C1 **7b-K-C-C2** **7b-K-C-C3** **7b-K-C-C4**

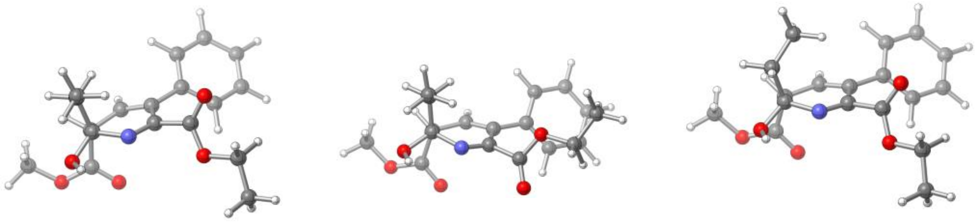

7b-K-C-C_5 **7b-K-C-C6** **7b-K-C-C7**

Figure S36. Conformers of ketone cyclization cis intermediate **7b-K-C**.

Table S35. Energies of conformers of ketone cyclization cis intermediate **7b-K-C**.[a]

Structure	ΔE	ΔH	ΔG	ΔG (GV50)	ΔΔG
**7b-K-C-C1**	-1131.318139	-1130.926748	-1131.006283	-1131.003659	0.00
**7b-K-C-C2**	-1131.317236	-1130.925845	-1131.005333	-1131.002849	0.51

**7b-K-C-C3**	-1131.317632	-1130.926213	-1131.005063	-1131.00276	0.56
**7b-K-C-C4**	-1131.316644	-1130.925136	-1131.004291	-1131.001865	1.13
**7b-K-C-C5**	-1131.314503	-1130.923161	-1131.00325	-1131.000344	2.08
**7b-K-C-C6**	-1131.314515	-1130.923328	-1131.002632	-1131.000216	2.16
**7b-K-C-C7**	-1131.314822	-1130.923425	-1131.002202	-1130.999593	2.55

[a]Energies were obtained as single points at the PW6B95(D3BJ) / def2-TZVPPD / SMD (DCM) level of theory based on structures optimized at the MN15 / def2-TZVP / SMD (DCM) level of theory. ΔE, ΔH, ΔG, and ΔG (GV50), hartree; ΔΔG, kcal/mol.

7b-Cs-E-T

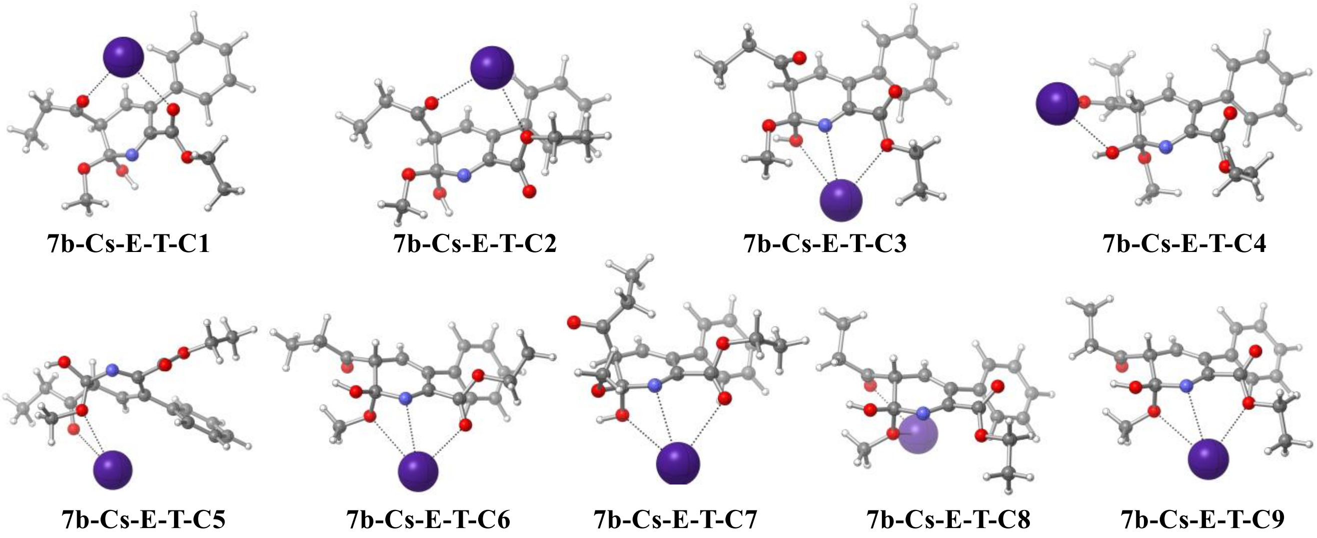

Figure S37. Conformers of cesium-coordinated ester cyclization trans intermediate **7b-Cs-E-T**.

Table S36. Energies of conformers of cesium-coordinated ester cyclization trans intermediate **7b-Cs-E-T**.[a]

Structure	ΔE	ΔH	ΔG	ΔG (GV50)	ΔΔG
**7b-Cs-E-T-C1**	-1151.408106	-1151.014243	-1151.101014	-1151.097741	0.00
**7b-Cs-E-T-C2**	-1151.405469	-1151.011552	-1151.096958	-1151.094135	2.26
**7b-Cs-E-T-C3**	-1151.404135	-1151.010165	-1151.095909	-1151.092836	3.08
**7b-Cs-E-T-C4**	-1151.400011	-1151.005953	-1151.093706	-1151.089887	4.93
**7b-Cs-E-T-C5**	-1151.399802	-1151.006218	-1151.090792	-1151.087885	6.18

**7b-Cs-E-T-C6**	-1151.398933	-1151.005312	-1151.090542	-1151.087701	6.30
**7b-Cs-E-T-C7**	-1151.399249	-1151.006569	-1151.089864	-1151.087603	6.36
**7b-Cs-E-T-C8**	-1151.397494	-1151.003751	-1151.090256	-1151.086766	6.89
**7b-Cs-E-T-C9**	-1151.39588	-1151.001861	-1151.086728	-1151.084545	8.28

[a]Energies were obtained as single points at the PW6B95(D3BJ) / def2-TZVPPD / SMD (DCM) level of theory based on structures optimized at the MN15 / def2-TZVP / SMD (DCM) level of theory. ΔE, ΔH, ΔG, and ΔG (GV50), hartree; ΔΔG, kcal/mol.

7b-Cs-E-C

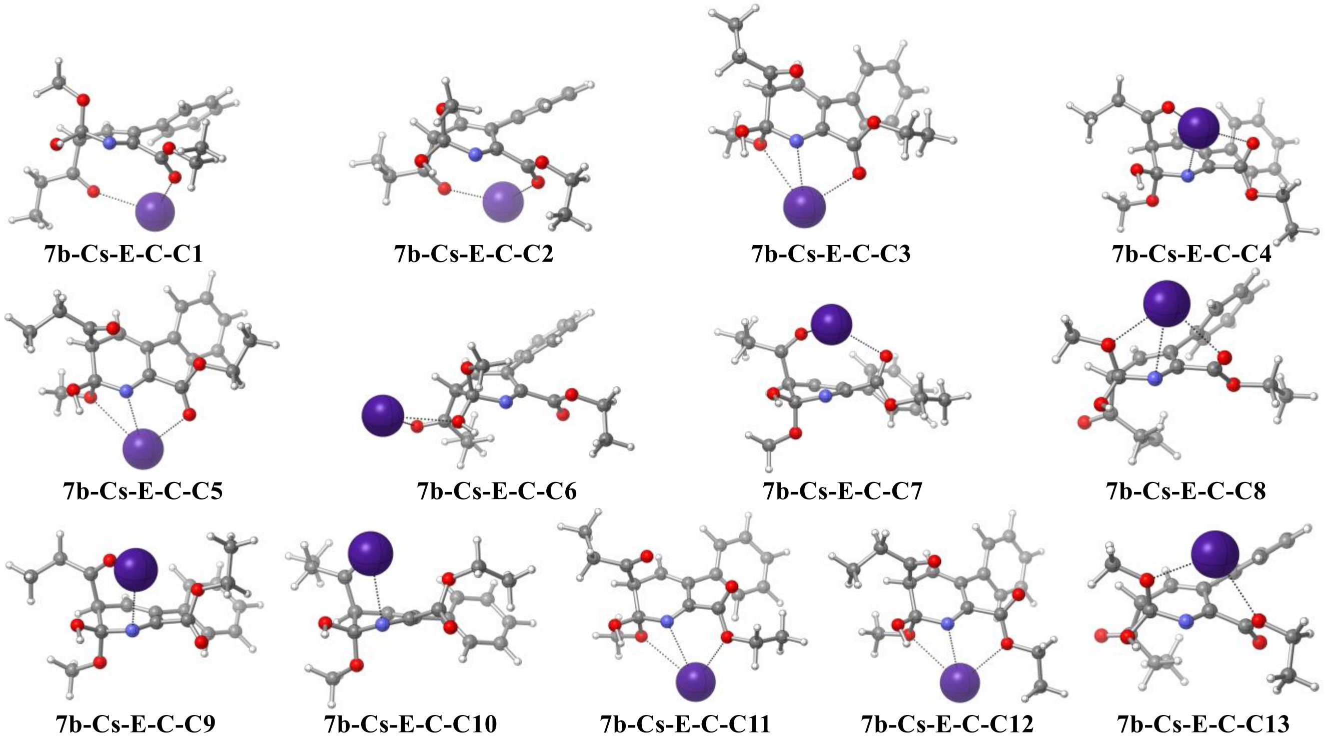

Figure S38. Conformers of cesium-coordinated ester cyclization cis intermediate **7b-Cs-E-C**.

Table S37. Energies of conformers of cesium-coordinated ester cyclization cis intermediate **7b-Cs-E-C**.[a]

Structure	ΔE	ΔH	ΔG	ΔG (GV50)	ΔΔG
**7b-Cs-E-C-C1**	-1151.410611	-1151.016717	-1151.103733	-1151.099996	0.00
**7b-Cs-E-C-C2**	-1151.40865	-1151.014482	-1151.102141	-1151.098273	1.08
**7b-Cs-E-C-C3**	-1151.406214	-1151.011933	-1151.097577	-1151.094794	3.26

**7b-Cs-E-C-C4**	-1151.405165	-1151.011058	-1151.098361	-1151.094493	3.45
**7b-Cs-E-C-C5**	-1151.40533	-1151.011152	-1151.097165	-1151.094162	3.66
**7b-Cs-E-C-C6**	-1151.404505	-1151.010382	-1151.097517	-1151.093956	3.79
**7b-Cs-E-C-C7**	-1151.405399	-1151.010938	-1151.097472	-1151.093778	3.90
**7b-Cs-E-C-C8**	-1151.40516	-1151.010764	-1151.09678	-1151.093666	3.97
**7b-Cs-E-C-C9**	-1151.404489	-1151.010328	-1151.096346	-1151.093289	4.21
**7b-Cs-E-C-C10**	-1151.405261	-1151.010858	-1151.095715	-1151.093046	4.36
**7b-Cs-E-C-C11**	-1151.403552	-1151.009252	-1151.095432	-1151.092217	4.88
**7b-Cs-E-C-C12**	-1151.403297	-1151.009092	-1151.094906	-1151.092135	4.93
**7b-Cs-E-C-C13**	-1151.402292	-1151.00806	-1151.094983	-1151.091417	5.38

[a]Energies were obtained as single points at the PW6B95(D3BJ) / def2-TZVPPD / SMD (DCM) level of theory based on structures optimized at the MN15 / def2-TZVP / SMD (DCM) level of theory. ΔE, ΔH, ΔG, and ΔG (GV50), hartree; ΔΔG, kcal/mol.

7b-Cs-K-T

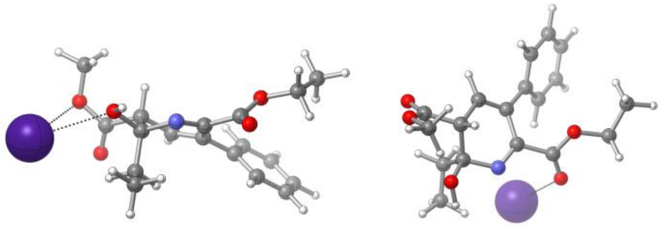

7b-Cs-K-T-C1 **7b-Cs-K-T-C2**

Figure S39. Conformers of cesium-coordinated ketone cyclization trans intermediate **7b-Cs-K-T**.

Table S38. Energies of conformers of cesium-coordinated ketone cyclization trans intermediate **7b-Cs-K-T**.[a]

Structure	ΔE	ΔH	ΔG	ΔG (GV50)	ΔΔG
**7b-Cs-K-T-C1**	-1151.416007	-1151.021834	-1151.109092	-1151.105355	0.00
**7b-Cs-K-T-C2**	-1151.41427	-1151.019559	-1151.105332	-1151.102053	2.07

[a]Energies were obtained as single points at the PW6B95(D3BJ) / def2-TZVPPD / SMD (DCM) level of theory based on structures optimized at the MN15 / def2-TZVP / SMD (DCM) level of theory. ΔE, ΔH, ΔG, and ΔG (GV50), hartree; ΔΔG, kcal/mol.

7b-Cs-K-C

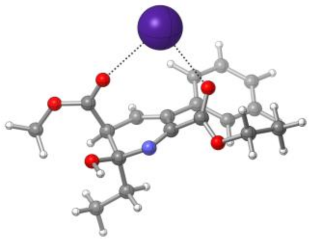

7b-Cs-K-C-C1

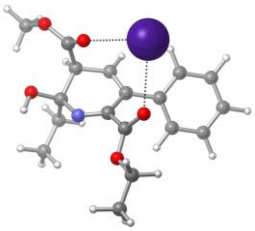

7b-Cs-K-C-C2

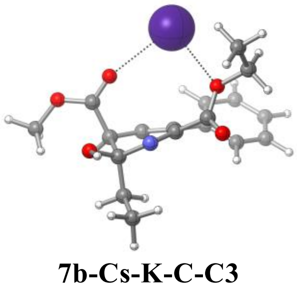

7b-Cs-K-C-C3

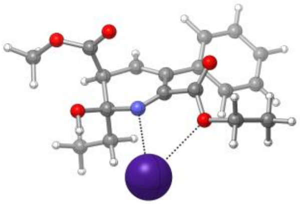

7b-Cs-K-C-C4

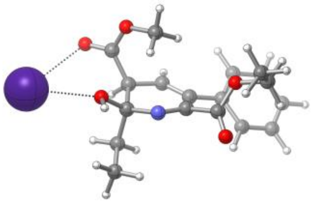

7b-Cs-K-C-C5

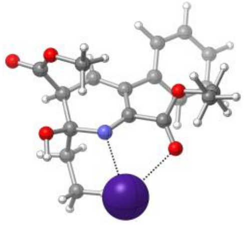

7b-Cs-K-C-C6

Figure S40. Conformers of cesium-coordinated ketone cyclization cis intermediate **7b-Cs-K-C**.

Table S39. Energies of conformers of cesium-coordinated ketone cyclization cis intermediate **7b-Cs-K-C**.[a]

Structure	ΔE	ΔH	ΔG	ΔG (GV50)	ΔΔG
**7b-Cs-K-C-C1**	-1151.423624	-1151.028927	-1151.114179	-1151.111496	0.00
**7b-Cs-K-C-C2**	-1151.423408	-1151.028722	-1151.114443	-1151.11142	0.05
**7b-Cs-K-C-C3**	-1151.420259	-1151.025543	-1151.110093	-1151.107469	2.53
**7b-Cs-K-C-C4**	-1151.414741	-1151.019973	-1151.105694	-1151.102788	5.46
**7b-Cs-K-C-C5**	-1151.413019	-1151.018234	-1151.104039	-1151.100872	6.67
**7b-Cs-K-C-C6**	-1151.412621	-1151.017725	-1151.102629	-1151.099637	7.44

[a]Energies were obtained as single points at the PW6B95(D3BJ) / def2-TZVPPD / SMD (DCM) level of theory based on structures optimized at the MN15 / def2-TZVP / SMD (DCM) level of theory. ΔE, ΔH, ΔG, and ΔG (GV50), hartree; ΔΔG, kcal/mol.

7b-Na-E-T

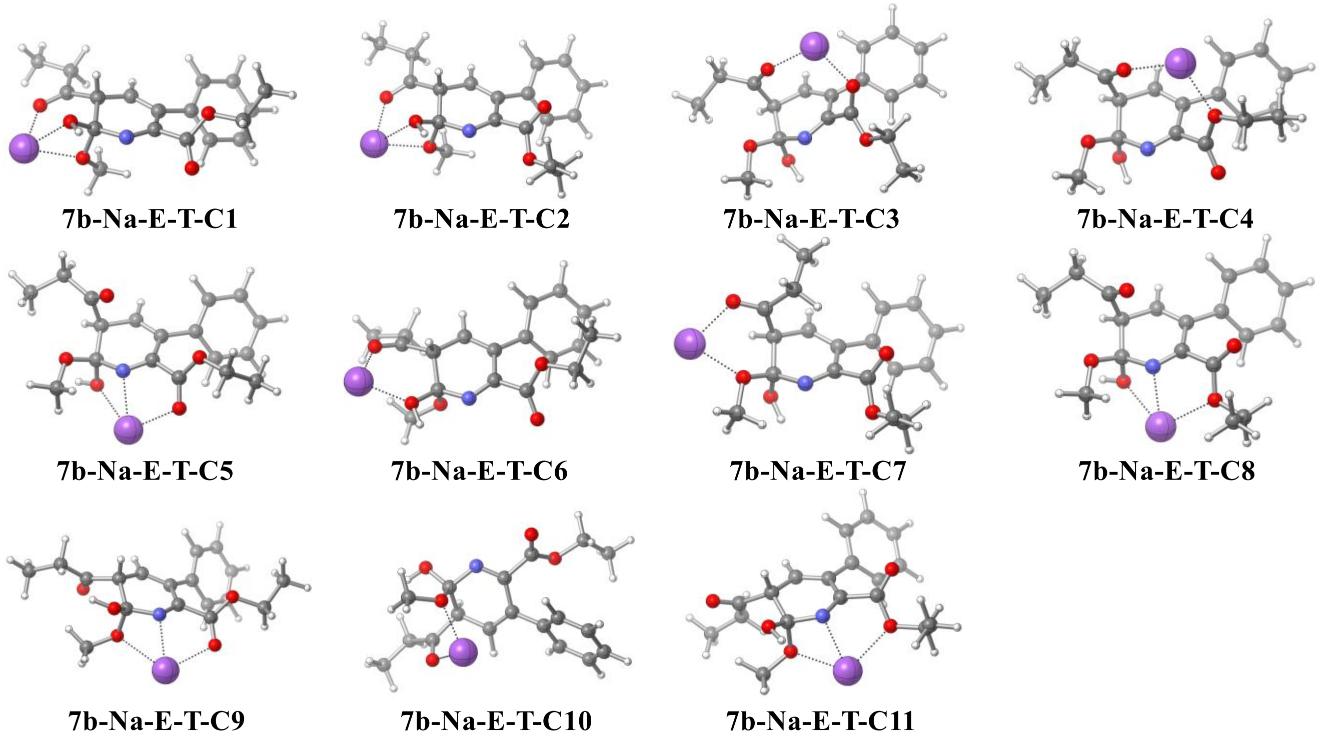

Figure S41. Conformers of sodium-coordinated ester cyclization trans intermediate **7b-Na-E-T**.

Table S40. Energies of conformers of sodium-coordinated ester cyclization trans intermediate **7b-Na-E-T**.[a]

Structure	ΔE	ΔH	ΔG	ΔG (GV50)	ΔΔG
**7b-Na-E-T-C1**	-1293.704301	-1293.310743	-1293.392234	-1293.3899	0.00
**7b-Na-E-T-C2**	-1293.703559	-1293.309746	-1293.391551	-1293.388892	0.63
**7b-Na-E-T-C3**	-1293.700853	-1293.306582	-1293.390343	-1293.387541	1.48
**7b-Na-E-T-C4**	-1293.695294	-1293.301481	-1293.383779	-1293.38172	5.13
**7b-Na-E-T-C5**	-1293.695113	-1293.300898	-1293.383297	-1293.381276	5.41
**7b-Na-E-T-C6**	-1293.694318	-1293.300526	-1293.38244	-1293.380374	5.98
**7b-Na-E-T-C7**	-1293.693394	-1293.299145	-1293.383041	-1293.38026	6.05
**7b-Na-E-T-C8**	-1293.690765	-1293.29675	-1293.380648	-1293.377966	7.49
**7b-Na-E-T-C9**	-1293.690821	-1293.296856	-1293.379276	-1293.377038	8.07
**7b-Na-E-T-C10**	-1293.689561	-1293.295775	-1293.37766	-1293.375597	8.98

**7b-Na-E-T-C11**	-1293.688103	-1293.294384	-1293.377299	-1293.374812	9.47

[a]Energies were obtained as single points at the PW6B95(D3BJ) / def2-TZVPPD / SMD (DCM) level of theory based on structures optimized at the MN15 / def2-TZVP / SMD (DCM) level of theory. ΔE, ΔH, ΔG, and ΔG (GV50), hartree; ΔΔG, kcal/mol.

7b-Na-E-C

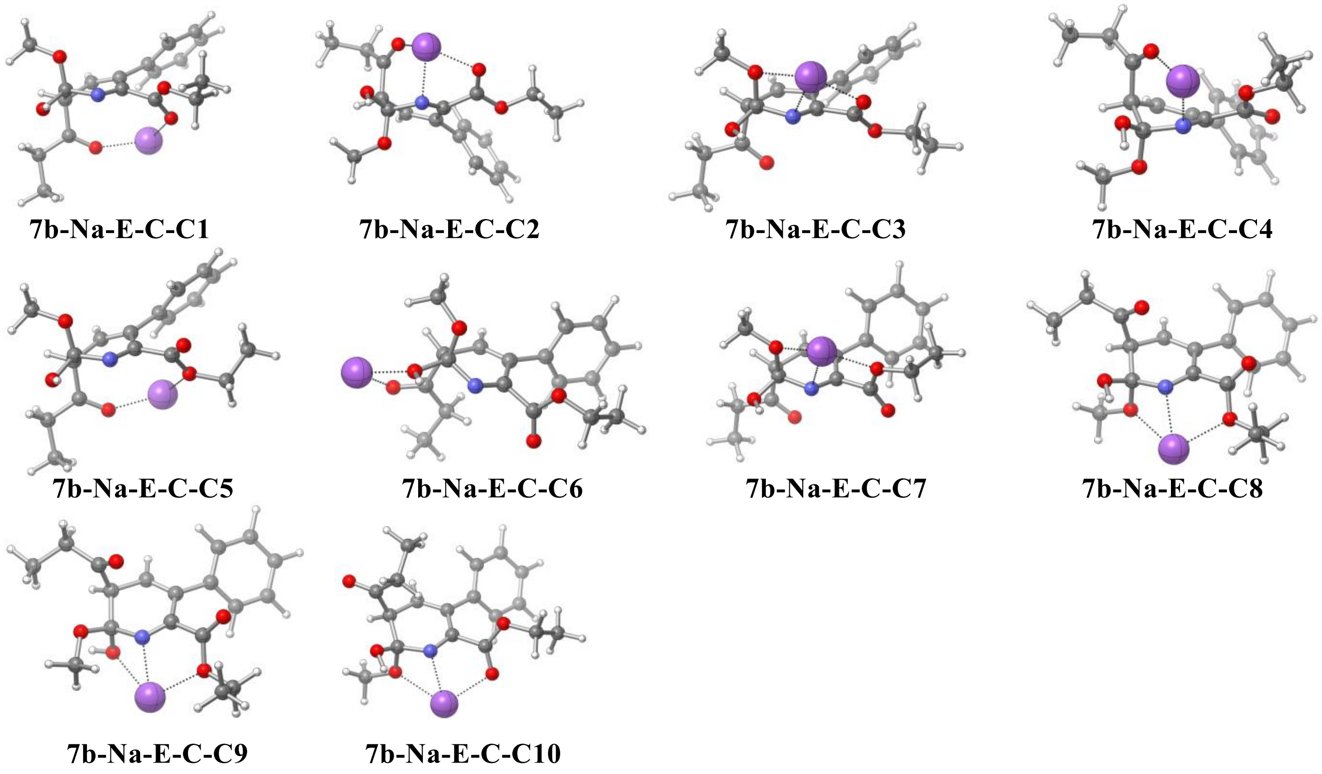

Figure S42. Conformers of sodium-coordinated ester cyclization cis intermediate **7b-Na-E-C**.

Table S41. Energies of conformers of sodium-coordinated ester cyclization cis intermediate **7b-Na-E-C**.[a]

Structure	ΔE	ΔH	ΔG	ΔG (GV50)	ΔΔG
**7b-Na-E-C-C1**	-1293.704417	-1293.310235	-1293.393168	-1293.390573	0.00
**7b-Na-E-C-C2**	-1293.702937	-1293.308389	-1293.391014	-1293.388526	1.28
**7b-Na-E-C-C3**	-1293.699282	-1293.305349	-1293.388848	-1293.386264	2.70
**7b-Na-E-C-C4**	-1293.700302	-1293.305633	-1293.387141	-1293.384931	3.54
**7b-Na-E-C-C5**	-1293.698217	-1293.30441	-1293.387178	-1293.384565	3.77
**7b-Na-E-C-C6**	-1293.691847	-1293.298136	-1293.381742	-1293.378702	7.45

**7b-Na-E-C-C7**	-1293.691442	-1293.297529	-1293.381299	-1293.378389	7.65
**7b-Na-E-C-C8**	-1293.691639	-1293.29746	-1293.380819	-1293.378351	7.67
**7b-Na-E-C-C9**	-1293.696171	-1293.300418	-1293.379064	-1293.377694	8.08
**7b-Na-E-C-C10**	-1293.683815	-1293.289548	-1293.373034	-1293.370215	12.78

[a]Energies were obtained as single points at the PW6B95(D3BJ) / def2-TZVPPD / SMD (DCM) level of theory based on structures optimized at the MN15 / def2-TZVP / SMD (DCM) level of theory. ΔE, ΔH, ΔG, and ΔG (GV50), hartree; ΔΔG, kcal/mol.

7b-Na-K-T

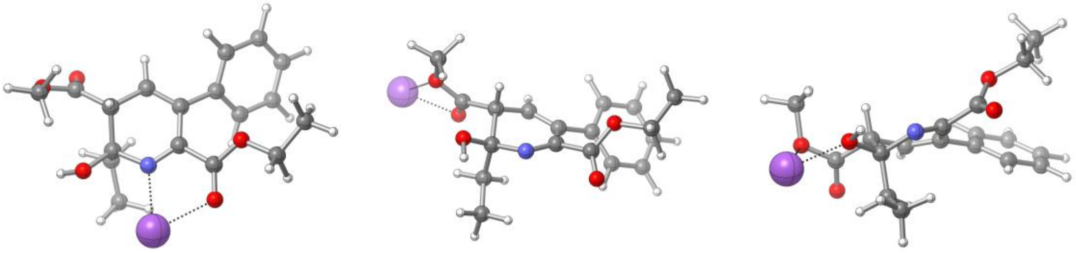

7b-Na-K-T-C1 **7b-Na-K-T-C2** **7b-Na-K-T-C3**

Figure S43. Conformers of sodium-coordinated ketone cyclization trans intermediate **7b-Na-K-T**.

Table S42. Energies of conformers sodium-coordinated ketone cyclization trans intermediate **7b-Na-K-T**.[a]

Structure	ΔE	ΔH	ΔG	ΔG (GV50)	ΔΔG
**7b-Na-K-T-C1**	-1293.710722	-1293.315948	-1293.399878	-1293.396803	0.00
**7b-Na-K-T-C2**	-1293.700964	-1293.306366	-1293.390166	-1293.387645	5.75
**7b-Na-K-T-C3**	-1293.698312	-1293.303819	-1293.387019	-1293.384412	7.78

[a]Energies were obtained as single points at the PW6B95(D3BJ) / def2-TZVPPD / SMD (DCM) level of theory based on structures optimized at the MN15 / def2-TZVP / SMD (DCM) level of theory. ΔE, ΔH, ΔG, and ΔG (GV50), hartree; ΔΔG, kcal/mol.

7b-Na-K-C

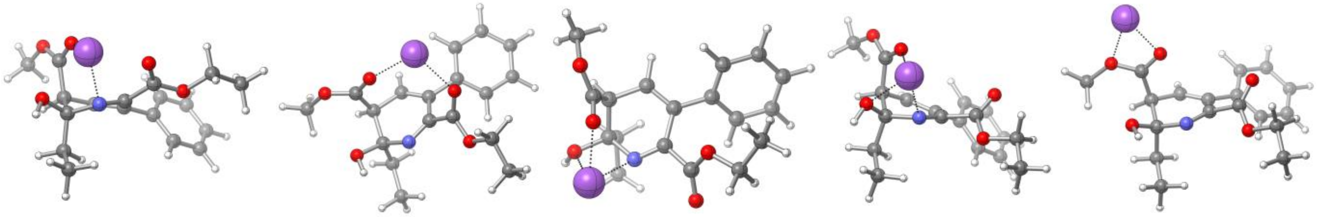

7b-Na-K-C-C1 **7b-Na-K-C-C2** **7b-Na-K-C-C3** **7b-Na-K-C-C4** **7b-Na-K-C-C5**

Figure S44. Conformers of sodium-coordinated ketone cyclization cis intermediate **7b-Na-K-C**.

Table S43. Energies of conformers sodium-coordinated ketone cyclization cis intermediate **7b-Na-K-C**.[a]

Structure	ΔE	ΔH	ΔG	ΔG (GV50)	ΔΔG
**7b-Na-K-C-C1**	-1293.719304	-1293.324572	-1293.408179	-1293.405272	0.00
**7b-Na-K-C-C2**	-1293.717835	-1293.322756	-1293.404913	-1293.402775	1.57
**7b-Na-K-C-C3**	-1293.717533	-1293.322484	-1293.40504	-1293.402618	1.67
**7b-Na-K-C-C4**	-1293.717183	-1293.322023	-1293.404056	-1293.401724	2.23
**7b-Na-K-C-C5**	-1293.70273	-1293.307912	-1293.392152	-1293.389087	10.16

[a]Energies were obtained as single points at the PW6B95(D3BJ) / def2-TZVPPD / SMD (DCM) level of theory based on structures optimized at the MN15 / def2-TZVP / SMD (DCM) level of theory. ΔE, ΔH, ΔG, and ΔG (GV50), hartree; ΔΔG, kcal/mol.

8-E

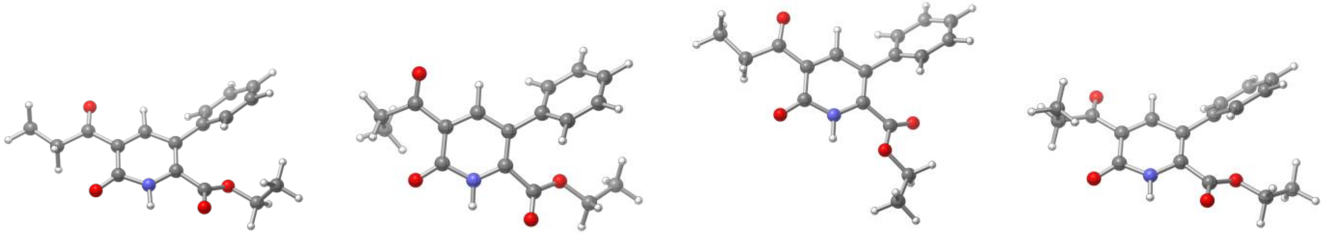

8-E-C1 **8-E-C2** **8-E-C3** **8-E-C4**

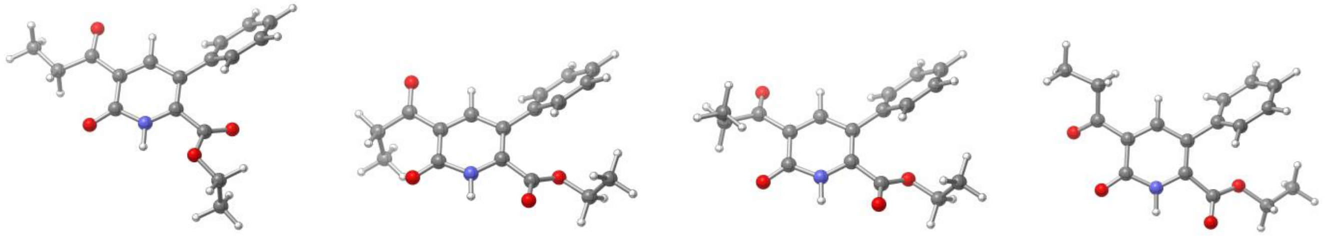

8-E-C5 **8-E-C6** **8-E-C7** **8-E-C8**

Figure S45. Conformers of pyridone product **8-E**.

Table S44. Energies of conformers of pyridone product **8-E**.[a]

Structure	Δ*E*	Δ*H*	Δ*G*	Δ*G* (GV50)	ΔΔ*G*
**8-E-C1**	-1015.439527	-1015.106237	-1015.178984	-1015.176366	0.00
**8-E-C2**	-1015.438641	-1015.105285	-1015.178905	-1015.175911	0.29
**8-E-C3**	-1015.438417	-1015.105192	-1015.178854	-1015.175872	0.31
**8-E-C4**	-1015.438703	-1015.10526	-1015.17859	-1015.175789	0.36
**8-E-C5**	-1015.437812	-1015.104461	-1015.177084	-1015.174549	1.14
**8-E-C6**	-1015.437904	-1015.104371	-1015.176596	-1015.174302	1.30
**8-E-C7**	-1015.437945	-1015.104363	-1015.17649	-1015.174217	1.35
**8-E-C8**	-1015.435252	-1015.101952	-1015.175419	-1015.172856	2.20

[a] Energies were obtained as single points at the PW6B95(D3BJ) / def2-TZVPPD / SMD (DCM) level of theory based on structures optimized at the MN15 / def2-TZVP / SMD (DCM) level of theory. ΔE, ΔH, ΔG, and ΔG (GV50), hartree; ΔΔG, kcal/mol.

8-K

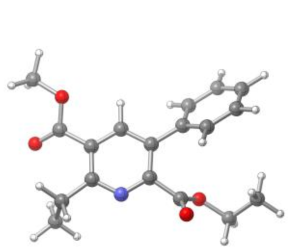

8-K-C1

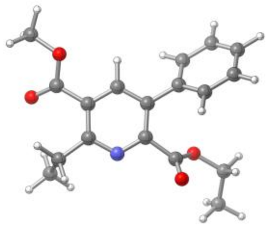

8-K-C2

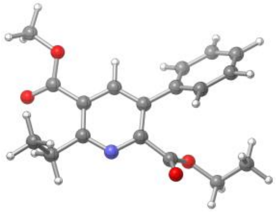

8-K-C3

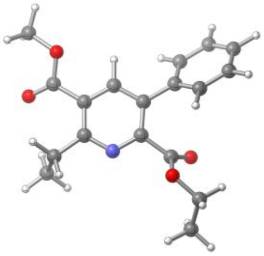

8-K-C4

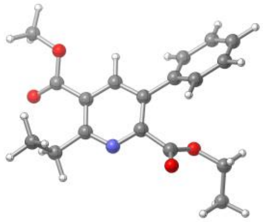

8-K-C5

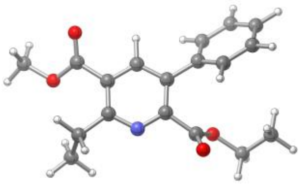

8-K-C6

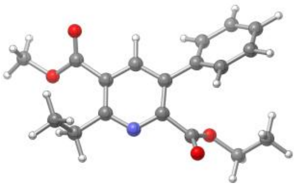

8-K-C7

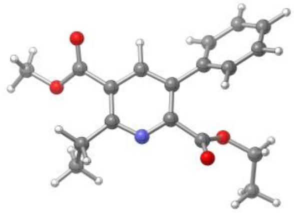

8-K-C8

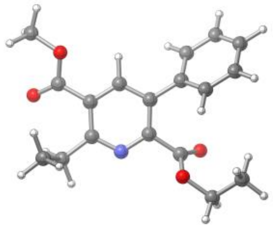

8-K-C9

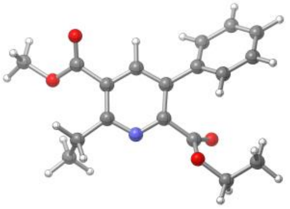

8-K-C10

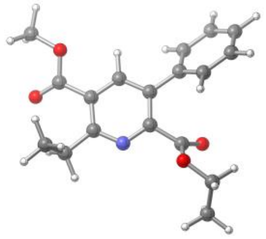

8-K-C11

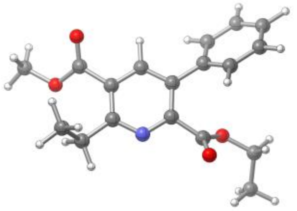

8-K-C12

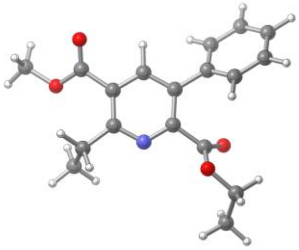

8-K-C13

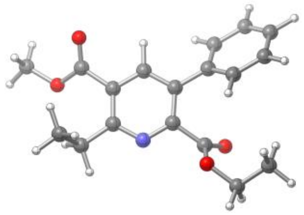

8-K-C14

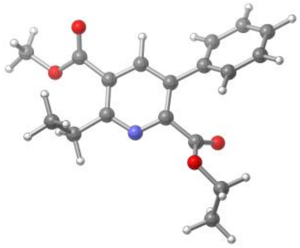

8-K-C15

Figure S46. Conformers of pyridine product **8-K**.

Table S45. Energies of conformers of pyridine product **8-K**.[a]

Structure	Δ*E*	Δ*H*	Δ*G*	Δ*G* (GV50)	ΔΔ*G*
**8-K-C1**	-1054.809355	-1054.445911	-1054.521945	-1054.519291	0.00
**8-K-C2**	-1054.808921	-1054.445435	-1054.521905	-1054.519148	0.09
**8-K-C3**	-1054.808898	-1054.445482	-1054.522044	-1054.518983	0.19
**8-K-C4**	-1054.808631	-1054.445091	-1054.521331	-1054.518709	0.37
**8-K-C5**	-1054.808457	-1054.444926	-1054.521898	-1054.518679	0.38

**8-K-C6**	-1054.808693	-1054.445217	-1054.521092	-1054.518479	0.51
**8-K-C7**	-1054.808474	-1054.445058	-1054.520796	-1054.518423	0.54
**8-K-C8**	-1054.808207	-1054.44468	-1054.521064	-1054.518371	0.58
**8-K-C9**	-1054.808431	-1054.444952	-1054.520964	-1054.51833	0.60
**8-K-C10**	-1054.808195	-1054.44473	-1054.520783	-1054.518274	0.64
**8-K-C11**	-1054.808143	-1054.444595	-1054.521143	-1054.518243	0.66
**8-K-C12**	-1054.80804	-1054.444531	-1054.520725	-1054.51817	0.70
**8-K-C13**	-1054.807908	-1054.444377	-1054.521045	-1054.518153	0.71
**8-K-C14**	-1054.807987	-1054.444587	-1054.520528	-1054.518118	0.74
**8-K-C15**	-1054.807679	-1054.444196	-1054.520725	-1054.51797	0.83

[a] Energies were obtained as single points at the PW6B95(D3BJ) / def2-TZVPPD / SMD (DCM) level of theory based on structures optimized at the MN15 / def2-TZVP / SMD (DCM) level of theory. ΔE, ΔH, ΔG, and ΔG (GV50), hartree; ΔΔG, kcal/mol.

Table S46. Energies of other structures.[a]

**Structure**	**Δ*E***	**Δ*H***	**Δ*G***	ΔG (GV50)
N_2O	-184.9544308	-184.9393862	-184.9642602	-184.9642602
H_2O	-76.55366451	-76.52870578	-76.55079278	-76.55079278
MeOH	-115.9109426	-115.8554341	-115.8826501	-115.8826511
Cs^+	-20.08973125	-20.08737107	-20.10664507	-20.10664507
Na^+	-162.3547732	-162.3524127	-162.3692017	-162.3692017

[a] Energies were obtained as single points at the PW6B95(D3BJ) / def2-TZVPPD / SMD (DCM) level of theory based on structures optimized at the MN15 / def2-TZVP / SMD (DCM) level of theory. ΔE, ΔH, ΔG, and ΔG (GV50), hartree; ΔΔG, kcal/mol.

Distortion/Interaction-Activation Strain Fragments and Electronic Energy by Structure – MN15 / def2-TZVP / SMD (DCM)

6b Fragment F1

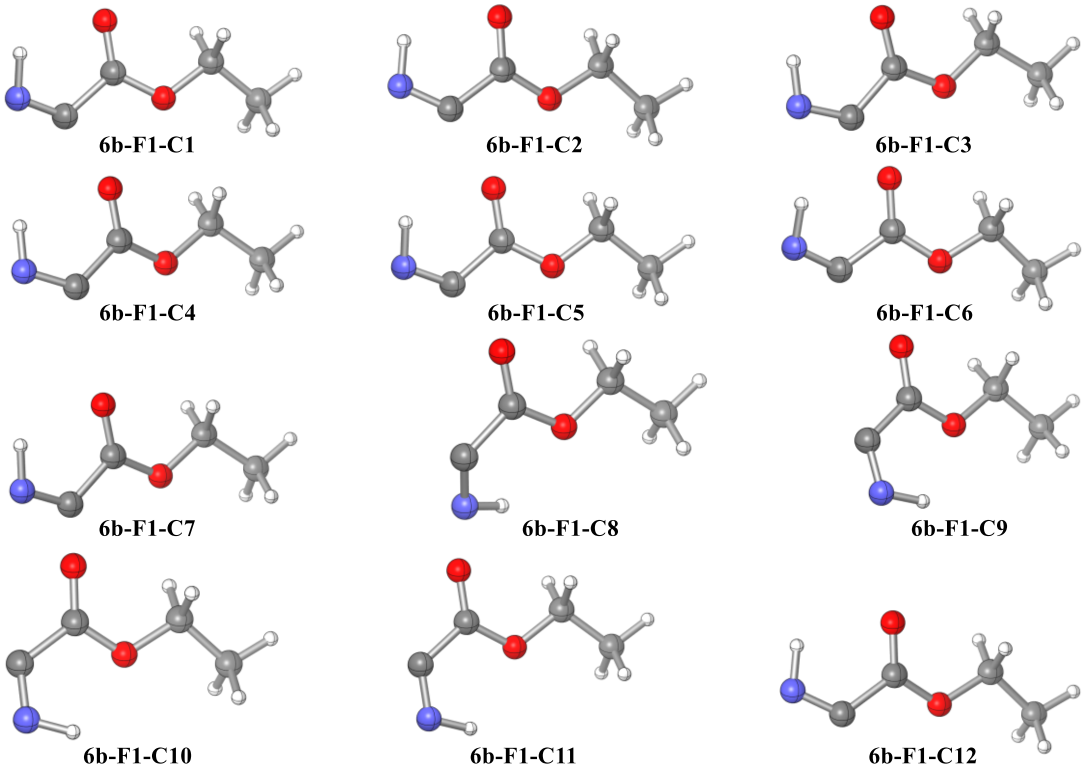

Figure S47. Conformers of fragment **F1** for intermediate **6b**.

Table S47. Energies of conformers of fragment **F1** for intermediate **6b**. [a]

Structure	Δ*E*
**6b-C3**	-360.890759
**6b-C2**	-360.8901045
**6b-C12**	-360.8900083
**6b-C7**	-360.8898753
**6b-C5**	-360.8895681
**6b-C1**	-360.8895596
**6b-C6**	-360.8892895
**6b-C4**	-360.8892737
**6b-C11**	-360.8886553

**6b-C9**	-360.888134
**6b-C8**	-360.8879095
**6b-C10**	-360.8878114

[a]Energies were obtained as single points at the MN15 / def2-TZVP / SMD (DCM) level of theory based on structures optimized at the same level of theory. ΔE, hartree.

6b Fragment F2

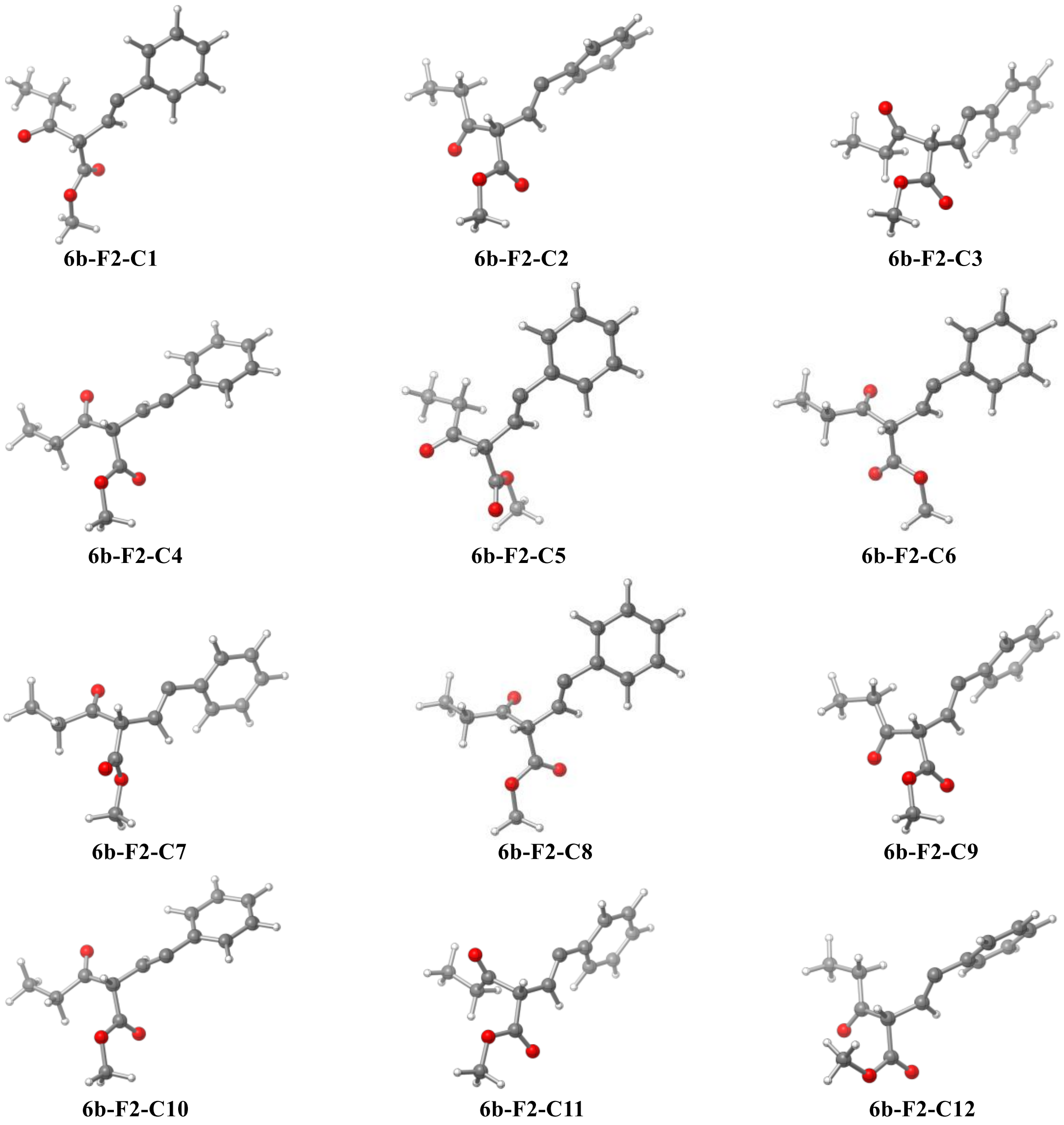

Figure S48. Conformers of fragment **F2** for intermediate **6b**.

Table S48. Energies of conformers of fragment **F2** for intermediate **6b**.[a]

Structure	Δ*E*
**6b-C2**	-767.5050699
**6b-C9**	-767.5047208
**6b-C11**	-767.5040672
**6b-C3**	-767.5038929
**6b-C8**	-767.5023681
**6b-C4**	-767.5023488
**6b-C10**	-767.5023311
**6b-C1**	-767.5021559
**6b-C6**	-767.501702
**6b-C7**	-767.501279
**6b-C5**	-767.5010874
**6b-C12**	-767.4973661

[a]Energies were obtained as single points at the MN15 / def2-TZVP / SMD (DCM) level of theory based on structures optimized at the same level of theory. ΔE, hartree.

6b-Cs Fragment F1

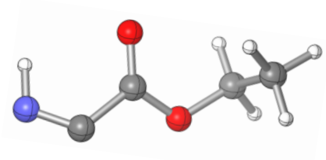

6b-Cs-F1-C1

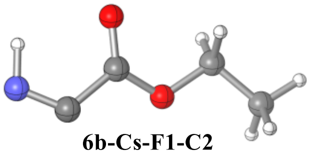

6b-Cs-F1-C2

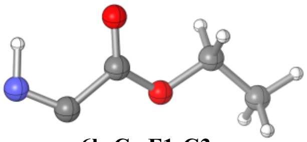

6b-Cs-F1-C3

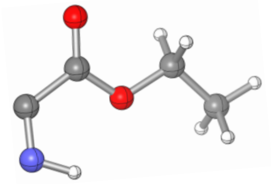

6b-Cs-F1-C4

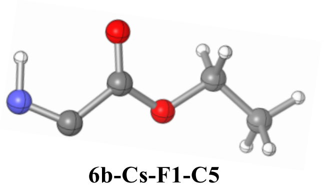

6b-Cs-F1-C5

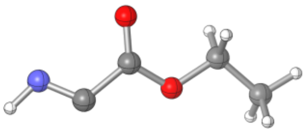

6b-Cs-F1-C6

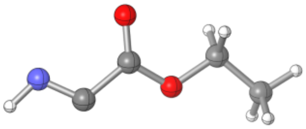

6b-Cs-F1-C7

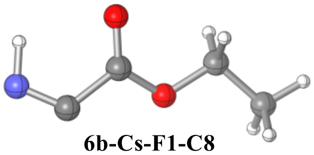

6b-Cs-F1-C8

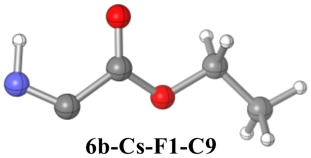

6b-Cs-F1-C9

Figure S49. Conformers of fragment **F1** for intermediate **6b-Cs**.

Table S49. Energies of conformers of fragment **F1** for intermediate **6b-Cs**. [a]

Structure	Δ*E*
**6b-Cs-C6**	-360.8949175
**6b-Cs-C7**	-360.8943334
**6b-Cs-C1**	-360.88957
**6b-Cs-C5**	-360.8894261
**6b-Cs-C9**	-360.8893931
**6b-Cs-C8**	-360.8892505
**6b-Cs-C3**	-360.8892505
**6b-Cs-C2**	-360.888974
**6b-Cs-C4**	-360.8882698

[a]Energies were obtained as single points at the MN15 / def2-TZVP / SMD (DCM) level of theory based on structures optimized at the same level of theory. ΔE, hartree.

6b-Cs Fragment F2

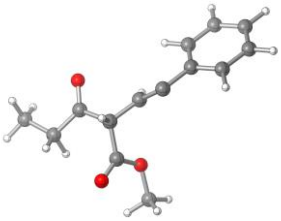

6b-Cs-F2-C1

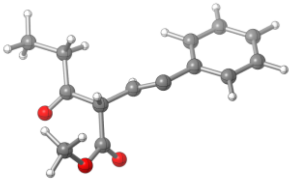

6b-Cs-F2-C2

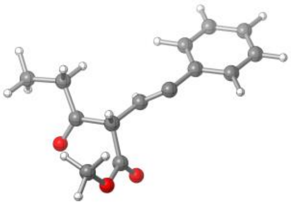

6b-Cs-F2-C3

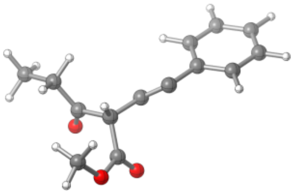

6b-Cs-F2-C5

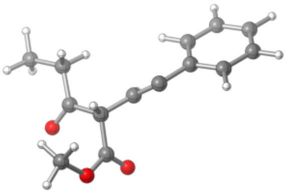

6b-Cs-F2-C4

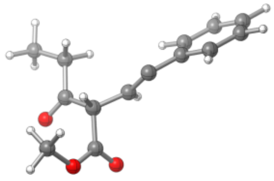

6b-Cs-F2-C6

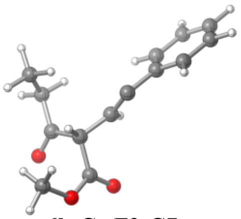

6b-Cs-F2-C7

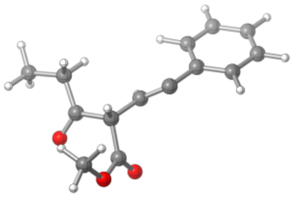

6b-Cs-F2-C8

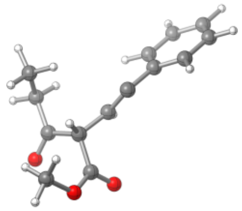

6b-Cs-F2-C9

Figure S50. Conformers of fragment **F2** for intermediate **6b-Cs**.

Table S50. Energies of conformers of fragment **F2** for intermediate **6b-Cs**.[a]

Structure	Δ*E*
**6b-Cs-C1**	-767.5017852
**6b-Cs-C6**	-767.4974734
**6b-Cs-C7**	-767.495785
**6b-Cs-C4**	-767.495432
**6b-Cs-C9**	-767.4949094
**6b-Cs-C2**	-767.4943543
**6b-Cs-C8**	-767.4935853
**6b-Cs-C3**	-767.4935853
**6b-Cs-C5**	-767.4919119

[a]Energies were obtained as single points at the MN15 / def2-TZVP / SMD (DCM) level of theory based on structures optimized at the same level of theory. ΔE, hartree.

6b-Na Fragment F1

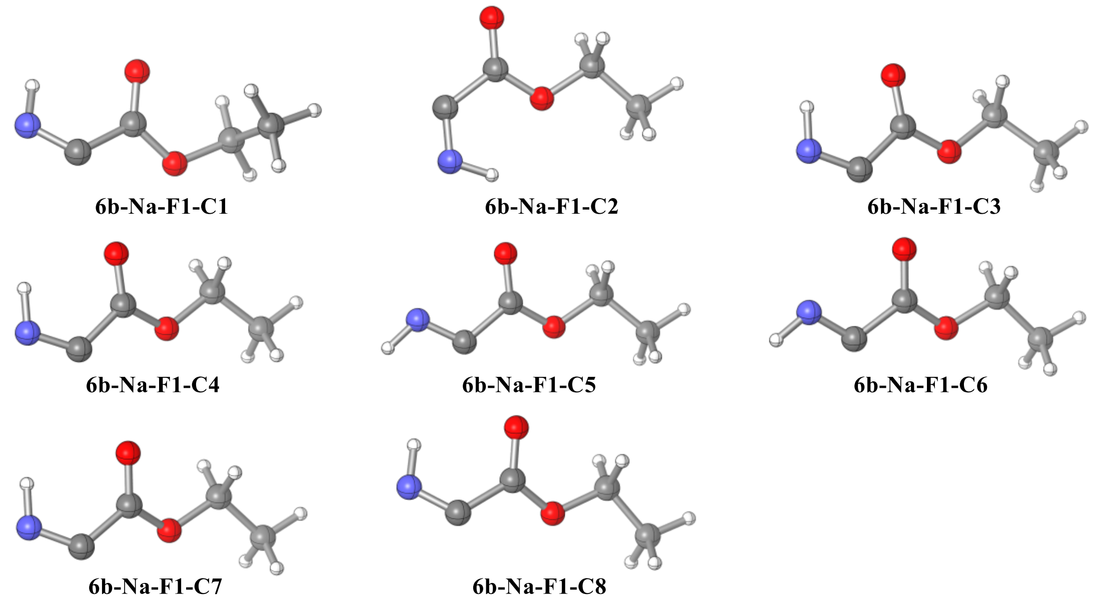

Figure S51. Conformers of fragment **F1** for intermediate **6b-Na**.

Table S51. Energies of conformers of fragment **F1** for intermediate **6b-Na**. [a]

Structure	Δ*E*
6b-Na-C5	-360.8931161
6b-Na-C6	-360.8929365
6b-Na-C4	-360.8898244
6b-Na-C8	-360.889313
6b-Na-C1	-360.8891442
6b-Na-C7	-360.8887943
6b-Na-C2	-360.8885236
6b-Na-C3	-360.8883351

[a]Energies were obtained as single points at the MN15 / def2-TZVP / SMD (DCM) level of theory based on structures optimized at the same level of theory. ΔE, hartree.

6b-Na Fragment F2

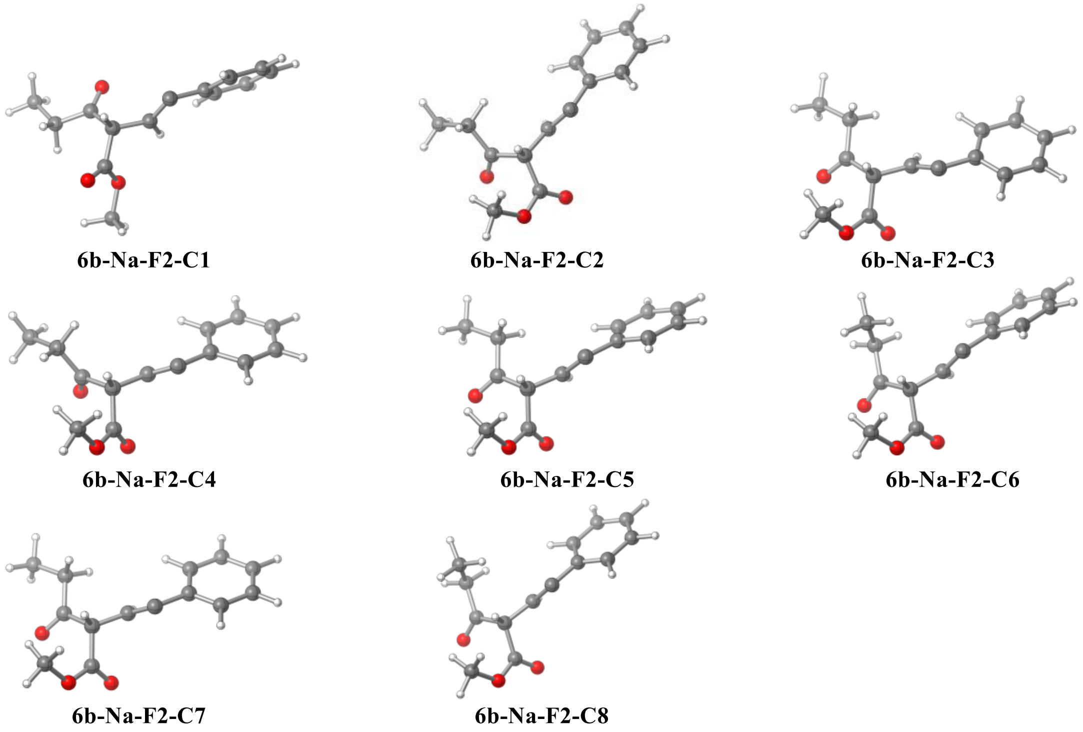

Figure S52. Conformers of fragment **F2** for intermediate **6b-Na**.

Table S52. Energies of conformers fragment **F2** for intermediate **6b-Na**. [a]

Structure	Δ*E*
**6b-Na-C1**	-767.5020854
**6b-Na-C5**	-767.4974223
**6b-Na-C6**	-767.4959084
**6b-Na-C2**	-767.4951958
**6b-Na-C8**	-767.4948013
**6b-Na-C7**	-767.4941417
**6b-Na-C3**	-767.4929534
**6b-Na-C4**	-767.4896099

[a]Energies were obtained as single points at the MN15 / def2-TZVP / SMD (DCM) level of theory based on structures optimized at the same level of theory. ΔE, hartree.

TSB-K-T Fragment F1

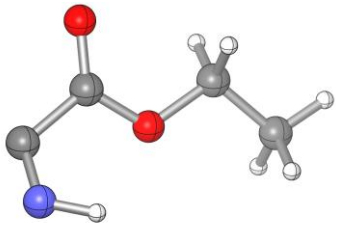

TSB-K-T-F1-C1

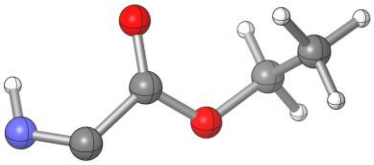

TSB-K-T-F1-C2

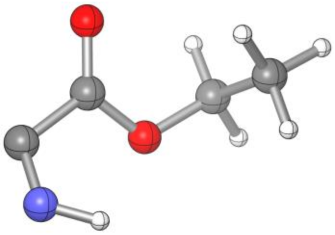

TSB-K-T-F1-C3

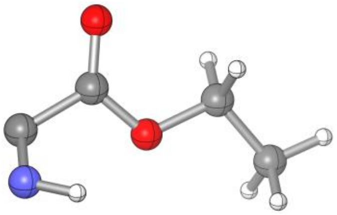

TSB-K-T-F1-C4

Figure S53. Conformers of fragment **F1** for transition state **TSB-K-T**.

Table S53. Energies of conformers of fragment **F1** for transition state **TSB-K-T**. [a]

Structure	Δ*E*
**TSB-K-T-C2**	-360.8870855
**TSB-K-T-C1**	-360.8853292

**TSB-K-T-C4**	-360.8852605
**TSB-K-T-C3**	-360.8850094

[a]Energies were obtained as single points at the MN15 / def2-TZVP / SMD (DCM) level of theory based on structures optimized at the same level of theory. ΔE, hartree.

TSB-K-T Fragment F2

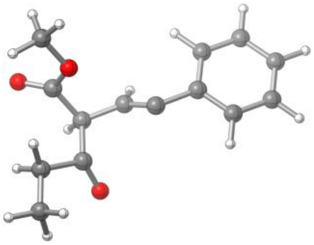

TSB-K-T-F2-C1

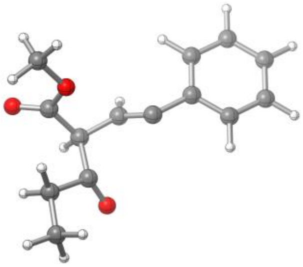

TSB-K-T-F2-C2

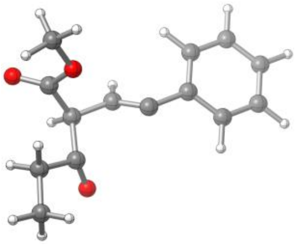

TSB-K-T-F2-C3

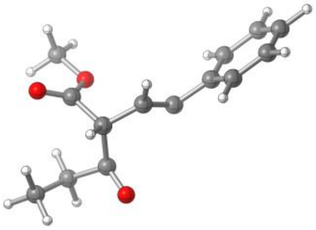

TSB-K-T-F2-C4

Figure S54. Conformers of fragment **F2** for transition state **TSB-K-T**.

Table S54. Energies of conformers of fragment **F2** for transition state **TSB-K-T**.[a]

Structure	Δ*E*
**TSB-K-T-C4**	-767.4693553
**TSB-K-T-C3**	-767.4692234
**TSB-K-T-C1**	-767.4690992
**TSB-K-T-C2**	-767.468291

[a]Energies were obtained as single points at the MN15 / def2-TZVP / SMD (DCM) level of theory based on structures optimized at the same level of theory. ΔE, hartree.

TSB-K-C Fragment F1

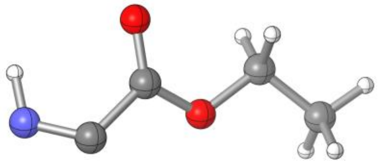

TSB-K-C-F1-C1

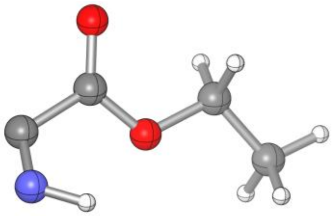

TSB-K-C-F1-C2

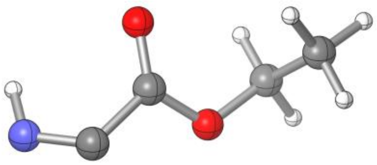

TSB-K-C-F1-C3

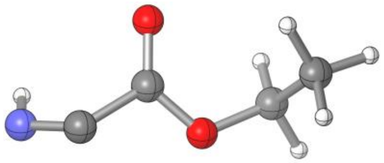

TSB-K-C-F1-C4

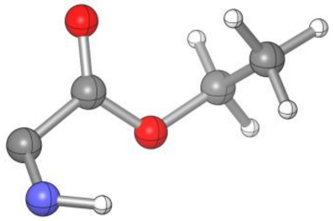

TSB-K-C-F1-C5

Figure S55. Conformers of fragment **F1** for transition state **TSB-K-C**.

Table S55. Energies of conformers of fragment **F1** for transition state **TSB-K-C**. [a]

Structure	Δ*E*
**TSB-K-C-C4**	-360.888161
**TSB-K-C-C3**	-360.8866489
**TSB-K-C-C1**	-360.8861748
**TSB-K-C-C5**	-360.8858119
**TSB-K-C-C2**	-360.8849229

[a]Energies were obtained as single points at the MN15 / def2-TZVP / SMD (DCM) level of theory based on structures optimized at the same level of theory. ΔE, hartree.

TSB-K-C Fragment F2

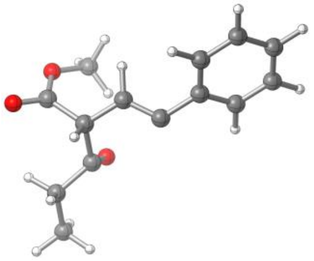

TSB-K-C-F2-C1

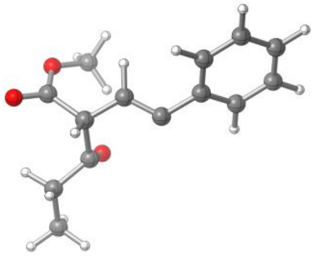

TSB-K-C-F2-C2

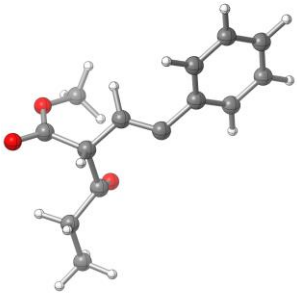

TSB-K-C-F2-C3

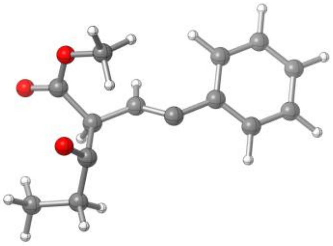

TSB-K-C-F2-C4

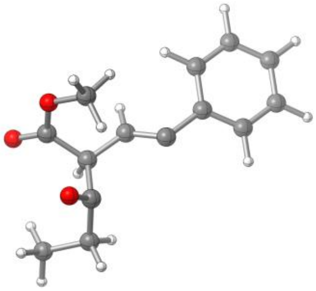

TSB-K-C-F2-C5

Figure S56. Conformers of fragment **F2** for transition state **TSB-K-C**.

Table S56. Energies of conformers of fragment **F2** for transition state **TSB-K-C**.[a]

Structure	Δ*E*
**TSB-K-C-C5**	-767.4609139
**TSB-K-C-C4**	-767.46032
**TSB-K-C-C1**	-767.4594254
**TSB-K-C-C3**	-767.4592898
**TSB-K-C-C2**	-767.4588573

[a]Energies were obtained as single points at the MN15 / def2-TZVP / SMD (DCM) level of theory based on structures optimized at the same level of theory. ΔE, hartree.

TSB-E-T Fragment F1

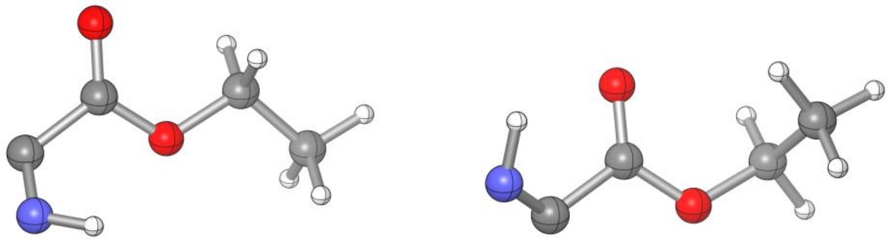

TSB-E-T-F1-C1 **TSB-E-T-F1-C2**

Figure S57. Conformers of fragment **F1** for transition state **TSB-E-T**.

Table S57. Energies of conformers of fragment **F1** for transition state **TSB-E-T**. [a]

Structure	Δ*E*
**TSB-E-T-C1**	-360.8866259
**TSB-E-T-C2**	-360.8863391

[a]Energies were obtained as single points at the MN15 / def2-TZVP / SMD (DCM) level of theory based on structures optimized at the same level of theory. ΔE, hartree.

TSB-E-T Fragment F2

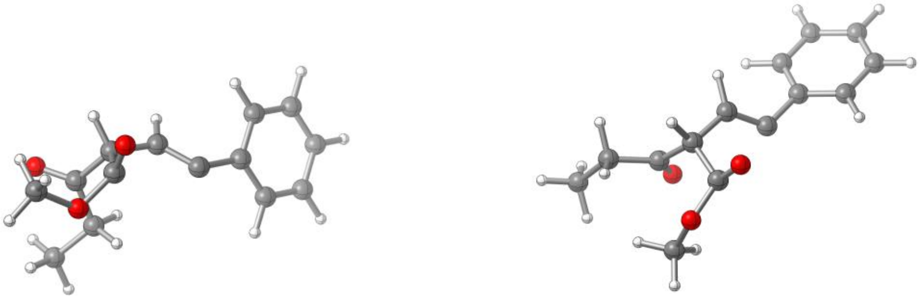

TSB-E-T-F2-C1 **TSB-E-T-F2-C2**

Figure S58. Conformers of fragment **F2** for transition state **TSB-E-T**.

Table S58. Energies of conformers of fragment **F2** for transition state **TSB-E-T**. [a]

Structure	Δ*E*
**TSB-E-T-C1**	-767.4618329
**TSB-E-T-C2**	-767.4522743

[a]Energies were obtained as single points at the MN15 / def2-TZVP / SMD (DCM) level of theory based on structures optimized at the same level of theory. ΔE, hartree.

TSB-E-C Fragment F1

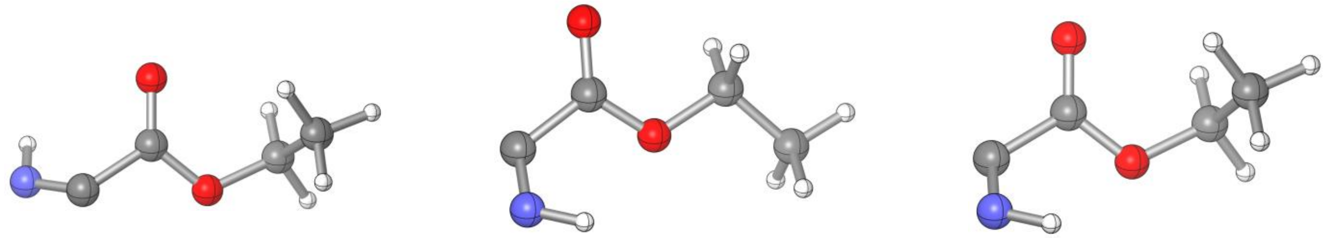

TSB-E-C-F1-C1 **TSB-E-C-F1-C2** **TSB-E-C-F1-C3**

Figure S59. Conformers of fragment **F1** for transition state **TSB-E-C**.

Table S59. Energies of conformers of fragment **F1** for transition state **TSB-E-C**. [a]

Structure	Δ*E*
**TSB-E-C-C1**	-360.8894664
**TSB-E-C-C2**	-360.8876299
**TSB-E-C-C3**	-360.8873482

[a]Energies were obtained as single points at the MN15 / def2-TZVP / SMD (DCM) level of theory based on structures optimized at the same level of theory. ΔE, hartree.

TSB-E-C Fragment F2

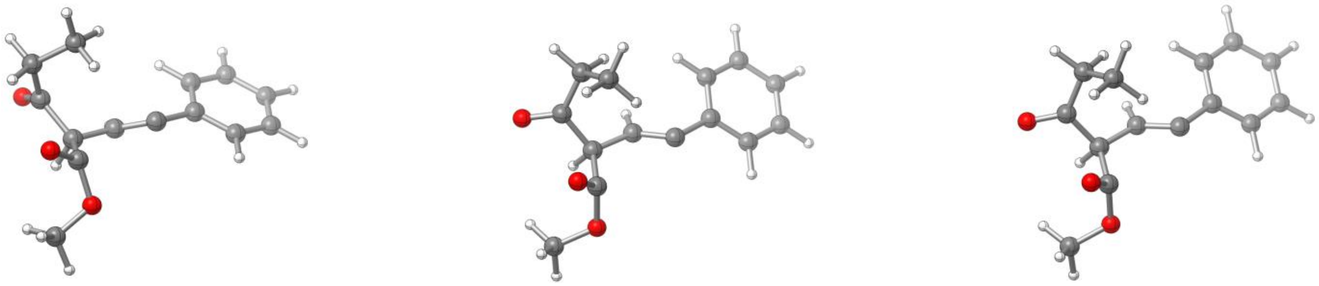

TSB-E-C-F2-C1 **TSB-E-C-F2-C2** **TSB-E-F2-C3**

Figure S60. Conformers of fragment **F2** for transition state **TSB-E-C**.

Table S60. Energies of conformers of fragment **F2** for transition state **TSB-E-C**. [a]

Structure	Δ*E*
**TSB-E-C-C3**	-767.457268
**TSB-E-C-C2**	-767.4572525
**TSB-E-C-C1**	-767.4569468

[a]Energies were obtained as single points at the MN15 / def2-TZVP / SMD (DCM) level of theory based on structures optimized at the same level of theory. ΔE, hartree.

TSB-Cs-K-T Fragment F1

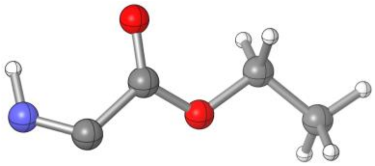

TSB-Cs-K-T-F1-C1

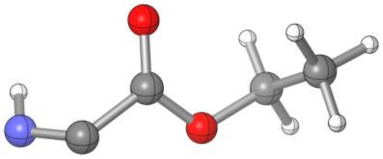

TSB-Cs-K-T-F1-C2

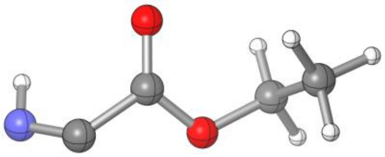

TSB-Cs-K-T-F1-C3

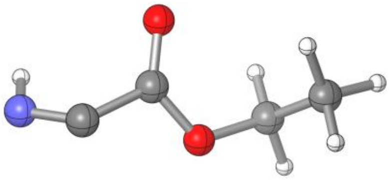

TSB-Cs-K-T-F1-C4

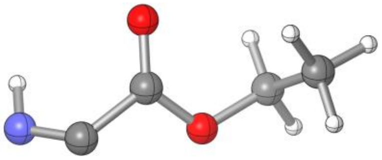

TSB-Cs-K-T-F1-C5

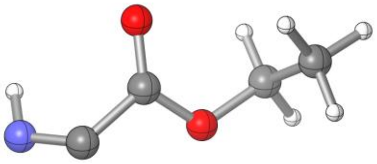

TSB-Cs-K-T-F1-C6

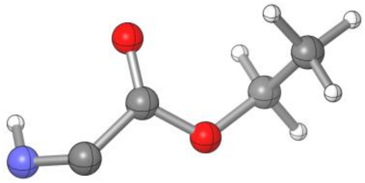

TSB-Cs-K-T-F1-C7

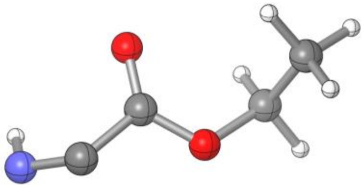

TSB-Cs-K-T-F1-C8

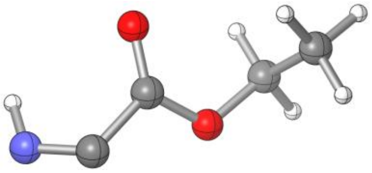

TSB-Cs-K-T-F1-C9

Figure S61. Conformers of fragment **F1** for transition state **TSB-Cs-K-T**.

Table S61. Energies of conformers of fragment **F1** for transition state **TSB-Cs-K-T**.[a]

Structure	Δ*E*
**TSB-Cs-K-T-C1**	-360.8890472
**TSB-Cs-K-T-C4**	-360.8884158
**TSB-Cs-K-T-C3**	-360.8882955
**TSB-Cs-K-T-C5**	-360.8876659
**TSB-Cs-K-T-C2**	-360.8876348
**TSB-Cs-K-T-C9**	-360.8875916
**TSB-Cs-K-T-C6**	-360.887549
**TSB-Cs-K-T-C7**	-360.8870643
**TSB-Cs-K-T-C8**	-360.8866972

[a]Energies were obtained as single points at the MN15 / def2-TZVP / SMD (DCM) level of theory based on structures optimized at the same level of theory. ΔE, hartree.

TSB-Cs-K-T Fragment F2

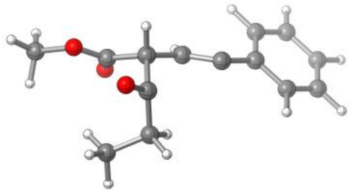

TSB-Cs-K-T-F2-C1

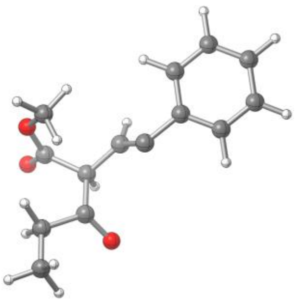

TSB-Cs-K-T-F2-C2

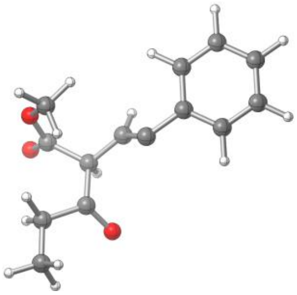

TSB-Cs-K-T-F2-C3

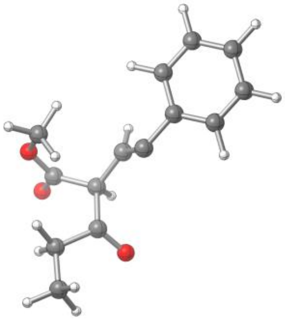

TSB-Cs-K-T-F2-C4

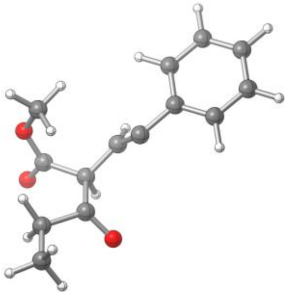

TSB-Cs-K-T-F2-C5

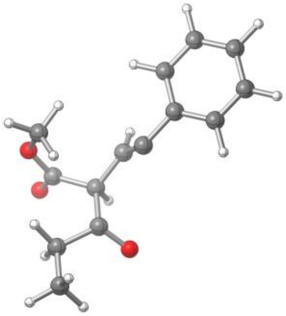

TSB-Cs-K-T-F2-C6

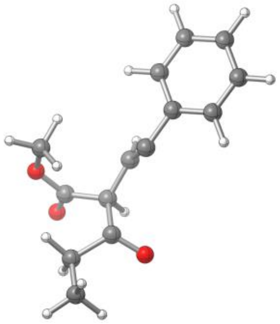

TSB-Cs-K-T-F2-C7

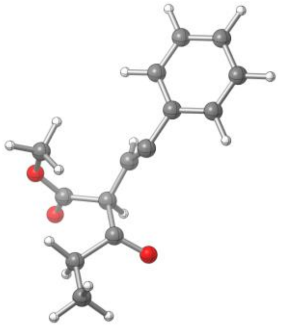

TSB-Cs-K-T-F2-C8

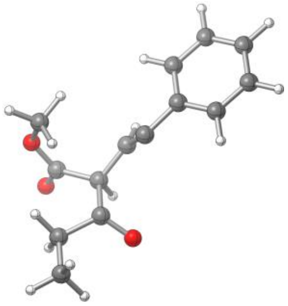

TSB-Cs-K-T-F2-C9

Figure S62. Conformers of fragment **F2** for transition state **TSB-Cs-K-T**.

Table S62. Energies of conformers of fragment **F2** for transition state **TSB-Cs-K-T**.[a]

Structure	Δ*E*
**TSB-Cs-K-T-C1**	-767.4793681
**TSB-Cs-K-T-C3**	-767.4641035
**TSB-Cs-K-T-C2**	-767.4625831
**TSB-Cs-K-T-C4**	-767.4610787
**TSB-Cs-K-T-C5**	-767.459925

**TSB-Cs-K-T-C7**	-767.4593369
**TSB-Cs-K-T-C6**	-767.4590215
**TSB-Cs-K-T-C8**	-767.457707
**TSB-Cs-K-T-C9**	-767.4565832

[a]Energies were obtained as single points at the MN15 / def2-TZVP / SMD (DCM) level of theory based on structures optimized at the same level of theory. ΔE, hartree.

TSB-Cs-K-C Fragment F1

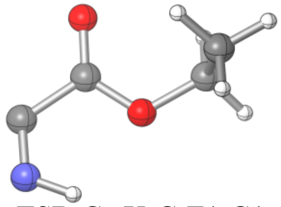
TSB-Cs-K-C-F1-C1

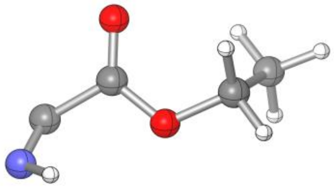
TSB-Cs-K-C-F1-C2

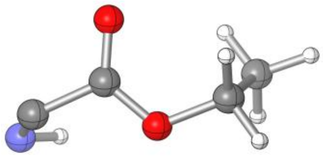
TSB-Cs-K-C-F1-C3

Figure S63. Conformers of fragment **F1** for transition state **TSB-Cs-K-C**.

Table S63. Energies of conformers of fragment **F1** for transition state **TSB-Cs-K-C**.[a]

**Structure**	**Δ*E***
**TSB-Cs-K-C-C2**	-360.8858021
**TSB-Cs-K-C-C1**	-360.8854029
**TSB-Cs-K-C-C3**	-360.8853893

[a]Energies were obtained as single points at the MN15 / def2-TZVP / SMD (DCM) level of theory based on structures optimized at the same level of theory. ΔE, hartree.

TSB-Cs-K-C Fragment F2

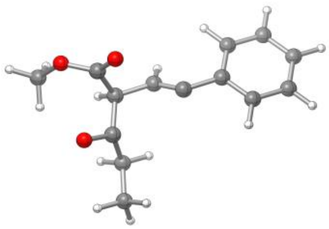
TSB-Cs-K-C-F2-C1

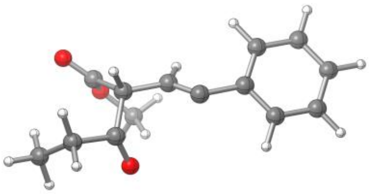
TSB-Cs-K-C-F2-C2

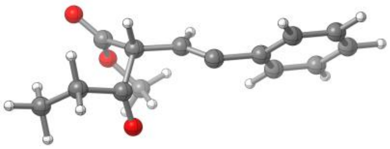
TSB-Cs-K-C-F2-C3

Figure S64. Conformers of fragment **F2** for transition state **TSB-Cs-K-C**.

Table S64. Energies of conformers of fragment **F2** for transition state **TSB-Cs-K-C**.[a]

Structure	Δ*E*
**TSB-Cs-K-C-C1**	-767.4654485
**TSB-Cs-K-C-C2**	-767.4651693
**TSB-Cs-K-C-C3**	-767.4621897

[a]Energies were obtained as single points at the MN15 / def2-TZVP / SMD (DCM) level of theory based on structures optimized at the same level of theory. ΔE, hartree.

TSB-Cs-E-T Fragment F1

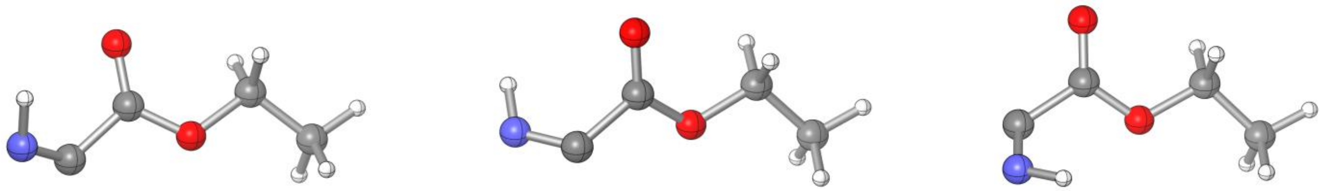

TSB-Cs-E-T-F1-C1 **TSB-Cs-E-T-F1-C2** **TSB-Cs-E-T-F1-C3**

Figure S65. Conformers of fragment **F1** for transition state **TSB-Cs-E-T**.

Table S65. Energies of conformers of fragment **F1** for transition state **TSB-Cs-E-T**.[a]

Structure	Δ*E*
**TSB-Cs-E-T-C2**	-360.8881578
**TSB-Cs-E-T-C1**	-360.8878187
**TSB-Cs-E-T-C3**	-360.8868463

[a]Energies were obtained as single points at the MN15 / def2-TZVP / SMD (DCM) level of theory based on structures optimized at the same level of theory. ΔE, hartree.

TSB-Cs-E-T Fragment F2

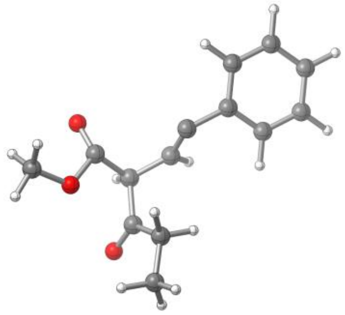

TSB-Cs-E-T-F2-C1

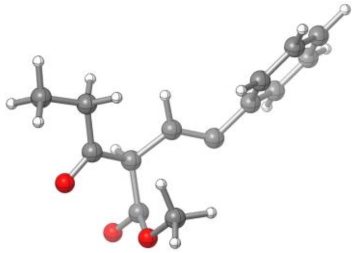

TSB-Cs-E-T-F2-C2

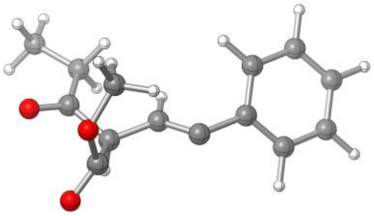

TSB-Cs-E-T-F2-C3

Figure S66. Conformers of fragment **F2** for transition state **TSB-Cs-E-T**.

Table S66. Energies of conformers of fragment **F2** for transition state **TSB-Cs-E-T**.[a]

Structure	Δ*E*
**TSB-Cs-E-T-C1**	-767.4677878
**TSB-Cs-E-T-C3**	-767.4618319
**TSB-Cs-E-T-C2**	-767.4615093

[a]Energies were obtained as single points at the MN15 / def2-TZVP / SMD (DCM) level of theory based on structures optimized at the same level of theory. ΔE, hartree.

TSB-Cs-E-C Fragment F1

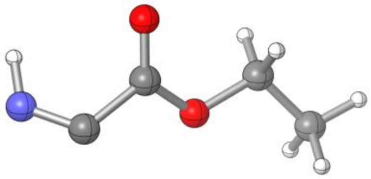

TSB-Cs-E-C-F1-C1

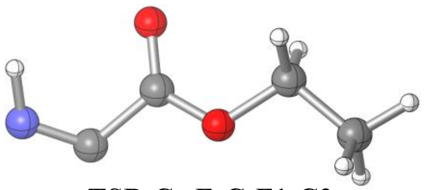

TSB-Cs-E-C-F1-C2

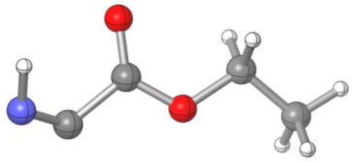

TSB-Cs-E-C-F1-C3

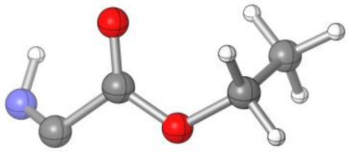

TSB-Cs-E-C-F1-C4

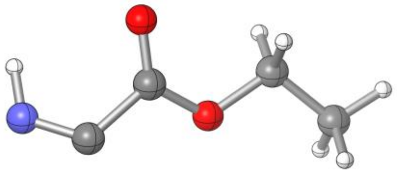

TSB-Cs-E-C-F1-C6

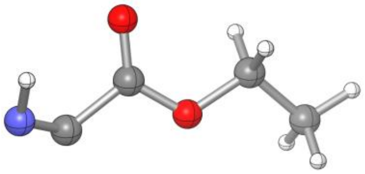

TSB-Cs-E-C-F1-C6

Figure S67. Conformers of fragment **F1** for transition state **TSB-Cs-E-C**.

Table S67. Energies of conformers of fragment **F1** for transition state **TSB-Cs-E-C**.[a]

Structure	Δ*E*
**TSB-Cs-E-C-C6**	-360.8885666
**TSB-Cs-E-C-C1**	-360.8878381
**TSB-Cs-E-C-C4**	-360.8877311
**TSB-Cs-E-C-C5**	-360.8877224
**TSB-Cs-E-C-C2**	-360.8875087

[a]Energies were obtained as single points at the MN15 / def2-TZVP / SMD (DCM) level of theory based on structures optimized at the same level of theory. ΔE, hartree.

TSB-Cs-E-C Fragment F2

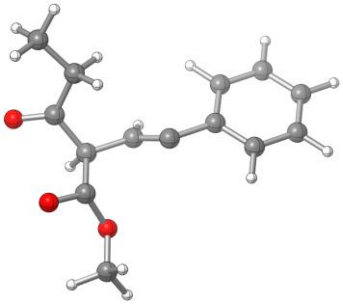

TSB-Cs-E-C-F2-C1

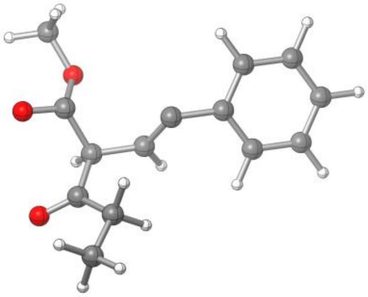

TSB-Cs-E-C-F2-C2

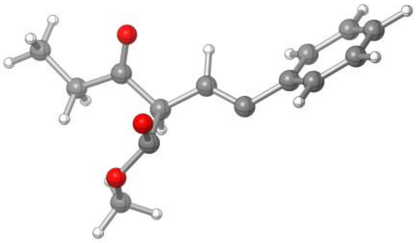

TSB-Cs-E-C-F2-C3

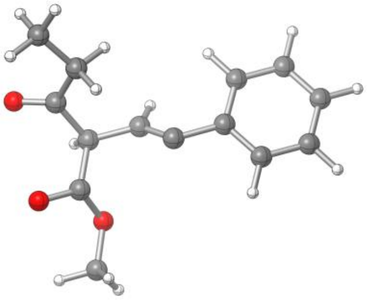

TSB-Cs-E-C-F2-C4

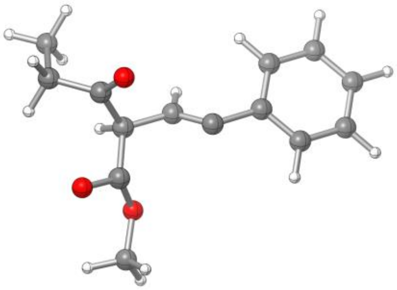

TSB-Cs-E-C-F2-C5

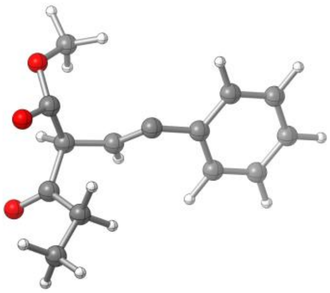

TSB-Cs-E-C-F2-C6

Figure S68. Conformers of fragment **F2** for transition state **TSB-Cs-E-C**.

Table S68. Energies of conformers of fragment **F2** for transition state **TSB-Cs-E-C**.[a]

Structure	Δ*E*
**TSB-Cs-E-C-C6**	-767.466413
**TSB-Cs-E-C-C5**	-767.4637485

**TSB-Cs-E-C-C4**	-767.4635251
**TSB-Cs-E-C-C1**	-767.4628295
**TSB-Cs-E-C-C2**	-767.4626709

[a]Energies were obtained as single points at the MN15 / def2-TZVP / SMD (DCM) level of theory based on structures optimized at the same level of theory. ΔE, hartree.

TSB-Na-K-T Fragment F1

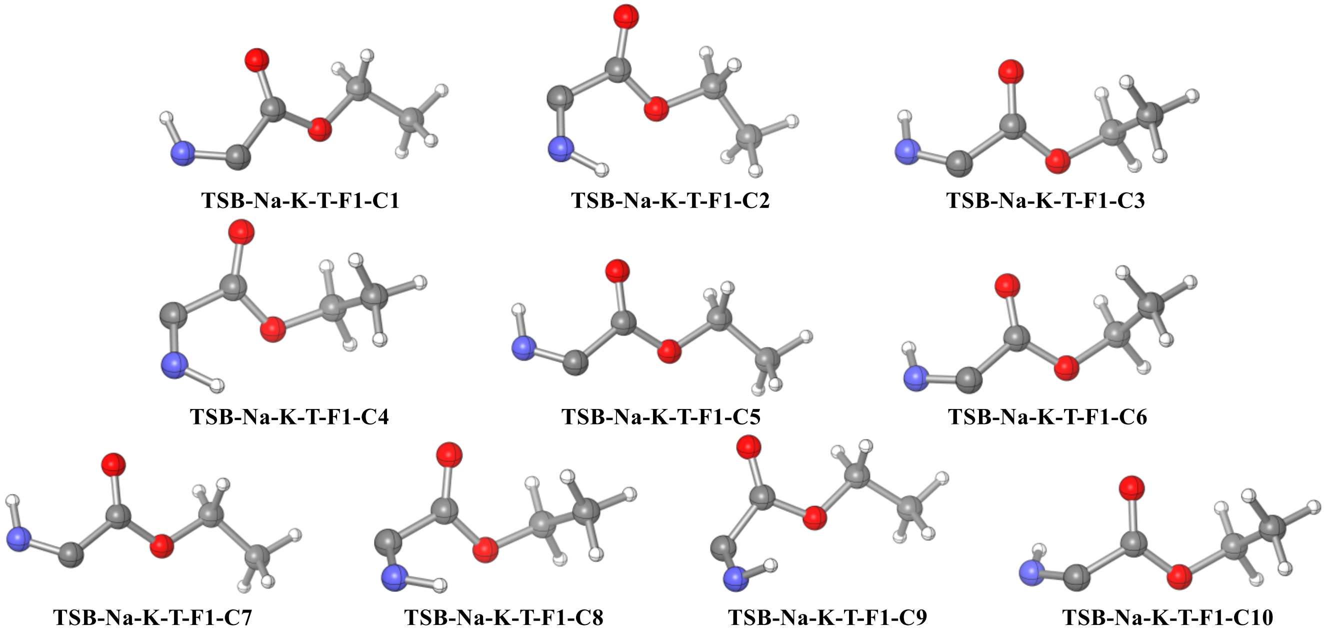

Figure S69. Conformers of fragment **F1** for transition state **TSB-Na-K-T**.

Table S69. Energies of conformers of fragment **F1** for transition state **TSB-Na-K-T**.[a]

Structure	Δ*E*
**TSB-Na-K-T-C3**	-360.8898057
**TSB-Na-K-T-C6**	-360.8897817
**TSB-Na-K-T-C7**	-360.889501
**TSB-Na-K-T-C5**	-360.8892917
**TSB-Na-K-T-C1**	-360.8892682
**TSB-Na-K-T-C2**	-360.8876763
**TSB-Na-K-T-C4**	-360.8874357
**TSB-Na-K-T-C8**	-360.8867019

**TSB-Na-K-T-C9**	-360.8858274
**TSB-Na-K-T-C10**	-360.885262

[a]Energies were obtained as single points at the MN15 / def2-TZVP / SMD (DCM) level of theory based on structures optimized at the same level of theory. ΔE, hartree.

TSB-Na-K-T Fragment F2

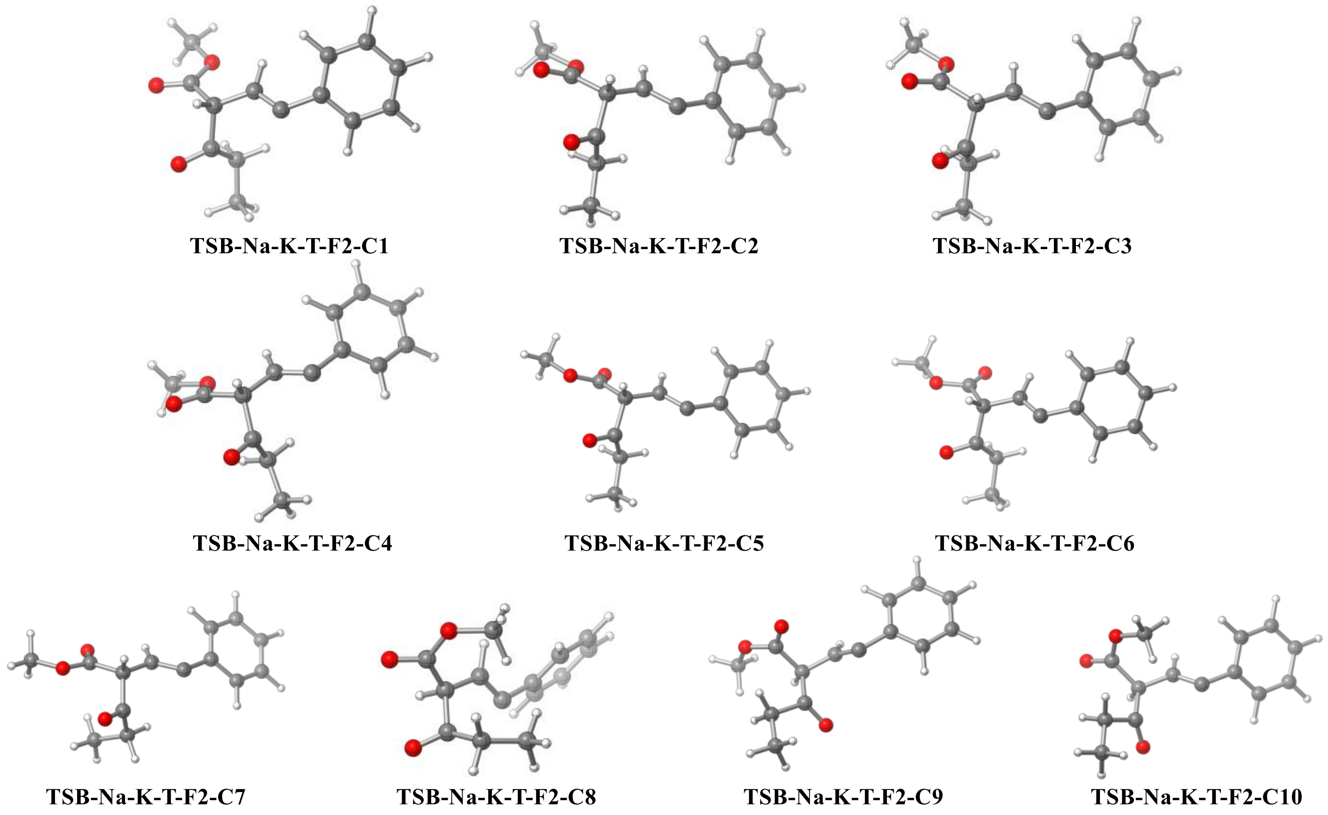

Figure S70. Conformers of fragment **F2** for transition state **TSB-Na-K-T**.

Table S70. Energies of conformers of fragment **F2** for transition state **TSB-Na-K-T**.[a]

Structure	Δ*E*
**TSB-Na-K-T-C7**	-767.483619
**TSB-Na-K-T-C6**	-767.4829992
**TSB-Na-K-T-C5**	-767.4825825
**TSB-Na-K-T-C3**	-767.4807686
**TSB-Na-K-T-C4**	-767.4804792
**TSB-Na-K-T-C2**	-767.4800816

**TSB-Na-K-T-C1**	-767.4800435
**TSB-Na-K-T-C9**	-767.4712748
**TSB-Na-K-T-C10**	-767.4634094
**TSB-Na-K-T-C8**	-767.4605217

[a]Energies were obtained as single points at the MN15 / def2-TZVP / SMD (DCM) level of theory based on structures optimized at the same level of theory. ΔE, hartree.

TSB-Na-K-C Fragment F1

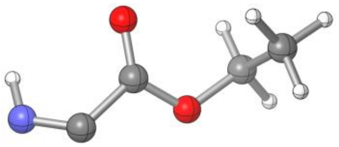

TSB-Na-K-C-F1-C1

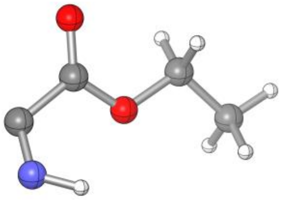

TSB-Na-K-C-F1-C2

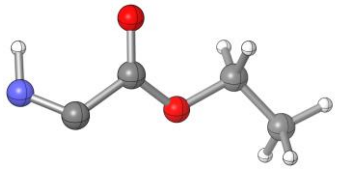

TSB-Na-K-C-F1-C3

Figure S71. Conformers of fragment **F1** for transition state **TSB-Na-K-C**.

Table S71. Energies of conformers of fragment **F1** for transition state **TSB-Na-K-C**.[a]

Structure	Δ*E*
**TSB-Na-K-C-C1**	-360.8893736
**TSB-Na-K-C-C3**	-360.8885053
**TSB-Na-K-C-C2**	-360.8879021

[a]Energies were obtained as single points at the MN15 / def2-TZVP / SMD (DCM) level of theory based on structures optimized at the same level of theory. ΔE, hartree.

TSB-Na-K-C Fragment F2

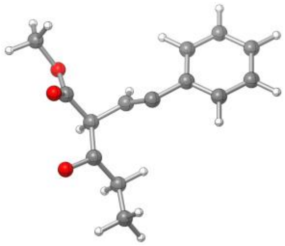

TSB-Na-K-C-F2-C1

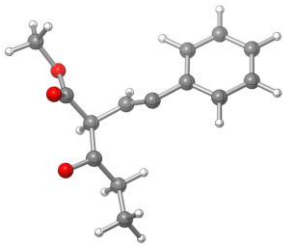

TSB-Na-K-C-F2-C2

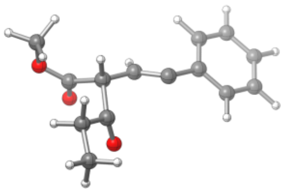

TSB-Na-K-C-F2-C3

Figure S72. Conformers of fragment **F2** for transition state **TSB-Na-K-C**.

Table S72. Energies of conformers of fragment **F2** for transition state **TSB-Na-K-C**.[a]

Structure	Δ*E*
**TSB-Na-K-C-C2**	-767.4757391
**TSB-Na-K-C-C1**	-767.4755163
**TSB-Na-K-C-C3**	-767.4701713

[a]Energies were obtained as single points at the MN15 / def2-TZVP / SMD (DCM) level of theory based on structures optimized at the same level of theory. ΔE, hartree.

TSB-Na-E-T Fragment F1

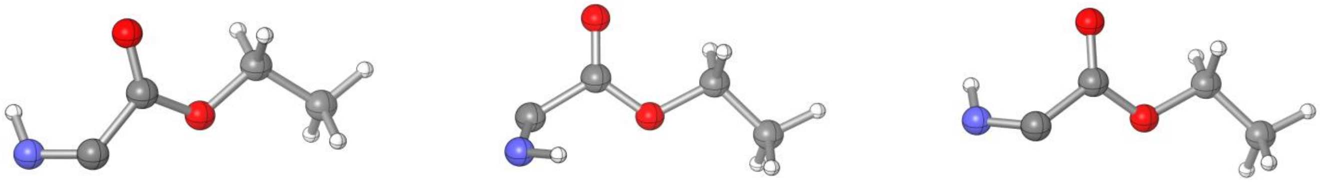

TSB-Na-E-T-F1-C1 **TSB-Na-E-T-F1-C2** **TSB-Na-E-T-F1-C3**

Figure S73. Conformers of fragment **F1** for transition state **TSB-Na-E-T**.

Table S73. Energies of conformers of fragment **F1** for transition state **TSB-Na-E-T**.[a]

Structure	Δ*E*
**TSB-Na-E-T-C1**	-360.8888639
**TSB-Na-E-T-C3**	-360.8886144
**TSB-Na-E-T-C2**	-360.8882133

[a]Energies were obtained as single points at the MN15 / def2-TZVP / SMD (DCM) level of theory based on structures optimized at the same level of theory. ΔE, hartree.

TSB-Na-E-T Fragment F2

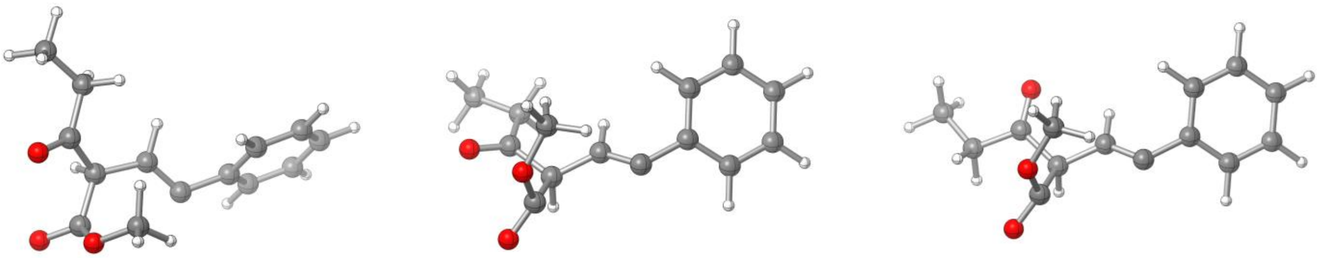

TSB-Na-E-T-F2-C1 **TSB-Na-E-T-F2-C2** **TSB-Na-E-T-F2-C3**

Figure S74. Conformers of fragment **F2** for transition state **TSB-Na-E-T**.

Table S74. Energies of conformers of fragment **F2** for transition state **TSB-Na-E-T**.[a]

Structure	Δ*E*
**TSB-Na-E-T-C3**	-767.4717853
**TSB-Na-E-T-C2**	-767.4678667
**TSB-Na-E-T-C1**	-767.4676947

[a]Energies were obtained as single points at the MN15 / def2-TZVP / SMD (DCM) level of theory based on structures optimized at the same level of theory. ΔE, hartree.

TSB-Na-E-C Fragment F1

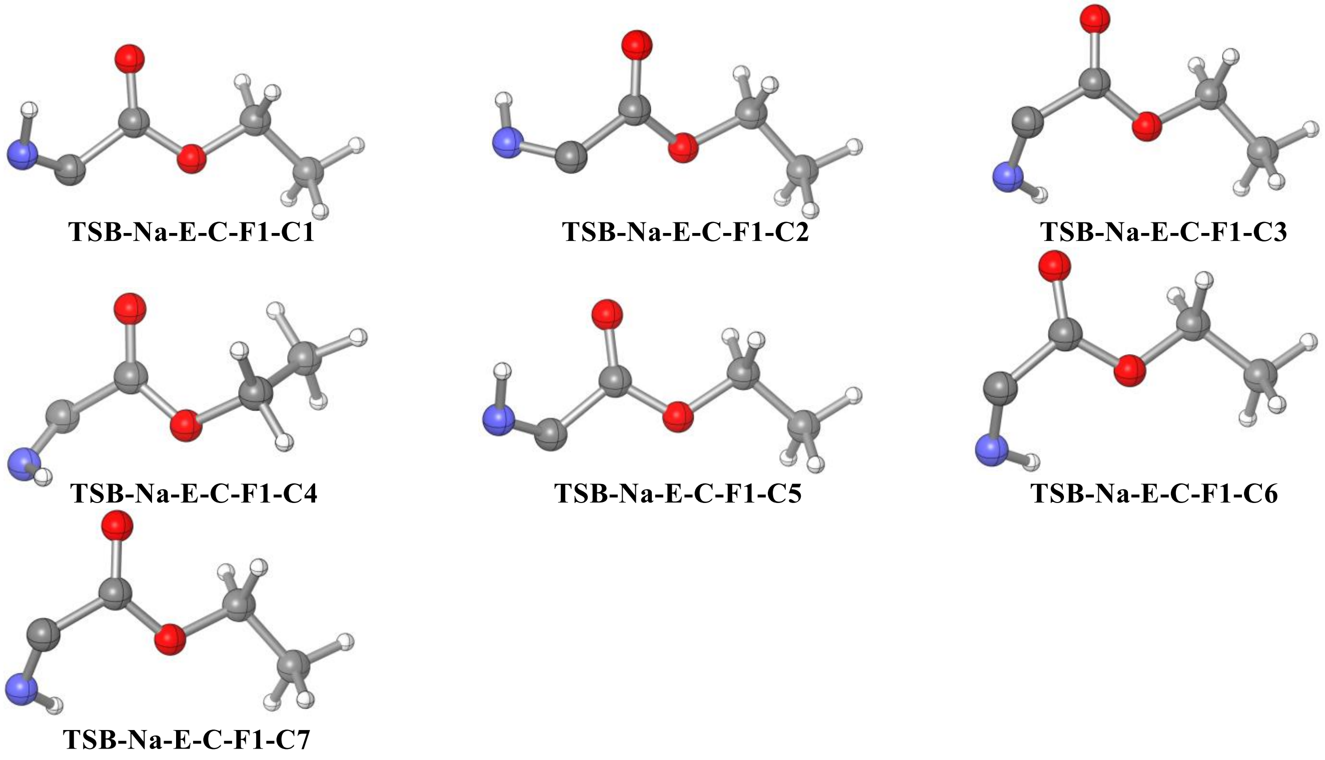

Figure S75. Conformers of fragment **F1** for transition state **TSB-Na-E-C**.

Table S75. Energies of conformers of fragment **F1** for transition state **TSB-Na-E-C**.[a]

Structure	Δ*E*
**TSB-Na-E-C5**	-360.8893635
**TSB-Na-E-C1**	-360.8881534

**TSB-Na-E-C2**	-360.8879725
**TSB-Na-E-C7**	-360.8878785
**TSB-Na-E-C6**	-360.8876561
**TSB-Na-E-C3**	-360.8869037
**TSB-Na-E-C4**	-360.8864558

[a]Energies were obtained as single points at the MN15 / def2-TZVP / SMD (DCM) level of theory based on structures optimized at the same level of theory. ΔE, hartree.

TSB-Na-E-C Fragment F2

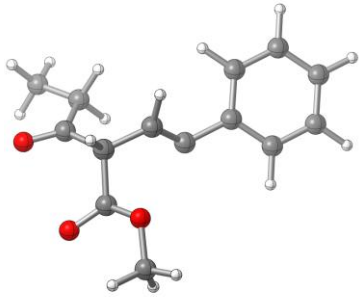

TSB-Na-E-C-F2-C1

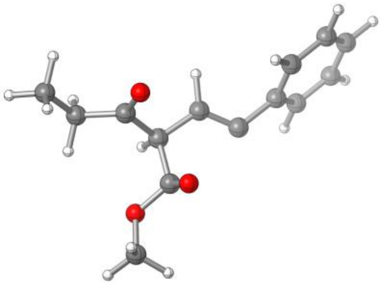

TSB-Na-E-C-F2-C2

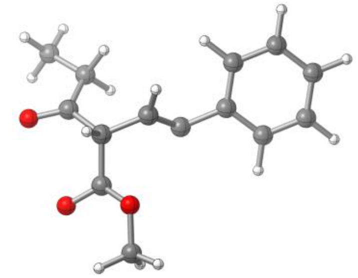

TSB-Na-E-C-F2-C3

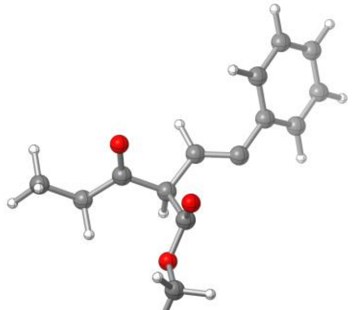

TSB-Na-E-C-F2-C4

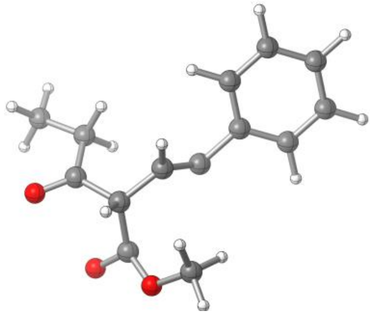

TSB-Na-E-C-F2-C5

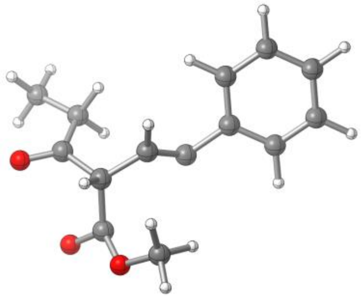

TSB-Na-E-C-F2-C6

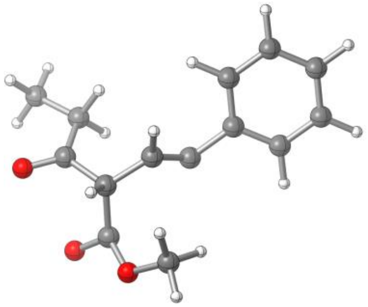

TSB-Na-E-C-F2-C7

Figure S76. Conformers of fragment **F2** for transition state **TSB-Na-E-C**.

Table S76. Energies of conformers of fragment **F2** for transition state **TSB-Na-E-C**.[a]

Structure	Δ*E*
**TSB-Na-E-C4**	-767.4713784
**TSB-Na-E-C2**	-767.4710121
**TSB-Na-E-C1**	-767.4682315
**TSB-Na-E-C3**	-767.4678666
**TSB-Na-E-C6**	-767.4673483
**TSB-Na-E-C7**	-767.4672787
**TSB-Na-E-C5**	-767.4669896

[a]Energies were obtained as single points at the MN15 / def2-TZVP / SMD (DCM) level of theory based on structures optimized at the same level of theory. ΔE, hartree.

9. NMR spectra

^{1}H-NMR (500 MHz, $CDCl_3$) of compound 4a

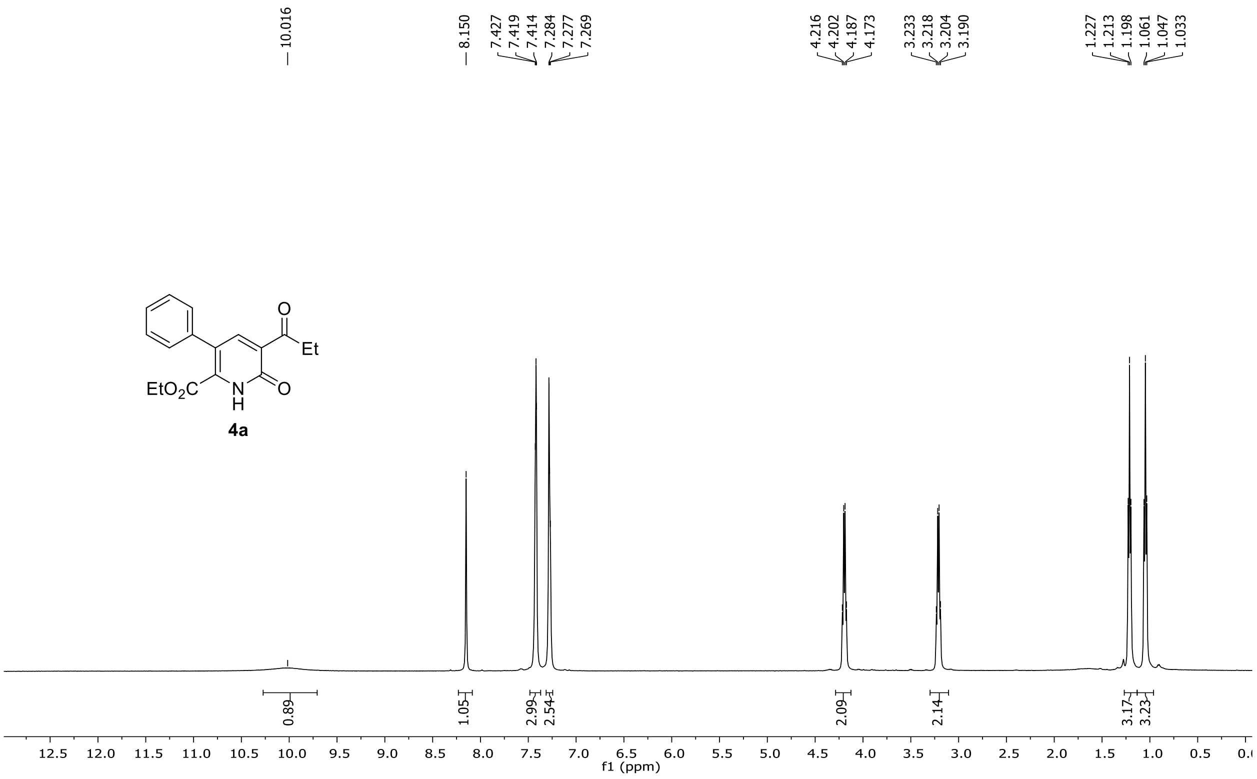

^{13}C-NMR (126 MHz, $CDCl_3$) of compound 4a

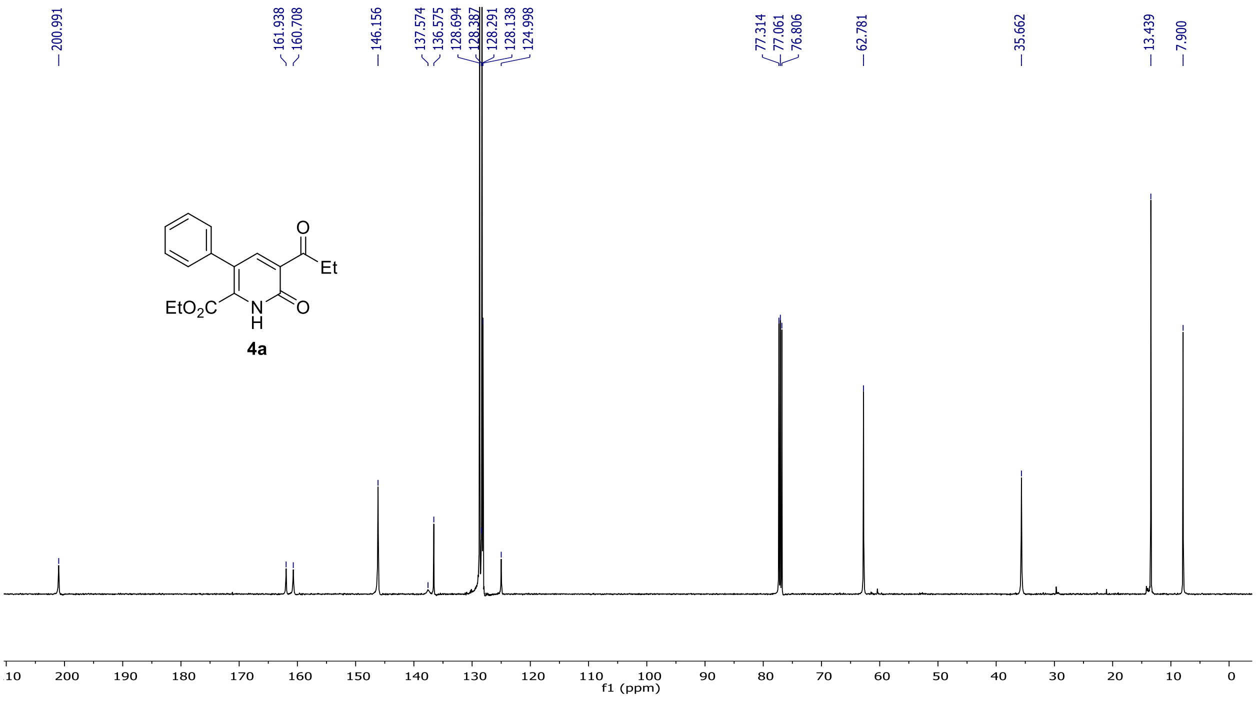

^{1}H-NMR (500 MHz, $CDCl_3$) of compound 4b

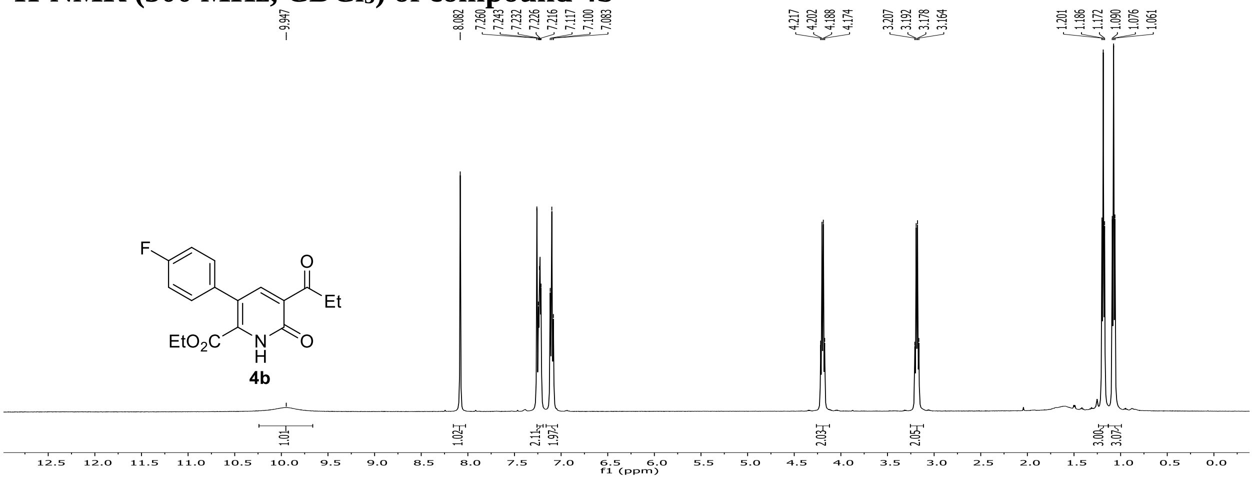

^{13}C-NMR (126 MHz, $CDCl_3$) of compound 4b

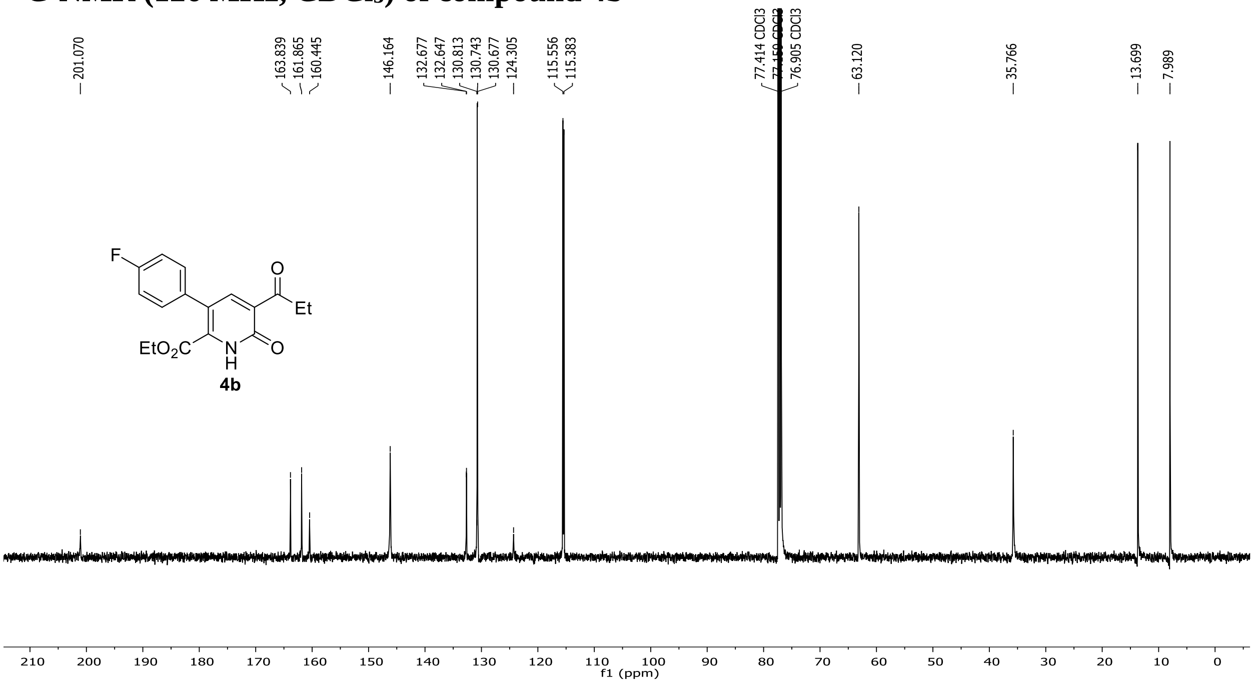

^{19}F-NMR (471 MHz, $CDCl_3$) of compound 4b

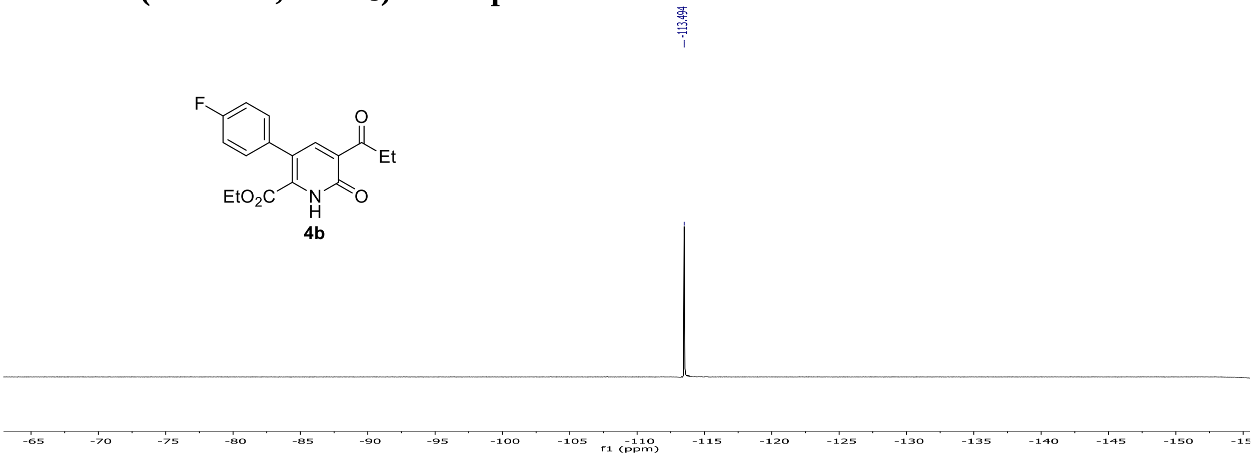

^{1}H-NMR (500 MHz, $CDCl_3$) of compound 4c

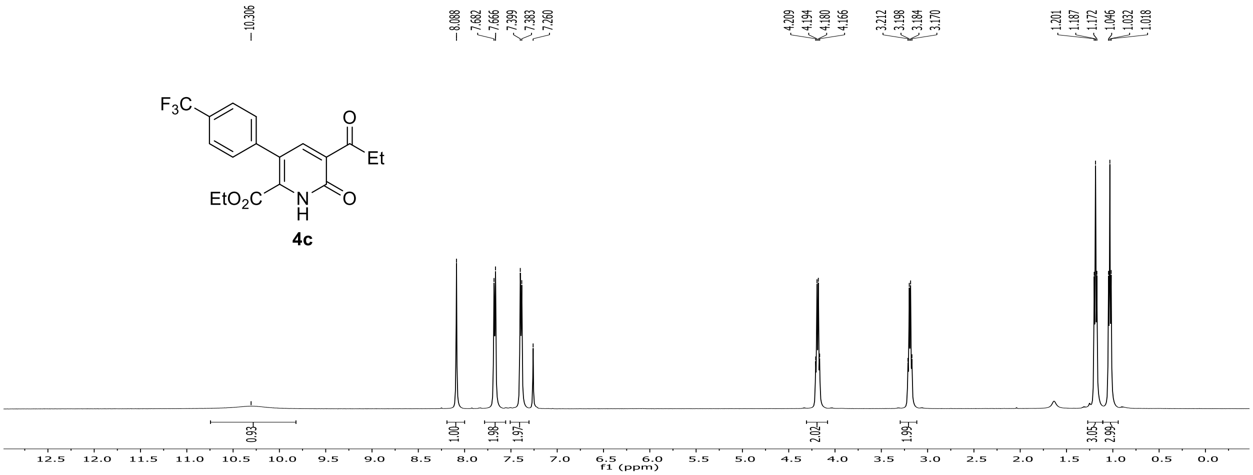

^{13}C-NMR (126 MHz, $CDCl_3$) of compound 4c

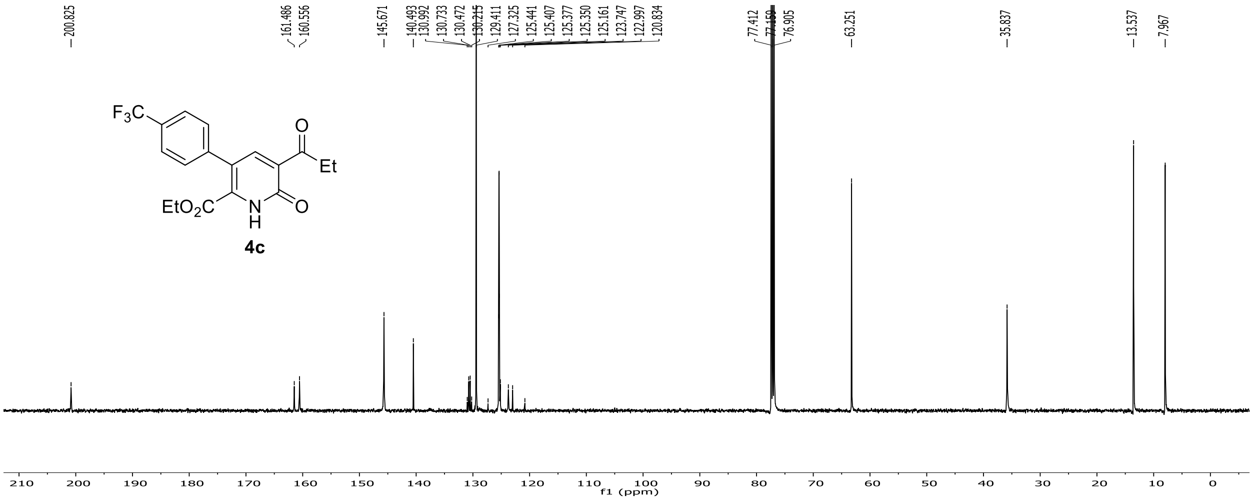

^{19}F-NMR (471 MHz, $CDCl_3$) of compound 4c

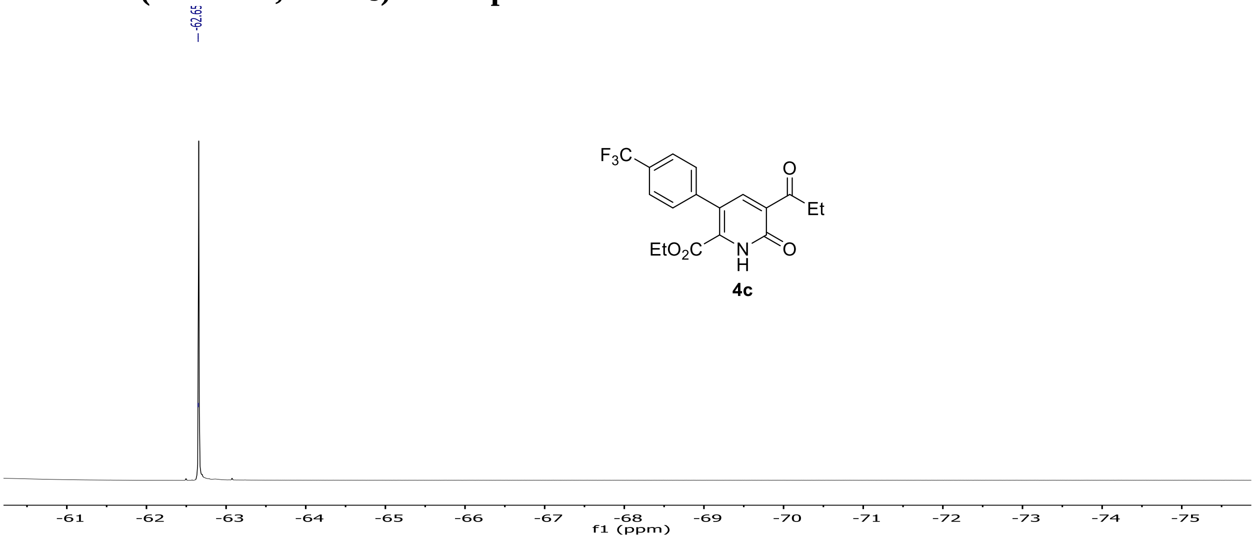

^{1}H-NMR (500 MHz, $CDCl_3$) of compound 4d

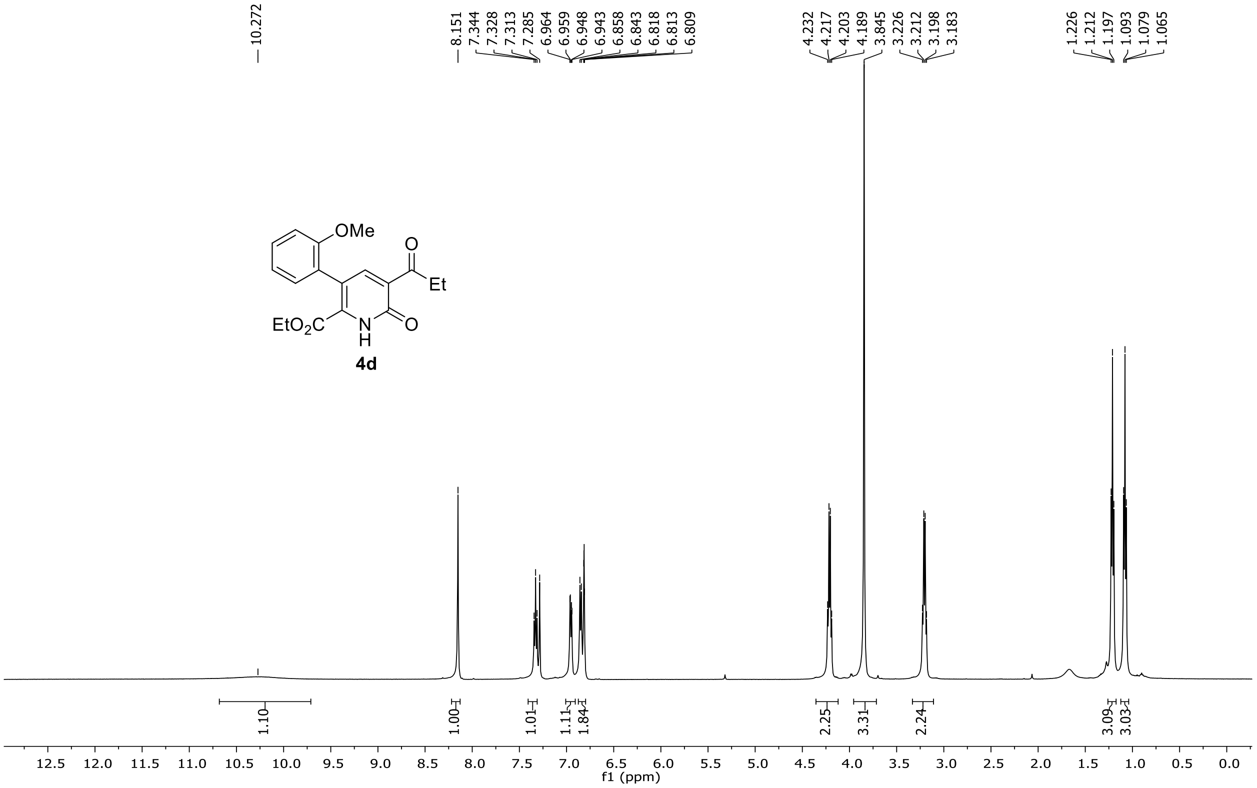

^{13}C-NMR (126 MHz, $CDCl_3$) of compound 4d

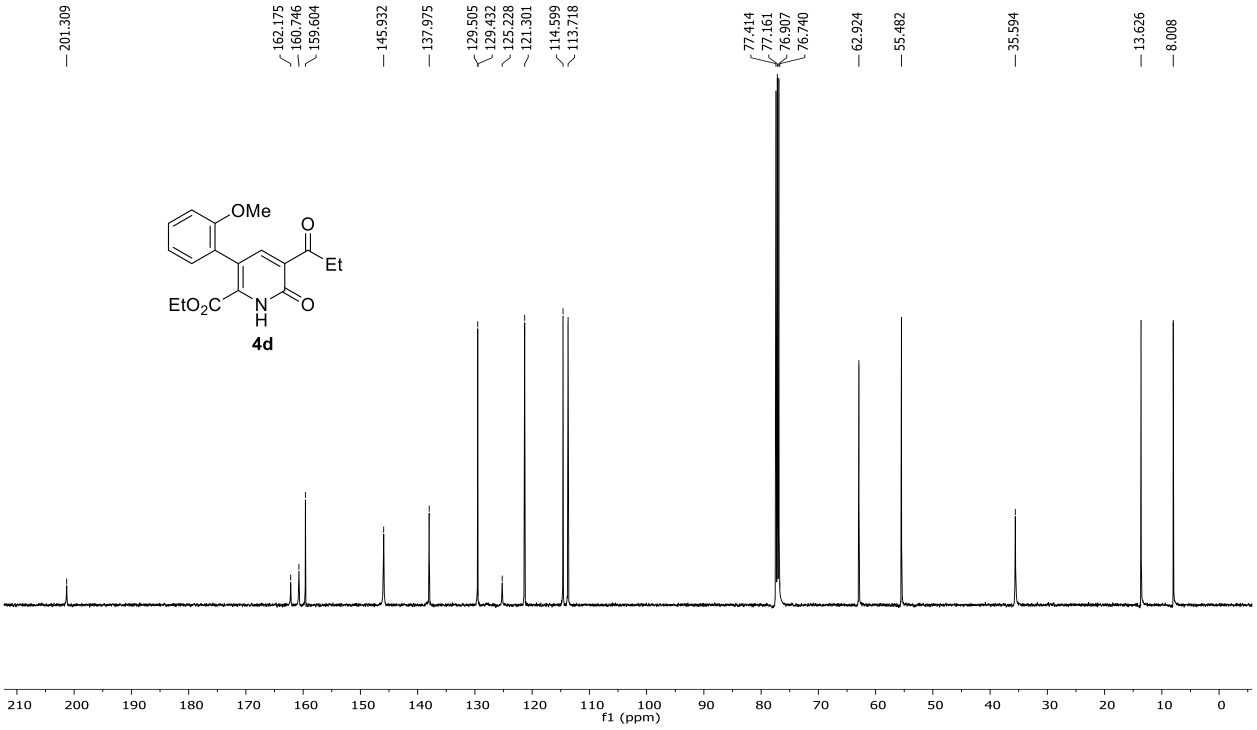

^{1}H-NMR (500 MHz, CDCl3) of compound 4e

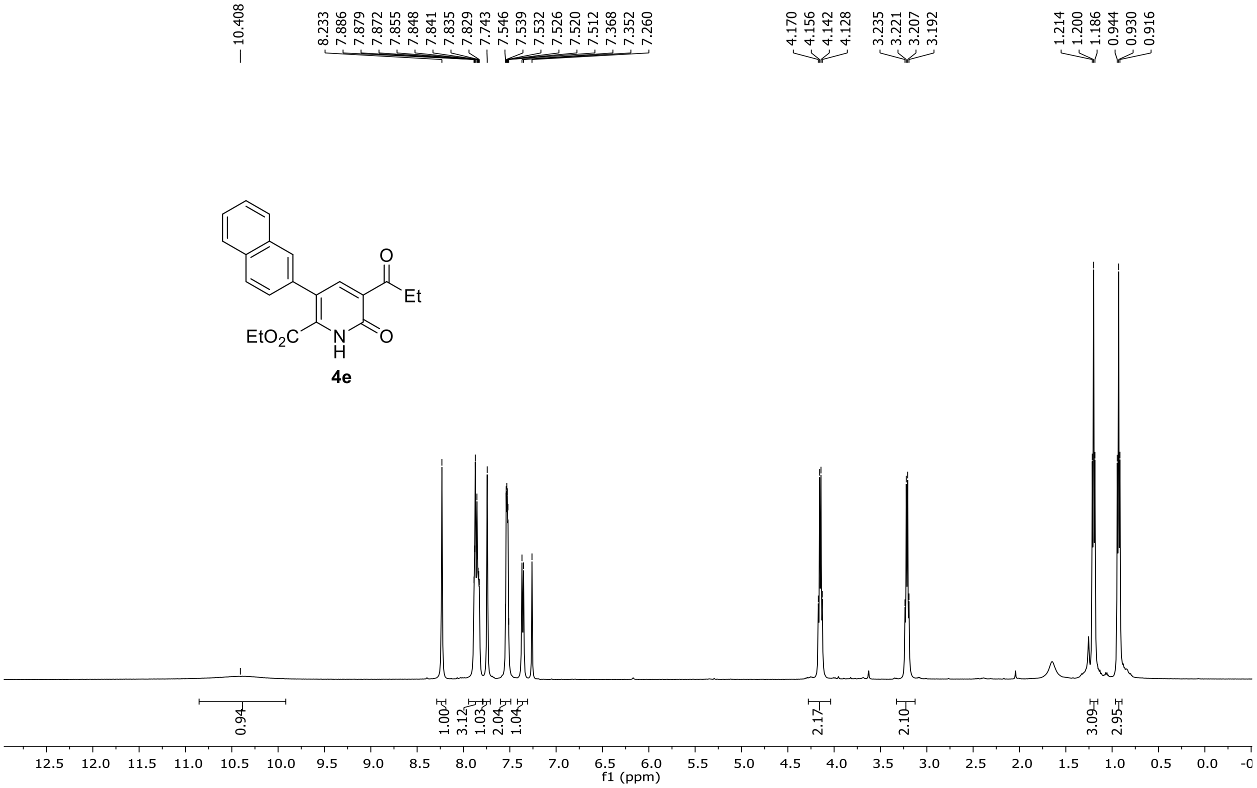

^{13}C-NMR (126 MHz, CDCl3) of compound 4e

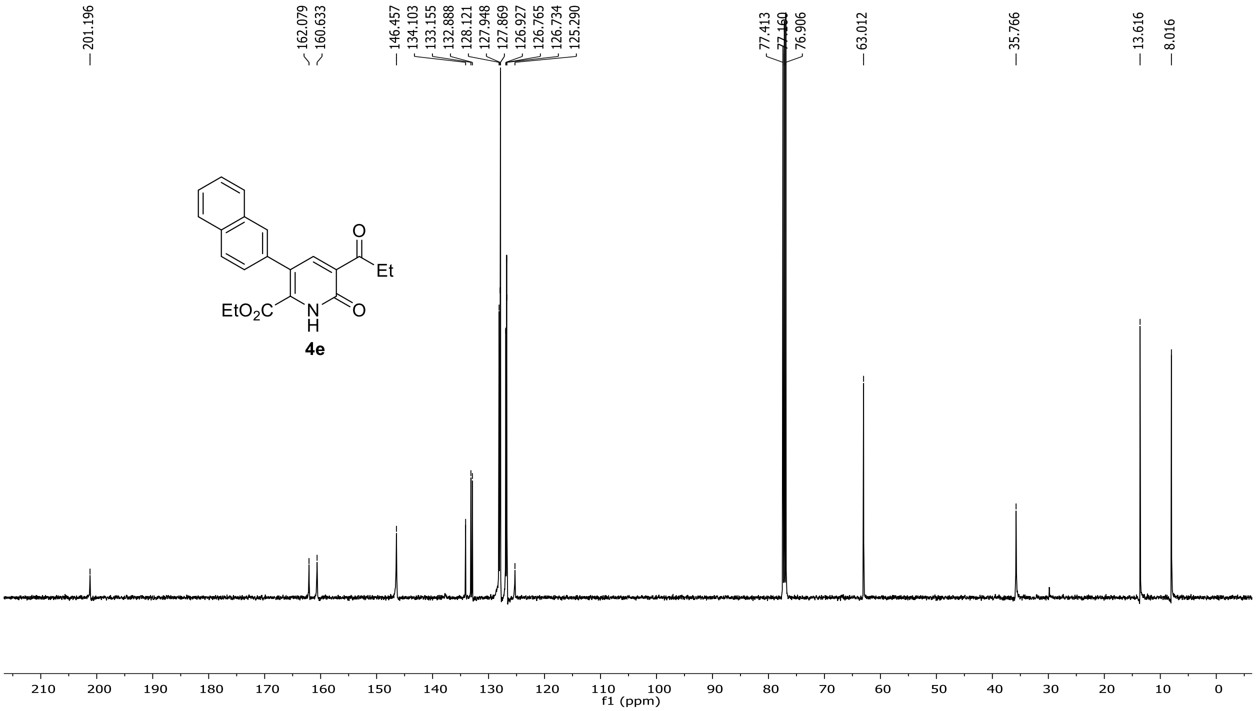

^{1}H-NMR (500 MHz, $CDCl_3$) of compound 4f

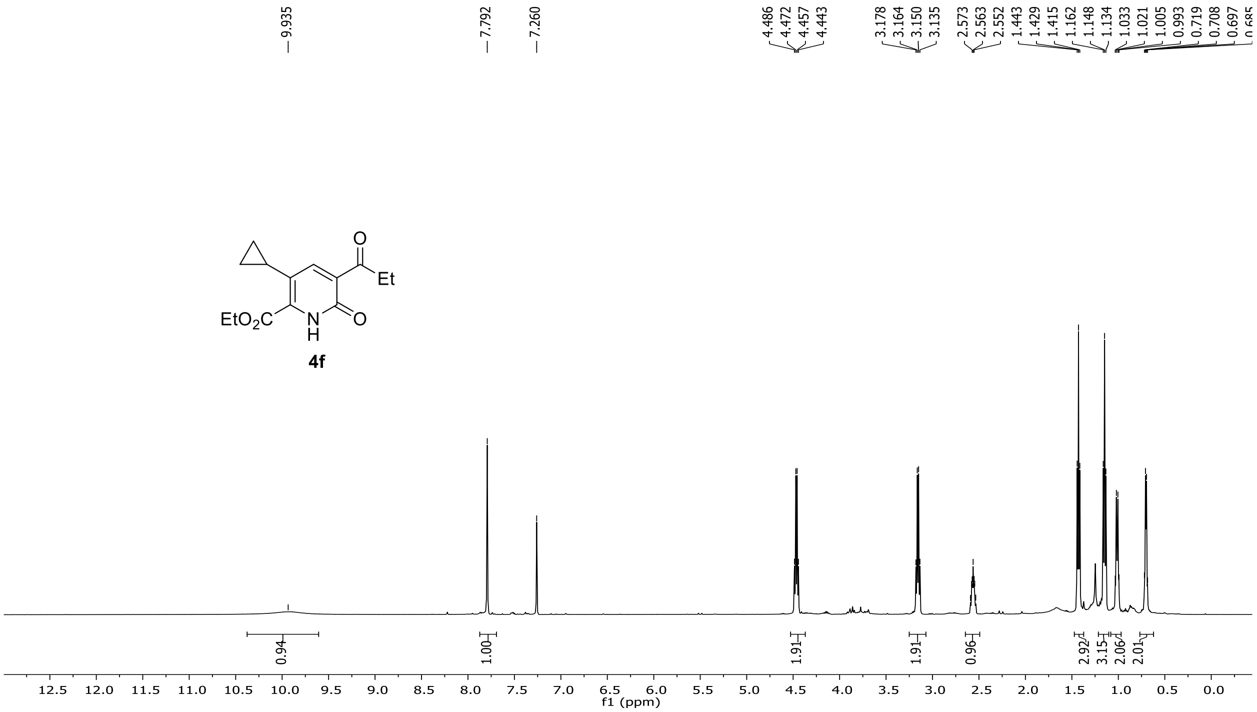

^{13}C-NMR (126 MHz, $CDCl_3$) of compound 4f

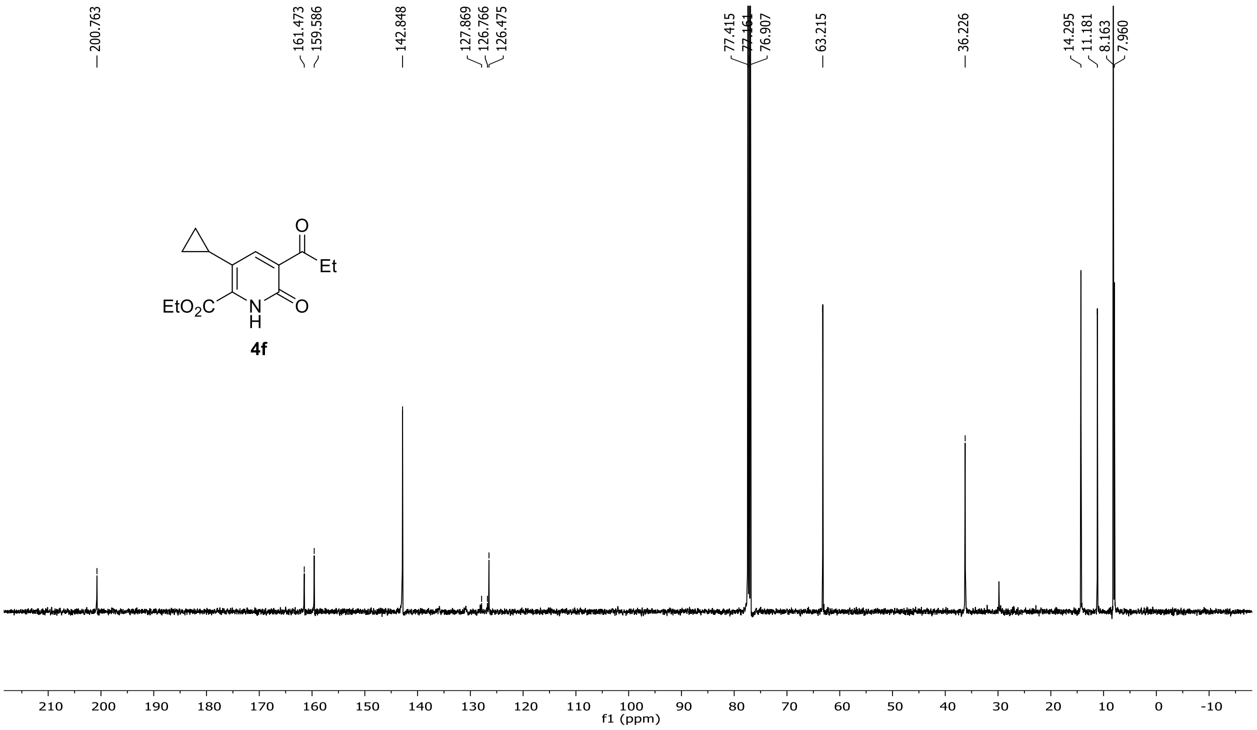

^{1}H-NMR (500 MHz, $CDCl_3$) of compound 4g

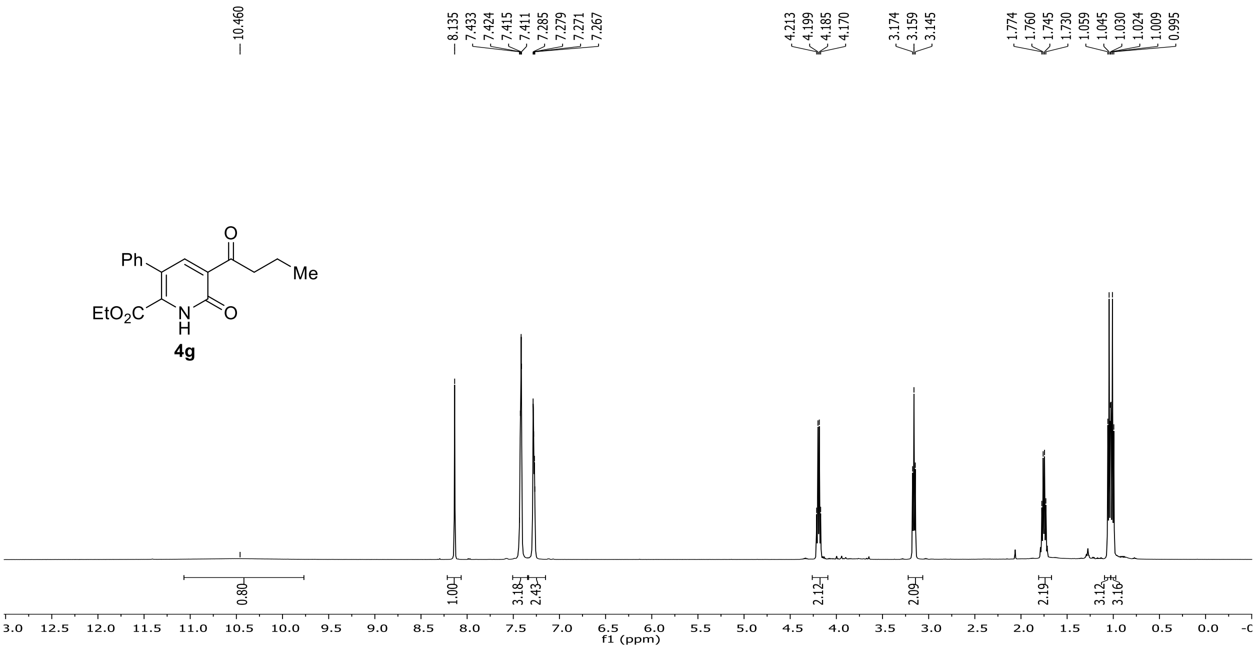

^{13}C-NMR (126 MHz, $CDCl_3$) of compound 4g

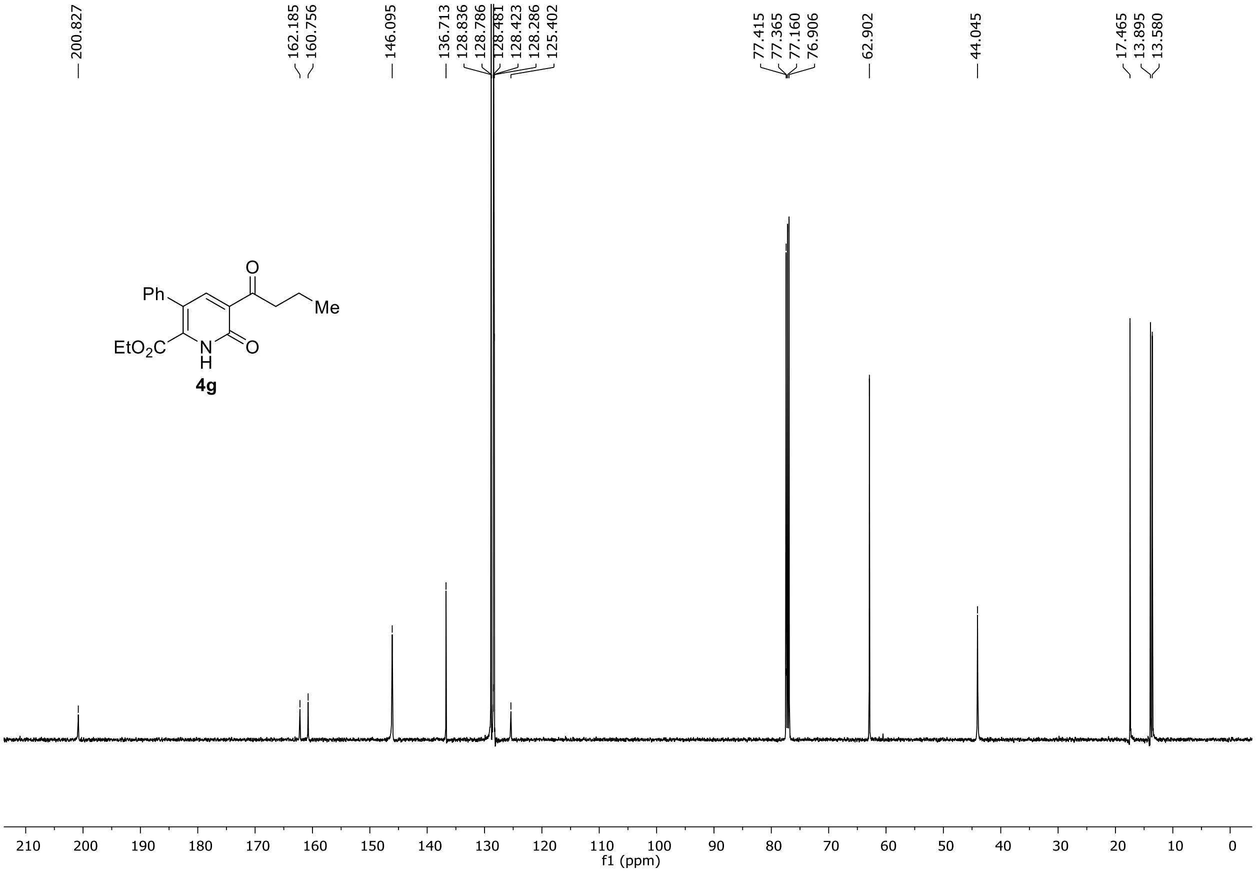

^{1}H-NMR (500 MHz, $CDCl_3$) of compound 4h

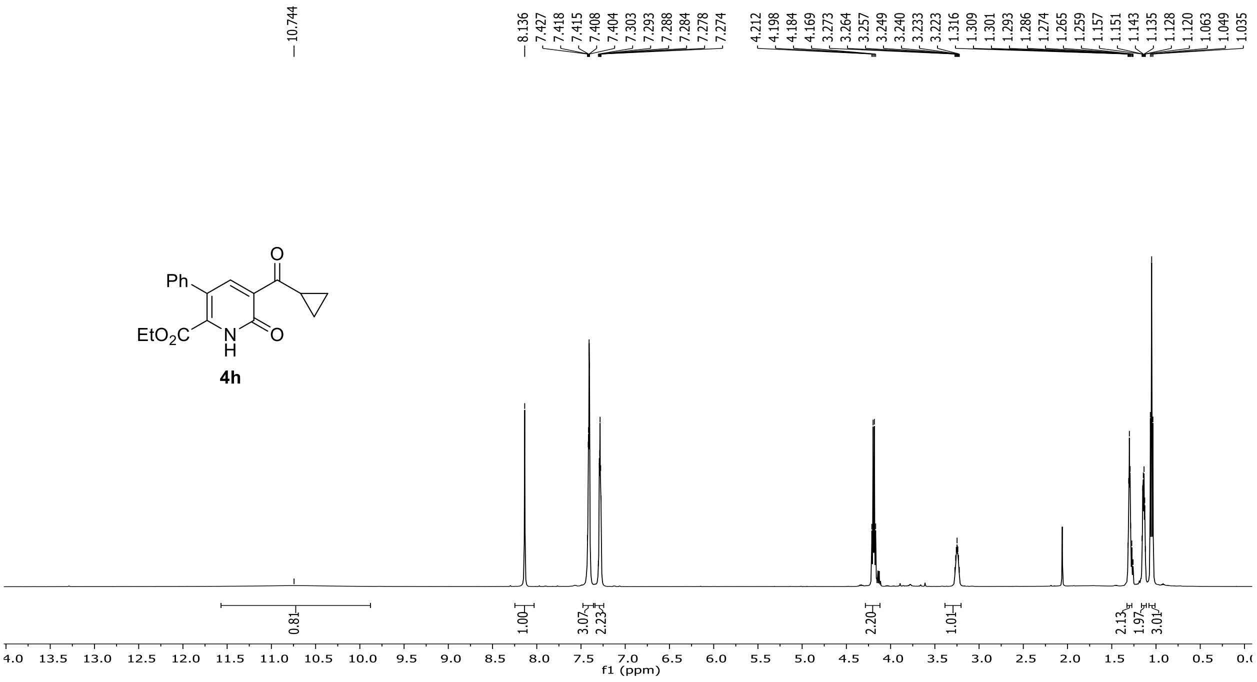

^{13}C-NMR (126 MHz, $CDCl_3$) of compound 4h

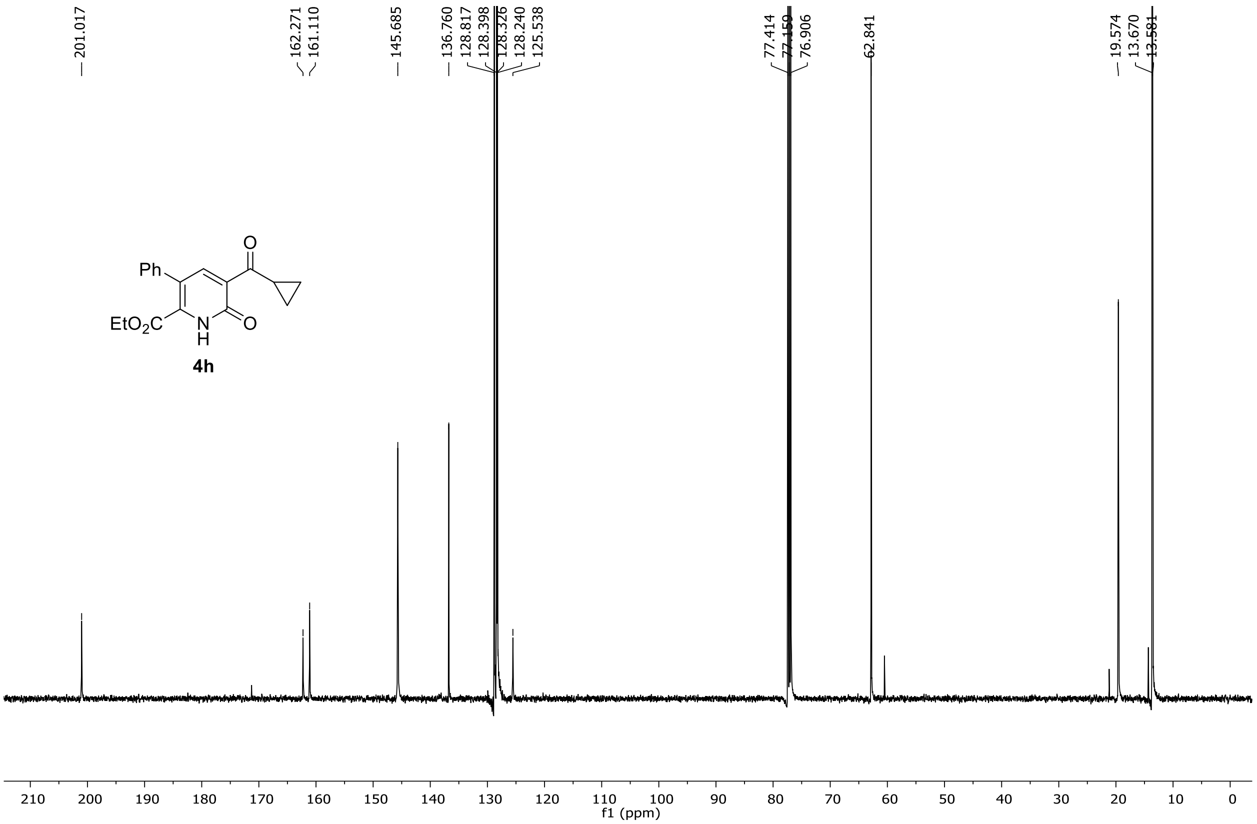

^{1}H-NMR (500 MHz, $CDCl_3$) of compound 4i

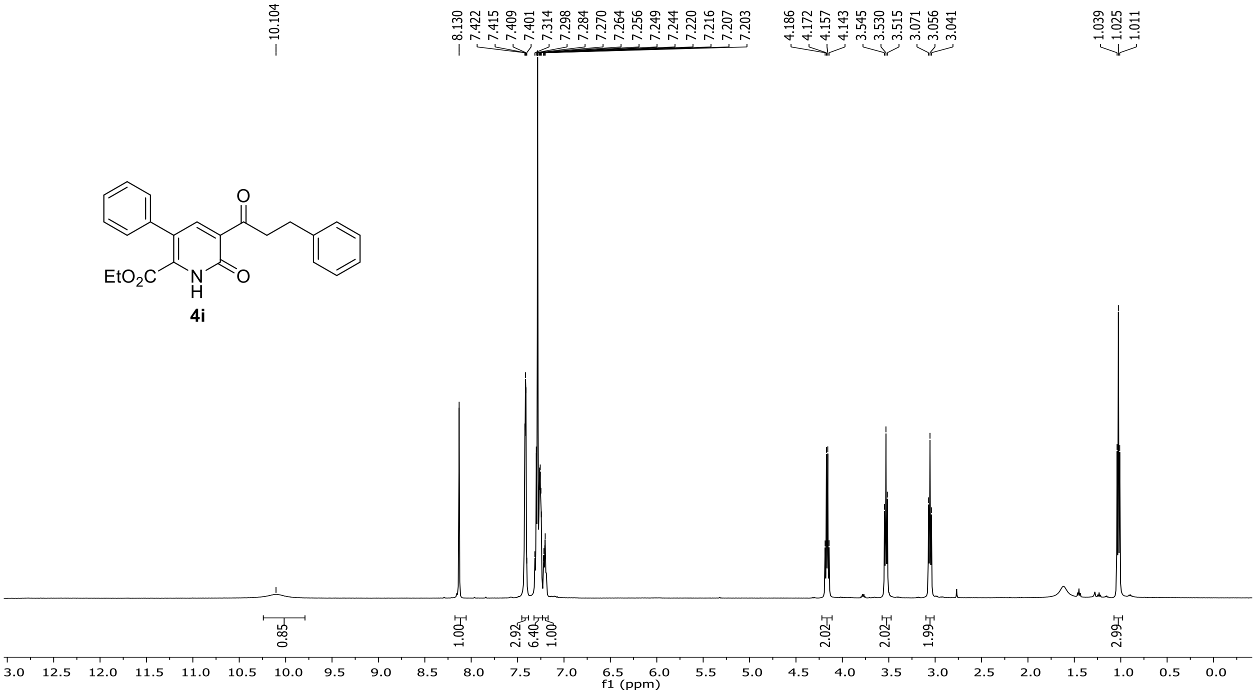

^{13}C-NMR (126 MHz, $CDCl_3$) of compound 4i

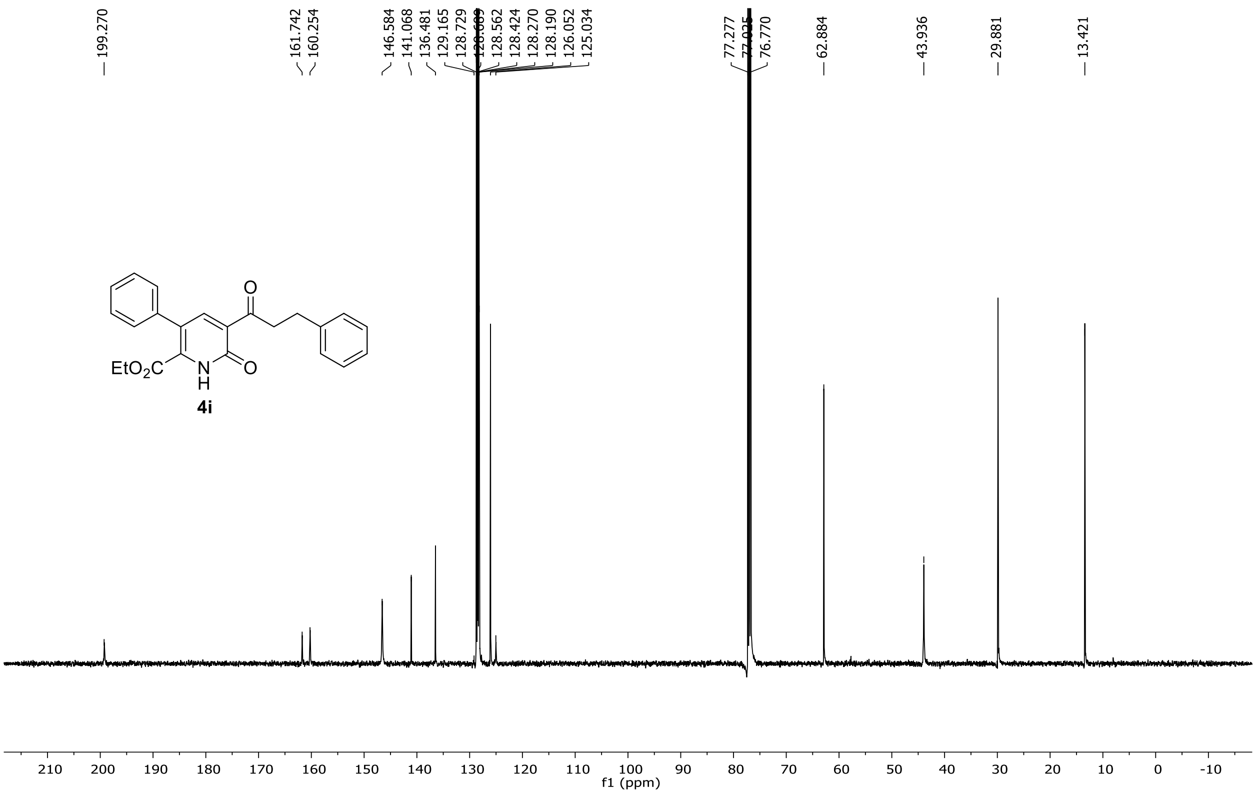

^{1}H-NMR (500 MHz, $CDCl_3$) of compound 4j

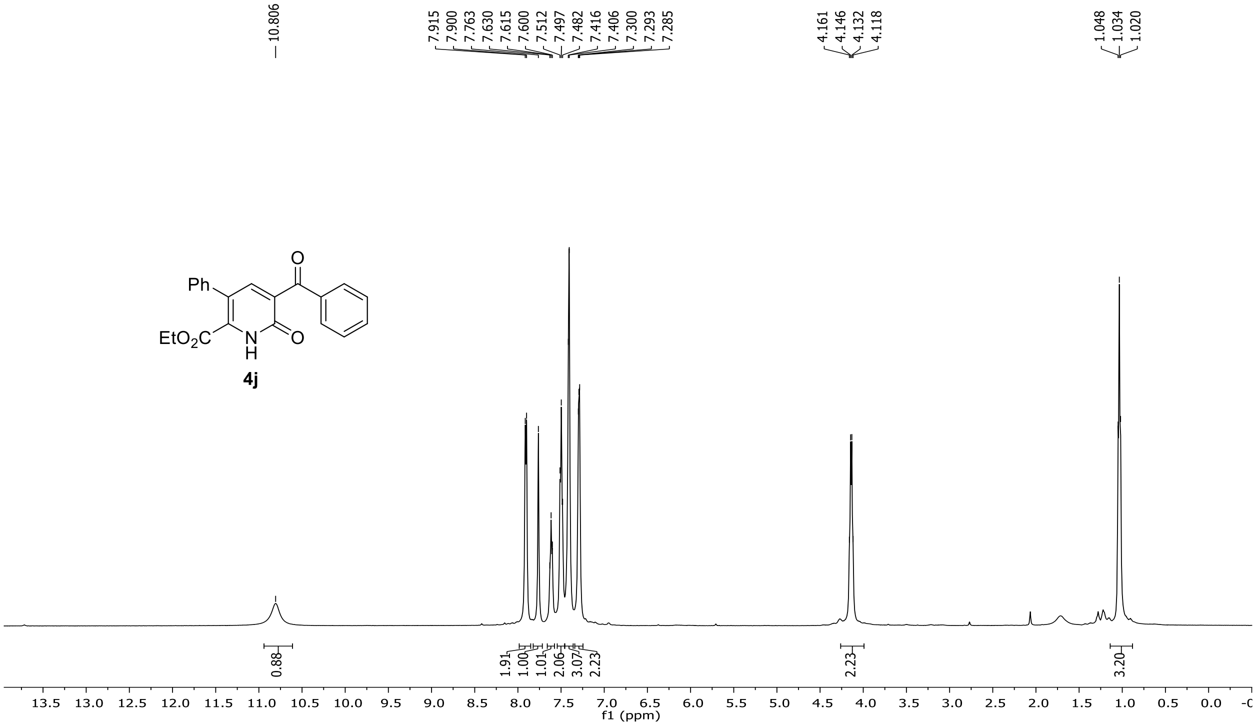

^{13}C-NMR (126 MHz, $CDCl_3$) of compound 4j

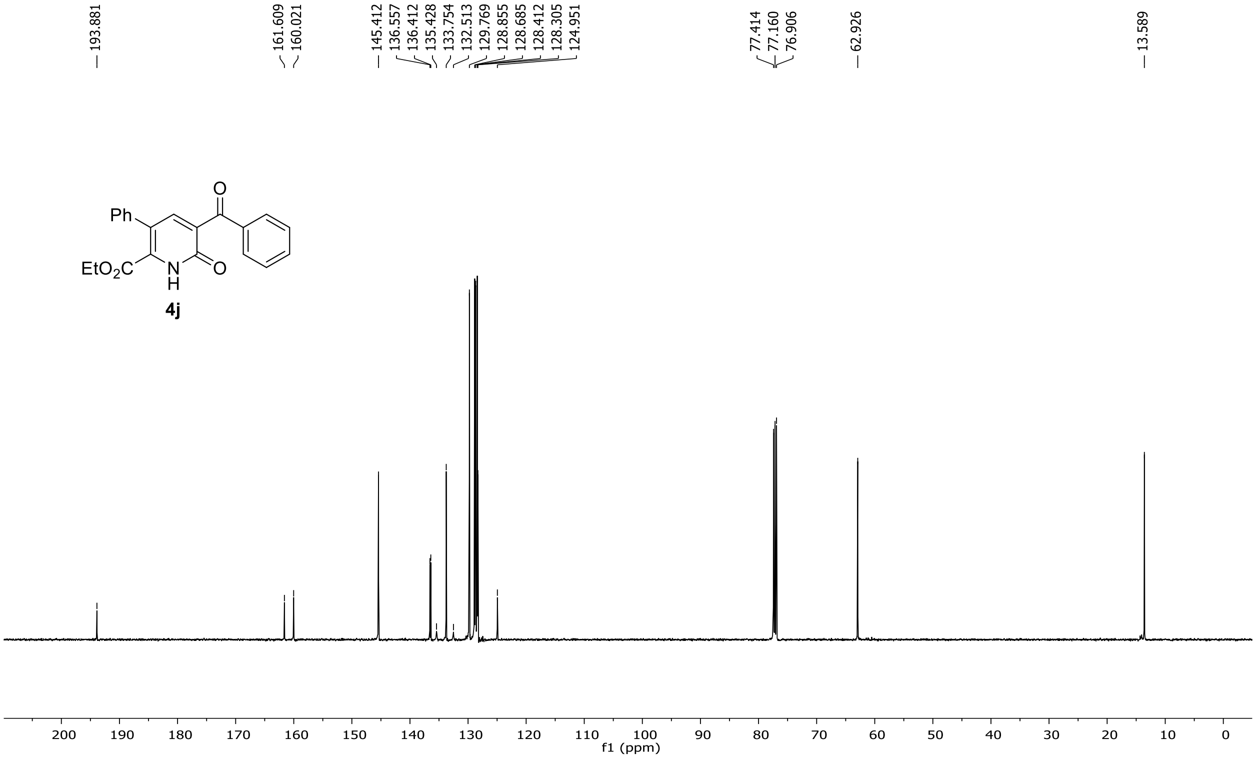

^{1}H-NMR (500 MHz, $CDCl_3$) of compound 4k

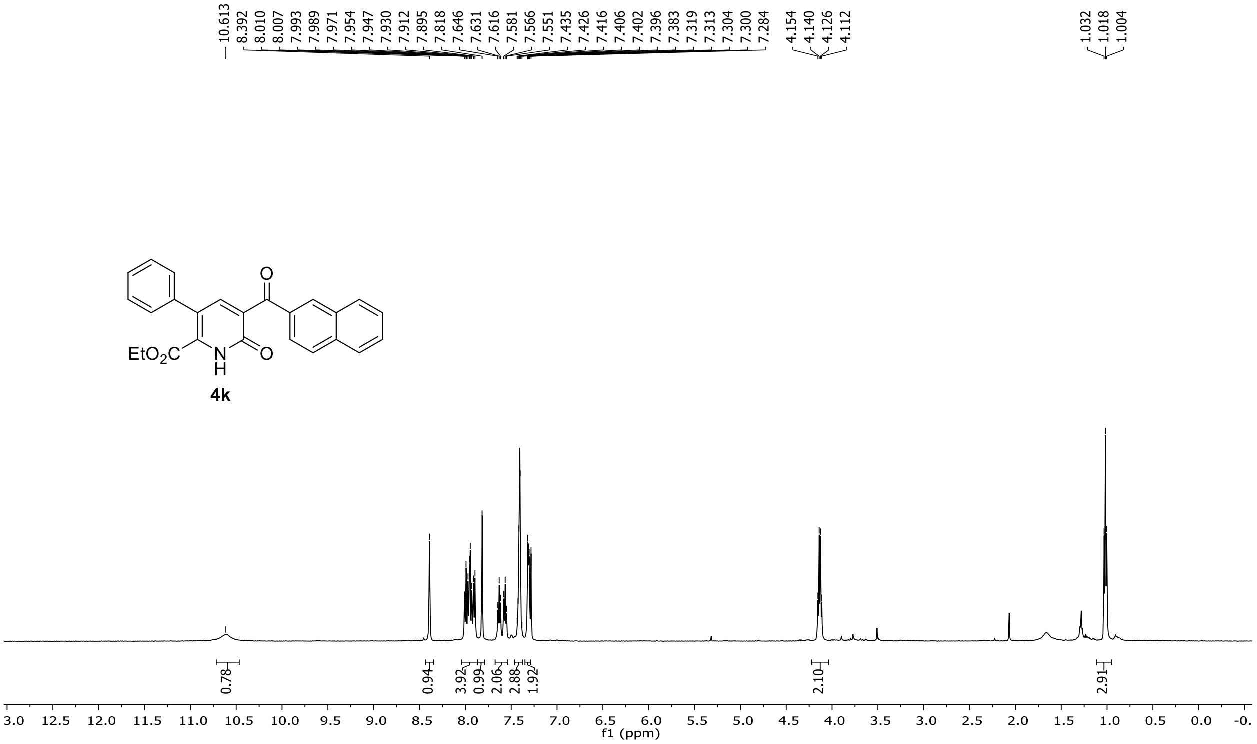

^{13}C-NMR (126 MHz, $CDCl_3$) of compound 4k

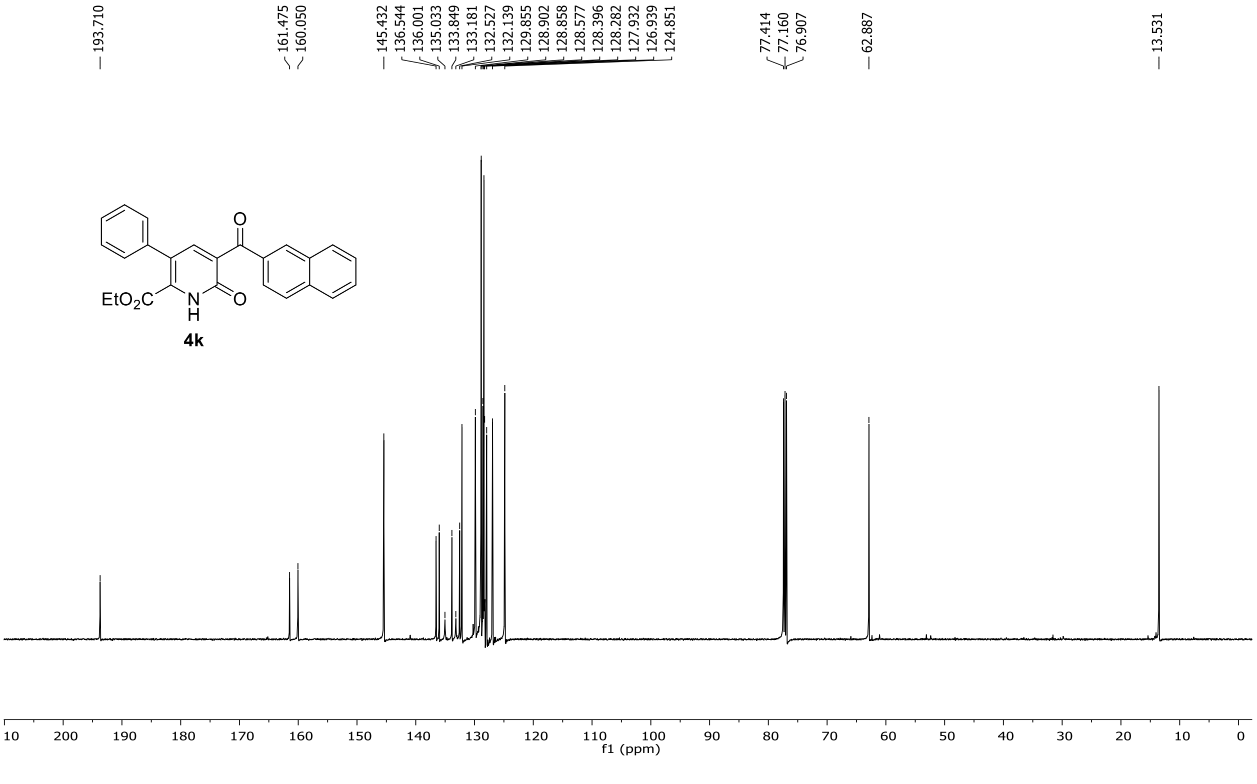

^{1}H-NMR (500 MHz, $CDCl_3$) of compound 4l

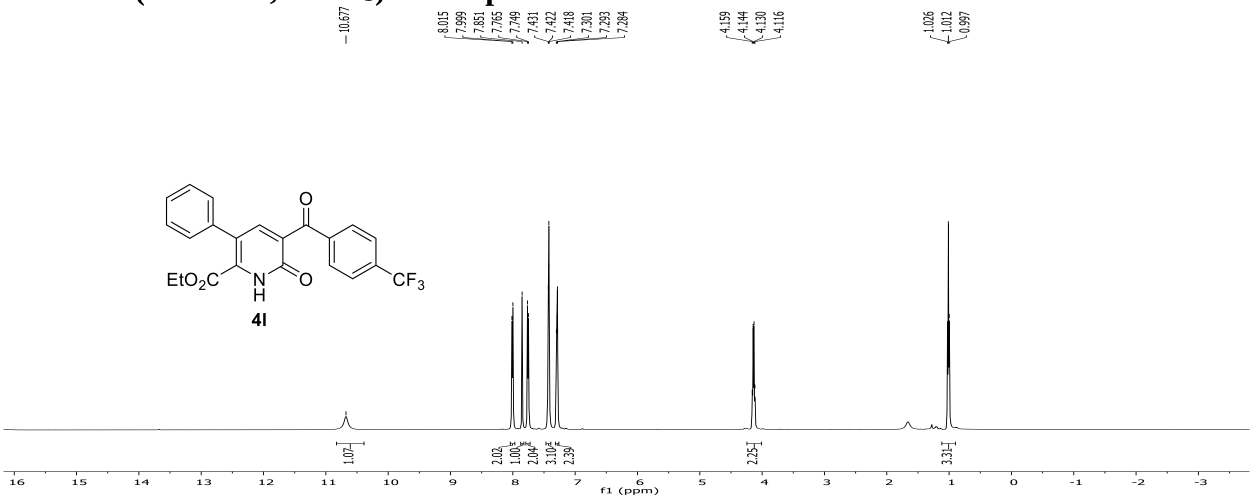

^{13}C-NMR (126 MHz, $CDCl_3$) of compound 4l

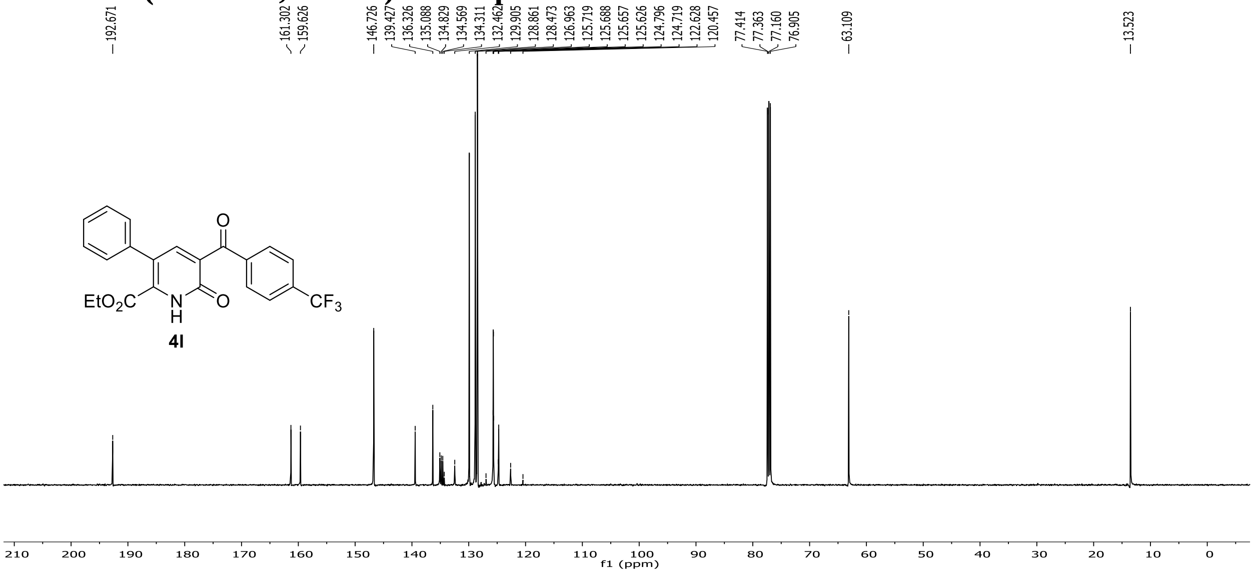

^{19}F-NMR (471 MHz, $CDCl_3$) of compound 4l

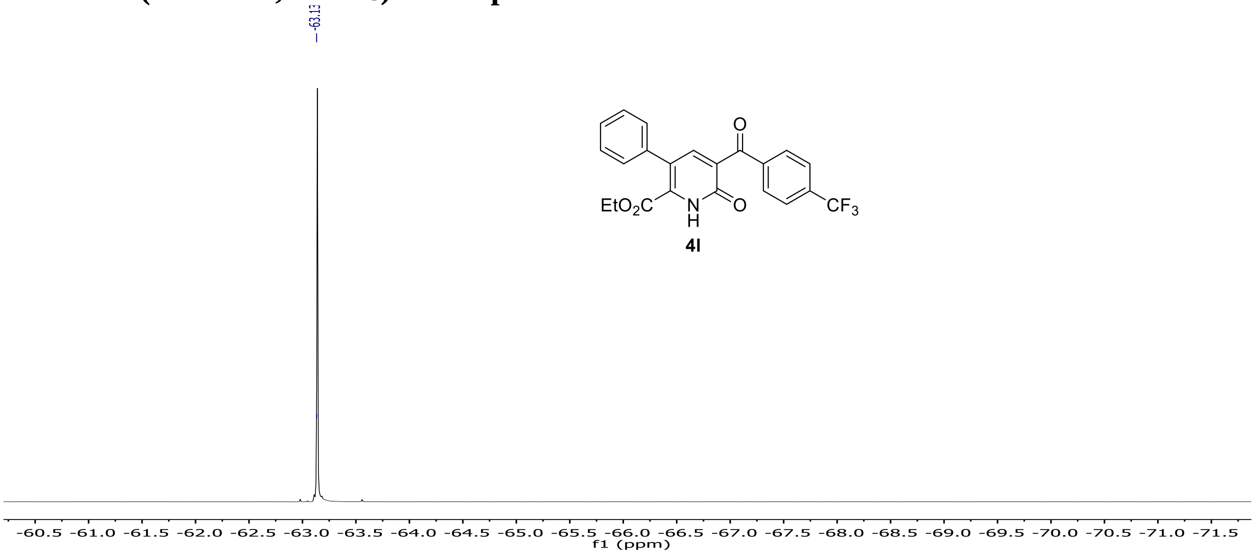

^{1}H-NMR (500 MHz, $CDCl_3$) of compound 4m

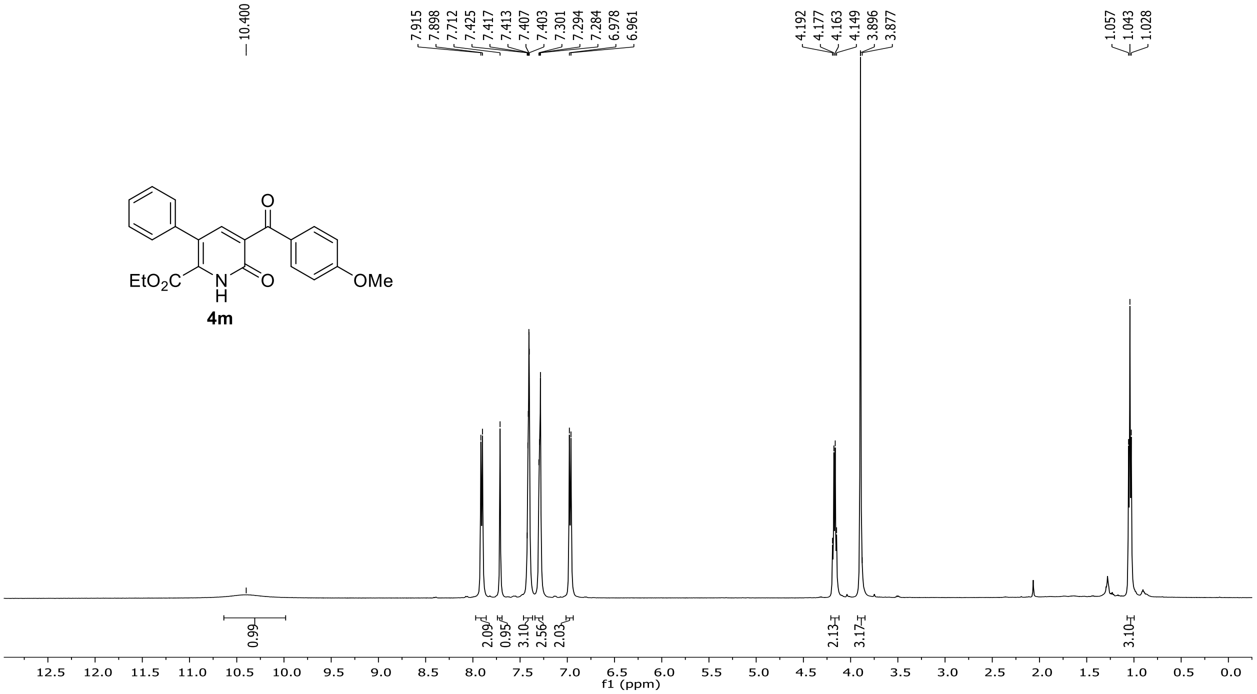

^{13}C-NMR (126 MHz, $CDCl_3$) of compound 4m

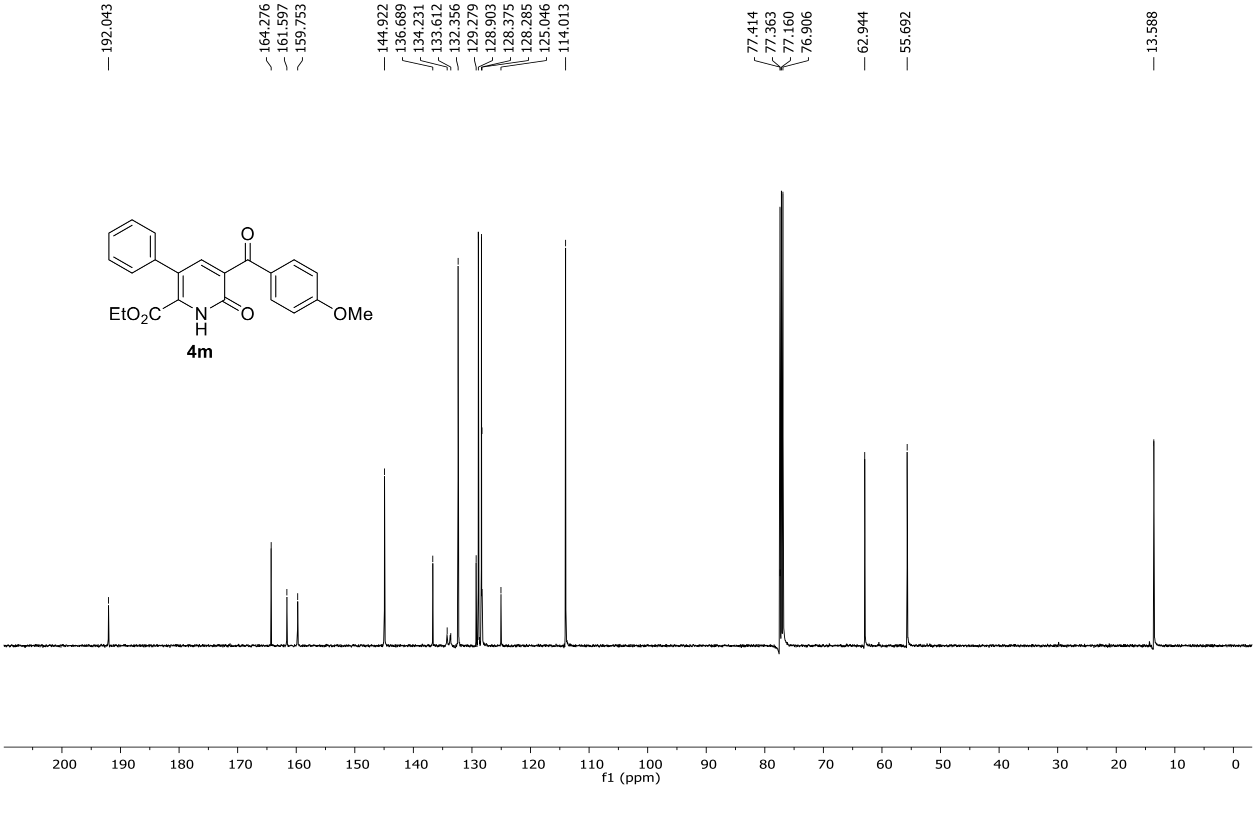

^{1}H-NMR (500 MHz, $CDCl_3$) of compound 4n

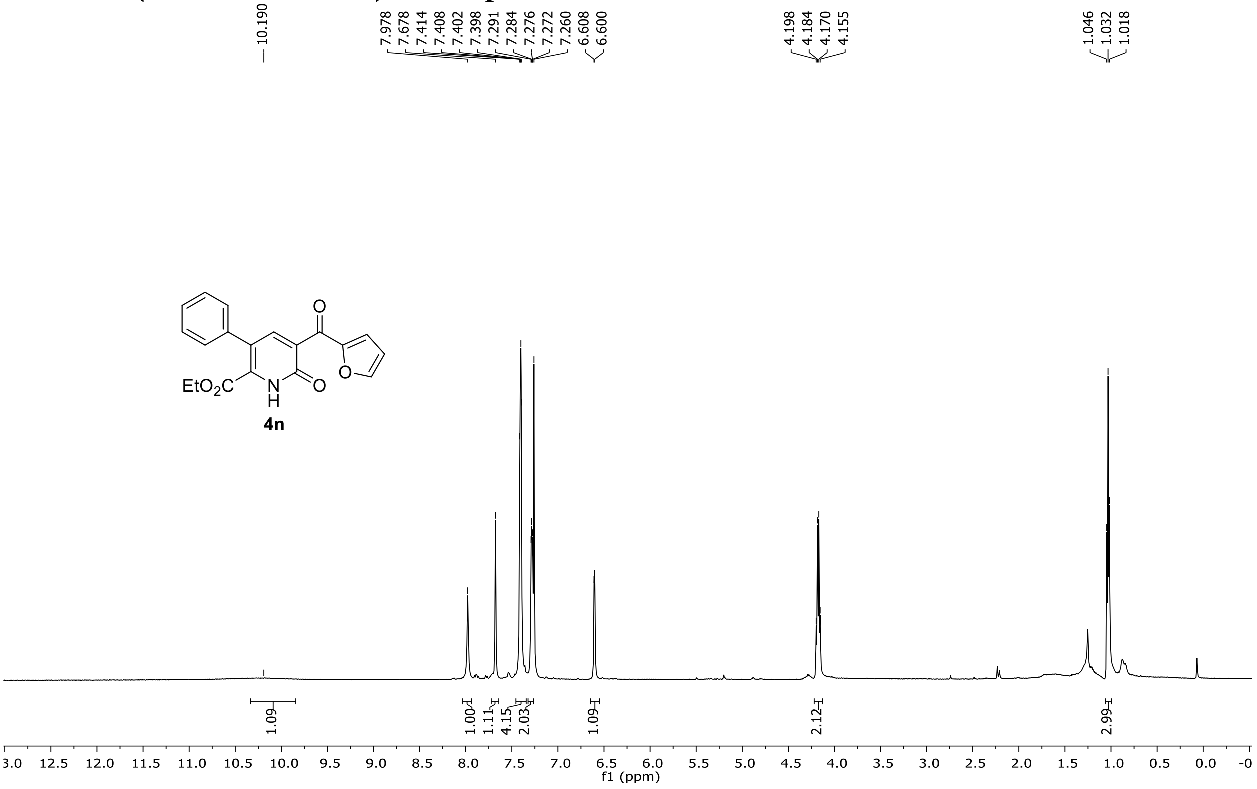

^{13}C-NMR (126 MHz, $CDCl_3$) of compound 4n

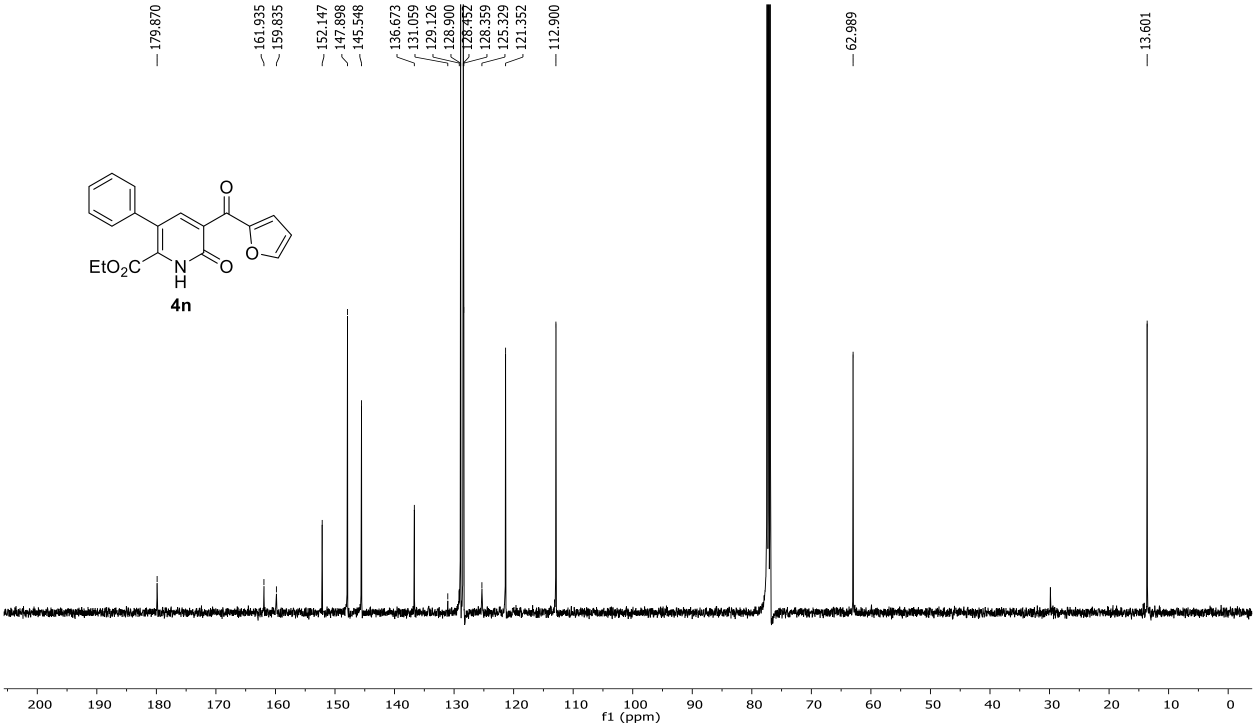

^{1}H-NMR (500 MHz, $CDCl_3$) of compound 4o

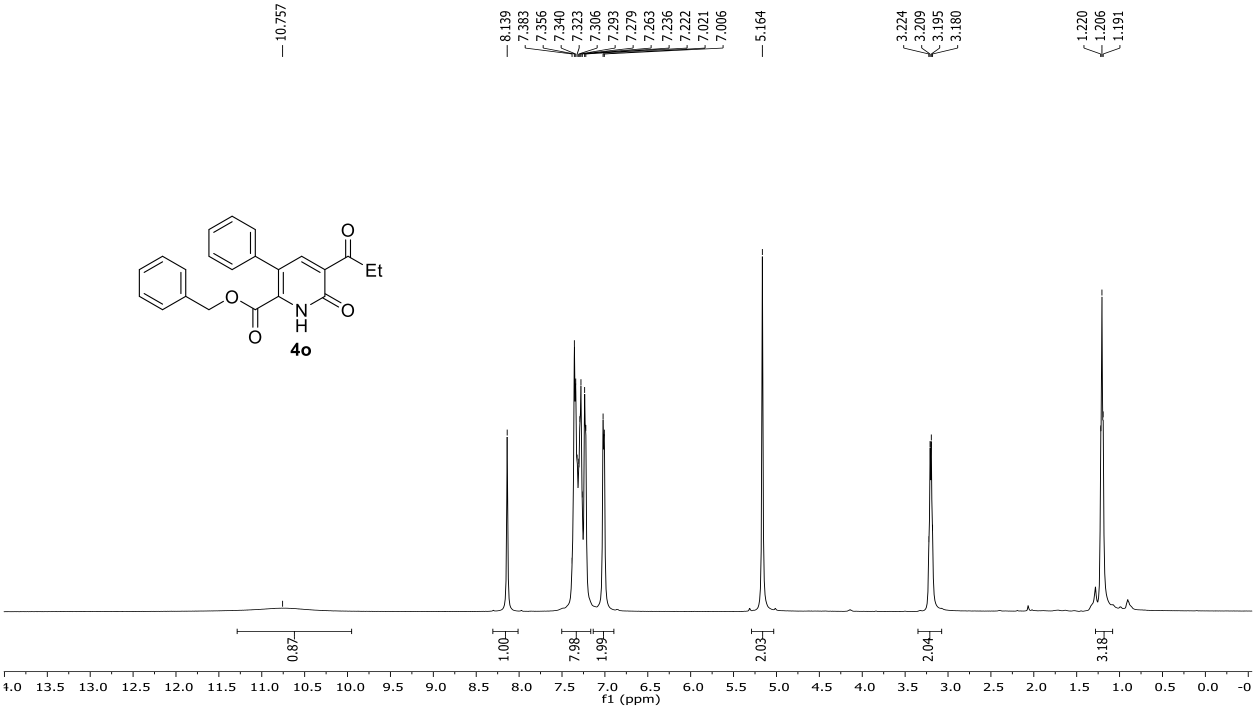

^{13}C-NMR (126 MHz, $CDCl_3$) of compound 4o

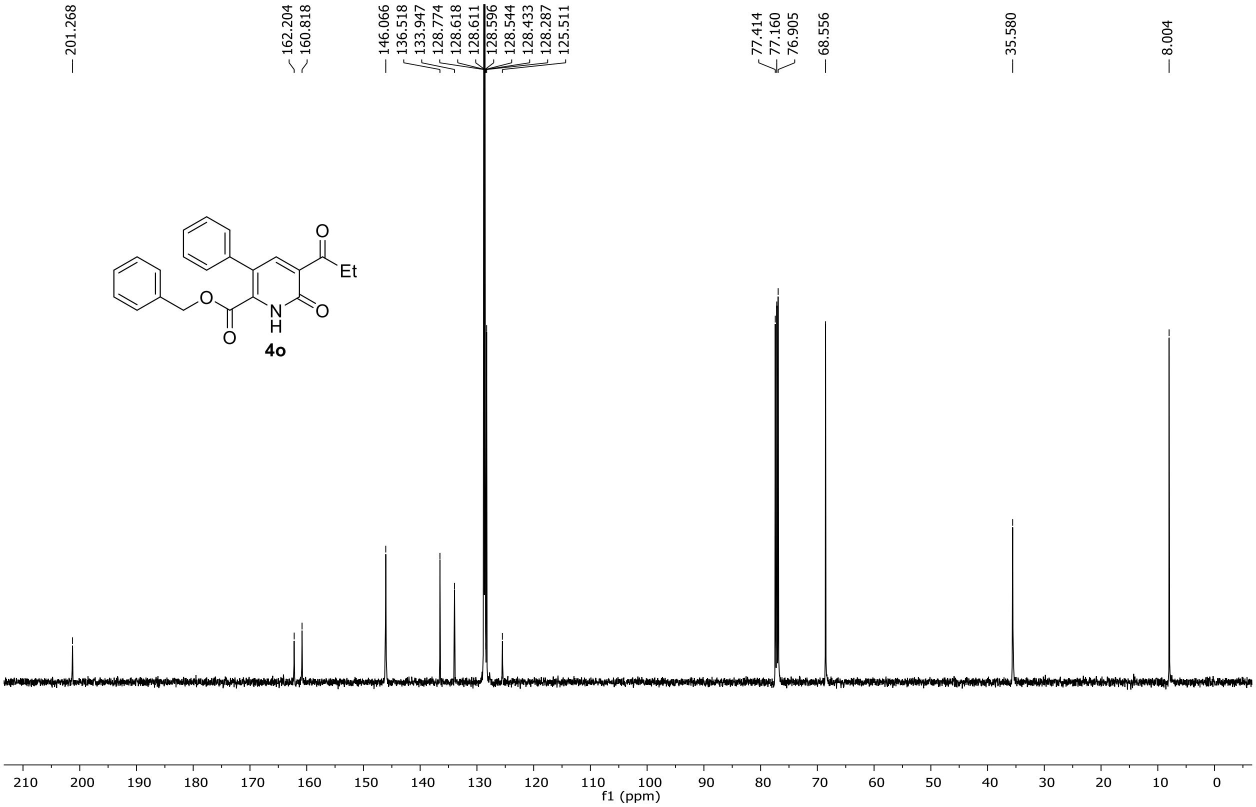

^{1}H-NMR (500 MHz, $CDCl_3$) of compound 4p

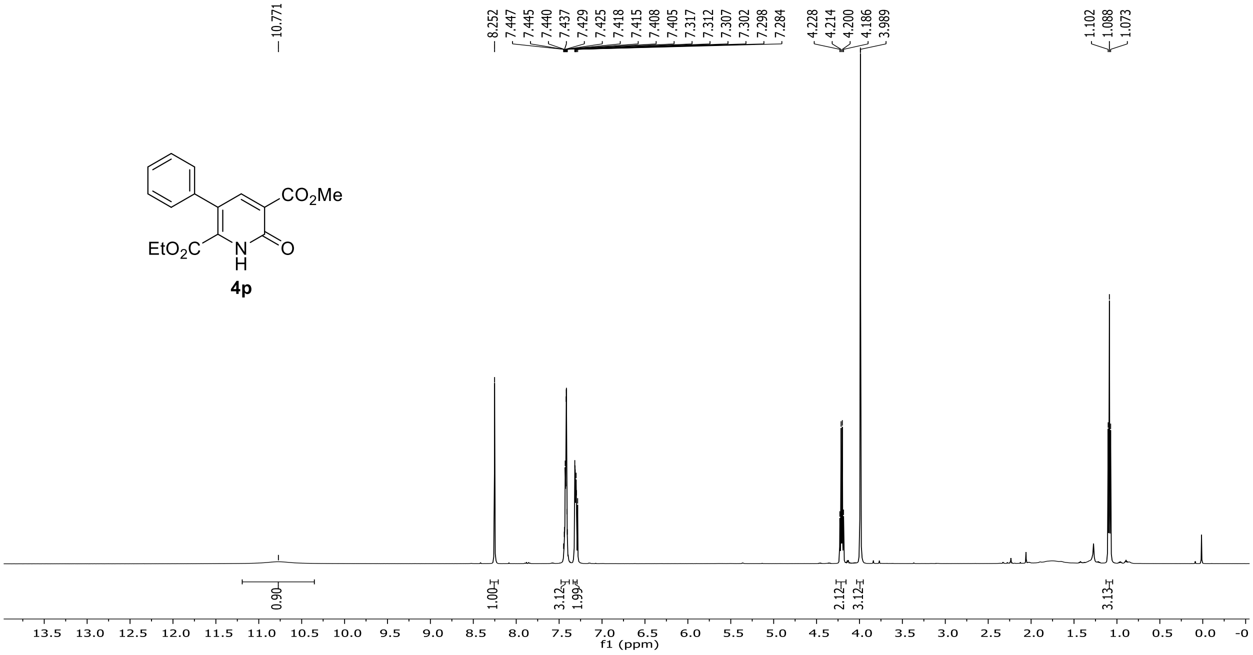

^{13}C-NMR (126 MHz, $CDCl_3$) of compound 4p

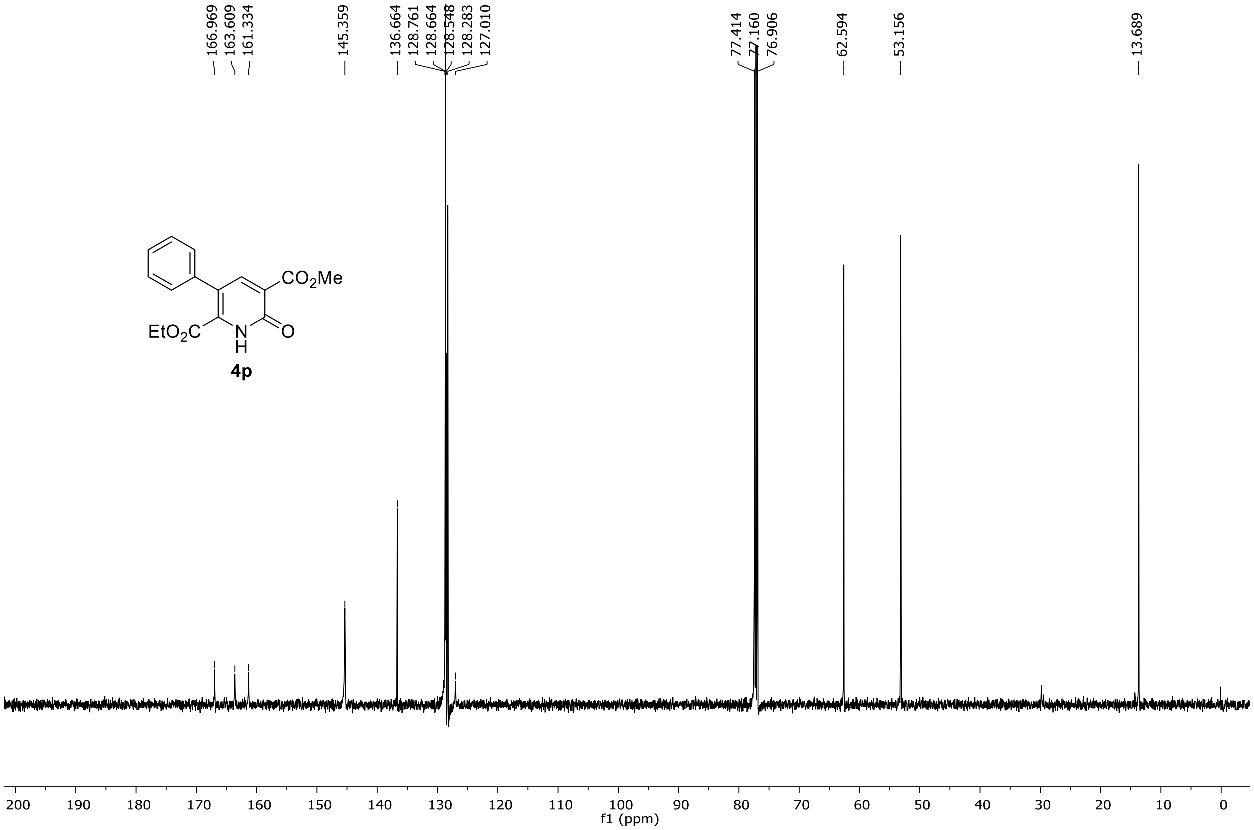

^{1}H-NMR (500 MHz, $CDCl_3$) of compound 4q

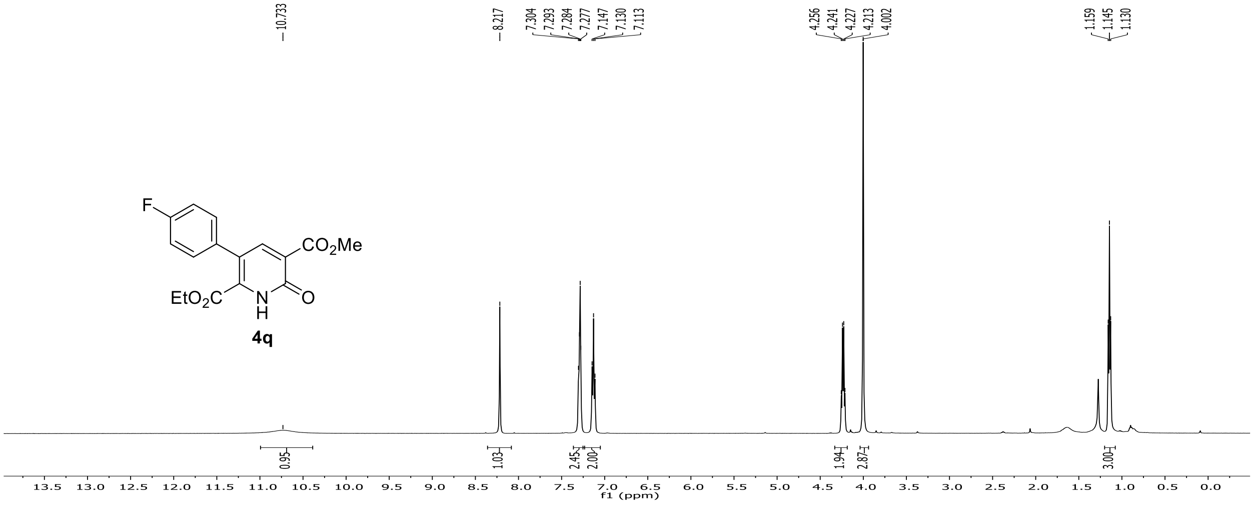

^{13}C-NMR (126 MHz, $CDCl_3$) of compound 4q

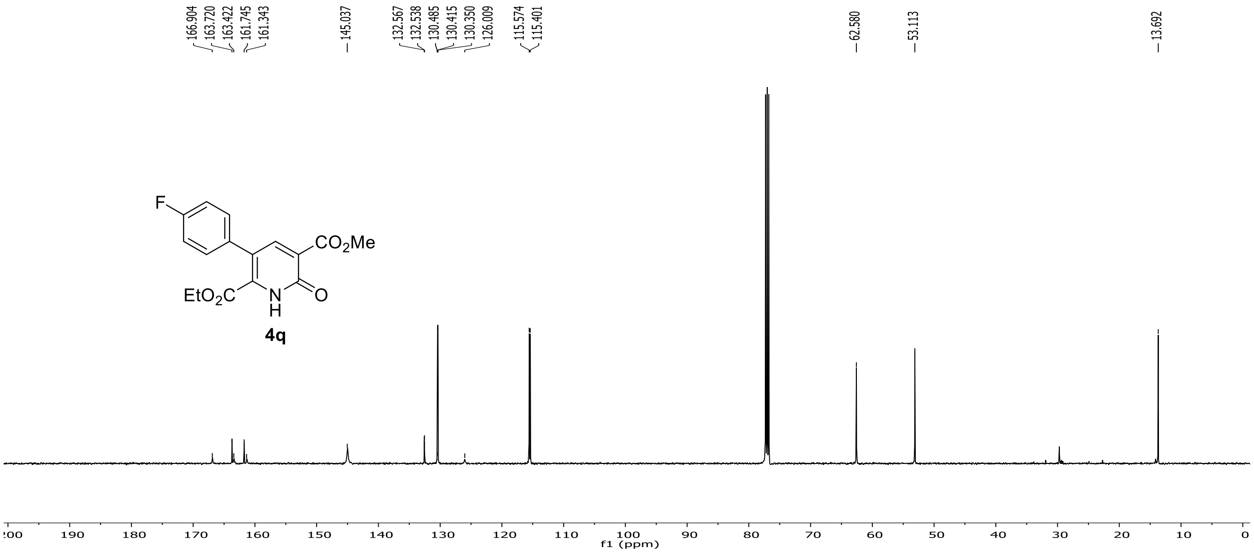

^{19}F-NMR (471 MHz, $CDCl_3$) of compound 4q

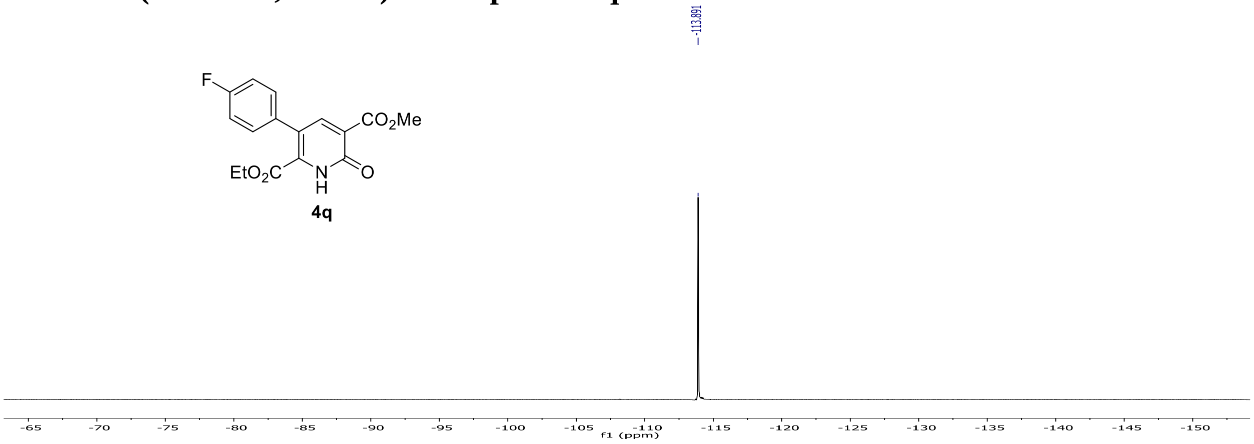

^{1}H-NMR (500 MHz, $CDCl_3$) of compound 4r

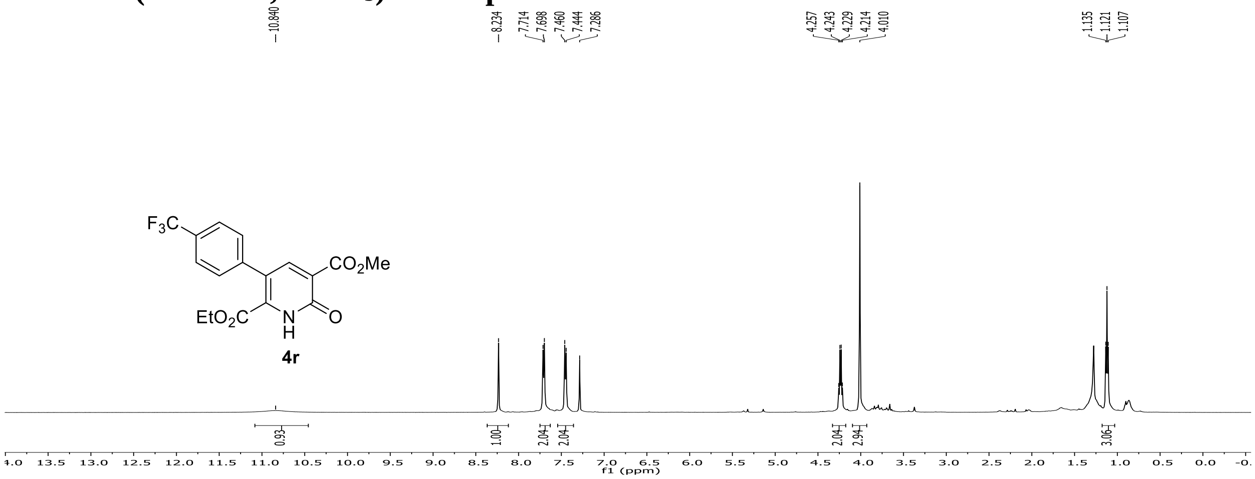

^{13}C-NMR (126 MHz, $CDCl_3$) of compound 4r

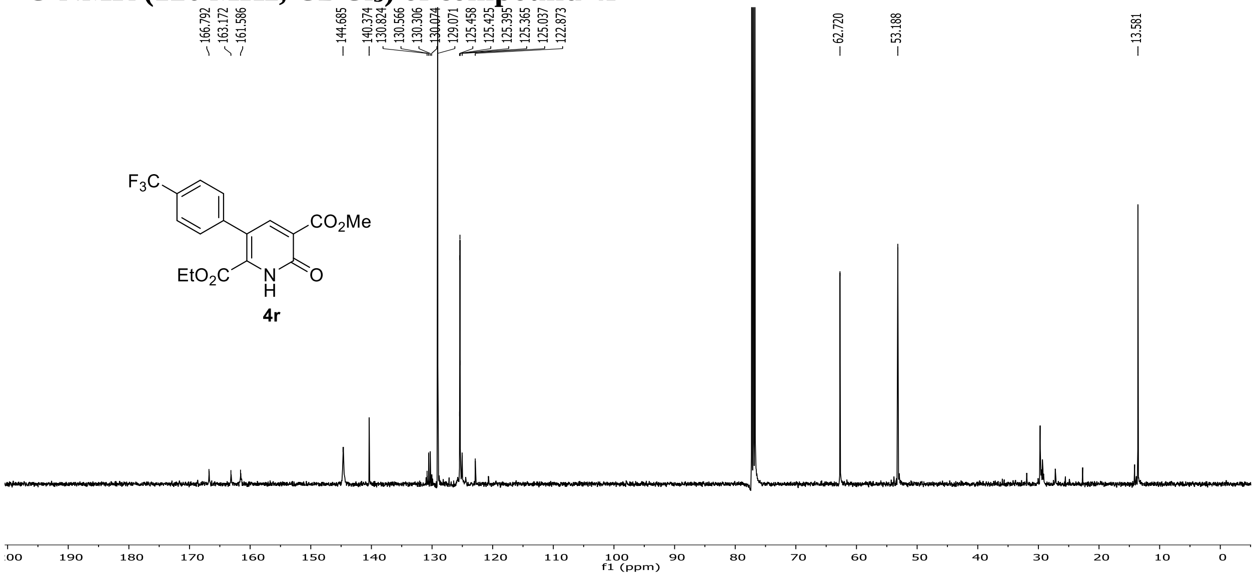

^{19}F-NMR (471 MHz, $CDCl_3$) of compound 4r

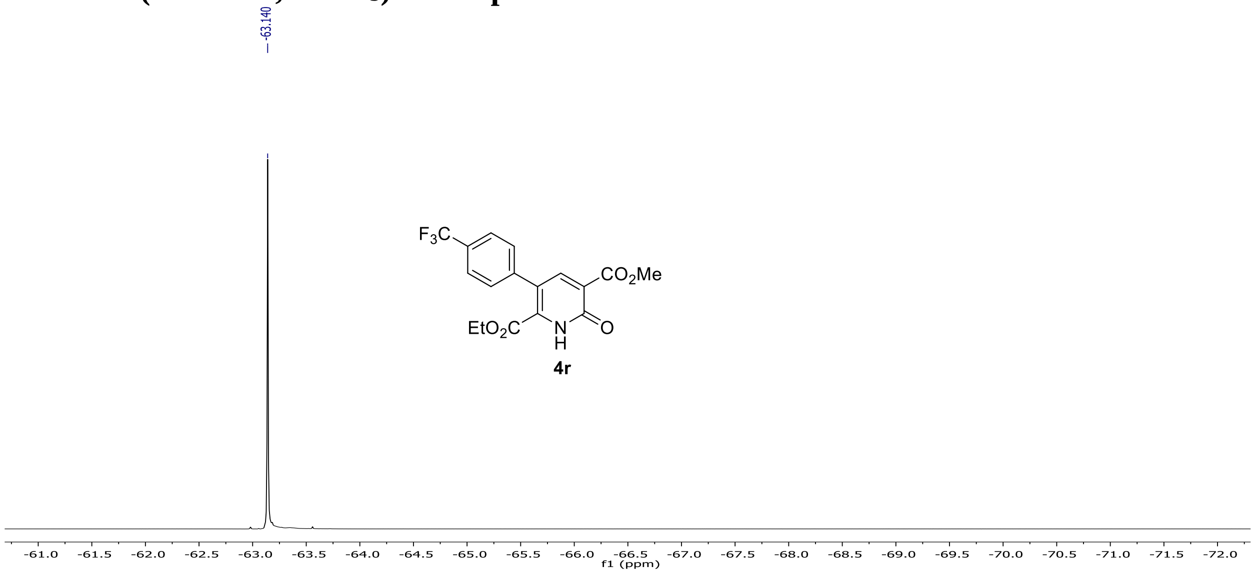

^{1}H-NMR (500 MHz, $CDCl_3$) of compound 4s

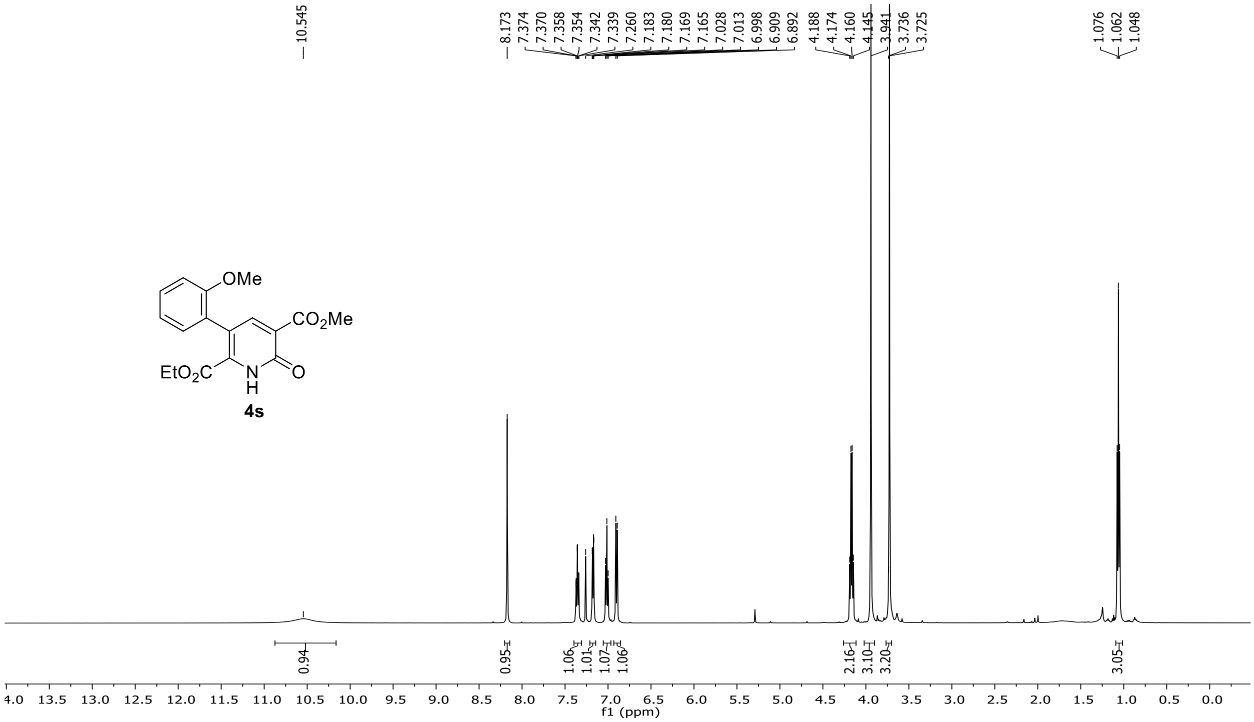

^{13}C-NMR (126 MHz, $CDCl_3$) of compound 4s

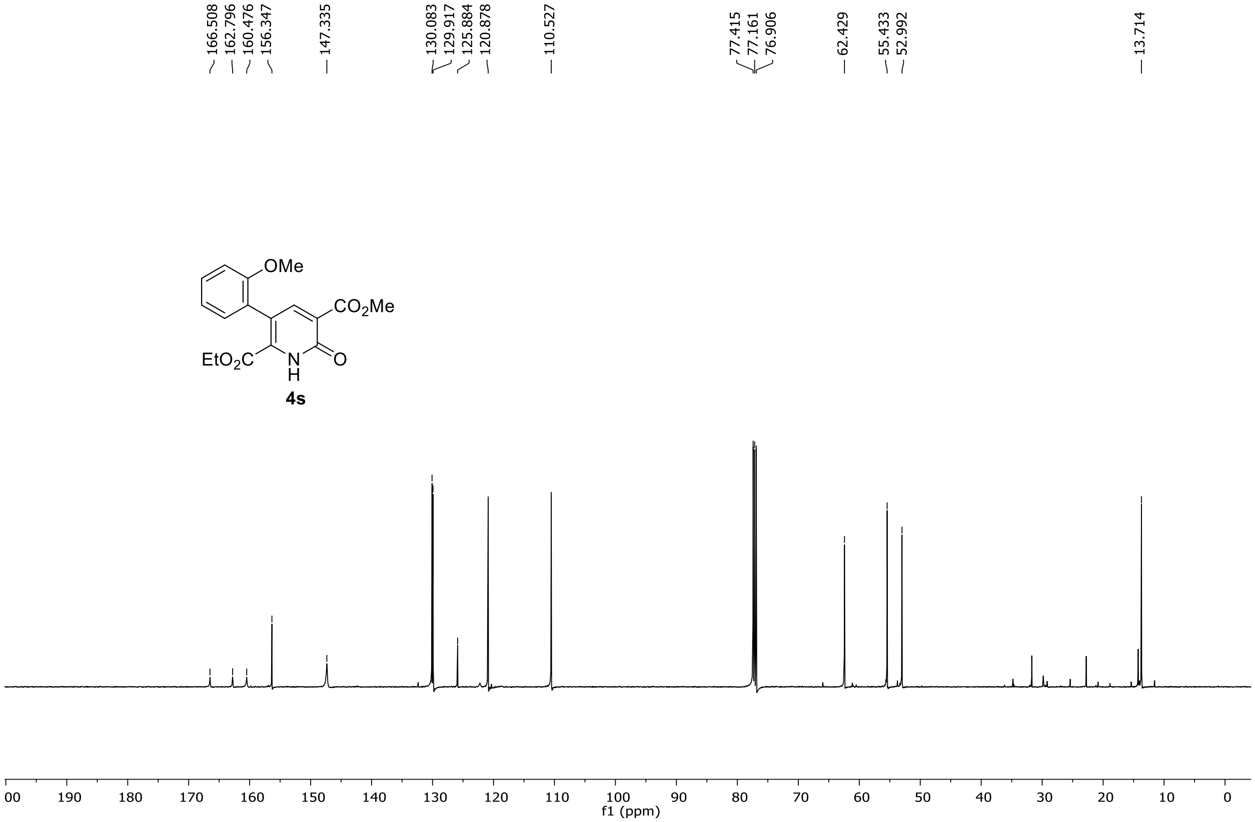

^{1}H-NMR (500 MHz, $CDCl_3$) of compound 4t

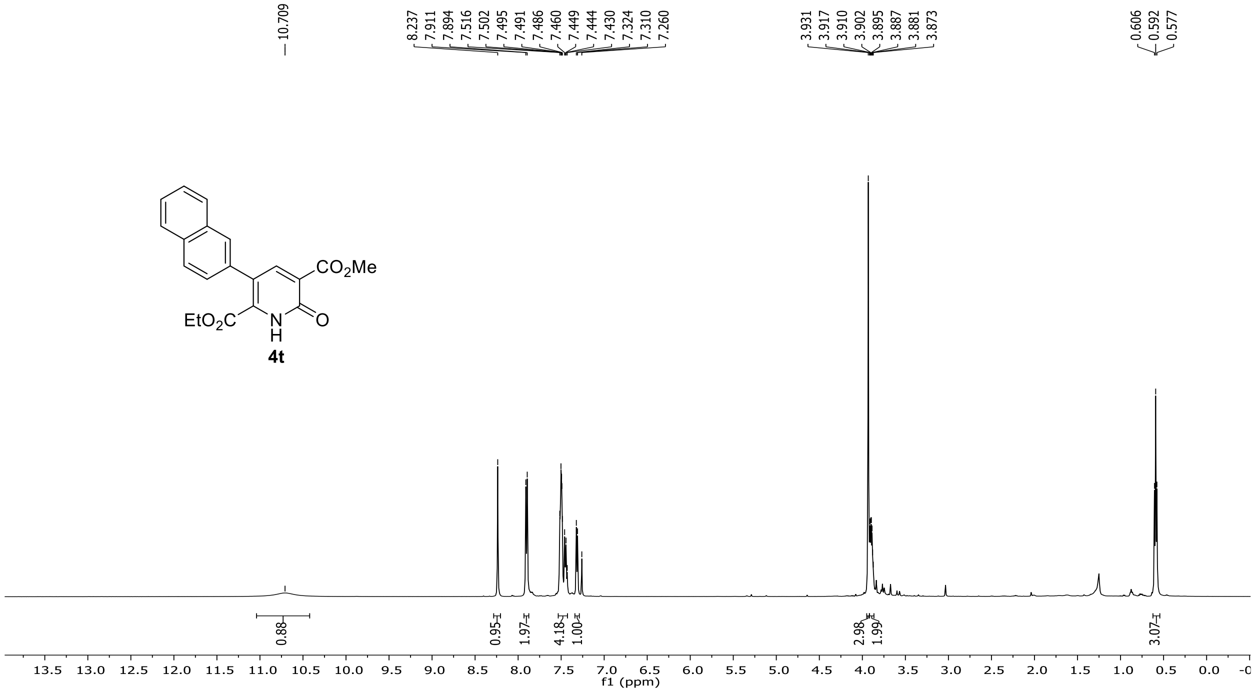

^{13}C-NMR (126 MHz, $CDCl_3$) of compound 4t

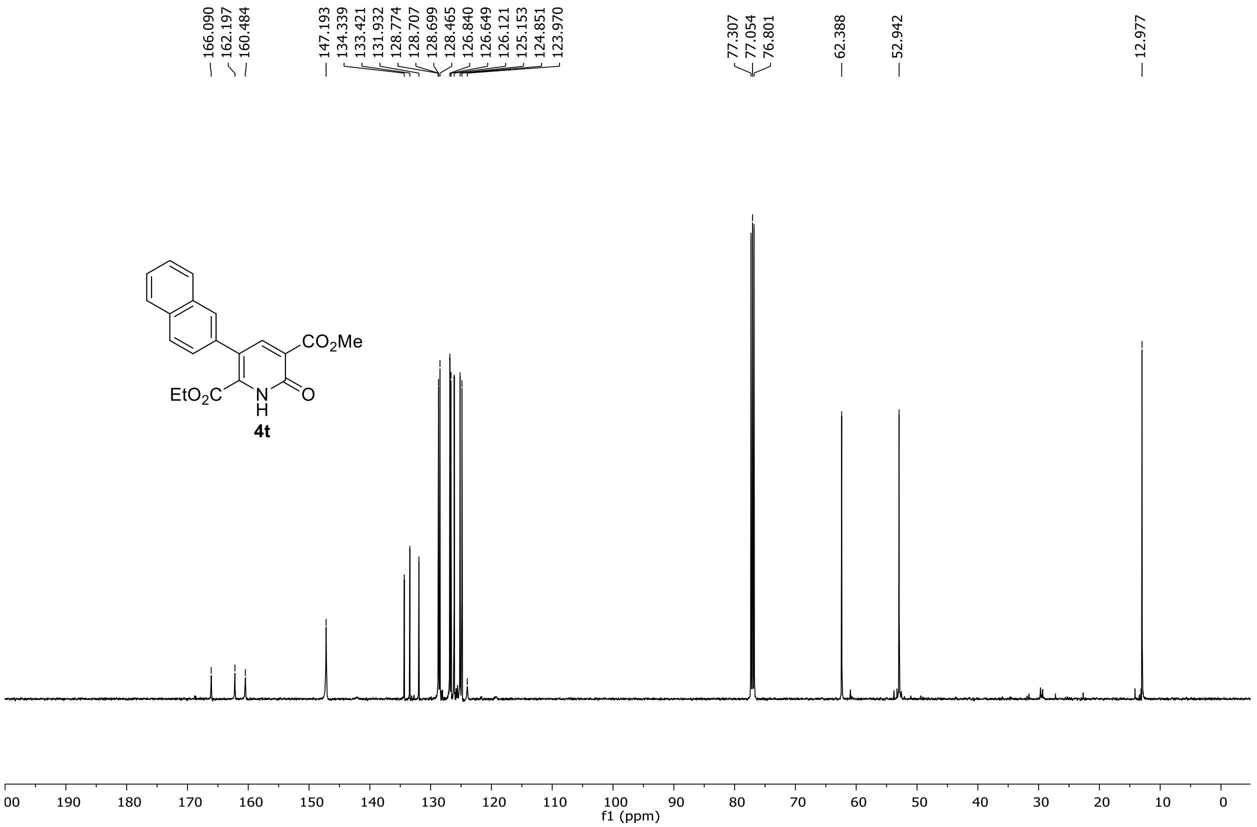

^{1}H-NMR (500 MHz, CDCl3) of compound 4u

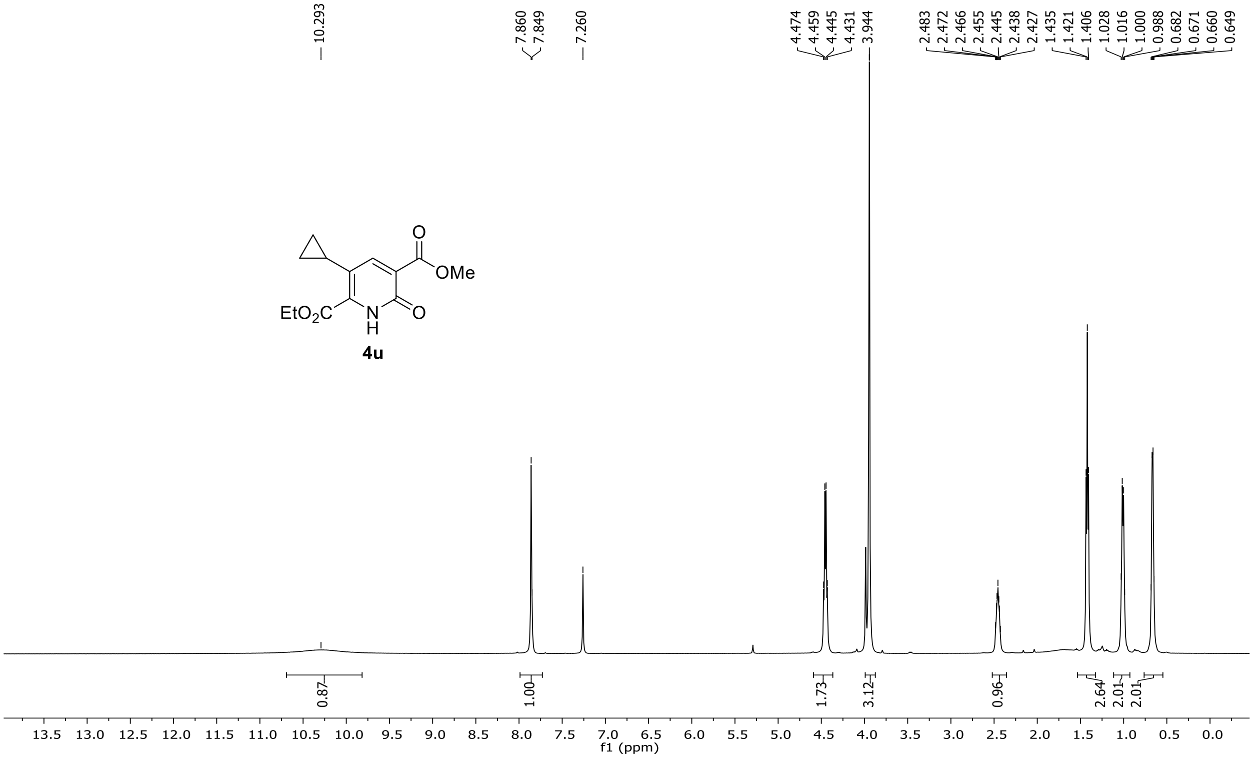

^{13}C-NMR (126 MHz, CDCl3) of compound 4u

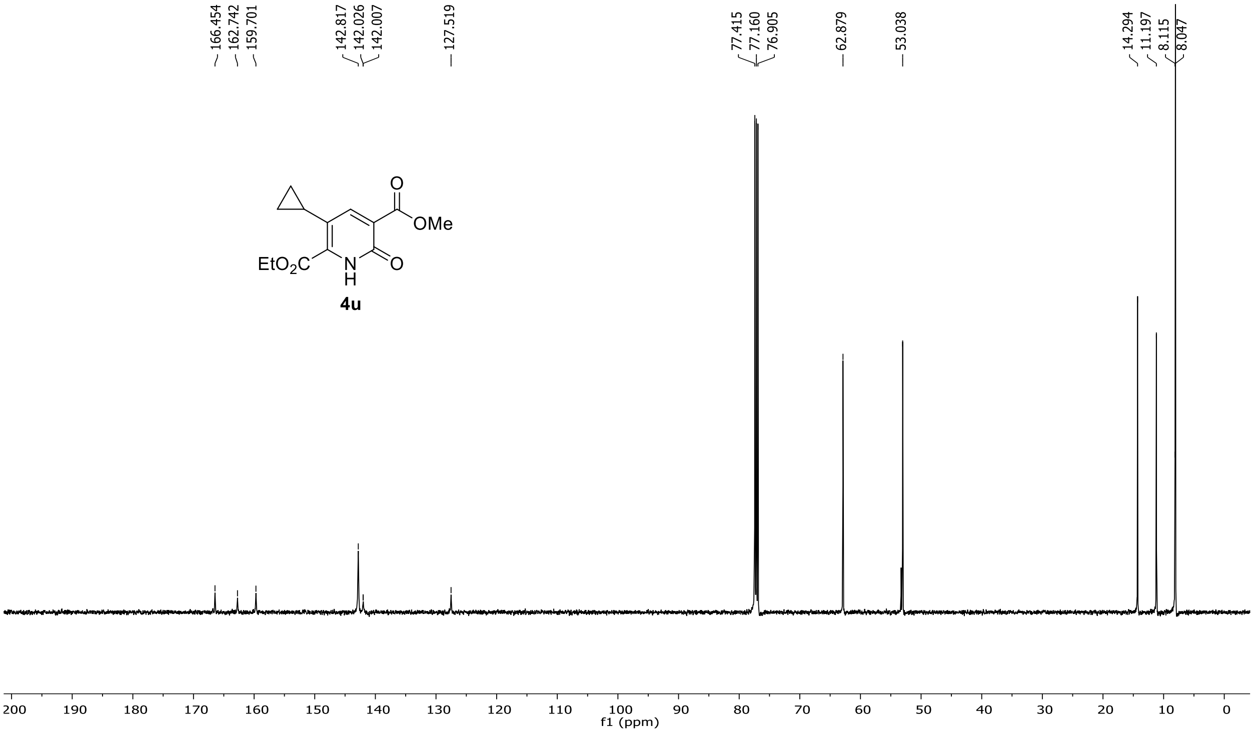

^{1}H-NMR (500 MHz, $CDCl_3$) of compound 9a

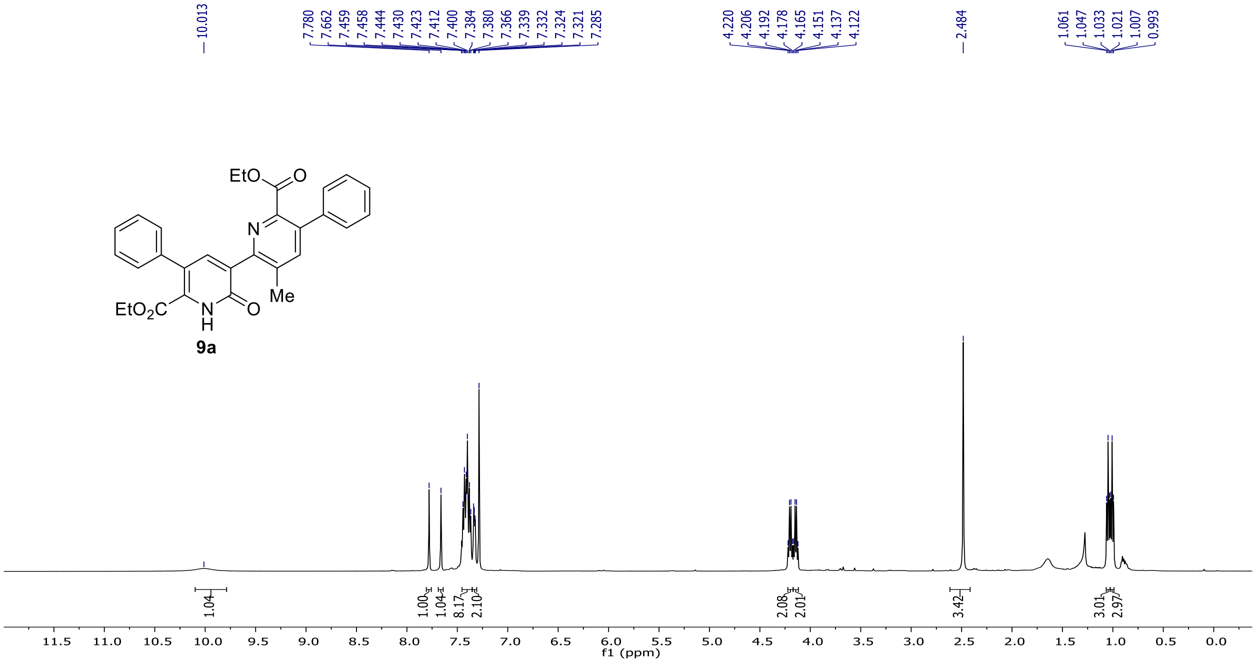

^{13}C-NMR (126 MHz, $CDCl_3$) of compound 9a

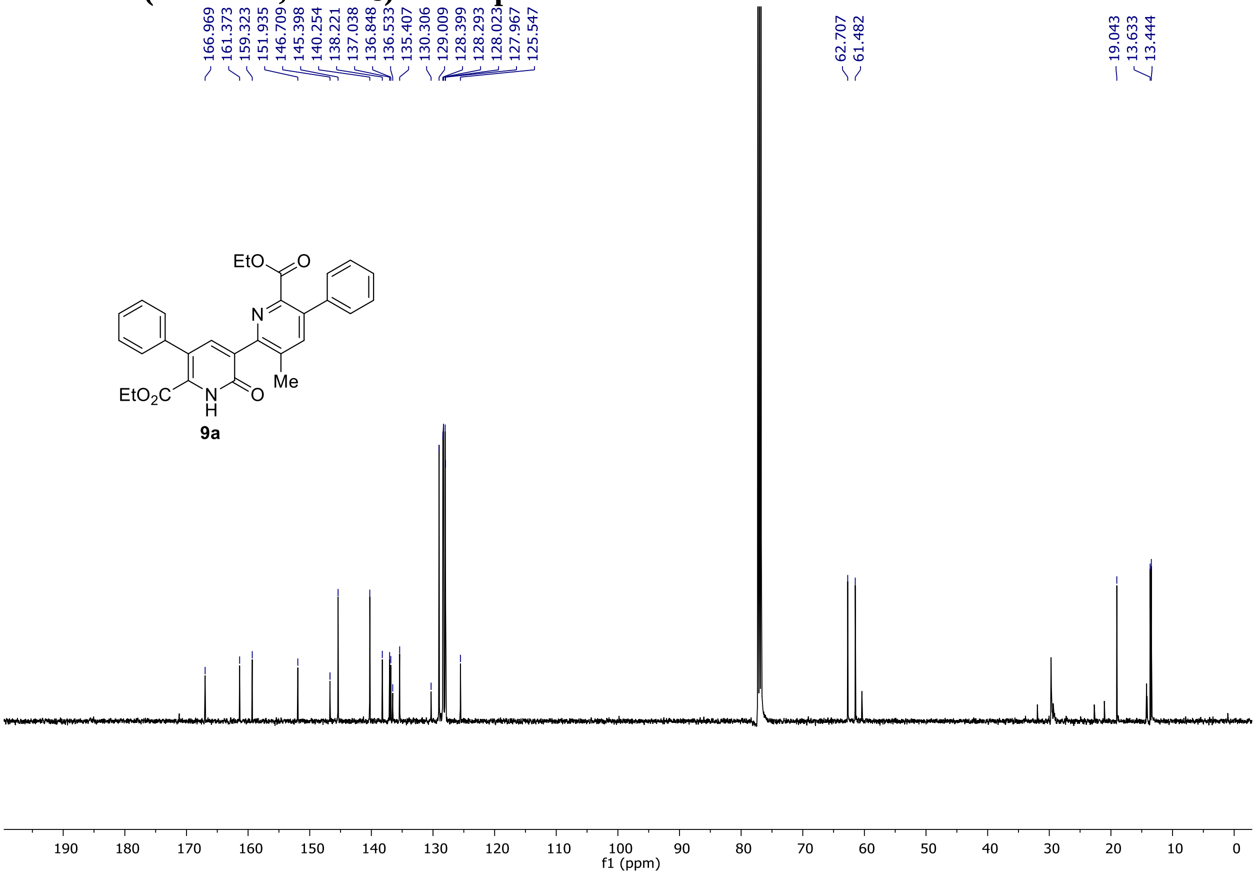

¹H-NMR (500 MHz, $CDCl_3$) of compound 9b

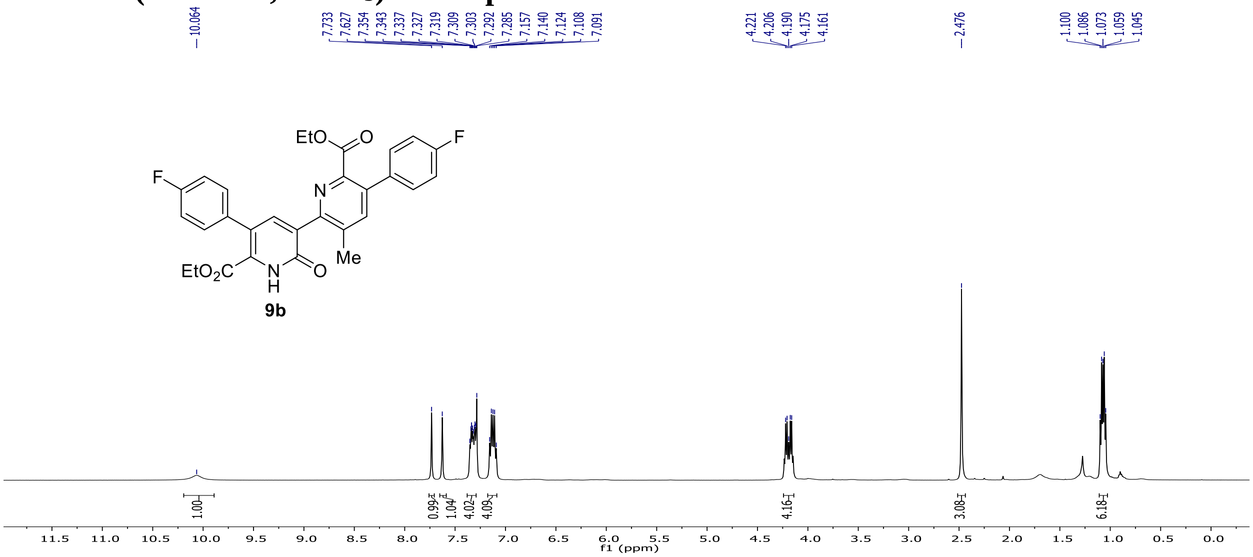

¹³C-NMR (126 MHz, $CDCl_3$) of compound 9b

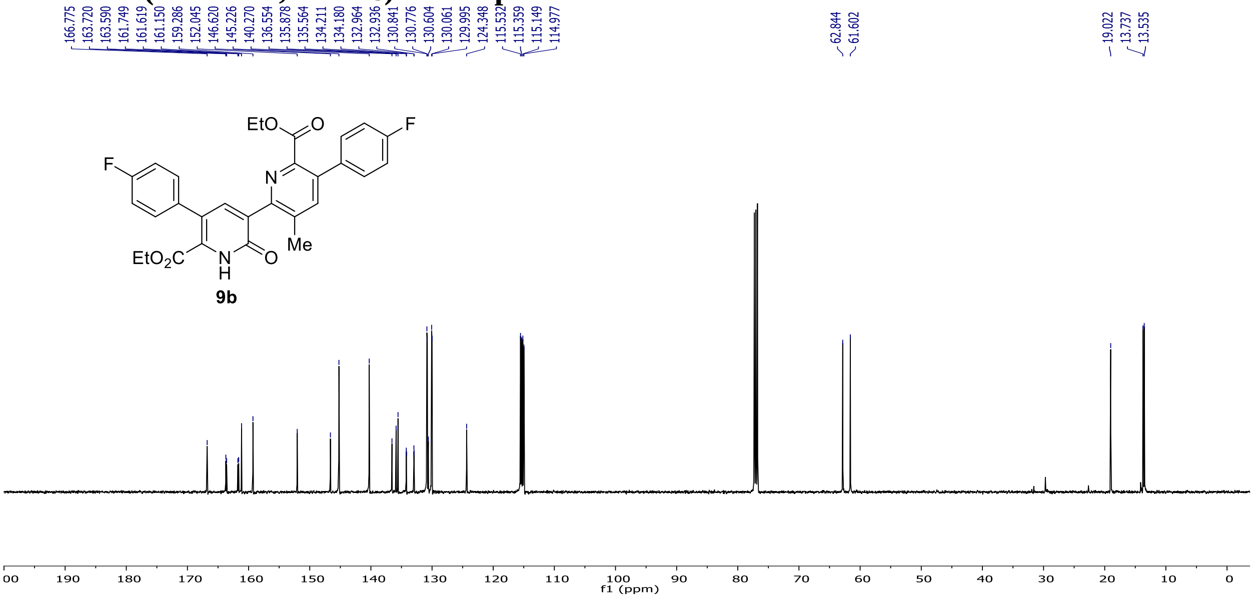

¹⁹F-NMR (471 MHz, $CDCl_3$) of compound 9b

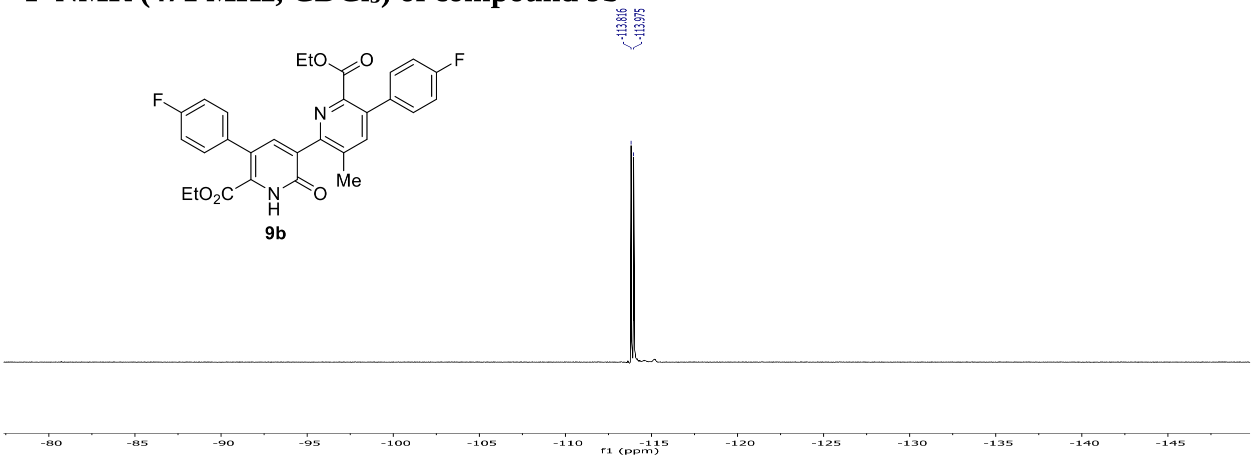

^{1}H-NMR (500 MHz, $CDCl_3$) of compound 9c

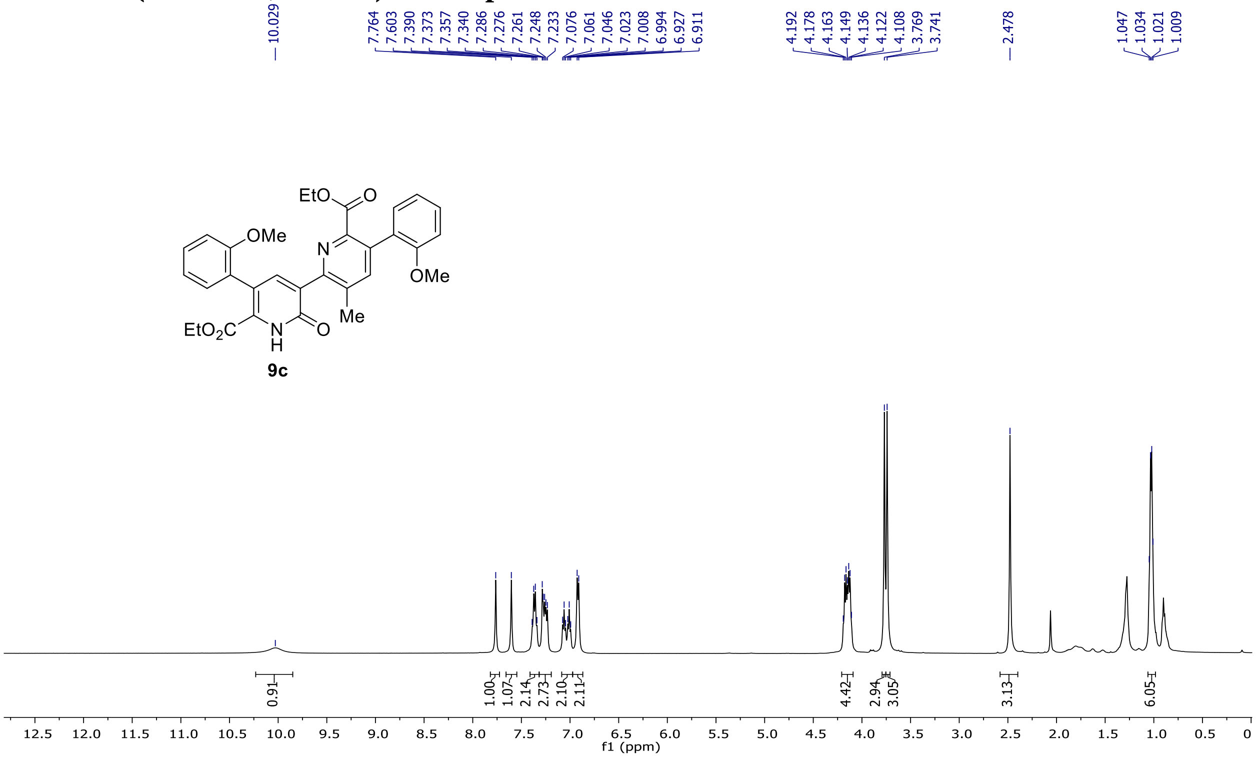

^{13}C-NMR (126 MHz, $CDCl_3$) of compound 9c

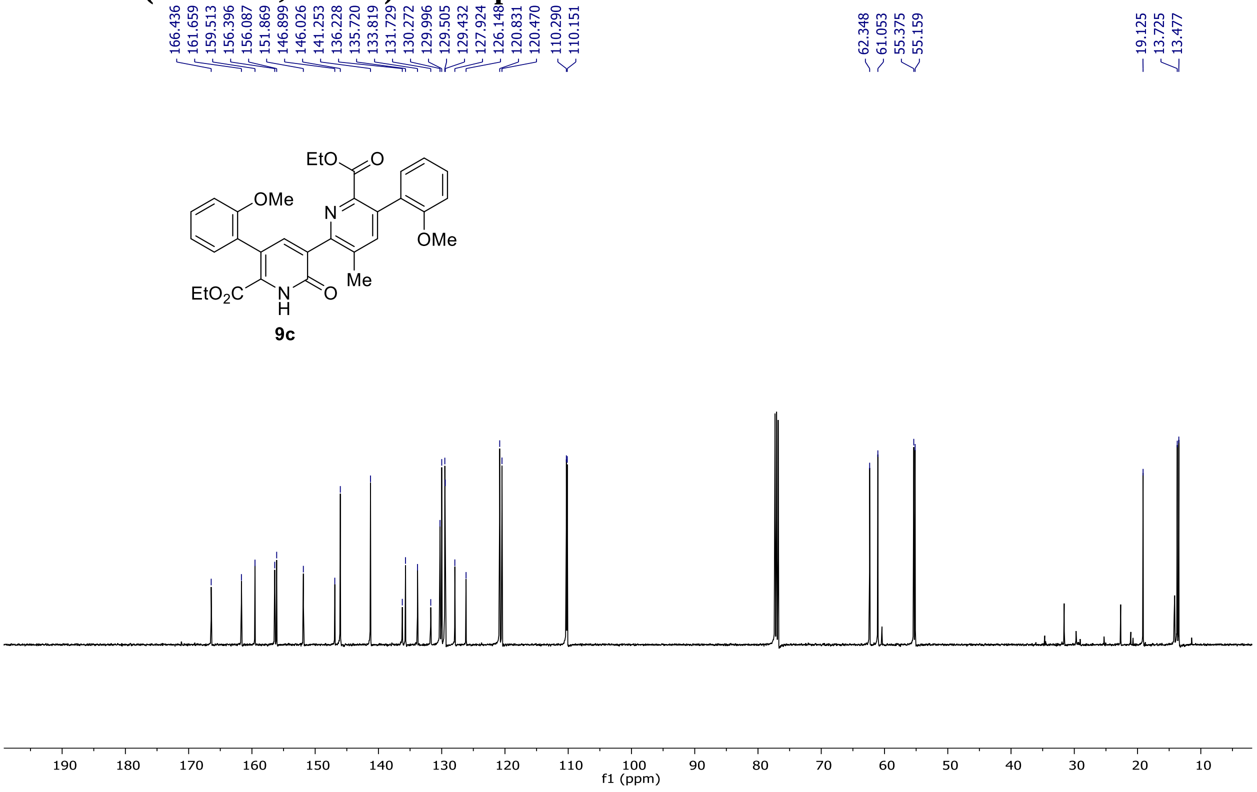

^{1}H-NMR (500 MHz, $CDCl_3$) of compound 9d

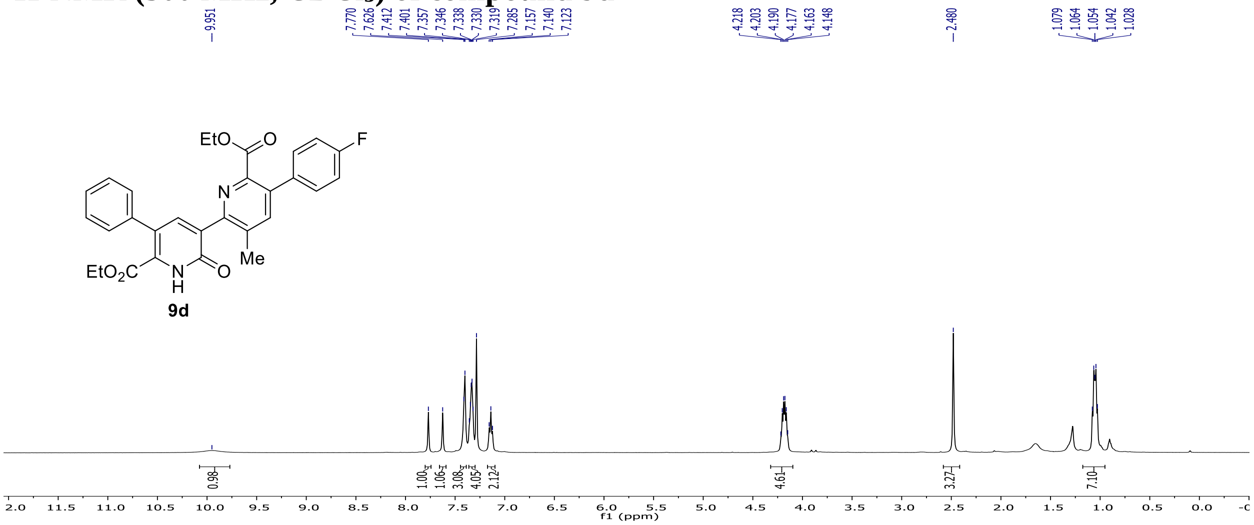

^{13}C-NMR (126 MHz, $CDCl_3$) of compound 9d

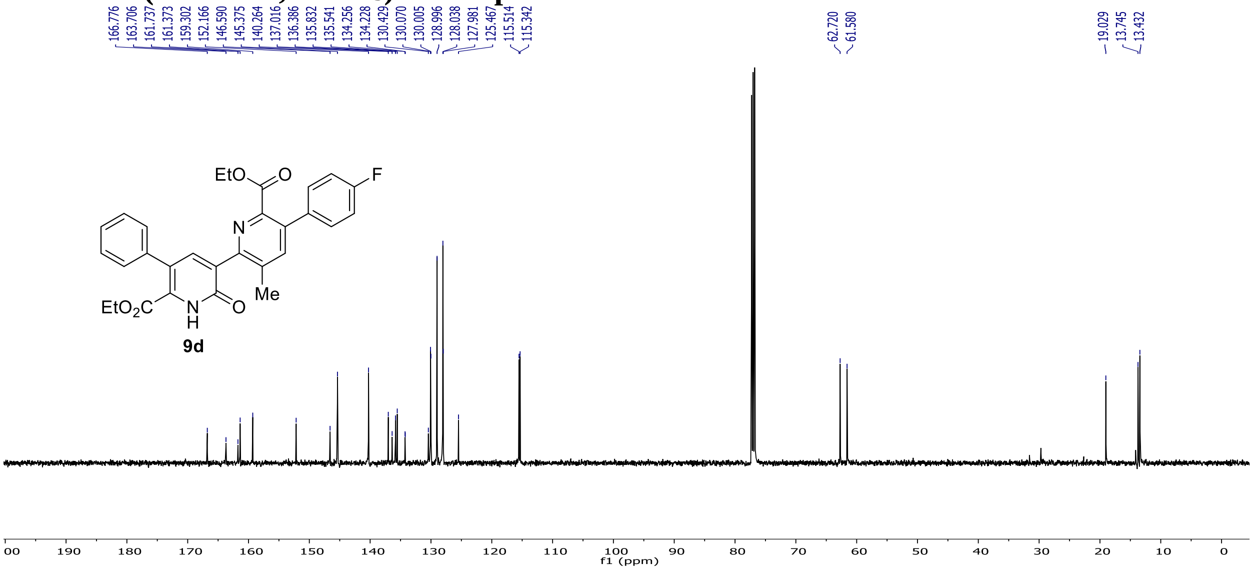

^{19}F-NMR (471 MHz, $CDCl_3$) of compound 9d

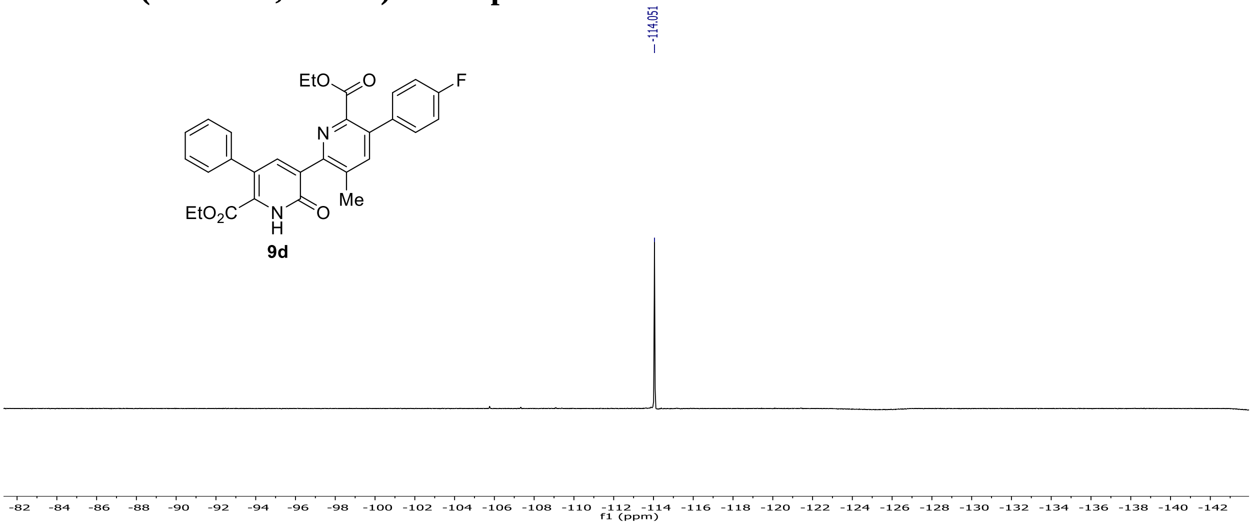

^{1}H-NMR (500 MHz, $CDCl_3$) of compound 9e

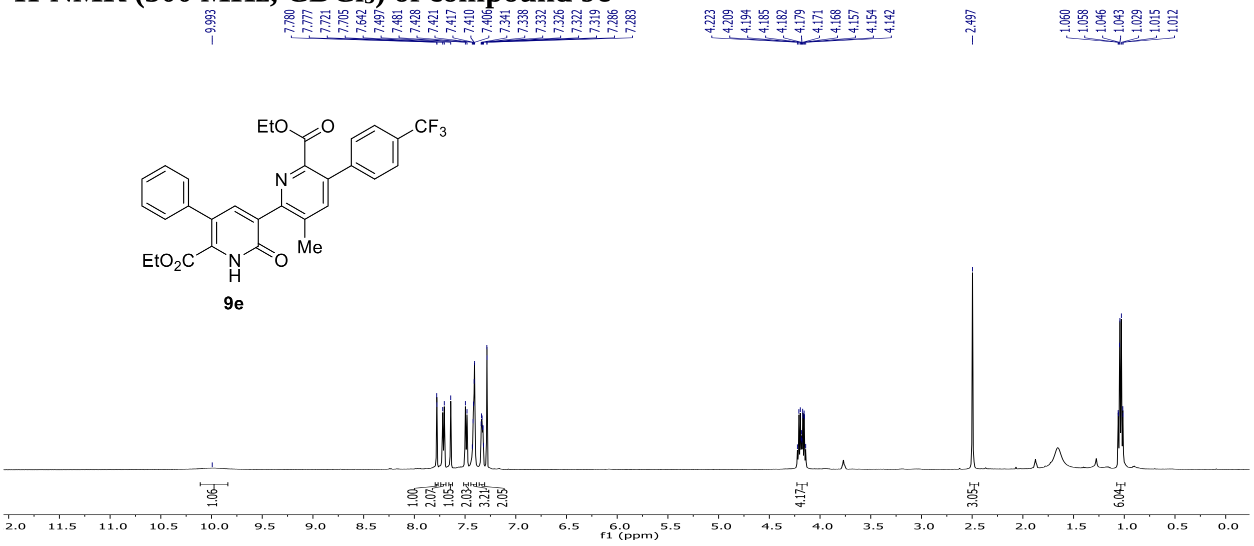

^{13}C-NMR (126 MHz, $CDCl_3$) of compound 9e

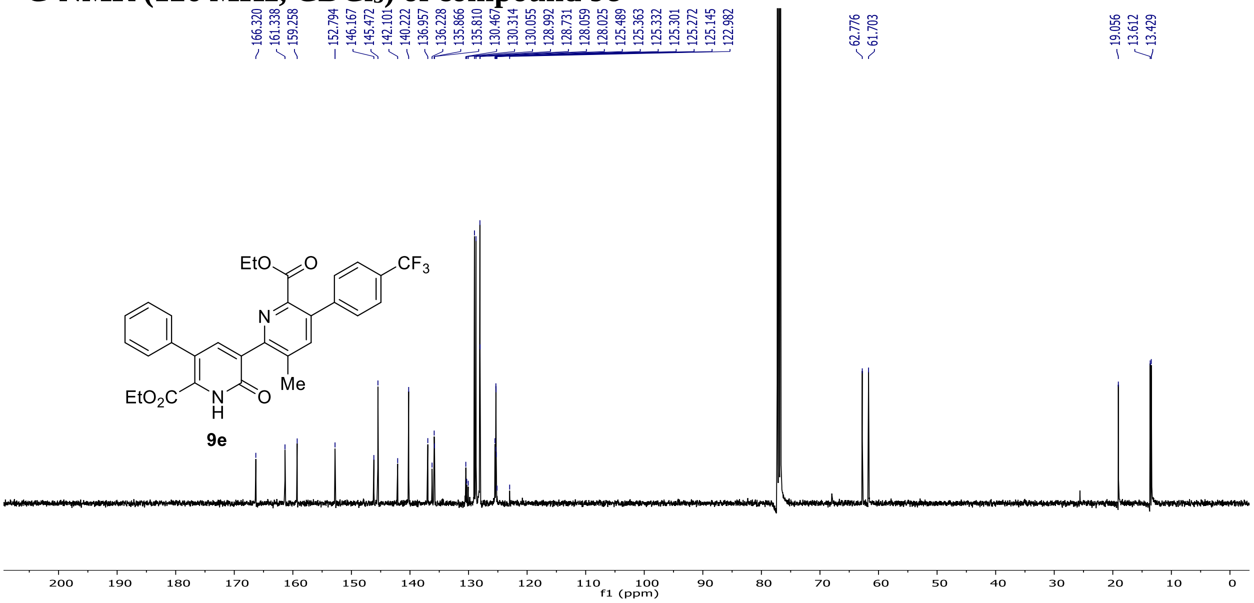

^{19}F-NMR (471 MHz, $CDCl_3$) of compound 9e

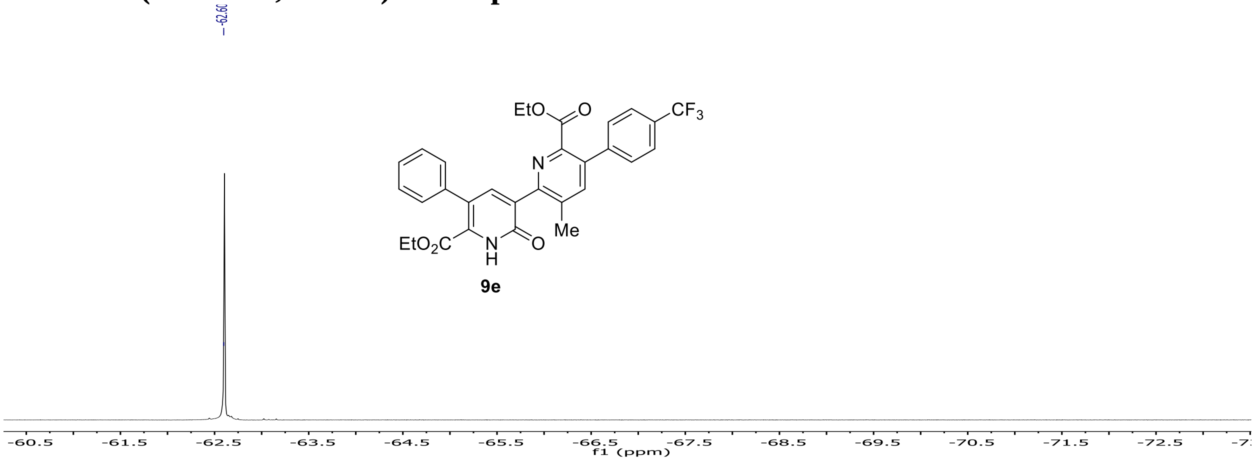

^{1}H-NMR (500 MHz, $CDCl_3$) of compound 9f

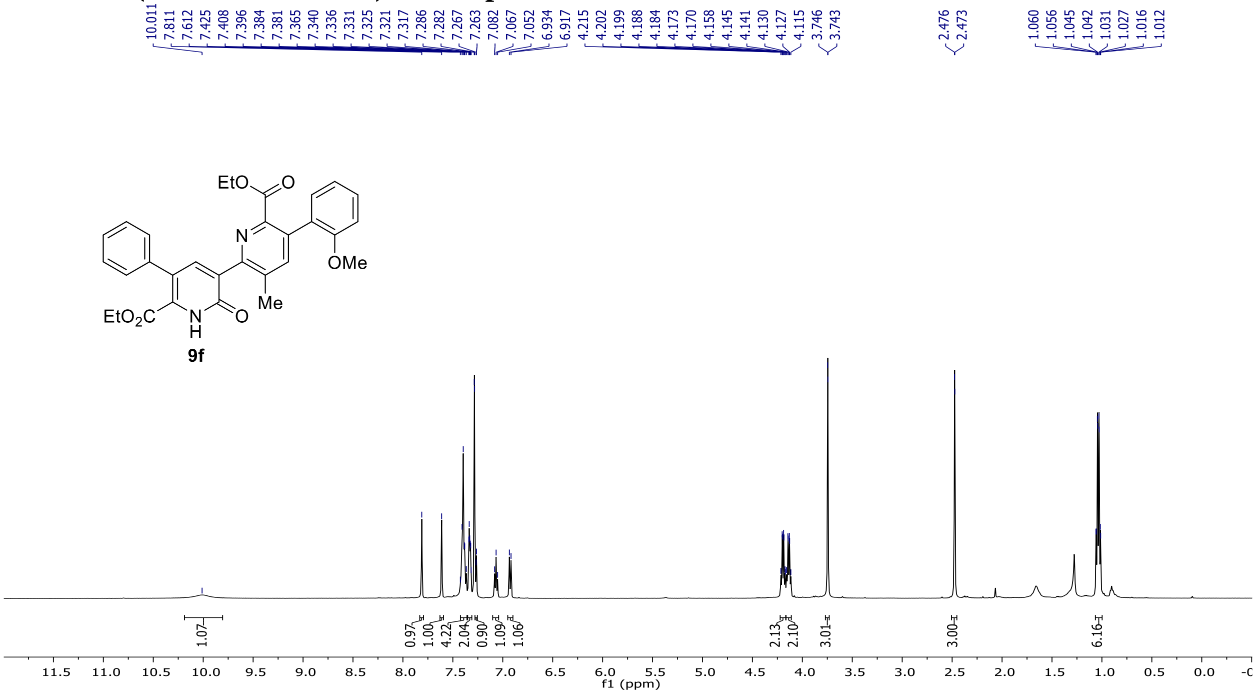

^{13}C-NMR (126 MHz, $CDCl_3$) of compound 9f

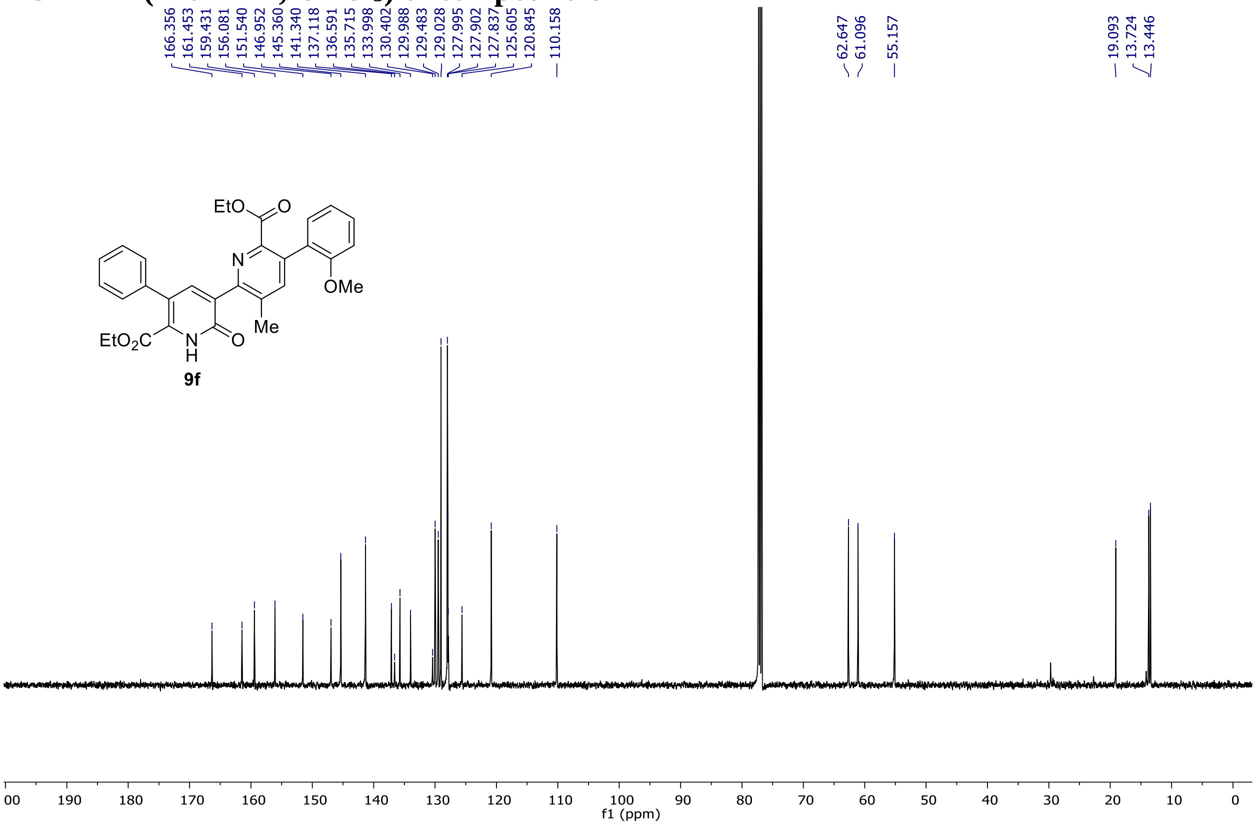

^{1}H-NMR (500 MHz, $CDCl_3$) of compound 9g

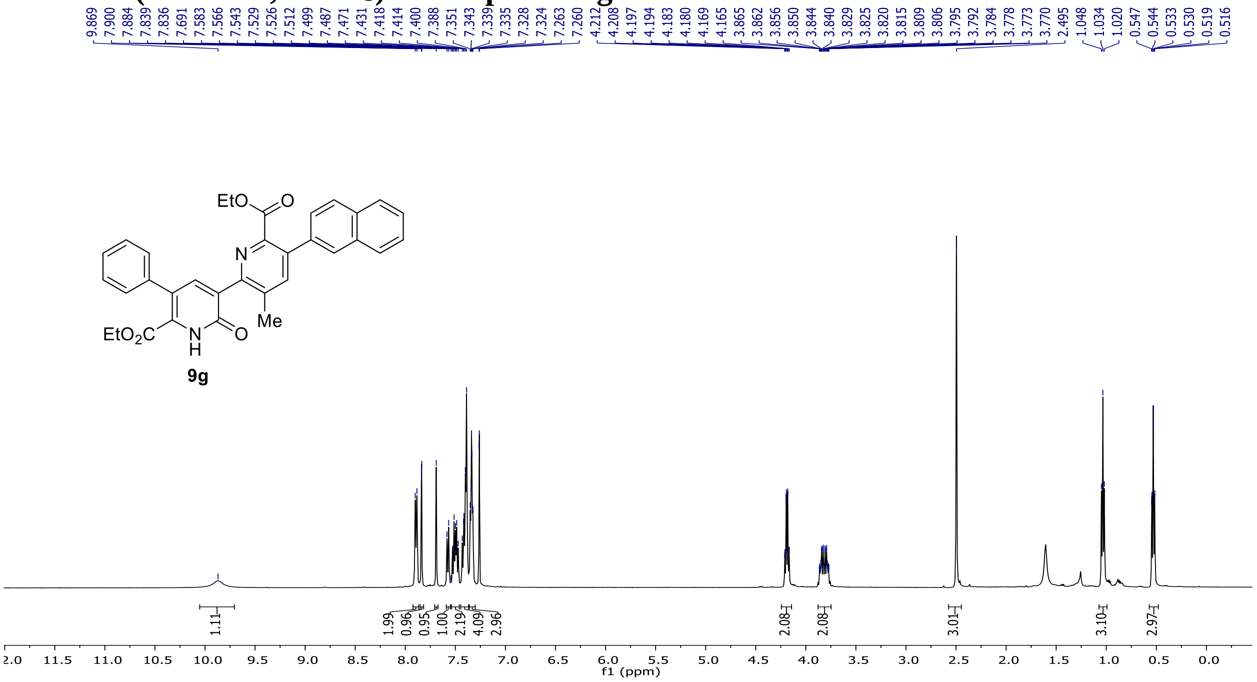

^{13}C-NMR (126 MHz, $CDCl_3$) of compound 9g

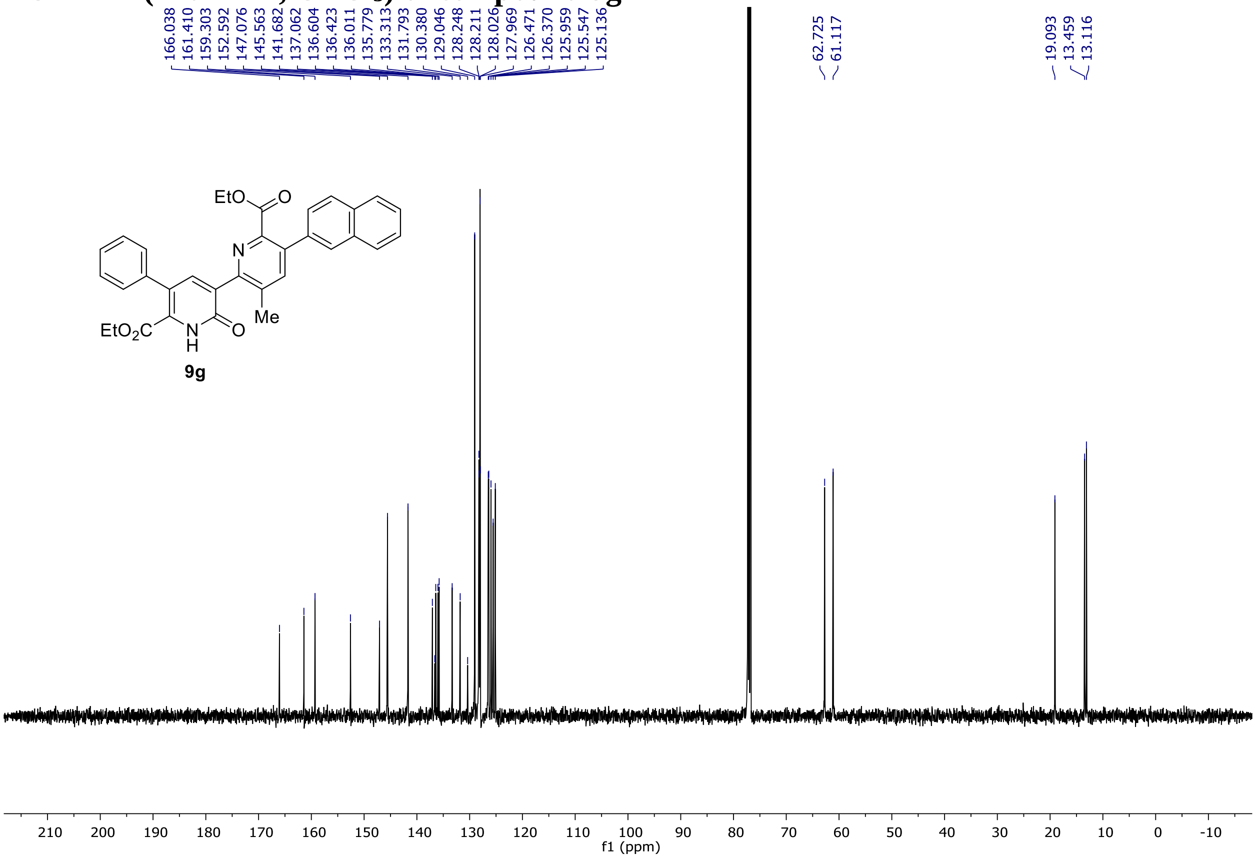

^{1}H-NMR (500 MHz, $CDCl_3$) of compound 9h

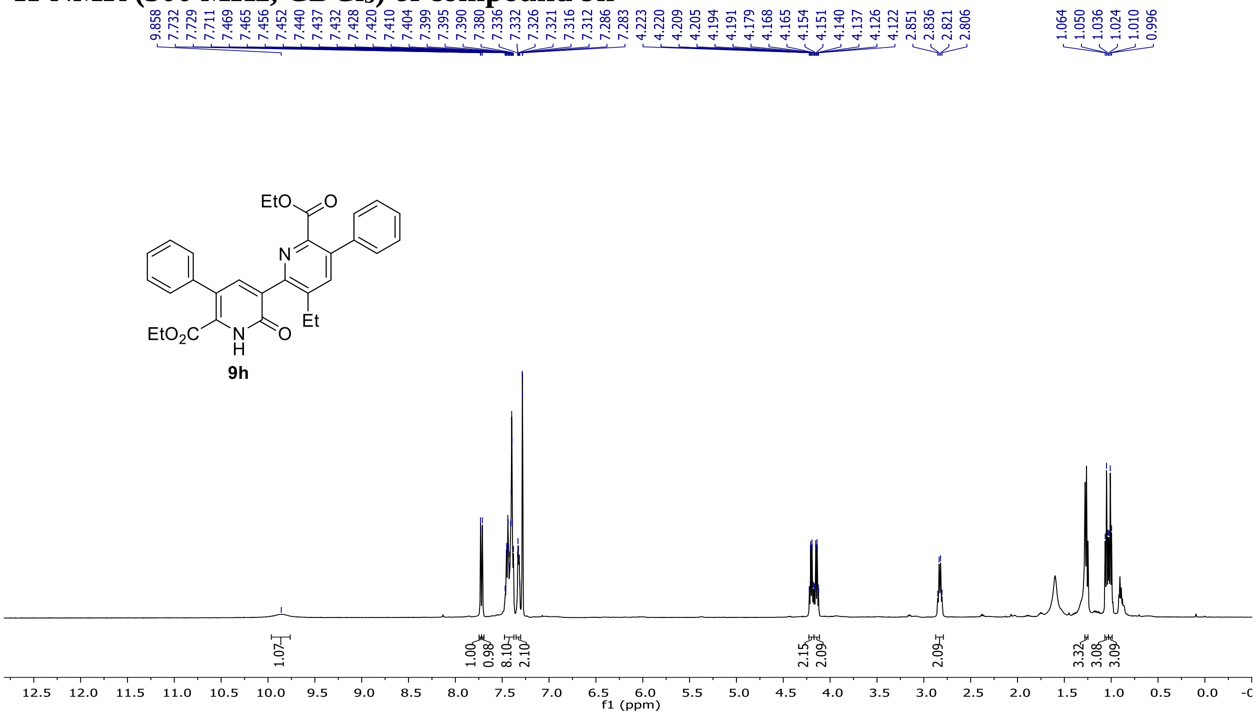

^{13}C-NMR (126 MHz, $CDCl_3$) of compound 9h

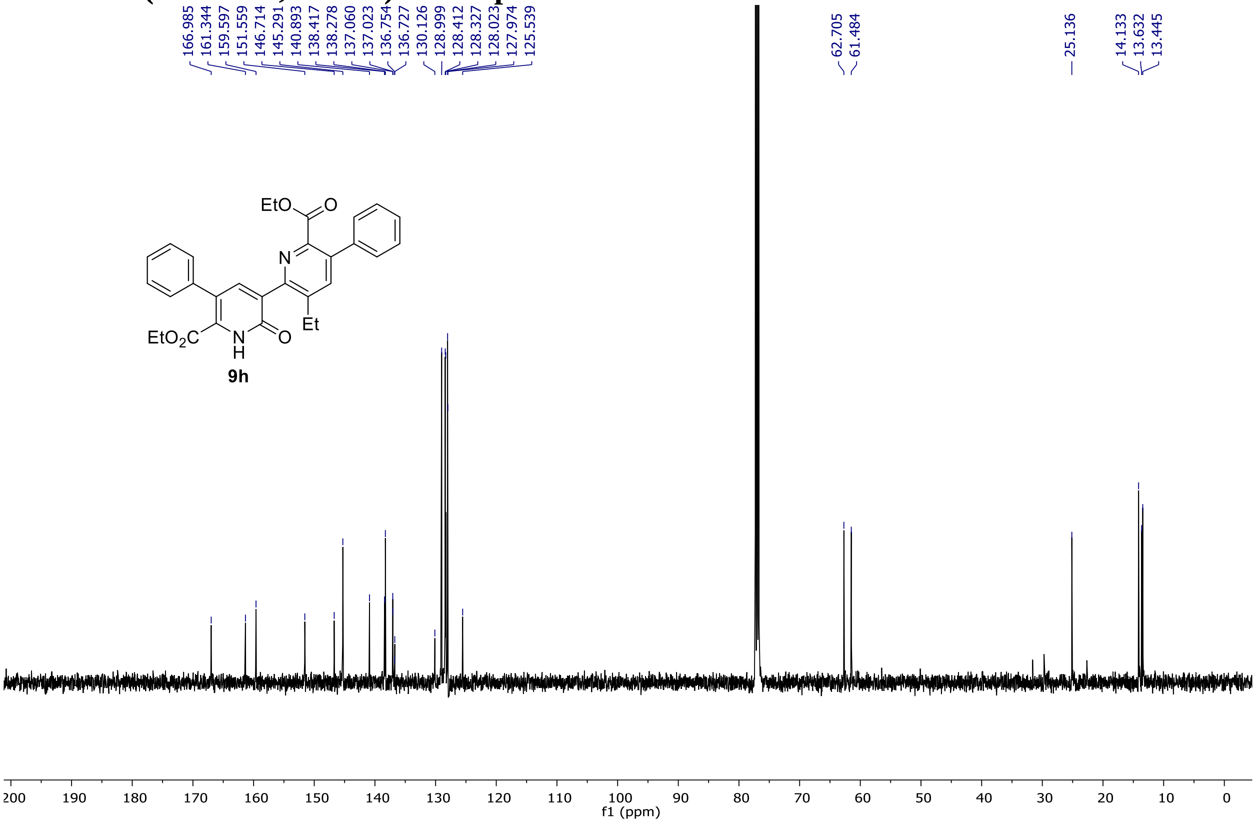

^{1}H-NMR (500 MHz, $CDCl_3$) of compound 9i

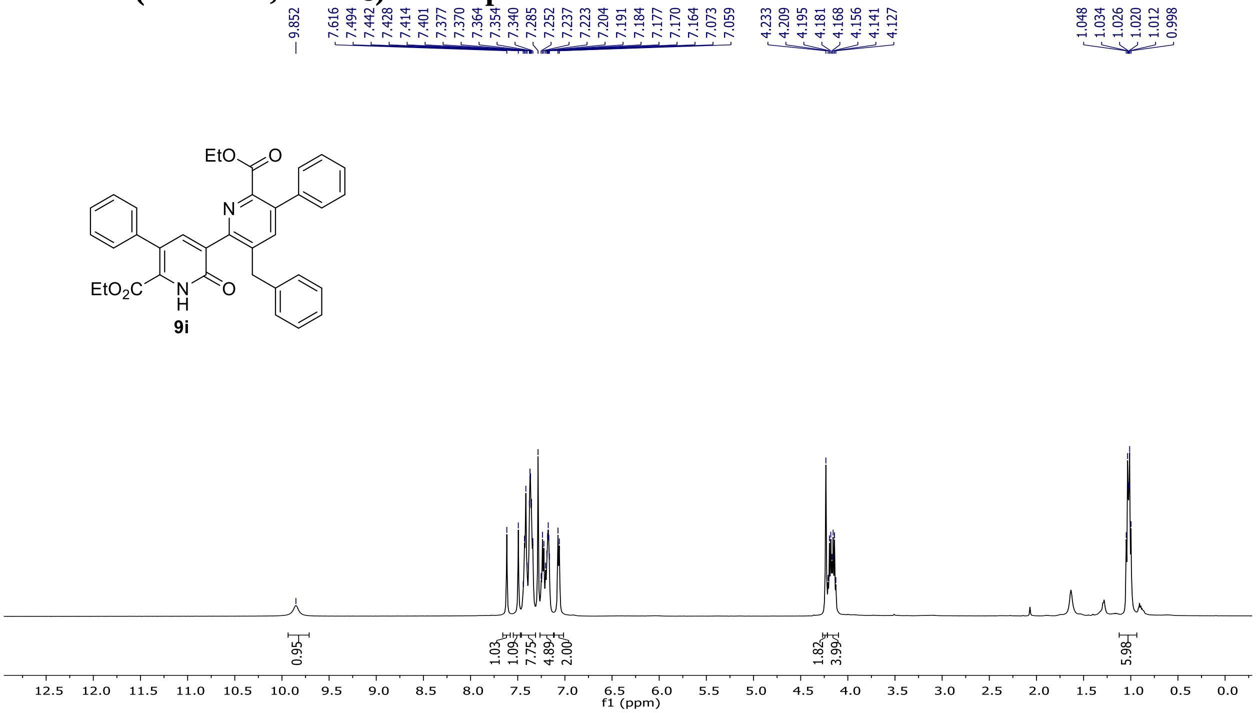

^{13}C-NMR (126 MHz, $CDCl_3$) of compound 9i

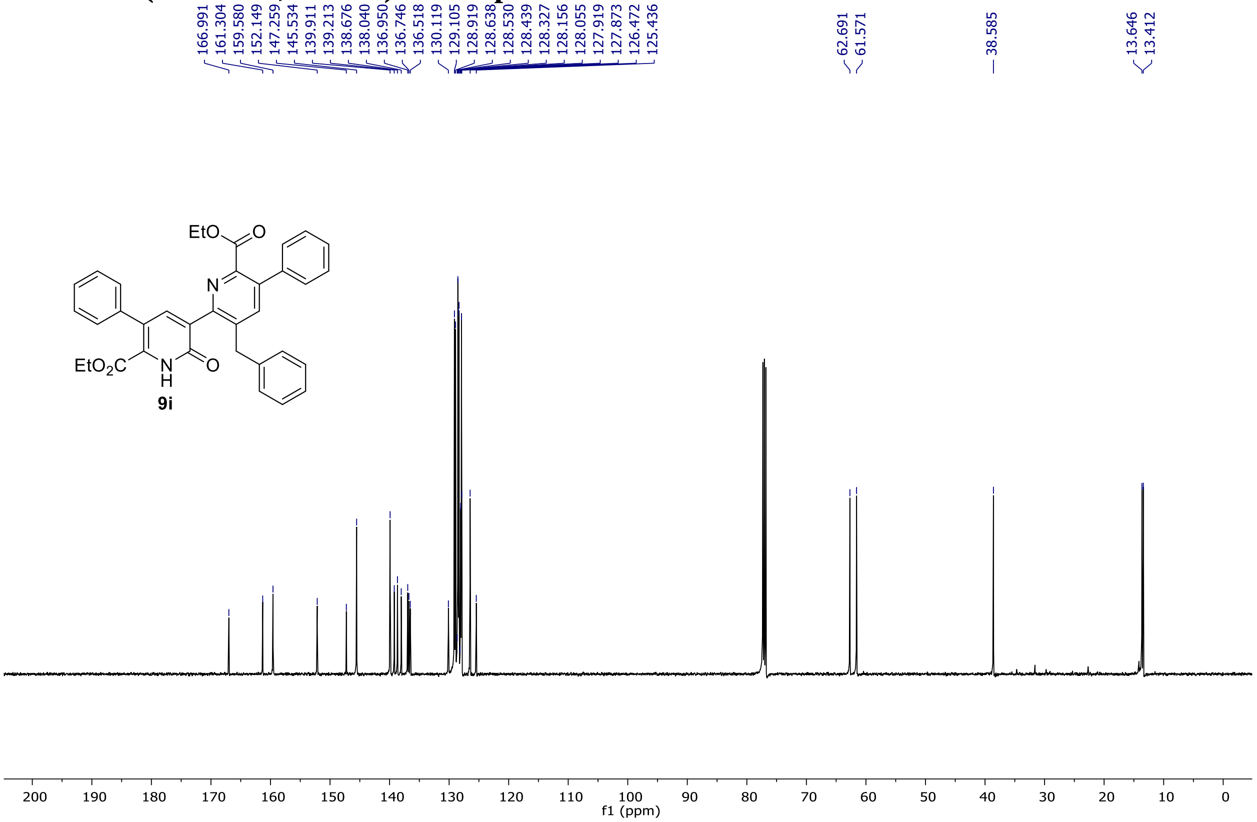

10. References

1. (a) A. Padwa, Y. S. Kulkarni and Z. J. Zhang, *J. Org. Chem*. 1990, **55**, 4144–4153; (b) H. M. L. Davis, P. W. Hougland and W. R. Cantrell Jr., *Synth Commun*. 1992, **22**, 971–978; (c) H. M. L. Davies, B. H. Hu, E. Saikali and P. R. Bruzinski, *J. Org. Chem.*1994, **59**, 4535–4541; d) M. P. Doyle, M. Yan and W. H. Hu, *J. Am. Chem. Soc.* 2003, **125**, 46924693; (e) V. V. Pagar, A. M. Jadhav and R. S. Liu, *J. Am. Chem. Soc.* 2011, **133**, 20728–2073; (f) H. F. Zheng, K. Y. Dong, D. Wherritt, H. Arman and M. P. Doyle, *Angew. Chem. Int. Ed.* **2020**, *59*, 13613–13617.

2. L. De Angelis, H. Zheng, M.T. Perz, H. Arman and M.P. Doyle, *Org. Lett.* 2021, **23**, 6542–6546.

3. CrysAlisPro 1.171.40.63a (Rigaku Oxford Diffraction, 2019).

4. SCALE3 ABSPACK - An Oxford Diffraction program (1.0.4,gui:1.0.3) (C) 2005 Oxford Diffraction Ltd.

5. O. V. Dolomanov, L. J. Bourhis, R. J. Gildea, J. A. K. Howard and H. Puschmann, *J. Appl. Cryst.* 2009, **42**, 339.

6. G. M. Sheldrick, *Acta Cryst.* 2015, **A71**, 3.

7. G. M. Sheldrick, *Acta Cryst.* 2008, **A64**, 112.

8. Gaussian 16, Revision A.03, M. J. Frisch, G. W. Trucks, H. B. Schlegel, G. E. Scuseria, M. A. Robb, J. R. Cheeseman, G. Scalmani, V. Barone, G. A. Petersson, H. Nakatsuji, X. Li, M. Caricato, A. V. Marenich, J. Bloino, B. G. Janesko, R. Gomperts, B. Mennucci, H. P. Hratchian, J. V. Ortiz, A. F. Izmaylov, J. L. Sonnenberg, D.Williams-Young, F. Ding, F. Lipparini, F. Egidi, J. Goings, B. Peng, A. Petrone, T. Henderson, D. Ranasinghe, V. G. Zakrzewski, J. Gao, N. Rega, G. Zheng, W. Liang, M. Hada, M. Ehara, K. Toyota, R. Fukuda, J. Hasegawa, M. Ishida, T. Nakajima, Y. Honda, O. Kitao, H. Nakai, T. Vreven, K. Throssell, J. A. Montgomery, Jr., J. E. Peralta, F. Ogliaro, M. J. Bearpark, J. J. Heyd, E. N. Brothers, K. N. Kudin, V. N. Staroverov, T. A. Keith, R. Kobayashi, J. Normand, K. Raghavachari, A. P. Rendell, J. C. Burant, S. S. Iyengar, J. Tomasi, M. Cossi, J. M. Millam, M. Klene, C. Adamo, R. Cammi, J. W. Ochterski, R. L. Martin, K. Morokuma, O. Farkas, J. B. Foresman and D. J. Fox, Gaussian, Inc., Wallingford CT, 2016.

9. S. Grimme, C. Bannwarth and P. Shushkov, *J Chem. Theory Comput.* 2017, **13**, 1989–2009.

10. CYLview, 1.0b, C. Y. Legault, Université de Sherbrooke, 2009 (http://www.cylview.org).

11. W. Humphrey, A. Dalke and K. Schulten, *J. Mol. Graph.* 1996, **14**, 33–38.

12. Chemcraft - graphical software for visualization of quantum chemistry computations. https://www.chemcraftprog.com

13. G. Luchini, J. V. Alegre-Requena, Y. Guan, I. Funes-Ardoiz and R. S. Paton, GoodVibes: GoodVibes v3.0.1 (2019).

14. https://www.basissetexchange.org/

15. S. Jin, G. C. Haug, R. Trevino, V. D. Nguyen, H. D. Arman and O. V. Larionov, *Chem. Sci.* 2021, **12**, 13914–13921.

16. F. M. Bickelhaupt and K. N. Houk, *Angew. Chem. Int. Ed.* 2017, **56**, 10070–10086.

[17] I. Fernández, F. M. Bickelhaupt, F. P. Cossío, *Chem. Eur. J.* **2014**, *20*, 10791–10801.
